# The Early Effect of Alendronate, Hop Extract and Their Combination on Bone Structural Properties in a Rat Model of Osteoporosis

**DOI:** 10.3390/medsci14020239

**Published:** 2026-05-05

**Authors:** Edi Rođak, Robert Grgac, Rok Kostanjšek, Milorad Zjalić, Nada Oršolić, Nikola Bijelić

**Affiliations:** 1 Department of Histology and Embryology, Faculty of Medicine Osijek, University of Osijek, J. Huttlera 4, 31000 Osijek, Croatia; erodak@mefos.hr (E.R.); rgrgac@mefos.hr (R.G.); 2 Department of Medicine, University of Connecticut Health Centre, 263 Farmington Ave., Farmington, CT 06030, USA; 3 Department of Biology, Biotechnical Faculty, University of Ljubljana, Jamnikarjeva 101, 1000 Ljubljana, Slovenia; rok.kostanjsek@bf.uni-lj.si; 4 Department of Histology and Embryology, School of Medicine, University of Zagreb, Šalata 2, 10000 Zagreb, Croatia; milorad.zjalic@mef.hr; 5 Department of Animal Physiology, Faculty of Science Zagreb, University of Zagreb, Rooseveltov trg 6, 10000 Zagreb, Croatia; nada.orsolic@biol.pmf.hr

**Keywords:** alendronate, bone, hop extract, micro-CT, osteoporosis, rat

## Abstract

**Background/Objectives**: New approaches using plant-based extracts are increasingly investigated for osteoporosis treatment, including hop extract, which contains potent compounds that favorably affect bone quality. The objective of this research was to evaluate the early effect of alendronate, standardized hop extract and their combination on bone tissue quality, hematological and biochemical parameters, and markers of bone turnover in a rat model of osteoporosis. **Methods**: The study was performed on 6 month-old female Wistar rats. A sham operation was performed on 10 animals, and bilateral ovariectomy on 60 animals. Ovariectomized animals were divided into six groups, one untreated, and the others treated with a low dose of alendronate, high dose of alendronate, hop extract, or a combination of low/high dose of alendronate and hop extract. Bone quality was assessed using micro-CT imaging. Hematology and serum biochemistry were analyzed using standardized kits, and bone turnover markers were measured using Western blot. **Results**: Treatment with high-dose alendronate, hop extract, and a combination of low-dose alendronate and hop extract significantly improved bone quality in ovariectomized animals, but high-dose alendronate combined with hop extract had no favorable effect. Hematological and biochemical parameters were mostly unaffected and remained within the normal range. Changes in markers of bone turnover were modest. **Conclusions**: For the first time, combining alendronate with hop extract was analyzed. Short-term treatment with low-dose alendronate and hop extract produced effects comparable to high-dose alendronate or hop extract alone, without clinically relevant alterations in hematological or biochemical parameters. Standardized hop extract may represent a valuable adjunct in osteoporosis therapy.

## 1. Introduction

Osteoporosis (OP) is a systemic disorder affecting the skeleton, characterized by reduced bone mass and changes in the micro- and macroarchitecture of bones, most commonly caused by reduced estrogen levels [1]. OP can be classified as primary or secondary, with primary OP further divided into postmenopausal and senile types [2]. OP is associated with an increased risk of fractures due to low bone mineral density (BMD). These fractures most often occur at the hip, spine, lower arm, and shoulder. They often heal slowly, and can cause chronic pain, disability, reduced quality of life and sometimes death [3]. In addition to health effects, OP imposes a significant financial burden on various health systems [4]. Postmenopausal women are at greater risk for OP and OP-related fractures than older men due to the important role of estrogen in bone health [5]. Estrogen reduces bone turnover and resorption, decreases osteoblast apoptosis, and increases osteoclast apoptosis [6]. Although estrogen replacement therapy has been shown to be effective in alleviating OP symptoms, it has largely been abandoned due to an increased risk of breast cancer, stroke, and cardiovascular complications and has also been classified as a known human carcinogen [7,8].

Modern OP therapy includes lifestyle changes and pharmacotherapy, which is roughly divided into anabolic and antiresorptive drugs [1,9]. Anabolic drugs, such as teriparatide and abaloparatide, stimulate bone deposition and increase BMD [1]. Antiresorptive drugs slow down bone resorption and are classified into four major groups, based on their mechanism of action: bisphosphonates, selective estrogen receptor modulators (SERMs), RANKL inhibitors, and sclerostin inhibitors [10]. Bisphosphonates are the oldest class of antiresorptive drugs and are often the first line in OP treatment. These drugs bind to hydroxyapatite sites in the bone matrix, disrupt osteoclast activity in several ways, and promote osteoclast apoptosis [11]. One of the most commonly used bisphosphonates is alendronate [12]. Although still widely prescribed, alendronate has several undesirable side effects, including atypical femoral fractures, jaw osteonecrosis, atrial fibrillation, hypocalcemia, muscle pain, kidney impairment, and irritation of the esophagus and stomach [13,14,15,16].

Alendronate’s effects on bone metabolism are well established and primarily result from its toxic action on osteoclasts, leading to decreased bone resorption [9,15,17]. Estrogen’s role in bone homeostasis is multifaceted and not yet fully understood. It is known to directly induce osteoclast apoptosis, reduce RANKL expression in bone-resident T and B lymphocytes, crucial for osteoclast differentiation, and promote the production of osteoprotegerin, which further limits RANKL availability. Estrogen also suppresses cytokines that stimulate bone resorption, including IL-1, IL-6, TNF-α, M-CSF, and prostaglandins. In osteoblasts, estrogen inhibits apoptosis and extends cell lifespan through activation of the Src/Shc/ERK pathway and suppression of the JNK pathway [6,18,19]. Additionally, estrogen acts as an antioxidant and plays a central role in calcium homeostasis; its deficiency leads to negative calcium balance and bone loss in both rats and postmenopausal women [20,21,22].

In addition to classical pharmacotherapies, numerous plant-derived bioactive compounds (e.g., flavonoids, phenolic acids, stilbenes, lignans, terpenoids, alkaloids) are emerging as promising modulators of bone remodeling in osteoporosis treatment [23]. Plant-derived macro- and micronutrients support bone health by enhancing matrix formation and mineralization, with contributions from factors such as acid-base balance, potassium, fiber, calcium, magnesium, and vitamin K [20]. Recent mechanistic reviews demonstrate that some of these phytochemicals can stimulate osteoblast differentiation and/or suppress osteoclastogenesis—acting via pathways such as Wnt/β-catenin, NF-κB, SIRT1/AMPK, and RANKL/OPG [24,25,26]. Clinical and preclinical evidence support their therapeutic potential: a comprehensive review of dietary polyphenols reports benefits on bone density, inflammation, and oxidative stress [23].

Phytoestrogens are plant-derived chemical compounds that, due to their structural similarity to estradiol, exert estrogenic effects in the body [27]. The main groups of phytoestrogens are isoflavones, coumestans, lignans, stilbenes and prenylflavonoids [28,29,30]. Major dietary sources of phytoestrogens include fruits, vegetables and nuts, such as soy, garlic, carrot, potato, apple, pomegranate, and others [30]. Recently, phytoestrogens from hops (*Humulus lupulus* L.) have been increasingly investigated as potential therapies for postmenopausal symptoms, such as hot flushes, and as a promising option for osteoporosis treatment [31,32]. Due to their structural similarity to estrogens, they can activate estrogen receptor α in the bone tissue and increase BMD in humans and animals [27]. The main prenylflavonoids found in hops are xanthohumol, isoxanthohumol, 6-prenylnaringenin (6PN), and 8-prenylnaringenin (8PN) [33]. Among these, 8PN is the most potent phytoestrogen discovered to date. Although its concentration in hops and hop extracts is low, xanthohumol and isoxanthohumol are metabolized into 8PN in the liver and gut [34].

The mechanisms underlying the positive effect of hop extract on bone quality include promoting the formation and activity of osteoblasts, inhibiting the formation and activity of osteoclasts, promoting osteogenic effects while decreasing pro-osteoclastogenic differentiation of bone marrow progenitor cells, and modulating the gut microbiota [35,36,37,38,39]. To date, possible interactions and potential synergistic effects between conventional OP therapy, such as alendronate, and hop extract have not been investigated. Given the undesirable effects of alendronate therapy, it is important to consider whether using hop extract as an adjunct could reduce the frequency and severity of side effects or allow for adjustments to the regimen or dose of alendronate use in human patients. The aim of this study was to investigate the early effects of alendronate, hop extract, and their combination on bone quality using a rat ovariectomy model. Bone structural parameters were considered as the primary endpoints, while hematological, biochemical, and bone turnover markers were considered exploratory outcomes.

## 2. Materials and Methods

### 2.1. Experimental Animals and Treatment

The research was conducted in accordance with Croatian regulations of animal welfare and protection, and the Guide for the Care and Use of Laboratory Animals, DHHS (NIH) Publ # 86-23 [40]. The approvals of Croatian National Ethical Committee for animals used for scientific research (EP 233/2020, approved on 27 March 2020) and of the Committee for bioethics and animal welfare of the Faculty of Science, University of Zagreb (Class: 643-02/19-01/3, Reg. No.: 251-58-10617-19-704, approved on 17 October 2019) were obtained as well as the approval of the Ethical Committee of the Faculty of Medicine Osijek (Class: 602-04/22-08/02 Reg. No.: 2158-61-46-22-16, approved on 25 February 2022).

A total of 70 female 6-month-old Wistar rats weighing 210–280 g were used in the study. The animals were divided into 7 groups with 10 rats assigned to each group using simple manual randomization, kept in standard 12 h light/dark cycles at 24 °C and fed 4 RF 21 standard chow (Mucedola S.R.L., Settimo Milanese, Milan, Italy) ad libitum. A formal a priori sample size calculation was not performed. Group size was selected based on previous experience with similar ovariectomized rat studies, published literature, and ethical considerations related to animal use. One group underwent sham surgery and served as the healthy control (C), while the remaining six groups underwent bilateral ovariectomy to induce osteoporosis. Uterine weights were recorded at the end of the experiment to confirm successful ovariectomy. All surgical procedures were performed under intraperitoneal anesthesia using a combination of ketamine (75 mg/kg) and xylazine (10 mg/kg). Postoperative analgesia was provided with ketoprofen (2–5 mg/kg, administered intraperitoneally). Animals were maintained in standard conditions for another month to allow the osteoporosis model to develop. After this period, the ovariectomized animals were divided into treatment groups, according to the intragastric (i.g.) therapy they would receive for the next two weeks. During the recovery period after ovariectomy, two animals died (one in C group, and one in X group), resulting in a final sample size of *n* = 68. The healthy control (C) group and the ovariectomized untreated group (OV) received a daily dose of 1 mL i.g. propylene glycol (used as the hop extract solvent) and water. The other five groups were treated as follows: the ovariectomized group treated with low-dose alendronate (AL) received 1 mg/kg i.g. alendronate once daily; the ovariectomized group treated with high-dose alendronate (AH) received 2 mg/kg i.g. alendronate once daily; the ovariectomized group treated with low-dose alendronate + hop extract (AL-X) received 1 mg/kg i.g. alendronate every other day and 60 mg/kg i.g. hop extract on the days between alendronate doses; the ovariectomized group treated with high-dose alendronate + hop extract (AH-X) received 2 mg/kg i.g. alendronate every other day and 60 mg/kg i.g. hop extract on the days between alendronate doses; the ovariectomized group treated with hop extract (X) received 60 mg/kg i.g. hop extract daily.

Doses and routes were selected based on previous studies in ovariectomized rats using oral alendronate at 1–3 mg/kg and hop extracts or standardized prenylflavonoid preparations in the range of tens to a few hundred mg/kg [31,41,42,43,44]. Hop extract was not soluble in water, whereas alendronate was administered in an aqueous solution made by crushing the tablets and preparing a stock solution to ensure an equal dose between the animals. To avoid potential interactions and ensure accurate dosing in the combination groups (AL-X and AH-X), we alternated their administration on consecutive days. Additionally, alternating treatments minimized stress from repeated gastric administration while maintaining consistent daily handling. The schedule is supported by the long bone retention of alendronate and the expected bioactivity duration of hop extract, ensuring each compound can exert its effects without interference [45,46]. During each dose administration, the animals were briefly anesthetized using sevoflurane. All solutions used for i.g. dosing with flexible polypropylene feeding tubes were prepared daily as working solutions, which were used to treat the animals based on their body weight (approx. 1 mL). After two weeks of therapy, the animals were euthanized under deep anesthesia (intraperitoneal ketamine 75 mg/kg + xylazine 10 mg/kg). Blood samples were collected for hematological analysis and biochemical serum analysis. Femurs were harvested and stored in 10% neutral buffered formaldehyde solution (NBF, Gram-Mol, Zagreb, Croatia).

The hop extract, XanthoFlav™, was kindly provided by Hopsteiner (New York, NY, USA). High-performance liquid chromatography (HPLC) analysis conducted at the Hopsteiner laboratory indicated the composition of prenylflavonoids as follows: xanthohumol 75%, isoxanthohumol 0.4%, 6PN 1.7%, and 8PN 0.3%. Alendronate was administered using Aledox 70 tablets (Belupo, Koprivnica, Croatia). Sevoflurane was used as the inhalation anesthetic (Sevorane, Abbott Laboratories, Chicago, IL, USA). Ketamine (Narketan^®^, Vetoquinol S.A., Lure Cedex, France), xylazine (Xylapan^®^, Vetoquinol S.A.), and ketoprofen (Ketonal^®^, 50 mg/mL ampoules, Sandoz, Basel, Switzerland) were used for anesthesia and analgesia.

### 2.2. Micro-CT Imaging

**Figure 1:**
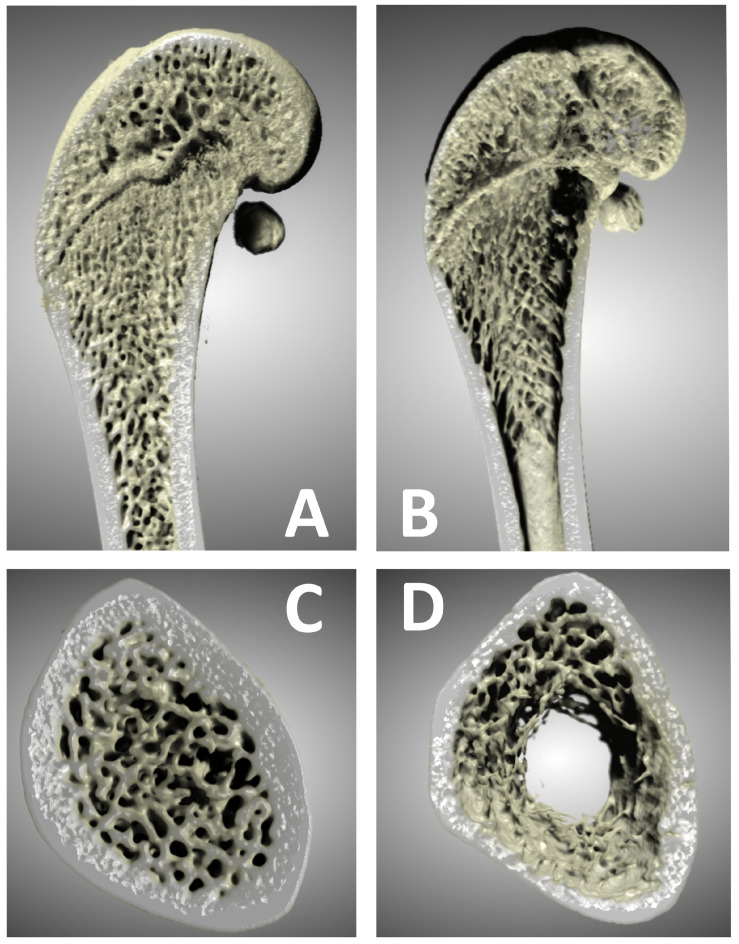
Representative 3D reconstructions of distal femora from a sham-operated control rat (**A**,**C**) and an ovariectomized, untreated rat (**B**,**D**). Panels (**A**,**B**) show sagittal section views of the bones, while panels (**C**,**D**) show a 2.5 mm-thick transverse section views of the same metaphyseal region of the bones in panels (**A**,**B**). Six-month-old rats were ovariectomized or sham-operated. One month later, animals were assigned to treatment groups for two weeks and then euthanized. Femurs were harvested and fixed in 10% neutral buffered formalin, followed by a micro-CT analysis. Notable differences in trabecular architecture are visible in these representative images.

Left femur samples from all animals were stored in 10% NBF until analysis by micro-computed tomography (micro-CT). Scanning of the distal femoral epiphyses was performed using a Neoscan N80 microtomograph (Neoscan, Mechelen, Belgium) at 50 kV, 200 µA, without an energy filter, at a resolution of 10 µm. The rotation step was set to 0.2° over 360°, with averaging set to 3. Virtual cross-sections of femoral epiphyses were reconstructed from sets of 1800 projection images of the samples using Neoscan 80 software (version 2.2.4) (Neoscan, Belgium). The distal femoral metaphysis/epiphysis was selected for analysis due to its high trabecular bone content and well-established sensitivity to ovariectomy-induced bone loss. The reconstructed images were imported into Dragonfly software (version 2021.1.0.977) (Object Research Systems Inc., Montreal, QC, Canada) for 3D reconstruction and segmentation analysis. Bone scans were denoised using a deep-learning model based on a U-net neural network within the Dragonfly software [47]. Regions of interest (ROIs) were defined using the bone analysis plugin in Dragonfly software. For each femur, a 1 cm longitudinal segment of the distal femur was analyzed, beginning at the first micro-CT slice in which the distal femoral condyles were visible and extending proximally for 10 mm along the femoral axis. This anatomical window includes the epiphysis and the adjacent metaphyseal region, capturing the full transition from subchondral trabeculae to compact cortical bone. Within this 10 mm volume, two primary ROIs were created: a total ROI, corresponding to all voxels within the periosteal boundary of the scanned segment, and a bone tissue ROI, obtained by global thresholding and including all mineralized bone. The bone tissue ROI was further segmented into a trabecular bone ROI, using the method of Buie et al. [48], in which cortical voxels are removed by morphological erosion and subsequent dilation to isolate the internal cancellous compartment, and a cortical bone ROI, defined as the remaining mineralized tissue after trabecular compartment removal. Morphometric bone parameters were then measured following the protocol established by Bouxsein et al. [49]. This approach allows quantification of both cortical and trabecular compartments across the entire distal 1 cm segment, capturing the epiphyseal–metaphyseal continuum. We note that the analyzed portion includes a thin band of primary spongiosa immediately distal to the growth plate, which should be considered when comparing our results to studies using standard metaphyseal ROIs. The following parameters were quantified: bone volume (BV), total volume (TV), bone volume fraction (BV/TV), average marrow area (Ma.Ar), average periosteal perimeter (Ps.Pm), periosteal surface (3D) (Ps.S3D), average endocortical perimeter (Ec.Pm), endocortical surface (3D) (Ec.S3D), average cortical area (Ct.Ar), average total area (Tt.Ar), average cortical area fraction (Ct.Ar/Tt.Ar), average cortical thickness (Ct.Th), average trabecular thickness (Tb.Th), and average trabecular separation (Tb.Sp). Representative 3D reconstructions of distal femora from the Dragonfly software are shown in Figure 1.

### 2.3. Hematology and Biochemical Analysis

A portion of the sampled blood was stored in heparinized BD Vacutainer^®^ containers (BD, Franklin Lakes, NJ, USA), stored at 4 °C and analyzed the following day using the ABX Micros ESV60 device (Horiba Medical, Kyoto, Japan) according to the standard rat protocol provided by the manufacturer. Standard hematological parameters were measured (the complete list of parameters is provided in the results below). The remaining blood was stored in non-heparinized BD Vacutainer^®^ containers. After coagulation, serum was separated by centrifugation at 3000× *g* for 10 min using an Eppendorf 5702 centrifuge (Eppendorf, Hamburg, Germany). A portion of the serum (100 µL) was used for biochemical analysis. Biochemical analysis was performed at the Croatian Veterinary Institute in Zagreb using the VetScan VS2 analyzer (Abaxis, Union City, CA, USA) along with the appropriate reagents and rotors (VetScan^®^ Comprehensive Diagnostic Profile; Abaxis, Union City, CA, USA). Hematology parameters included red blood cell count (RBC, ×10^12^/L), white blood cell count (WBC, ×10^9^/L), platelet count (PLT, ×10^9^/L), hemoglobin (Hb, g/L), hematocrit (Hct, L/L), mean corpuscular volume (MCV, fL), mean corpuscular hemoglobin (MCH, pg), and mean corpuscular hemoglobin concentration (MCHC, g/L). Biochemical parameters included calcium (Ca, mmol/L), phosphorus (P, mmol/L), sodium (Na, mmol/L), potassium (K, mmol/L), glucose (Glc, mmol/L), blood urea nitrogen (BUN, mmol/L), creatinine (CRE, µmol/L), alkaline phosphatase (ALP, U/L), alanine aminotransferase (ALT, U/L), total bilirubin (TBIL, mmol/L), amylase (AMY, U/L), albumin (ALB, g/L), globulins (GLO, g/L), and total protein (TP, g/L). Several samples were discarded due to hemolysis.

### 2.4. CTX1 and P1NP Western Blot

Serum markers of bone turnover, Type I collagen cross-linked C-telopeptide (CTX1) and Procollagen 1 N-terminal propeptide (P1NP) were analyzed using the Western blot technique. Total serum protein concentrations were obtained during the previously described biochemical analysis. All samples were diluted with phosphate-buffered saline (PBS) to a standardized protein concentration of 1 mg/mL and prepared for electrophoresis in Laemmli buffer. Serum proteins were separated by sodium dodecyl sulfate-polyacrylamide gel electrophoresis (SDS-PAGE) using the Hoefer Mighty Small system (Hoefer, Holliston, MA, USA). Samples were denatured at 100 °C for 5 min, cooled on ice, and stored until electrophoresis. Discontinuous gels were prepared (stacking gel: 5% acrylamide; separation gel: 10% acrylamide, 3 mm thick), incorporating trichloroethanol for stain-free normalization. Five microliters of stained protein electrophoresis standard SeeBlue 2 Plus (Thermo Fisher Scientific, Waltham, MA, USA) and 14 samples (50 µL, containing 50 µg proteins) were applied to each gel. For each bone turnover marker, five 15-well gels were used. Following electrophoresis, trichloroethanol was UV-activated using the ChemiDoc™ Imaging System (BioRad, Hercules, CA, USA). Proteins were then transferred onto polyvinylidene fluoride (PVDF) membranes (pore size 0,45 μm, Thermo Fisher Scientific, Waltham, MA, USA) using the Hoefer Mighty Small Transfer Tank system (Hoefer, Holliston, MA, USA) in Towbin transfer buffer. After the transfer, membranes were washed in PBS with 0.1% Tween 20 (PBS-T) and imaged using the ChemiDoc™ Imaging System using stain-free blot settings. The images were used for sample normalization before the analysis.

Immunodetection was performed using a mouse monoclonal primary antibody for CTX1 (Cat. No. LS-B16343, LSBio. Seattle, WA, USA) and a rabbit polyclonal primary antibody for P1NP (Cat. No. 141967, USBiological, Salem, MA, USA), both diluted 1:2000 in blocking buffer (3% BSA in PBS-T) and incubated overnight at 4 °C. After washing three times for 10 min each in PBS-T, biotinylated secondary antibodies (1:20,000) were applied for 2 h; for CTX1 membranes: goat anti-mouse polyclonal antibody (Cat. No. 115-065-071, Jackson ImmunoResearch, West Grove, PA, USA); for P1NP membranes: goat anti-rabbit polyclonal antibody (Cat. No. 111-065-144, Jackson ImmunoResearch, West Grove, PA, USA). After the incubation, membranes were washed again (three times for 10 min each in PBS-T) and treated with a tertiary complex consisting of streptavidin-horseradish peroxidase polymer (Cat. No. S2438, Sigma-Aldrich, St. Louis, MO, USA) diluted 1:1000 in PBS-T for 1 h. Membranes were then washed (3 × 10 min in PBS-T) and imaged using the ChemiDoc™ Imaging System with chemiluminescence settings. Immobilon Forte (Cat. No. WBLUF0500, Merck, Darmstadt, Germany) substrate was used for visualization. Signal intensities were compared to the stained protein standard (140 kDa for CTX1 and 53 kDa for P1NP). Multichannel images were generated and analyzed in Image Lab software v 6.1. (Bio-Rad, Hercules, CA, USA). Both stain-free and chemiluminescence images were used for quantitative analysis. The data were normalized and expressed as the ratio of the experimental group sample signal strength to the control group sample signal strength in the same blot. The resulting values were used for statistical analysis.

### 2.5. Statistical Analysis

All statistical analyses were performed using JASP (version 0.95.2) [50]. Normality was tested using the Shapiro–Wilk test. Before statistical analysis of hematology and biochemistry results, data were screened for outliers within each experimental group using Grubbs’ and Dixon’s Q tests. Data points identified as significant outliers (*p* < 0.05) were excluded from all subsequent related analyses. Differences between groups were assessed using Welch’s ANOVA. This approach was applied to all measured parameters for consistency, as Levene’s test indicated unequal variances for some variables. Post hoc comparisons were performed using the Tukey test. Statistical significance was defined as *p* < 0.05. Data are presented as box-and-whisker plots (median and interquartile range) to illustrate data distribution and variability for micro-CT and Western blot analyses, and as tables showing mean and standard deviation for biochemistry and hematology analyses. Detailed outputs from the JASP software are available as Supplementary Data.

## 3. Results

**Figure 2:**
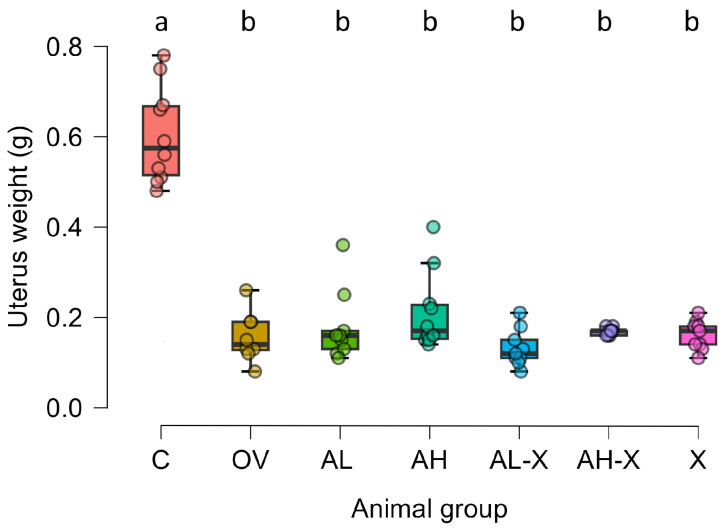
Uterine weight comparison. Six-month-old rats were ovariectomized (*n* = 60) or sham-operated (*n* = 10). One month later, animals were assigned to treatment groups for two weeks and then euthanized. Uteri were harvested and weighed. Groups: C (*n* = 10), sham-operated control group; OV (*n* = 9), untreated ovariectomized animals; AL (*n* = 10), ovariectomized animals, low alendronate dose; AH (*n* = 10), ovariectomized animals, high alendronate dose; AL-X (*n* = 10), ovariectomized animals, low alendronate dose and hop extract; AH-X (*n* = 10), ovariectomized animals, high alendronate dose and hop extract; X (*n* = 9), ovariectomized animals, hop extract. Data are shown as box-and-whisker plots (median, interquartile range, and full range). Statistical significance was assessed using Welch’s ANOVA, followed by Tukey post hoc tests. Statistically significant differences are indicated using a compact letter display (*p* < 0.001), where samples sharing the same letter are not significantly different.

Body weights did not differ significantly between the experimental groups. Uterine weights were measured at the end of the experimental period to confirm successful ovariectomy. Welch ANOVA revealed a significant difference between groups in uterine weight (*F*(6, 22.47) = 25.55, *p* < 0.001). Post hoc Tukey tests showed that all ovariectomized groups (OV, AL, AH, AL-X, AH-X, X) had significantly lower uterine weights compared with the sham-operated control group (C) (*p* < 0.001 for all comparisons). No significant differences in uterine weight were observed among the ovariectomized groups (Figure 2).

### 3.1. Micro-CT Analysis

**Figure 3:**
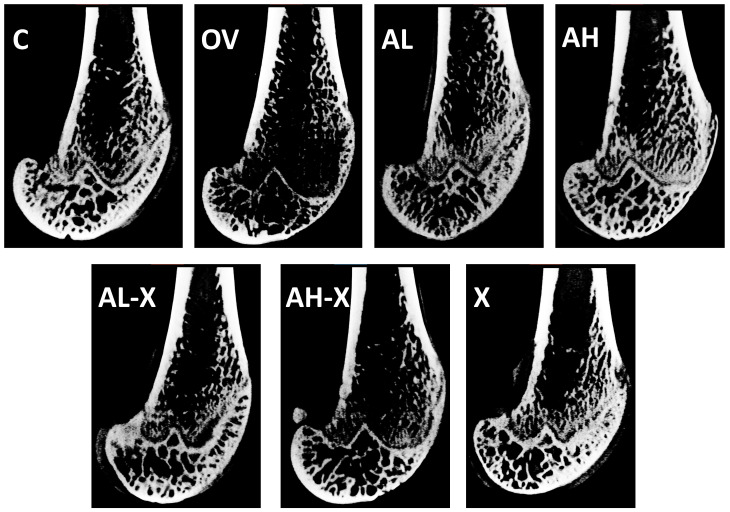
Representative sagittal scans of distal femora across the groups. Six-month-old rats were ovariectomized (*n* = 60) or sham-operated (*n* = 10). One month later, animals were assigned to treatment groups for two weeks and then euthanized. Femurs were harvested and fixed in 10% neutral buffered formalin, followed by a micro-CT analysis. Groups: C, sham-operated control group; OV, untreated ovariectomized animals; AL, ovariectomized animals, low alendronate dose; AH, ovariectomized animals, high alendronate dose; AL-X, ovariectomized animals, low alendronate dose and hop extract; AH-X, ovariectomized animals, high alendronate dose and hop extract; X, ovariectomized animals, hop extract.

All measured parameters describing changes within the distal epiphysis of the femur presented below have been categorized into general bone measurements, periosteal and endocortical measurements, cortical bone measurements, and trabecular bone measurements. Examples of bone scans from each group are shown in Figure 3.

**Figure 4:**
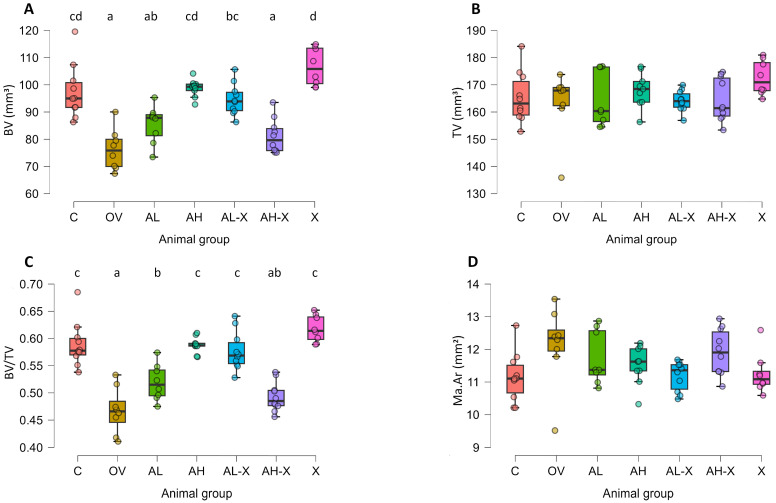
Results of analyses of general bone tissue measurements. The figure shows bone volume (BV) (**A**), total volume (TV) (**B**), bone volume fraction (BV/TV) (**C**), and average marrow area (Ma.Ar) (**D**). Six-month-old rats were ovariectomized (*n* = 60) or sham-operated (*n* = 10). One month later, animals were assigned to treatment groups for two weeks and then euthanized. Femurs were harvested and fixed in 10% neutral buffered formalin for micro-CT analysis. Groups: C (*n* = 10), sham-operated control group; OV (*n* = 9), untreated ovariectomized animals; AL (*n* = 10), ovariectomized animals, low alendronate dose; AH (*n* = 10), ovariectomized animals, high alendronate dose; AL-X (*n* = 10), ovariectomized animals, low alendronate dose and hop extract; AH-X (*n* = 10), ovariectomized animals, high alendronate dose and hop extract; X (*n* = 9), ovariectomized animals, hop extract. Data are shown as box-and-whisker plots (median, interquartile range, and full range). Statistical significance was assessed using Welch’s ANOVA, followed by Tukey post hoc tests. Statistically significant differences are indicated using a compact letter display (*p* < 0.05), where samples sharing the same letter are not significantly different. Absence of letter annotations indicates no statistically significant differences between groups.

The results of the general bone measurement analysis are shown in Figure 4. The total volume of the measured part of the femur was consistent across groups. Significant differences between groups were noticed for bone volume (*F*(6, 26.3) = 19.82911, *p* < 0.001) and bone volume fraction (*F*(6, 26.4) = 26.43182, *p* < 0.001) (Figure 4A,C). The bone volume and bone volume fraction were significantly reduced in the OV group compared to the C group, as expected from the experimental model. They were also significantly lower compared to the AH, AL-X and X group. Additionally, they were significantly lower in the AH-X group compared to the C, AH and X groups. The AL group had a lower bone volume fraction compared to the X group. For all the mentioned differences in BV and BV/TV, *p* was < 0.001, except for C vs. AL (BV *p* = 0.005), AL-X vs. AH-X (BV *p* = 0.003), AL-X vs. X (BV *p* = 0.03), OV vs. AL (BV/TV *p* = 0.03) and AL vs. AL-X (BV/TV *p* = 0.006). Therefore, the highest BV and BV/TV were noted in the C, AH, AL-X and X groups, and the lowest in the OV and AH-X groups. Average marrow area did not differ significantly between the groups. For detailed post hoc test results and all *p*-values, please see JASP output in the Supplementary Material (Table S1).

**Figure 5:**
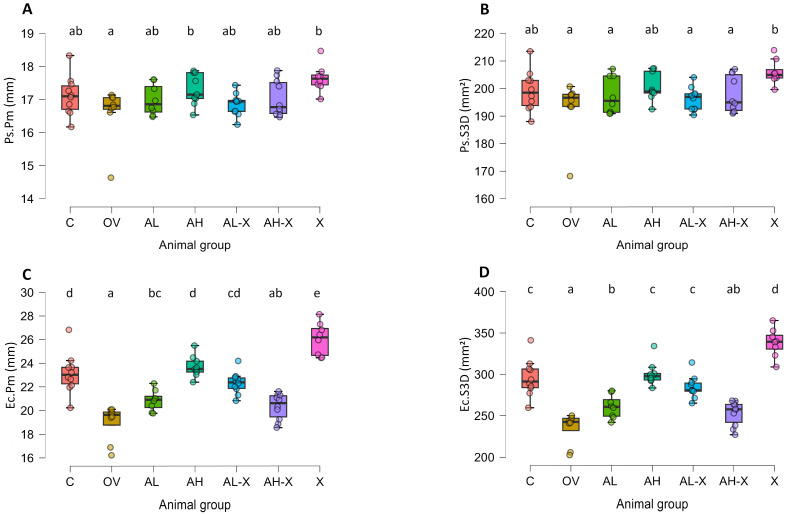
Results of periosteal and endocortical measurements. The figure shows average periosteal perimeter (Ps.Pm) (**A**), periosteal surface (3D) (Ps.S3D) (**B**), average endocortical perimeter (Ec.Pm) (**C**), and endocortical surface (3D) (Ec.S3D) (**D**). Six-month-old rats were ovariectomized (*n* = 60) or sham-operated (*n* = 10). One month later, animals were assigned to treatment groups for two weeks and then euthanized. Femurs were harvested and fixed in 10% neutral buffered formalin for micro-CT analysis. Groups: Groups: C (*n* = 10), sham-operated control group; OV (*n* = 9), untreated ovariectomized animals; AL (*n* = 10), ovariectomized animals, low alendronate dose; AH (*n* = 10), ovariectomized animals, high alendronate dose; AL-X (*n* = 10), ovariectomized animals, low alendronate dose and hop extract; AH-X (*n* = 10), ovariectomized animals, high alendronate dose and hop extract; X (*n* = 9), ovariectomized animals, hop extract. Data are shown as box-and-whisker plots (median, interquartile range, and full range). Statistical significance was assessed using Welch’s ANOVA, followed by Tukey post hoc tests. Statistically significant differences are indicated using a compact letter display (*p* < 0.05), where samples sharing the same letter are not significantly different. Absence of letter annotations indicates no statistically significant differences between groups.

The results of periosteal and endocortical measurements are shown in Figure 5. Significant differences between groups were noticed for all parameters; however, changes in periosteal measurements were relatively small compared to endocortical (average periosteal perimeter (*F*(6, 26.3) = 2.59984, *p* = 0.04), periosteal surface (3D) (*F*(6, 26.2) = 3.59494 *p* = 0.01), average endocortical perimeter (*F*(6, 26.3) = 27.33963, *p* < 0.001), and endocortical surface (3D) (*F*(6, 26.5) = 37.49222, *p* < 0.001)). Subsequent Tukey post hoc comparisons for Ps.Pm identified statistically significant differences only for OV vs. AH (*p* = 0.04) and OV vs. X (*p* = 0.005). Ps.S3D was significantly higher in the X group than in the OV (*p* < 0.001), AL (*p* = 0.03), AL-X (*p* = 0.01) and AH-X groups (*p* = 0.04). Ec.Pm and Ec.S3D were significantly higher in the X group compared to all other groups (*p* < 0.001 for all cases except for AH vs. X Ec.Pm, *p* = 0.02). Furthermore, AH and AL-X had comparable values to the C group, significantly higher than the other three groups. The AL group had slightly higher values than the OV group (*p* = 0.03 (Ec.Pm) and 0.02 (Ec.S3D)), and for AH-X, they were similar to the OV group. For detailed post hoc test results and all *p*-values, please see JASP output in the Supplementary Material (Table S1).

**Figure 6:**
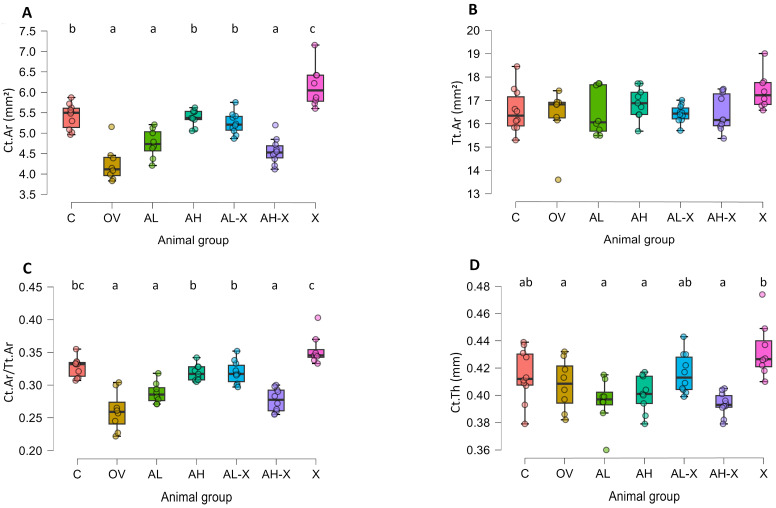
Results of cortical bone tissue measurements. The figure shows average cortical area (Ct.Ar) (**A**), average total area (Tt.Ar) (**B**), average cortical area fraction (Ct.Ar/Tt.Ar) (**C**), and average cortical thickness (Ct.Th) (**D**). Six-month-old rats were ovariectomized (*n* = 60) or sham-operated (*n* = 10). One month later, animals were assigned to treatment groups for two weeks and then euthanized. Femurs were harvested and fixed in 10% neutral buffered formalin for micro-CT analysis. Groups: C (*n* = 10), sham-operated control group; OV (*n* = 9), untreated ovariectomized animals; AL (*n* = 10), ovariectomized animals, low alendronate dose; AH (*n* = 10), ovariectomized animals, high alendronate dose; AL-X (*n* = 10), ovariectomized animals, low alendronate dose and hop extract; AH-X (*n* = 10), ovariectomized animals, high alendronate dose and hop extract; X (*n* = 9), ovariectomized animals, hop extract. Data are shown as box-and-whisker plots (median, interquartile range, and full range). Statistical significance was assessed using Welch’s ANOVA, followed by Tukey post hoc tests. Statistically significant differences are indicated using a compact letter display (*p* < 0.05), where samples sharing the same letter are not significantly different. Absence of letter annotations indicates no statistically significant differences between groups.

The analysis of changes in cortical bone is shown in Figure 6. Significant differences between groups were noticed for average cortical area (*F*(6, 26.2) = 20.13942, *p* < 0.001), average cortical area fraction (*F*(6, 26.4) = 20.36143, *p* < 0.001) and average cortical thickness (*F*(6, 25.9) = 6.61544, *p* < 0.001) (Figure 6A,C,D). The average total (cortical + marrow) area did not differ significantly between groups (Figure 6B). Ct.Ar and Ct.Ar/Tt.Ar were significantly higher in the C, AH, AL-X and X groups compared to other groups (almost all cases had *p* < 0.001). Furthermore, compared to the AH and AL-X groups, the X group exhibited a significantly higher Ct.Ar (*p* values 0.008, <0.001, respectively) and Ct.Ar/Tt.Ar (*p* values 0.04, 0.01, respectively), and it also had a higher Ct.Ar than the C group (*p* = 0.004). Ct.Th was significantly higher in the X group compared to the OV, AL, AH and AH-X groups (*p* values 0.02, <0.001, 0,02, <0.001, respectively). For detailed post hoc test results and all *p*-values, please see JASP output in the Supplementary Material (Table S1).

**Figure 7:**
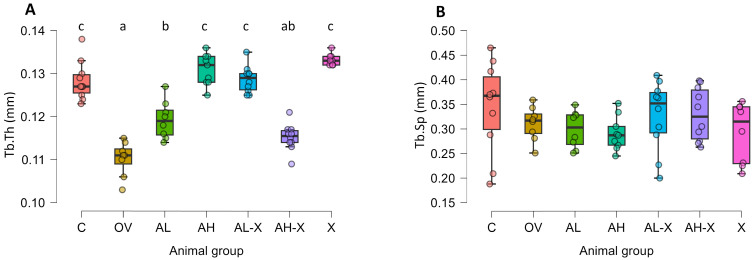
Results of trabecular bone tissue measurements. The figure shows average trabecular thickness (Tb.Th) (**A**) and average trabecular separation (Tb.Sp) (**B**). Six-month-old rats were ovariectomized (*n* = 60) or sham-operated (*n* = 10). One month later, animals were assigned to treatment groups for two weeks and then euthanized. Femurs were harvested and fixed in 10% neutral buffered formalin for micro-CT analysis. Groups: C (*n* = 10), sham-operated control group; OV (*n* = 9), untreated ovariectomized animals; AL (*n* = 10), ovariectomized animals, low alendronate dose; AH (*n* = 10), ovariectomized animals, high alendronate dose; AL-X (*n* = 10), ovariectomized animals, low alendronate dose and hop extract; AH-X (*n* = 10), ovariectomized animals, high alendronate dose and hop extract; X (*n* = 9), ovariectomized animals, hop extract. Data are shown as box-and-whisker plots (median, interquartile range, and full range). Statistical significance was assessed using Welch’s ANOVA, followed by Tukey post hoc tests. Statistically significant differences are indicated using a compact letter display (*p* < 0.05), where samples sharing the same letter are not significantly different. Absence of letter annotations indicates no statistically significant differences between groups.

Structural differences in the trabecular bone are shown in Figure 7. Significant differences between groups were observed in average trabecular thickness (*F*(6, 26.5) = 51.20497, *p* < 0.001, Figure 7A), while average trabecular separation did not differ significantly between the groups (Figure 7B). Tb.Th in the OV group was significantly lower compared to all groups, except for AH-X, and significantly larger in C, AH, AL-X and X than all other groups (all *p* values < 0.001). For detailed post hoc test results and all *p*-values, please see JASP output in the Supplementary Material (Table S1).

### 3.2. Hematology and Serum Biochemistry

#### 3.2.1. Hematology

To assess the general impact of the tested substances on animals, different parameters from whole blood or serum were analyzed. Several samples were discarded due to hemolysis. Table 1 shows the results of hematology tests. Although erythrocyte counts were higher in all groups compared to the control group, only the increase in the AL-X group was statistically significant (*p* = 0.006) compared to the control, as well as to the X group (*p* = 0.04), with values for C and X being very similar. Hematocrit values were also significantly higher in AL-X compared to the C and X group (*p* value 0.002 and 0.03, respectively). Results for hemoglobin values were slightly different, as the AL and AL-X groups had significantly higher hemoglobin values than controls (*p* = 0.004 and 0.001, respectively). However, although Welch’s ANOVA found a significant difference in the MCH, Tukey’s post hoc test did not yield any significant differences between groups. On the other hand, MCHC was significantly higher in the AH group than in the C and OV groups (*p* = 0.046 and 0.048, respectively). MCV did not differ significantly among the groups.

Regarding leukocyte counts, Welch’s ANOVA indicated a significant difference among groups (*F*(6, 22.1) = 4.32, *p* = 0.005). However, pairwise comparisons using Tukey’s post hoc test did not identify any significant differences between individual groups. Platelet counts were significantly reduced in all groups receiving alendronate, when compared to controls (AL (*p* = 0.04), and AH, AL-X, and AH-X (*p* < 0.001)), and a high dose of alendronate accounted for the lowest values.

#### 3.2.2. Electrolytes, Renal and Metabolic Parameters

Table 2 presents the results of electrolytes, renal and metabolic parameters, while parameters describing liver function, protein metabolism, and enzymes can be found in Table 3. No significant changes were observed in calcium, phosphate, sodium, potassium, glucose, blood urea nitrogen, and creatinine levels.

#### 3.2.3. Liver Function, Protein Metabolism, and Enzymes

No significant differences were found for alkaline phosphatase, alanine aminotransferase, total bilirubin, amylase, and globulins. Albumin concentrations were lower across all ovariectomized groups compared to the control, and although Welch’s ANOVA reported significant differences, Tukey’s post hoc test did not show any significant differences between groups except for a borderline value for comparison of the C and OV groups (*p* = 0.05). Total serum protein concentrations were lower in all ovariectomized groups compared to the control, with statistically significant reductions in the OV and AH-X groups (*p* = 0.02 and 0.007, respectively).

### 3.3. CTX1 and P1NP Western Blot Analysis

**Figure 8:**
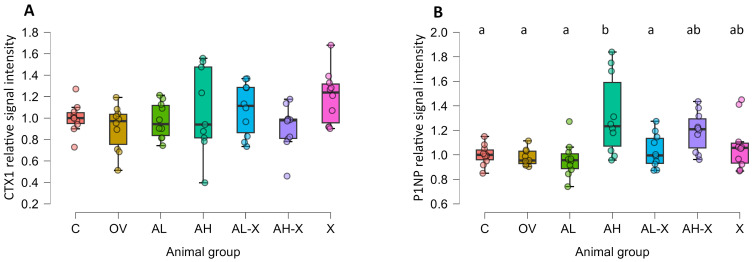
Results of Western blot analyses. The figure shows relative signal intensity for the C-terminal cross-linked telopeptide of type 1 collagen (CTX1) (**A**), and N-terminal propeptide of type 1 procollagen (P1NP) (**B**). Six-month-old rats were ovariectomized (*n* = 60) or sham-operated (*n* = 10). One month later, animals were assigned to treatment groups for two weeks and then euthanized. Blood was collected for hematology and serum biochemistry; a portion of serum was used for Western blot. Groups: C (*n* = 10), sham-operated control group; OV (*n* = 9), untreated ovariectomized animals; AL (*n* = 10), ovariectomized animals, low alendronate dose; AH (*n* = 10), ovariectomized animals, high alendronate dose; AL-X (*n* = 10), ovariectomized animals, low alendronate dose and hop extract; AH-X (*n* = 10), ovariectomized animals, high alendronate dose and hop extract; X (*n* = 9), ovariectomized animals, hop extract. Data are shown as box-and-whisker plots (median, interquartile range, and full range). Statistical significance was assessed using Welch’s ANOVA, followed by Tukey post hoc tests. Statistically significant differences are indicated using a compact letter display (*p* < 0.05), where samples sharing the same letter are not significantly different. Absence of letter annotations indicates no statistically significant differences between groups.

**Figure 9:**
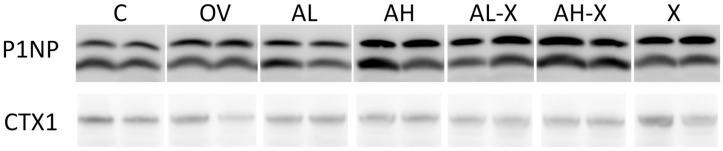
Digital photographs of representative membranes obtained during the Western blot analysis of serum markers of bone turnover. Abbreviations: CTX1, C-terminal cross-linked telopeptide of type 1 collagen; P1NP, N-terminal propeptide of type 1 procollagen; C (*n* = 10), sham-operated control group; OV (*n* = 9), untreated ovariectomized animals; AL (*n* = 10), ovariectomized animals, low alendronate dose; AH (*n* = 10), ovariectomized animals, high alendronate dose; AL-X (*n* = 10), ovariectomized animals, low alendronate dose and hop extract; AH-X (*n* = 10), ovariectomized animals, high alendronate dose and hop extract; X (*n* = 9), ovariectomized animals, hop extract. Lanes were digitally reordered for consistency with other data presentations.

The intensity of signals for the analyzed serum markers of bone remodeling, CTX1 and P1NP, was quantified from digital photographs of membranes. The obtained intensity was normalized and expressed relative to the C group. The results were analyzed using Welch’s ANOVA test with Tukey post hoc test for group comparisons. The results are shown in Figure 8. No significant differences were observed for CTX1, while some were found for P1NP (CTX1: *F*(6, 27.1) = 1.720, *p* = 0.15; P1NP: *F*(6, 27.2) = 3.872, *p* = 0.006). In the AH, P1NP levels were significantly higher compared to most groups (C, OV, AL, AL-X). For detailed post hoc test results and all *p*-values, please see JASP output in the Supplementary Material (Table S3). Digital photographs of the membranes are shown in Figure 9.

## 4. Discussion

This study investigated the early effects of a short-term treatment with low and high doses of alendronate, standardized hop extract, and their combinations on an ovariectomized rat model of osteoporosis.

Previous studies have shown that hop extract can exert effects on trabecular bone comparable to estradiol, and pure 8-prenylnaringenin (8PN) can improve BMD [31,32]. In this study, hop extract significantly improved bone volume in the distal femoral epiphysis, comparable to high-dose alendronate. Both treatments restored trabecular bone volume and volume fraction toward control levels. Similarly, the combination of low-dose alendronate and hop extract yielded comparable preservation of bone mass. In contrast, the combination of high-dose alendronate and hop extract resulted in reduced bone mass, nearly equivalent to OV animals.

### 4.1. Changes in Bone Parameters

Periosteal and endosteal measurements indicate that hop extract, high-dose alendronate, and the combination of low-dose alendronate with hop extract promote endosteal bone apposition but do not significantly affect bone width. These findings are consistent with prior research showing that estrogen stimulates endosteal apposition without influencing periosteal expansion in female rodents [51]. While some studies found no effect of hop extract on cortical bone thickness or quality [31], we observed increased cortical thickness and surface area when compared to ovariectomized controls. Hop extract maintained cortical bone surface similar to that of the control group, matching the outcomes of both high-dose alendronate and the low-dose alendronate–hop extract combination. However, the high-dose combination (AH-X group) did not show benefits, with cortical bone levels similar to the untreated OV group. Hop extract alone showed effects comparable to high-dose alendronate in preserving trabecular thickness, aligning with earlier studies [31], as was the low-dose combination. Again, the AH-X group showed markedly inferior bone quality, comparable to the OV group.

The results in the X group confirm previously described beneficial effects of hop-derived phytoestrogens on bone quality. Notably, hop extract alone produced effects similar to those observed with high-dose alendronate. This finding warrants further investigation, especially in the context of human use. However, it remains unclear why combining high-dose alendronate and hop extract failed to replicate the positive effects observed with either treatment alone. One hypothesis is that excessive osteoclast suppression might impair osteoblast activation. While alendronate induces osteoclast apoptosis via the mevalonate pathway [52], bone resorption persists to some extent, releasing cytokines and matrix-derived factors (e.g., TGF-β, IGF1, IGF2, BMPs) that promote osteoblast differentiation and activity [6,53]. These molecules, along with juxtacrine and paracrine signals from osteoclasts, are essential for osteoblast function. Estrogen and phytoestrogens binding to ERα on osteoclasts also reduce their activity and differentiation [54,55]. It is possible that hop extract and alendronate affect osteoclasts via distinct mechanisms that combine to excessively suppress osteoclast activity, inadvertently hindering osteoblast signaling and function. In addition to alendronate-induced osteoclast apoptosis, 8PN inhibits osteoclast differentiation via suppression of RANKL expression, a key regulator of osteoclast differentiation [54]. This dual effect could reduce osteoclast activity below the threshold required for adequate osteoblast activation, which may contribute to the decreased bone mass observed in the AH-X group. In contrast, in the AL-X group, supratherapeutic doses of alendronate with hop extract may result in a level of osteoclast inhibition that, despite acting through both apoptosis and RANKL suppression, remains sufficient to permit osteoblast activation through clastokine secretion. Another consideration is that high doses of alendronate increase the number of osteoclasts; although bone resorption is slowed under these conditions, these osteoclasts could still release TRAP and other molecules affecting bone metabolism [56]. Another study reported that alendronate and other nitrogen bisphosphonates can provoke short-term inflammatory responses and increased cytokine expression (IL-6, TNF-α) [57], which could facilitate osteoclastogenesis despite the inhibitory and pro-apoptotic effects of bisphosphonates on osteoclasts. As no negative effects were observed in the AH group, there is clearly a missing link related to hop extract administration. Recent studies and reviews document that flavonoids can act as antioxidants or pro-oxidants depending on dose, the presence of redox-active transition metals (Fe, Cu), and different cellular conditions [58,59,60]. There is no evidence that alendronate directly induces flavonoid pro-oxidation, but alendronate-related changes in inflammation or tissue redox balance could, in principle, modify the microenvironment in ways that influence flavonoid redox behavior; however, this is speculative and should be tested in future studies. In the context of osteoporosis therapy, this outcome is likely not concerning, as the high alendronate dose used in the AH and AH-X groups exceeded therapeutic levels. Notably, the combination of low-dose alendronate and hop extract was as effective as high-dose alendronate in improving cortical and trabecular bone quality. This suggests that hop extract may have potential as an adjunct to alendronate therapy. However, all of the aforementioned mechanistic interpretations should be regarded as hypothesis-generating and require confirmation in studies incorporating direct cellular and histological analyses, particularly to clarify the effects observed in the AH-X group through investigation of potential alterations in osteoclast–osteoblast interactions.

### 4.2. Hematology and Serum Parameters

RBC counts were higher in all ovariectomized groups compared to controls, but this increase reached statistical significance only in the AL-X group. Regardless of significance, RBC counts remained within the reference range for rats, indicating that alendronate, hop extract, or their combination had no clinically relevant effect on erythrocyte numbers. Hematocrit and hemoglobin values showed similar patterns. Estrogen modulates intracellular erythrocyte signaling, which may be relevant in the context of phytoestrogen therapy as selective ERα modulators [61]. Additionally, estrogen is involved in the regulation of the proliferation, self-renewal, and differentiation of hematopoietic cells, suggesting that observed blood changes could reflect potential alterations in the bone marrow [62,63]. Although not observed in this study, alendronate has been reported to increase leukocyte counts (monocytes and neutrophils) in rats [21]. Future studies should investigate the potential effects of the tested compounds on bone marrow hematopoietic cells and assess leukocyte differential counts in addition to total counts with longer treatment duration.

Platelet counts were reduced in all ovariectomized groups, particularly in alendronate-treated animals (significant in AH, AL-X, and AH-X groups), but values remained within the reference range. This is consistent with findings that platelet counts decline after menopause [64]. Some studies report higher platelet counts in individuals with osteopenia or osteoporosis compared to those with normal BMD [65,66], while others report lower counts with reduced BMD [67]. Platelets and their precursors (megakaryocytes) express estrogen receptors; however, the influence of estrogen on platelet formation and function remains unclear [68]. Although no clinically significant hematologic alterations were observed in this study, future research should examine the long-term effects of these compounds on blood parameters and hematopoietic cells.

Serum biochemical parameters were also analyzed. No significant differences were found in measured electrolytes, although sodium levels were at or slightly below the lower reference limit in all groups, possibly representing an experimental artifact. Serum ALT levels did not differ significantly. Research linking liver enzyme levels to osteoporosis is somewhat conflicting, making these results difficult to interpret in the context of current knowledge [69,70]. Alendronate rarely causes hepatotoxicity [71]. Total bilirubin, as another marker of liver function, showed no significant differences between groups. Urea and creatinine levels were similar across groups, indicating no impairment of renal function during the treatment period, consistent with reports that even the long-term alendronate does not affect renal function in patients [13]. Increased serum ALP following OVX is a well-known phenomenon, and estrogen therapy does not significantly reduce ALP levels in ovariectomized sheep [72]. Our results were consistent with this trend, although differences were not statistically significant, possibly due to variability within groups. Serum amylase, as a marker of pancreatic exocrine function, showed no significant changes.

Total protein and albumin levels were at the upper reference limit or slightly above in all groups and were lower in all ovariectomized groups compared to controls, though not significantly, except for total protein in the OV and AH-X groups. Globulin levels did not differ significantly. These findings partially align with studies reporting reduced albumin levels in osteoporosis patients [73]. They are also consistent with results from another study that found total protein reduction in the serum of ovariectomized rats [74]. Alendronate and hop extract seem to partly mitigate this effect, at least in early stages of the treatment. Hop extract might have a positive effect due to the protective action of 8PN on liver metabolism, which has already been demonstrated for glucose and lipid metabolism [75,76], but further clarification is needed regarding its effects on protein synthesis and metabolism. Interestingly, one study reported that isoflavone therapy combined with exercise restored protein expression in liver tissues to normal after it was reduced in ovariectomized rats [77]. The effect of alendronate may be indirect, through the reduction in serum inflammatory cytokines [78], or through another pathway, but the actual mechanisms for both substances should be further investigated. Future research should also examine the effects of these compounds on individual plasma protein fractions in more detail.

### 4.3. CTX1 and P1NP Markers of Bone Metabolism

After the two-week treatment period with alendronate, hop extract, or their combination, no indicators of adverse effects on serum biochemical parameters were observed. However, the long-term impact of these interventions warrants further investigation.

Western blot analysis of bone turnover markers showed significantly elevated P1NP levels in the AH group compared to the C, OV, AL, and AL-X groups, indicating a dose-dependent anabolic effect of alendronate. This is consistent with previous findings demonstrating that high-dose alendronate stimulates osteoblast differentiation via mesenchymal progenitors [79]. CTX1 levels, a marker of bone resorption, did not significantly differ among groups, despite expectations of increased CTX1 in ovariectomized rats. CTX1 levels can be affected by circadian rhythm, feeding state, and other factors [80]. Since samples were collected 30 days post-ovariectomy, when the model was already established, it is plausible that CTX1 levels had peaked earlier and then stabilized. Uniform CTX1 levels across groups may suggest no additional resorption, although this interpretation is limited by the semi-quantitative nature of the method.

According to Song et al., CTX1 levels stabilize at elevated levels post-ovariectomy, while P1NP levels decline, although their study used mice and ELISA rather than Western blot [81]. A high-dose combination of red clover and hop extracts reduced serum CTX1 in one study [82], while soy isoflavones decreased both CTX1 and P1NP in another [83]. However, such effects were not observed here, which might be due to the Western blot not being precise enough to show subtle differences between groups. Future studies should monitor CTX1 and P1NP levels at regular intervals following ovariectomy and during long-term treatment to better understand biomarker dynamics.

### 4.4. Translational Relevance and Phytoestrogen Context

As previously noted, numerous phytoestrogens offer protective effects on bone. In vitro and in vivo data on 8PN and hop extract, which contains 8PN and its precursors, support their role in preventing bone loss and enhancing bone formation [31,32,84]. Since hop extract and purified 8PN may have fewer side effects than current osteoporosis drugs, their therapeutic potential is under active investigation [31,32]. However, the long-term safety of hop-derived phytoestrogens remains incompletely understood [37].

Pharmacokinetically, 8PN has a higher affinity for ERα than ERβ, classifying it as a SERM. In vitro, its estrogenic activity matches that of endogenous estrogens (e.g., estrone), while its in vivo effect on reproductive tissues is about 20,000 times weaker than estradiol [85]. Although some researchers have reported estrogenic effects of hop extract on reproductive organs, these effects are generally much weaker than those of estrogen and are probably dose- and route- dependent [39,76]. In line with this, our previously published results show that neither of the therapeutics used affected the uterine endometrium [86]. These findings confirm previous reports that 8PN is selective and does not show estrogenic effects on various estrogen-dependent organs, such as uterus, in which estrogenic effects might contribute to malignancies [31,32].

This study generated valuable data on the early effects of a short-term treatment with high and low doses of alendronate, hop extract, and their combinations on bone quality and remodeling, as well as hematological and biochemical blood indices, using the ovariectomized rat model of osteoporosis. The previously reported beneficial effects of alendronate and hop extract were generally supported. However, for the first time, data were obtained following the administration of combined high or low doses of alendronate with hop extract. The combination of a low dose of alendronate with hop extract produced effects comparable in magnitude to those observed with high-dose alendronate or hop extract monotherapy. However, this effect cannot be attributed with certainty to a synergistic or additive effect of hop extract, as the combination groups received less total alendronate than the monotherapy groups. Additionally, the observed differences between combination and monotherapy groups may be partially influenced by the reduced dosing frequency of alendronate. These findings warrant further investigation into the therapeutic potential of lower alendronate doses in combination with hop extract, as this approach may reduce the incidence and severity of adverse effects associated with alendronate therapy.

Hop extract alone demonstrated effects comparable to, and in some parameters numerically greater than those observed with alendronate on the evaluated bone parameters. This observation for hop extract monotherapy is consistent with pre-clinical and clinical studies that report beneficial effects of hop extract or 8PN in both rodents and humans. Recent in vitro and in vivo research has reported the beneficial effects of hop extract on the osteogenic potential of stem cells, bone microarchitecture, and biomechanical parameters in rats [31,38,39,87,88]. A review of the literature also confirms that 8PN from hop extract prevents osteoporosis by promoting osteoblast differentiation and inhibiting osteoclast activity [37]. This is noteworthy given that 8PN is considered among the most potent phytoestrogens identified to date. Human studies on hop extract and bone quality are rare; however, a study by Lecomte et al. reported favorable outcomes in BMD in postmenopausal women after 48 weeks of hop extract use [35]. Additionally, a meta-analysis of non-hop isoflavone usage showed beneficial effects on BMD and safe usage in postmenopausal women (primarily genistein and a synthetic isoflavone) [89]. A comprehensive review on the effects of different phytoestrogens on bone health and estrogen metabolism in animals and humans was prepared by Tomczyk-Warunek et al., highlighting the potential of phytoestrogens in osteoporosis prevention [27]. More studies on the effect of hop-extract phytoestrogens in humans are needed. In addition to its possible therapeutic application in osteoporotic patients, 8PN may serve as a promising lead compound for the development of more potent derivatives for therapeutic purposes, analogous to stilbene-based SERMs already approved for clinical use, including raloxifene, toremifene, and tamoxifen, which have demonstrated efficacy in the treatment and prevention of postmenopausal osteoporosis and hormone-receptor positive cancers (e.g., breast cancer) [90]. Nevertheless, some researchers also promote the notion that dietary interventions might be a better approach than a single-compound treatment, as there are multiple bioactive ingredients in plant extracts [27]. For example, recent research shows that bitter acids from hops also have a protective effect against OP, which might be an added value [91]. This suggests there is a potential for developing functional foods for osteoporosis prevention and treatment containing standardized hop and other plant extracts. Hematological and biochemical parameters remained largely within physiological ranges across all groups, with no evidence of clinically relevant adverse effects following treatment with alendronate, hop extract, or their combination. These findings suggest that the tested interventions were well tolerated under the conditions of this short-term study.

### 4.5. Limitations

There are several important limitations to our experimental design that should be considered.

First, the two-week treatment period is relatively short compared to typical ovariectomy studies, so our findings are likely to reflect early rather than sustained effects of the interventions. Nevertheless, prenylflavonoids from hop extract have been shown in rodents to achieve tissue exposure and bone-protective activity within four weeks [32]. Rats’ higher bone turnover compared to humans means that short-term dosing can model some aspects of human postmenopausal bone loss within a condensed timeframe. While longer-term studies are necessary, our data provide initial evidence that hop-derived phytoestrogens alone or in combination with alendronate can rapidly modulate bone remodeling. In terms of translation to humans, 2 weeks in adult rats roughly corresponds to one year, which is consistent with clinical observations of BMD improvement after 48 weeks and menopause-related symptom relief after 12 weeks of hop extract [35,92,93]. Nevertheless, visible changes after just two weeks of treatment provide valuable insight into the effectiveness of the tested treatments.

Second, the study relied on a single dose of hop extract (60 mg/kg), which limits assessment of dose–response relationships, limiting knowledge of the optimal dose range and its extrapolation to human doses. Future studies should address an expanded hop-extract dose range in combination with alendronate. Additionally, alendronate was administered on alternate days in combination groups, but daily in monotherapy groups, resulting in lower cumulative alendronate exposure in the combination groups. Consequently, differences observed between monotherapy and combination treatments cannot be unequivocally attributed to the addition of hop extract, as they may be partially influenced by differences in dosing frequency. Future studies incorporating matched dosing schedules and appropriate control groups (e.g., alternate-day alendronate without hop extract) are required. The absence of sham-operated animals receiving alendronate or hop extract limits the ability to determine whether the observed effects are specific to the osteoporotic condition or also occur in animals with normal bone mass. Inclusion of such groups would help clarify whether these interventions primarily prevent bone loss or exert anabolic effects independent of estrogen deficiency.

Third, BMD is a standard endpoint in osteoporosis studies. Although a calibration phantom was used, the high-throughput scanning necessary to accommodate all samples introduced signal variability and noise, preventing reliable BMD quantification. Nevertheless, structural parameters such as BV/TV, trabecular thickness, and connectivity were robustly measured and provide meaningful insight into bone architecture. Unfortunately, this limitation prevents direct comparability with the majority of published studies in this field, and the results should therefore be interpreted with caution. Additionally, the study is the evaluation of a single skeletal site. Although the distal femur is a well-established and sensitive region for detecting OVX-induced trabecular bone loss, bone responses are known to be site-specific. Therefore, inclusion of additional clinically relevant sites, such as the femoral neck and lumbar spine, would provide a more comprehensive assessment of skeletal effects.

Fourth, circulating bone turnover markers (CTX and P1NP) were assessed by Western blotting. While this method allows detection of relative changes, it is semi-quantitative and less precise than validated immunoassay platforms such as ELISA, ECLIA, or CLIA, and may lack sensitivity for detecting subtle differences in circulating levels. Consequently, we cannot provide standardized bone turnover marker concentrations, and future studies using dedicated immunoassays are warranted to confirm these findings.

Fifth, we did not directly assess osteoclast or osteoblast activity and regulation. Consequently, while we analyzed structural changes and biomarkers, the precise contribution of osteoclast inhibition versus osteoblast activation is missing. Consequently, the proposed mechanisms underlying the observed effects, particularly for the AH-X combination, remain speculative. Future studies incorporating histological and histomorphometric endpoints (e.g., TRAP staining, osteoblast number, osteoid surface) are required to clarify the cellular basis of these findings. Incorporating these cellular and molecular endpoints would strengthen mechanistic insights into the observed bone effects.

Finally, due to limited and heterogeneous data on the subject matter, this study was primarily designed as exploratory in nature. Therefore, findings related to hematological, biochemical, and bone turnover markers should be interpreted as such and require additional confirmation.

### 4.6. Conclusions

Well-controlled pre-clinical and clinical combination trials of bisphosphonate and nutraceutical combinations are rare. Most studies either compare isoflavones to alendronate, or test nutraceuticals alone or in combination with other nutraceuticals/exercise. A recent paper suggests that combining certain herbal medicines with bisphosphonates is associated with improved BMD in a clinical cohort of primary osteoporosis patients, but the quality of evidence was low due to high risk of bias and significant heterogeneity [94]. Improved spinal fusion with an alendronate and curcumin combination has been reported, as well as prevention of glucocorticoid-induced osteoporosis and enhanced anti-osteoporotic effects of alendronate when combined with quercetin [95,96]. An in vitro study demonstrated a synergistic suppression of RANKL-induced osteoclastic differentiation by a combination of alendronate and genistein [97]. Also, a study evaluating combinations of red clover and hop extract suggests positive effects on bone metabolism in OV rats [82]. To our knowledge, our experimental design is among the first to assess the combination of a bisphosphonate and a standardized hop extract, and our findings are generally consistent with the few available studies on combination therapy, with the novel finding of a negative effect of high-dose alendronate dose with hop extract, as well as noticeable changes after a relatively short treatment period of 2 weeks. Our explorative preclinical data suggest that hop extract warrants further investigation as a possible adjunct in osteoporosis therapy, and the observed negative effect of the high-dose alendronate and hop extract combination highlights a potential interaction that warrants cautious interpretation and further investigation in translational and clinical studies. This area of research is promising, and further investigation should explore the underlying molecular mechanisms, as a deeper understanding of these interactions could facilitate the design of future combination therapies, the determination of optimal dose ranges, and the development of novel therapeutic strategies for osteoporosis.

## Figures and Tables

**Table 1:** Analysis of hematological blood determinants *.

Group (n)	RBC (×10^12^/L)	WBC (×10^9^/L)	PLT (×10^9^/L)	Hb (g/L)	Hct (L/L)	MCV (fL)	MCH (pg)	MCHC (g/L)
**C** (10)	6.96 (0.26)	3.52 (0.9)	716.5 (37.73)	152.2 (5.2)	0.41 (0.01)	58.4 (1.96)	21.89 (0.68)	375.1 (8.03)
**OV** (7)	7.82 (0.51)	5.3 (2.79)	623 (64.2)	170.14 (11.33)	0.46 (0.03)	58.29 (2.63)	21.8 (1.07)	373.29 (4.82)
**AL** (10)	7.79 (0.81)	4.31 (1.14)	594.38 (104.03)	175.88 (17.87)	0.46 (0.05)	58.75 (1.04)	22.56 (0.73)	384.63 (10.68)
**AH** (9)	7.27 (0.63)	5.22 (1.21)	543.44 (94.71)	167.89 (10.35)	0.43 (0.03)	58.89 (1.36)	23.21 (1.95)	394.89 (29.17)
**AL-X** (6)	7.99 (0.32)	3.92 (1.17)	556.2 (98.3)	176.7 (17.73)	0.48 (0.05)	59.4 (3.95)	22.37 (1.34)	376.22 (6.3)
**AH-X** (10)	7.32 (0.81)	5.22 (1.31)	507.2 (90.08)	166.8 (11.61)	0.43 (0.04)	59.5 (1.9)	22.88 (1.22)	384.6 (10.74)
**X** (5)	7.02 (0.49)	5.57 (0.9)	620.83 (29.71)	161.5 (9.2)	0.41 (0.02)	59.17 (1.17)	23.02 (0.57)	390 (8.83)
***p***	**<0.001**	**0.005**	**<0.001**	**<0.001**	**0.001**	0.88	**0.04**	**0.005**

* Abbreviations: RBC, red blood cells; WBC, white blood cells; PLT, platelets; Hb, hemoglobin; Hct, hematocrit; MCV, mean corpuscular volume; MCH, mean corpuscular hemoglobin; MCHC, mean corpuscular hemoglobin concentration. Six-month-old rats were ovariectomized (*n* = 60) or sham-operated (*n* = 10). One month later, animals were assigned to treatment groups for two weeks and then euthanized. Blood was collected for hematology and serum biochemistry. Groups: C, sham-operated control group; OV, untreated ovariectomized animals; AL, ovariectomized animals, low alendronate dose; AH, ovariectomized animals, high alendronate dose; AL-X, ovariectomized animals, low alendronate dose and hop extract; AH-X, ovariectomized animals, high alendronate dose and hop extract; X, ovariectomized animals, hop extract. The values in the table are presented as mean (standard deviation). Statistical significance was assessed using Welch’s ANOVA, followed by Tukey post hoc tests. For medians and interquartile ranges, as well as normality tests and other details, please see Supplementary Material (Table S2).

**Table 2:** Electrolytes, renal and metabolic parameters *.

Group (n)	Ca (mmol/L)	P (mmol/L)	Na (mmol/L)	K (mmol/L)	Glc (mmol/L)	BUN (mmol/L)	CRE (µmol/L)
**C** (10)	2.73 (0.09)	2.13 (0.27)	131.3 (6.02)	4.52 (0.39)	13.7 (2.49)	5.66 (0.91)	39.7 (12.4)
**OV** (7)	2.62 (0.05)	2.11 (0.24)	131.57 (4.43)	4.5 (0.45)	15.4 (2.95)	5.79 (1.05)	36.14 (8.15)
**AL** (10)	2.66 (0.14)	1.98 (0.3)	131.5 (4.9)	4.59 (0.31)	15.35 (2.31)	5.95 (0.64)	38.13 (7)
**AH** (9)	2.58 (0.1)	1.97 (0.5)	127.67 (7.31)	4.7 (0.64)	15.86 (1.94)	6 (0.93)	27 (14.59)
**AL-X** (6)	2.67 (0.09)	2.06 (0.2)	132.3 (3.86)	4.67 (0.57)	16.25 (1.59)	5.64 (0.47)	45.7 (16.61)
**AH-X** (10)	2.66 (0.05)	2.08 (0.21)	132.5 (4.74)	4.81 (0.5)	15.68 (1.87)	6.47 (0.57)	42.2 (19.05)
**X** (9)	2.52 (0.31)	2.1 (0.24)	132 (4.86)	4.7 (0.89)	15.19 (1.34)	6.49 (1)	56.67 (14.54)
***p***	0.06	0.94	0.81	0.85	0.34	0.08	0.09

* Abbreviations: Glc, glucose; BUN, blood urea nitrogen; CRE, creatinine. Six-month-old rats were ovariectomized (*n* = 60) or sham-operated (*n* = 10). One month later, animals were assigned to treatment groups for two weeks and then euthanized. Blood was collected for hematology and serum biochemistry. Groups: C, sham-operated control group; OV, untreated ovariectomized animals; AL, ovariectomized animals, low alendronate dose; AH, ovariectomized animals, high alendronate dose; AL-X, ovariectomized animals, low alendronate dose and hop extract; AH-X, ovariectomized animals, high alendronate dose and hop extract; X, ovariectomized animals, hop extract. The values in the table are presented as mean (standard deviation). Statistical significance was assessed using Welch’s ANOVA, followed by Tukey post hoc tests. For medians and interquartile ranges, as well as normality tests, please see Supplementary Material (Table S2).

**Table 3:** Parameters describing liver function, protein metabolism, and enzymes *.

Group (n)	ALP (U/L)	ALT (U/L)	TBIL (mmol/L)	AMY (U/L)	ALB (g/L)	GLO (g/L)	TP (g/L)
**C** (10)	66.1 (29.34)	24.1 (10.72)	4.5 (0.53)	508.2 (100.11)	52.6 (3.95)	12 (3.27)	64.6 (2.63)
**OV** (7)	97.57 (40.48)	21.86 (7.34)	4.71 (1.5)	454.14 (59.68)	46.29 (4.5)	12.29 (4.72)	58.57 (3.51)
**AL** (10)	68.13 (19.42)	28.25 (19.25)	5 (0.93)	477.63 (63.88)	46.75 (4.5)	14 (2.27)	60.75 (3.73)
**AH** (9)	92 (33.99)	21.33 (9.68)	4.78 (0.44)	512.33 (53.84)	50.11 (5.06)	10.44 (1.42)	60.67 (4.44)
**AL-X** (6)	90.9 (43.46)	32.7 (18.3)	4.8 (0.79)	468.5 (71.53)	49.4 (5.17)	12.3 (2.31)	61.6 (4.48)
**AH-X** (10)	102.2 (49.17)	44.4 (33.24)	4.8 (0.63)	492.3 (60.41)	47.1 (2.51)	11.4 (2.5)	58.6 (2.01)
**X** (9)	112.5 (36.83)	24.43 (7.93)	5 (0.89)	466.43 (58.69)	48 (2.37)	11.67 (1.86)	61.14 (3.53)
***p***	0.11	0.38	0.81	0.51	**0.04**	0.07	**0.002**

* Abbreviations: ALP, alkaline phosphatase; ALT, alanine aminotransferase; TBIL, total bilirubin; AMY, amylase; ALB, albumin; GLO, globulin; TP, total protein. Six-month-old rats were ovariectomized (*n* = 60) or sham-operated (*n* = 10). One month later, animals were assigned to treatment groups for two weeks and then euthanized. Blood was collected for hematology and serum biochemistry. Groups: C, sham-operated control group; OV, untreated ovariectomized animals; AL, ovariectomized animals, low alendronate dose; AH, ovariectomized animals, high alendronate dose; AL-X, ovariectomized animals, low alendronate dose and hop extract; AH-X, ovariectomized animals, high alendronate dose and hop extract; X, ovariectomized animals, hop extract. The values in the table are presented as mean (standard deviation). Statistical significance was assessed using Welch’s ANOVA, followed by Tukey post hoc tests. For medians and interquartile ranges, as well as normality tests, please see Supplementary Material (Table S2).

## Data Availability

The original contributions presented in this study are included in the article/Supplementary Material. Further inquiries can be directed to the corresponding author.

## Supplementary Materials

The following supporting information can be downloaded at: https://www.mdpi.com/article/10.3390/medsci14020239/s1, Table S1: Statistical data for bone parameters; Table S2: Statistical data for hematology and biochemistry parameters; Table S3: Statistical data for Western blot parameters (JASP output HTML files).
