# Supplementary material for: The Early Effect of Alendronate, Hop Extract and Their Combination on Bone Structural Properties in a Rat Model of Osteoporosis
Source: Med Sci (Basel). 2026 May 5;14(2):239. doi: 10.3390/medsci14020239 (PMC13214732; doi:10.3390/medsci14020239)
Supplement: Supplementary file 1 [file medsci-14-00239-s001.zip › Table S1.html]

JASP 


# Results

## Descriptive Statistics

| Descriptive Statistics | | | | | | | | | | | | | | | | | | | | | | | | | |
| --- | --- | --- | --- | --- | --- | --- | --- | --- | --- | --- | --- | --- | --- | --- | --- | --- | --- | --- | --- | --- | --- | --- | --- | --- | --- |
|  | |  | | Valid | | Missing | | Median | | Mean | | Std. Deviation | | Shapiro-Wilk | | P-value of Shapiro-Wilk | | Minimum | | Maximum | | 25th percentile | | 75th percentile | |
| Bone volume (BV) mm³ |  | C |  | 10 |  | 0 |  | 94.99410 |  | 97.47100 |  | 9.98329 |  | 0.90062 |  | .22258 |  | 86.30250 |  | 119.53970 |  | 91.68825 |  | 100.79258 |  |
| Bone volume (BV) mm³ |  | OV |  | 9 |  | 0 |  | 73.96560 |  | 73.98310 |  | 9.69799 |  | 0.98228 |  | .97496 |  | 56.21700 |  | 90.02880 |  | 69.44510 |  | 79.48220 |  |
| Bone volume (BV) mm³ |  | AL |  | 9 |  | 0 |  | 87.61940 |  | 83.99281 |  | 7.86214 |  | 0.92699 |  | .45322 |  | 72.23990 |  | 95.32890 |  | 78.62540 |  | 88.79870 |  |
| Bone volume (BV) mm³ |  | AH |  | 10 |  | 0 |  | 99.45480 |  | 100.32952 |  | 5.87744 |  | 0.84154 |  | .04603 |  | 92.77900 |  | 114.65260 |  | 98.10508 |  | 100.43413 |  |
| Bone volume (BV) mm³ |  | AL-X |  | 10 |  | 0 |  | 93.89190 |  | 94.61633 |  | 5.81079 |  | 0.96734 |  | .86517 |  | 86.32310 |  | 105.66510 |  | 90.51705 |  | 97.23453 |  |
| Bone volume (BV) mm³ |  | AH-X |  | 10 |  | 0 |  | 79.62380 |  | 81.00531 |  | 6.27806 |  | 0.88127 |  | .13496 |  | 75.08660 |  | 93.52030 |  | 75.81953 |  | 83.89420 |  |
| Bone volume (BV) mm³ |  | X |  | 9 |  | 0 |  | 102.94930 |  | 105.95830 |  | 6.76545 |  | 0.83229 |  | .04737 |  | 99.04310 |  | 114.87730 |  | 100.54980 |  | 113.19240 |  |
| Bone volume fraction (BV/TV) |  | C |  | 10 |  | 0 |  | 0.57770 |  | 0.58890 |  | 0.04132 |  | 0.89298 |  | .18318 |  | 0.53760 |  | 0.68490 |  | 0.56963 |  | 0.59998 |  |
| Bone volume fraction (BV/TV) |  | OV |  | 9 |  | 0 |  | 0.46270 |  | 0.45761 |  | 0.04892 |  | 0.96456 |  | .84460 |  | 0.38140 |  | 0.53280 |  | 0.41750 |  | 0.47380 |  |
| Bone volume fraction (BV/TV) |  | AL |  | 9 |  | 0 |  | 0.50690 |  | 0.51346 |  | 0.03559 |  | 0.96750 |  | .87252 |  | 0.46640 |  | 0.57370 |  | 0.49130 |  | 0.53980 |  |
| Bone volume fraction (BV/TV) |  | AH |  | 10 |  | 0 |  | 0.58825 |  | 0.59810 |  | 0.03521 |  | 0.73431 |  | .00230 |  | 0.56610 |  | 0.68980 |  | 0.58615 |  | 0.60343 |  |
| Bone volume fraction (BV/TV) |  | AL-X |  | 10 |  | 0 |  | 0.56850 |  | 0.57675 |  | 0.03586 |  | 0.92577 |  | .40759 |  | 0.52820 |  | 0.64150 |  | 0.55335 |  | 0.59250 |  |
| Bone volume fraction (BV/TV) |  | AH-X |  | 10 |  | 0 |  | 0.48525 |  | 0.49262 |  | 0.02710 |  | 0.93129 |  | .46066 |  | 0.45640 |  | 0.53770 |  | 0.47658 |  | 0.50475 |  |
| Bone volume fraction (BV/TV) |  | X |  | 9 |  | 0 |  | 0.61210 |  | 0.61278 |  | 0.02722 |  | 0.94789 |  | .66686 |  | 0.57380 |  | 0.65220 |  | 0.59020 |  | 0.63840 |  |
| Average cortical area (Ct.Ar) mm² |  | C |  | 10 |  | 0 |  | 5.49870 |  | 5.41452 |  | 0.31010 |  | 0.93947 |  | .54709 |  | 4.96290 |  | 5.87250 |  | 5.14008 |  | 5.60922 |  |
| Average cortical area (Ct.Ar) mm² |  | OV |  | 9 |  | 0 |  | 4.08530 |  | 4.16287 |  | 0.46627 |  | 0.92803 |  | .46278 |  | 3.53920 |  | 5.15450 |  | 3.87370 |  | 4.38890 |  |
| Average cortical area (Ct.Ar) mm² |  | AL |  | 9 |  | 0 |  | 4.68000 |  | 4.68506 |  | 0.38682 |  | 0.94145 |  | .59720 |  | 4.14960 |  | 5.20900 |  | 4.37190 |  | 5.00350 |  |
| Average cortical area (Ct.Ar) mm² |  | AH |  | 10 |  | 0 |  | 5.44635 |  | 5.45905 |  | 0.31084 |  | 0.89333 |  | .18480 |  | 5.05540 |  | 6.16380 |  | 5.33890 |  | 5.53505 |  |
| Average cortical area (Ct.Ar) mm² |  | AL-X |  | 10 |  | 0 |  | 5.20810 |  | 5.24225 |  | 0.26644 |  | 0.97150 |  | .90440 |  | 4.86690 |  | 5.75360 |  | 5.07285 |  | 5.40968 |  |
| Average cortical area (Ct.Ar) mm² |  | AH-X |  | 10 |  | 0 |  | 4.52730 |  | 4.55897 |  | 0.31335 |  | 0.96451 |  | .83576 |  | 4.11670 |  | 5.19600 |  | 4.39505 |  | 4.69005 |  |
| Average cortical area (Ct.Ar) mm² |  | X |  | 9 |  | 0 |  | 5.87270 |  | 6.08961 |  | 0.51506 |  | 0.87576 |  | .14162 |  | 5.59360 |  | 7.15650 |  | 5.72070 |  | 6.41840 |  |
| Average cortical area fraction (Ct.Ar/Tt.Ar) |  | C |  | 10 |  | 0 |  | 0.33165 |  | 0.32708 |  | 0.01476 |  | 0.91644 |  | .32824 |  | 0.30710 |  | 0.35510 |  | 0.31383 |  | 0.33400 |  |
| Average cortical area fraction (Ct.Ar/Tt.Ar) |  | OV |  | 9 |  | 0 |  | 0.25660 |  | 0.25782 |  | 0.02909 |  | 0.91449 |  | .34859 |  | 0.22250 |  | 0.30440 |  | 0.23920 |  | 0.26460 |  |
| Average cortical area fraction (Ct.Ar/Tt.Ar) |  | AL |  | 9 |  | 0 |  | 0.28250 |  | 0.28593 |  | 0.01670 |  | 0.93144 |  | .49519 |  | 0.26710 |  | 0.31810 |  | 0.27140 |  | 0.29380 |  |
| Average cortical area fraction (Ct.Ar/Tt.Ar) |  | AH |  | 10 |  | 0 |  | 0.31965 |  | 0.32401 |  | 0.01999 |  | 0.85434 |  | .06540 |  | 0.30470 |  | 0.37050 |  | 0.30928 |  | 0.32795 |  |
| Average cortical area fraction (Ct.Ar/Tt.Ar) |  | AL-X |  | 10 |  | 0 |  | 0.31690 |  | 0.31921 |  | 0.01767 |  | 0.94724 |  | .63594 |  | 0.29690 |  | 0.35210 |  | 0.30518 |  | 0.33008 |  |
| Average cortical area fraction (Ct.Ar/Tt.Ar) |  | AH-X |  | 10 |  | 0 |  | 0.27770 |  | 0.27700 |  | 0.01780 |  | 0.88809 |  | .16138 |  | 0.25500 |  | 0.29980 |  | 0.26092 |  | 0.29225 |  |
| Average cortical area fraction (Ct.Ar/Tt.Ar) |  | X |  | 9 |  | 0 |  | 0.34510 |  | 0.35240 |  | 0.02161 |  | 0.75599 |  | .00636 |  | 0.33260 |  | 0.40330 |  | 0.34410 |  | 0.34930 |  |
| Average cortical thickness (Ct.Th) mm |  | C |  | 10 |  | 0 |  | 0.41215 |  | 0.41464 |  | 0.01926 |  | 0.94504 |  | .61032 |  | 0.37950 |  | 0.43880 |  | 0.40707 |  | 0.43005 |  |
| Average cortical thickness (Ct.Th) mm |  | OV |  | 9 |  | 0 |  | 0.40360 |  | 0.40439 |  | 0.02043 |  | 0.93417 |  | .52207 |  | 0.37720 |  | 0.43190 |  | 0.38560 |  | 0.41950 |  |
| Average cortical thickness (Ct.Th) mm |  | AL |  | 9 |  | 0 |  | 0.39530 |  | 0.39359 |  | 0.01664 |  | 0.93000 |  | .48134 |  | 0.35960 |  | 0.41520 |  | 0.38670 |  | 0.39850 |  |
| Average cortical thickness (Ct.Th) mm |  | AH |  | 10 |  | 0 |  | 0.40220 |  | 0.40475 |  | 0.01748 |  | 0.96849 |  | .87651 |  | 0.37880 |  | 0.43930 |  | 0.39545 |  | 0.41495 |  |
| Average cortical thickness (Ct.Th) mm |  | AL-X |  | 10 |  | 0 |  | 0.41275 |  | 0.41594 |  | 0.01468 |  | 0.92402 |  | .39171 |  | 0.39880 |  | 0.44260 |  | 0.40403 |  | 0.42763 |  |
| Average cortical thickness (Ct.Th) mm |  | AH-X |  | 10 |  | 0 |  | 0.39315 |  | 0.39360 |  | 0.00854 |  | 0.94405 |  | .59893 |  | 0.37890 |  | 0.40510 |  | 0.39110 |  | 0.39985 |  |
| Average cortical thickness (Ct.Th) mm |  | X |  | 9 |  | 0 |  | 0.42590 |  | 0.43118 |  | 0.01979 |  | 0.86539 |  | .10970 |  | 0.40980 |  | 0.47430 |  | 0.41800 |  | 0.43650 |  |
| Endocortical perimeter (Ec.Pm) mm |  | C |  | 10 |  | 0 |  | 23.01845 |  | 23.13400 |  | 1.71776 |  | 0.94129 |  | .56744 |  | 20.22850 |  | 26.82590 |  | 22.25813 |  | 23.65183 |  |
| Endocortical perimeter (Ec.Pm) mm |  | OV |  | 9 |  | 0 |  | 19.48840 |  | 18.82752 |  | 1.47952 |  | 0.80540 |  | .02353 |  | 16.21990 |  | 20.09110 |  | 17.72110 |  | 19.80440 |  |
| Endocortical perimeter (Ec.Pm) mm |  | AL |  | 9 |  | 0 |  | 20.82060 |  | 20.74687 |  | 0.87527 |  | 0.92760 |  | .45882 |  | 19.75890 |  | 22.28900 |  | 19.91460 |  | 21.02770 |  |
| Endocortical perimeter (Ec.Pm) mm |  | AH |  | 10 |  | 0 |  | 23.56845 |  | 23.79100 |  | 0.88495 |  | 0.96940 |  | .88527 |  | 22.39770 |  | 25.49550 |  | 23.30235 |  | 24.40523 |  |
| Endocortical perimeter (Ec.Pm) mm |  | AL-X |  | 10 |  | 0 |  | 22.37580 |  | 22.32202 |  | 0.93182 |  | 0.96818 |  | .87351 |  | 20.82360 |  | 24.19640 |  | 21.86930 |  | 22.75335 |  |
| Endocortical perimeter (Ec.Pm) mm |  | AH-X |  | 10 |  | 0 |  | 20.61685 |  | 20.33415 |  | 1.10588 |  | 0.90166 |  | .22844 |  | 18.56690 |  | 21.60890 |  | 19.45790 |  | 21.24587 |  |
| Endocortical perimeter (Ec.Pm) mm |  | X |  | 9 |  | 0 |  | 25.95830 |  | 25.77717 |  | 1.51367 |  | 0.94807 |  | .66890 |  | 23.68800 |  | 28.14630 |  | 24.48430 |  | 26.81870 |  |
| Endocortical surface (3D) (Ec.S3D) mm² |  | C |  | 10 |  | 0 |  | 291.31910 |  | 295.46392 |  | 22.44363 |  | 0.96693 |  | .86099 |  | 259.58510 |  | 341.22200 |  | 283.90483 |  | 306.41275 |  |
| Endocortical surface (3D) (Ec.S3D) mm² |  | OV |  | 9 |  | 0 |  | 241.55320 |  | 232.59904 |  | 19.02657 |  | 0.78842 |  | .01505 |  | 202.69240 |  | 250.12670 |  | 214.94520 |  | 246.70510 |  |
| Endocortical surface (3D) (Ec.S3D) mm² |  | AL |  | 9 |  | 0 |  | 259.60840 |  | 259.47508 |  | 13.70932 |  | 0.91649 |  | .36400 |  | 241.96070 |  | 280.16640 |  | 248.66520 |  | 266.24870 |  |
| Endocortical surface (3D) (Ec.S3D) mm² |  | AH |  | 10 |  | 0 |  | 298.84130 |  | 303.57891 |  | 16.34735 |  | 0.85950 |  | .07528 |  | 283.56970 |  | 334.24300 |  | 293.77190 |  | 306.68083 |  |
| Endocortical surface (3D) (Ec.S3D) mm² |  | AL-X |  | 10 |  | 0 |  | 280.62060 |  | 284.39630 |  | 13.55117 |  | 0.92277 |  | .38066 |  | 265.16310 |  | 314.29930 |  | 279.65795 |  | 289.44735 |  |
| Endocortical surface (3D) (Ec.S3D) mm² |  | AH-X |  | 10 |  | 0 |  | 257.67200 |  | 253.02799 |  | 14.75244 |  | 0.87522 |  | .11493 |  | 227.24450 |  | 268.12870 |  | 242.01210 |  | 263.63645 |  |
| Endocortical surface (3D) (Ec.S3D) mm² |  | X |  | 9 |  | 0 |  | 340.00570 |  | 338.61907 |  | 16.24357 |  | 0.97250 |  | .91519 |  | 308.96780 |  | 365.16120 |  | 333.17250 |  | 345.32800 |  |
| Average marrow area (Ma.Ar) mm² |  | C |  | 10 |  | 0 |  | 11.10390 |  | 11.15302 |  | 0.76331 |  | 0.92799 |  | .42842 |  | 10.21100 |  | 12.73050 |  | 10.66630 |  | 11.51180 |  |
| Average marrow area (Ma.Ar) mm² |  | OV |  | 9 |  | 0 |  | 12.29160 |  | 12.03120 |  | 1.15723 |  | 0.91095 |  | .32259 |  | 9.51570 |  | 13.53940 |  | 11.77710 |  | 12.42690 |  |
| Average marrow area (Ma.Ar) mm² |  | AL |  | 9 |  | 0 |  | 11.37290 |  | 11.70239 |  | 0.77725 |  | 0.83777 |  | .05454 |  | 10.81290 |  | 12.86660 |  | 11.29420 |  | 12.52300 |  |
| Average marrow area (Ma.Ar) mm² |  | AH |  | 10 |  | 0 |  | 11.48340 |  | 11.40426 |  | 0.64736 |  | 0.92574 |  | .40730 |  | 10.32030 |  | 12.19010 |  | 11.09285 |  | 11.91875 |  |
| Average marrow area (Ma.Ar) mm² |  | AL-X |  | 10 |  | 0 |  | 11.35760 |  | 11.18702 |  | 0.45442 |  | 0.87391 |  | .11100 |  | 10.47910 |  | 11.68050 |  | 10.78028 |  | 11.52880 |  |
| Average marrow area (Ma.Ar) mm² |  | AH-X |  | 10 |  | 0 |  | 11.90330 |  | 11.91194 |  | 0.70876 |  | 0.93809 |  | .53194 |  | 10.86390 |  | 12.93740 |  | 11.32035 |  | 12.53258 |  |
| Average marrow area (Ma.Ar) mm² |  | X |  | 9 |  | 0 |  | 10.97280 |  | 11.18278 |  | 0.60955 |  | 0.83634 |  | .05258 |  | 10.58870 |  | 12.58900 |  | 10.85720 |  | 11.23920 |  |
| Periosteal perimeter (Ps.Pm) mm |  | C |  | 10 |  | 0 |  | 17.09905 |  | 17.10995 |  | 0.60087 |  | 0.97280 |  | .91553 |  | 16.17190 |  | 18.33130 |  | 16.70425 |  | 17.41300 |  |
| Periosteal perimeter (Ps.Pm) mm |  | OV |  | 9 |  | 0 |  | 16.77840 |  | 16.51848 |  | 0.81231 |  | 0.74782 |  | .00512 |  | 14.63820 |  | 17.13820 |  | 16.61210 |  | 17.05230 |  |
| Periosteal perimeter (Ps.Pm) mm |  | AL |  | 9 |  | 0 |  | 16.82640 |  | 16.96918 |  | 0.43804 |  | 0.88575 |  | .18033 |  | 16.47800 |  | 17.60540 |  | 16.65940 |  | 17.32490 |  |
| Periosteal perimeter (Ps.Pm) mm |  | AH |  | 10 |  | 0 |  | 17.23215 |  | 17.30260 |  | 0.45318 |  | 0.93183 |  | .46614 |  | 16.53430 |  | 17.86850 |  | 17.03630 |  | 17.75238 |  |
| Periosteal perimeter (Ps.Pm) mm |  | AL-X |  | 10 |  | 0 |  | 16.94350 |  | 16.85386 |  | 0.33990 |  | 0.96518 |  | .84296 |  | 16.24340 |  | 17.43520 |  | 16.64208 |  | 16.97398 |  |
| Periosteal perimeter (Ps.Pm) mm |  | AH-X |  | 10 |  | 0 |  | 16.76990 |  | 17.03073 |  | 0.54821 |  | 0.84762 |  | .05442 |  | 16.47000 |  | 17.87660 |  | 16.58065 |  | 17.51555 |  |
| Periosteal perimeter (Ps.Pm) mm |  | X |  | 9 |  | 0 |  | 17.55690 |  | 17.52122 |  | 0.53932 |  | 0.95739 |  | .77066 |  | 16.53070 |  | 18.46960 |  | 17.41010 |  | 17.70490 |  |
| Periosteal surface (3D) (Ps.S3D) mm² |  | C |  | 10 |  | 0 |  | 198.48945 |  | 199.12591 |  | 7.36420 |  | 0.97418 |  | .92669 |  | 188.01980 |  | 213.51810 |  | 193.82828 |  | 202.94615 |  |
| Periosteal surface (3D) (Ps.S3D) mm² |  | OV |  | 9 |  | 0 |  | 195.68290 |  | 191.92126 |  | 10.36424 |  | 0.76224 |  | .00751 |  | 168.23190 |  | 200.72440 |  | 193.35560 |  | 197.81730 |  |
| Periosteal surface (3D) (Ps.S3D) mm² |  | AL |  | 9 |  | 0 |  | 194.41310 |  | 197.03729 |  | 6.57648 |  | 0.82759 |  | .04196 |  | 190.89570 |  | 207.11250 |  | 191.51710 |  | 204.54990 |  |
| Periosteal surface (3D) (Ps.S3D) mm² |  | AH |  | 10 |  | 0 |  | 199.20340 |  | 201.34941 |  | 5.32723 |  | 0.86752 |  | .09351 |  | 192.49520 |  | 207.60540 |  | 198.45878 |  | 206.99150 |  |
| Periosteal surface (3D) (Ps.S3D) mm² |  | AL-X |  | 10 |  | 0 |  | 196.98240 |  | 196.25881 |  | 4.24276 |  | 0.94359 |  | .59356 |  | 190.39910 |  | 204.06530 |  | 192.60023 |  | 198.08618 |  |
| Periosteal surface (3D) (Ps.S3D) mm² |  | AH-X |  | 10 |  | 0 |  | 194.97450 |  | 197.84167 |  | 6.68205 |  | 0.82677 |  | .03059 |  | 190.96920 |  | 207.01880 |  | 192.05263 |  | 205.03248 |  |
| Periosteal surface (3D) (Ps.S3D) mm² |  | X |  | 9 |  | 0 |  | 205.35690 |  | 207.96802 |  | 7.45464 |  | 0.84765 |  | .07021 |  | 199.63160 |  | 224.45830 |  | 203.90180 |  | 210.74910 |  |
| Total volume (TV) mm³ |  | C |  | 10 |  | 0 |  | 163.13440 |  | 165.35914 |  | 9.40053 |  | 0.94229 |  | .57875 |  | 152.83170 |  | 184.13880 |  | 158.90623 |  | 171.22893 |  |
| Total volume (TV) mm³ |  | OV |  | 9 |  | 0 |  | 167.75780 |  | 161.65399 |  | 12.26152 |  | 0.81863 |  | .03325 |  | 135.87490 |  | 173.78210 |  | 161.33090 |  | 168.97730 |  |
| Total volume (TV) mm³ |  | AL |  | 9 |  | 0 |  | 160.04960 |  | 163.54687 |  | 10.06296 |  | 0.75961 |  | .00701 |  | 154.54390 |  | 176.78460 |  | 154.87380 |  | 176.53460 |  |
| Total volume (TV) mm³ |  | AH |  | 10 |  | 0 |  | 167.72965 |  | 167.84323 |  | 6.06519 |  | 0.96355 |  | .82547 |  | 156.33790 |  | 176.65390 |  | 164.26960 |  | 170.78725 |  |
| Total volume (TV) mm³ |  | AL-X |  | 10 |  | 0 |  | 164.00410 |  | 164.09678 |  | 3.81820 |  | 0.97220 |  | .91049 |  | 156.91330 |  | 169.90500 |  | 161.77938 |  | 166.69178 |  |
| Total volume (TV) mm³ |  | AH-X |  | 10 |  | 0 |  | 161.42210 |  | 164.39746 |  | 7.86562 |  | 0.88110 |  | .13432 |  | 153.38390 |  | 174.69330 |  | 158.51943 |  | 172.47263 |  |
| Total volume (TV) mm³ |  | X |  | 9 |  | 0 |  | 173.56170 |  | 172.84671 |  | 5.96014 |  | 0.92011 |  | .39311 |  | 164.78390 |  | 180.95030 |  | 168.07400 |  | 177.55510 |  |
| Average trabecular separation (Tb.Sp) mm |  | C |  | 10 |  | 0 |  | 0.36760 |  | 0.34453 |  | 0.09204 |  | 0.93098 |  | .45763 |  | 0.18840 |  | 0.46460 |  | 0.29925 |  | 0.40595 |  |
| Average trabecular separation (Tb.Sp) mm |  | OV |  | 9 |  | 0 |  | 0.31870 |  | 0.33862 |  | 0.08913 |  | 0.74766 |  | .00509 |  | 0.25120 |  | 0.56000 |  | 0.29410 |  | 0.34290 |  |
| Average trabecular separation (Tb.Sp) mm |  | AL |  | 9 |  | 0 |  | 0.32310 |  | 0.33093 |  | 0.10188 |  | 0.71168 |  | .00194 |  | 0.25140 |  | 0.58550 |  | 0.27270 |  | 0.33030 |  |
| Average trabecular separation (Tb.Sp) mm |  | AH |  | 10 |  | 0 |  | 0.28130 |  | 0.28302 |  | 0.04035 |  | 0.98339 |  | .98073 |  | 0.21530 |  | 0.35150 |  | 0.26248 |  | 0.30105 |  |
| Average trabecular separation (Tb.Sp) mm |  | AL-X |  | 10 |  | 0 |  | 0.35220 |  | 0.32710 |  | 0.07081 |  | 0.91215 |  | .29607 |  | 0.20040 |  | 0.40860 |  | 0.29208 |  | 0.37363 |  |
| Average trabecular separation (Tb.Sp) mm |  | AH-X |  | 10 |  | 0 |  | 0.32545 |  | 0.32978 |  | 0.05403 |  | 0.88244 |  | .13917 |  | 0.26280 |  | 0.39780 |  | 0.27970 |  | 0.37965 |  |
| Average trabecular separation (Tb.Sp) mm |  | X |  | 9 |  | 0 |  | 0.33550 |  | 0.29781 |  | 0.05988 |  | 0.80799 |  | .02518 |  | 0.20900 |  | 0.35580 |  | 0.23090 |  | 0.34450 |  |
| Average trabecular thickness (Tb.Th) mm |  | C |  | 10 |  | 0 |  | 0.12700 |  | 0.12827 |  | 0.00438 |  | 0.90654 |  | .25809 |  | 0.12340 |  | 0.13780 |  | 0.12560 |  | 0.12995 |  |
| Average trabecular thickness (Tb.Th) mm |  | OV |  | 9 |  | 0 |  | 0.11120 |  | 0.11014 |  | 0.00398 |  | 0.95817 |  | .77899 |  | 0.10290 |  | 0.11540 |  | 0.10790 |  | 0.11210 |  |
| Average trabecular thickness (Tb.Th) mm |  | AL |  | 9 |  | 0 |  | 0.11820 |  | 0.11890 |  | 0.00408 |  | 0.95560 |  | .75135 |  | 0.11380 |  | 0.12660 |  | 0.11600 |  | 0.12060 |  |
| Average trabecular thickness (Tb.Th) mm |  | AH |  | 10 |  | 0 |  | 0.13235 |  | 0.13184 |  | 0.00415 |  | 0.96931 |  | .88442 |  | 0.12510 |  | 0.13880 |  | 0.12848 |  | 0.13440 |  |
| Average trabecular thickness (Tb.Th) mm |  | AL-X |  | 10 |  | 0 |  | 0.12890 |  | 0.12856 |  | 0.00319 |  | 0.92206 |  | .37448 |  | 0.12470 |  | 0.13510 |  | 0.12600 |  | 0.13013 |  |
| Average trabecular thickness (Tb.Th) mm |  | AH-X |  | 10 |  | 0 |  | 0.11570 |  | 0.11519 |  | 0.00328 |  | 0.93173 |  | .46514 |  | 0.10860 |  | 0.12140 |  | 0.11413 |  | 0.11653 |  |
| Average trabecular thickness (Tb.Th) mm |  | X |  | 9 |  | 0 |  | 0.13260 |  | 0.13251 |  | 0.00274 |  | 0.87864 |  | .15191 |  | 0.12640 |  | 0.13650 |  | 0.13180 |  | 0.13400 |  |
| Average total (cortical + marrow) area (Tt.Ar) mm² |  | C |  | 10 |  | 0 |  | 16.34630 |  | 16.56753 |  | 0.94427 |  | 0.94156 |  | .57051 |  | 15.29850 |  | 18.45080 |  | 15.90730 |  | 17.15305 |  |
| Average total (cortical + marrow) area (Tt.Ar) mm² |  | OV |  | 9 |  | 0 |  | 16.81580 |  | 16.19408 |  | 1.22639 |  | 0.81936 |  | .03389 |  | 13.60110 |  | 17.41300 |  | 16.14920 |  | 16.91110 |  |
| Average total (cortical + marrow) area (Tt.Ar) mm² |  | AL |  | 9 |  | 0 |  | 16.00500 |  | 16.38747 |  | 1.00775 |  | 0.76400 |  | .00787 |  | 15.49290 |  | 17.73200 |  | 15.53400 |  | 17.65350 |  |
| Average total (cortical + marrow) area (Tt.Ar) mm² |  | AH |  | 10 |  | 0 |  | 16.79820 |  | 16.86334 |  | 0.64229 |  | 0.95796 |  | .76244 |  | 15.68080 |  | 17.72290 |  | 16.45575 |  | 17.29833 |  |
| Average total (cortical + marrow) area (Tt.Ar) mm² |  | AL-X |  | 10 |  | 0 |  | 16.43325 |  | 16.42928 |  | 0.37637 |  | 0.97402 |  | .92545 |  | 15.70700 |  | 17.00750 |  | 16.20828 |  | 16.67328 |  |
| Average total (cortical + marrow) area (Tt.Ar) mm² |  | AH-X |  | 10 |  | 0 |  | 16.15835 |  | 16.47091 |  | 0.78482 |  | 0.87582 |  | .11678 |  | 15.36910 |  | 17.48680 |  | 15.91558 |  | 17.27753 |  |
| Average total (cortical + marrow) area (Tt.Ar) mm² |  | X |  | 9 |  | 0 |  | 17.06800 |  | 17.27237 |  | 0.83109 |  | 0.92652 |  | .44890 |  | 16.25450 |  | 19.00740 |  | 16.77480 |  | 17.74520 |  |
|  | | | | | | | | | | | | | | | | | | | | | | | | | |

### Boxplots

#### Bone volume (BV) mm³

#### Bone volume fraction (BV/TV)

#### Average cortical area (Ct.Ar) mm²

#### Average cortical area fraction (Ct.Ar/Tt.Ar)

#### Average cortical thickness (Ct.Th) mm

#### Endocortical perimeter (Ec.Pm) mm

#### Endocortical surface (3D) (Ec.S3D) mm²

#### Average marrow area (Ma.Ar) mm²

#### Periosteal perimeter (Ps.Pm) mm

#### Periosteal surface (3D) (Ps.S3D) mm²

#### Total volume (TV) mm³

#### Average trabecular separation (Tb.Sp) mm

#### Average trabecular thickness (Tb.Th) mm

#### Average total (cortical + marrow) area (Tt.Ar) mm²

## Bone volume mm3

| ANOVA - Bone volume (BV) mm³ | | | | | | | | | | | | | | | | | | | | | | | | | |
| --- | --- | --- | --- | --- | --- | --- | --- | --- | --- | --- | --- | --- | --- | --- | --- | --- | --- | --- | --- | --- | --- | --- | --- | --- | --- |
|  | | | | | | | | | | | | | | | | 95% CI for η² | | | |  | | 95% CI for ω² | | | |
| Homogeneity Correction | | Cases | | Sum of Squares | | df | | Mean Square | | F | | p | | η² | | Lower | | Upper | | ω² | | Lower | | Upper | |
| None |  | Animal group |  | 7478.31078 |  | 6.00000 |  | 1246.38513 |  | 21.49192 |  | < .00001 |  | 0.68246 |  | 0.53055 |  | 0.76471 |  | 0.64728 |  | 0.48091 |  | 0.73734 |  |
|  |  | Residuals |  | 3479.59187 |  | 60.00000 |  | 57.99320 |  |  |  |  |  |  |  |  |  |  |  |  |  |  |  |  |  |
| Welch |  | Animal group |  | 7478.31078 |  | 6.00000 |  | 1246.38513 |  | 19.82911 |  | < .00001 |  | 0.68246 |  | 0.53055 |  | 0.76471 |  | 0.64728 |  | 0.48091 |  | 0.73734 |  |
|  |  | Residuals |  | 3479.59187 |  | 26.34449 |  | 132.08044 |  |  |  |  |  |  |  |  |  |  |  |  |  |  |  |  |  |
|  | | | | | | | | | | | | | | | | | | | | | | | | | |
|  |  |  |  |  |  |  |  |  |  |  |  |  |  |  |  |  |  |  |  |  |  |  |  |  |  |
| --- | --- | --- | --- | --- | --- | --- | --- | --- | --- | --- | --- | --- | --- | --- | --- | --- | --- | --- | --- | --- | --- | --- | --- | --- | --- |
| *Note.*  Type III Sum of Squares | | | | | | | | | | | | | | | | | | | | | | | | | |

### Descriptives

| Descriptives - Bone volume (BV) mm³ | | | | | | | | | | | |
| --- | --- | --- | --- | --- | --- | --- | --- | --- | --- | --- | --- |
| Animal group | | N | | Mean | | SD | | SE | | Coefficient of variation | |
| C |  | 10 |  | 97.47100 |  | 9.98329 |  | 3.15699 |  | 0.10242 |  |
| OV |  | 9 |  | 73.98310 |  | 9.69799 |  | 3.23266 |  | 0.13108 |  |
| AL |  | 9 |  | 83.99281 |  | 7.86214 |  | 2.62071 |  | 0.09360 |  |
| AH |  | 10 |  | 100.32952 |  | 5.87744 |  | 1.85861 |  | 0.05858 |  |
| AL-X |  | 10 |  | 94.61633 |  | 5.81079 |  | 1.83753 |  | 0.06141 |  |
| AH-X |  | 10 |  | 81.00531 |  | 6.27806 |  | 1.98530 |  | 0.07750 |  |
| X |  | 9 |  | 105.95830 |  | 6.76545 |  | 2.25515 |  | 0.06385 |  |
|  | | | | | | | | | | | |

#### Bar plots

#####

### Assumption Checks

| Test for Equality of Variances (Levene's) | | | | | | | |
| --- | --- | --- | --- | --- | --- | --- | --- |
| F | | df1 | | df2 | | p | |
| 1.05370 |  | 6.00000 |  | 60.00000 |  | .40037 |  |
|  | | | | | | | |

### Post Hoc Tests

#### Standard (HSD)

| Post Hoc Comparisons - Animal group | | | | | | | | | | | | | | | | | | | |
| --- | --- | --- | --- | --- | --- | --- | --- | --- | --- | --- | --- | --- | --- | --- | --- | --- | --- | --- | --- |
|  | | | | | | 95% CI for Mean Difference | | | |  | | | | | | | | | |
|  | |  | | Mean Difference | | Lower | | Upper | | SE | | df | | t | | ptukey | | pbonf | |
| C |  | OV |  | 23.48790 |  | 12.81399 |  | 34.16181 |  | 3.49900 |  | 60 |  | 6.71274 |  | < .00001 | \*\*\* | < .00001 | \*\*\* |
|  |  | AL |  | 13.47819 |  | 2.80428 |  | 24.15210 |  | 3.49900 |  | 60 |  | 3.85201 |  | .00507 | \*\* | .00604 | \*\* |
|  |  | AH |  | -2.85852 |  | -13.24774 |  | 7.53070 |  | 3.40568 |  | 60 |  | -0.83934 |  | .97968 |  | 1.00000 |  |
|  |  | (AL-X) |  | 2.85467 |  | -7.53455 |  | 13.24389 |  | 3.40568 |  | 60 |  | 0.83821 |  | .97982 |  | 1.00000 |  |
|  |  | (AH-X) |  | 16.46569 |  | 6.07647 |  | 26.85491 |  | 3.40568 |  | 60 |  | 4.83478 |  | .00019 | \*\*\* | .00020 | \*\*\* |
|  |  | X |  | -8.48730 |  | -19.16121 |  | 2.18661 |  | 3.49900 |  | 60 |  | -2.42564 |  | .20619 |  | .38444 |  |
| OV |  | AL |  | -10.00971 |  | -20.96091 |  | 0.94149 |  | 3.58990 |  | 60 |  | -2.78830 |  | .09486 |  | .14885 |  |
|  |  | AH |  | -26.34642 |  | -37.02033 |  | -15.67251 |  | 3.49900 |  | 60 |  | -7.52970 |  | < .00001 | \*\*\* | < .00001 | \*\*\* |
|  |  | (AL-X) |  | -20.63323 |  | -31.30714 |  | -9.95932 |  | 3.49900 |  | 60 |  | -5.89689 |  | < .00001 | \*\*\* | < .00001 | \*\*\* |
|  |  | (AH-X) |  | -7.02221 |  | -17.69612 |  | 3.65170 |  | 3.49900 |  | 60 |  | -2.00692 |  | .42123 |  | 1.00000 |  |
|  |  | X |  | -31.97520 |  | -42.92640 |  | -21.02400 |  | 3.58990 |  | 60 |  | -8.90699 |  | < .00001 | \*\*\* | < .00001 | \*\*\* |
| AL |  | AH |  | -16.33671 |  | -27.01062 |  | -5.66280 |  | 3.49900 |  | 60 |  | -4.66896 |  | .00034 | \*\*\* | .00037 | \*\*\* |
|  |  | (AL-X) |  | -10.62352 |  | -21.29743 |  | 0.05039 |  | 3.49900 |  | 60 |  | -3.03616 |  | .05187 |  | .07438 |  |
|  |  | (AH-X) |  | 2.98750 |  | -7.68641 |  | 13.66141 |  | 3.49900 |  | 60 |  | 0.85382 |  | .97785 |  | 1.00000 |  |
|  |  | X |  | -21.96549 |  | -32.91669 |  | -11.01429 |  | 3.58990 |  | 60 |  | -6.11869 |  | < .00001 | \*\*\* | < .00001 | \*\*\* |
| AH |  | (AL-X) |  | 5.71319 |  | -4.67603 |  | 16.10241 |  | 3.40568 |  | 60 |  | 1.67755 |  | .63328 |  | 1.00000 |  |
|  |  | (AH-X) |  | 19.32421 |  | 8.93499 |  | 29.71343 |  | 3.40568 |  | 60 |  | 5.67412 |  | < .00001 | \*\*\* | < .00001 | \*\*\* |
|  |  | X |  | -5.62878 |  | -16.30269 |  | 5.04513 |  | 3.49900 |  | 60 |  | -1.60868 |  | .67733 |  | 1.00000 |  |
| (AL-X) |  | (AH-X) |  | 13.61102 |  | 3.22180 |  | 24.00024 |  | 3.40568 |  | 60 |  | 3.99657 |  | .00321 | \*\* | .00375 | \*\* |
|  |  | X |  | -11.34197 |  | -22.01588 |  | -0.66806 |  | 3.49900 |  | 60 |  | -3.24149 |  | .03023 | \* | .04079 | \* |
| (AH-X) |  | X |  | -24.95299 |  | -35.62690 |  | -14.27908 |  | 3.49900 |  | 60 |  | -7.13146 |  | < .00001 | \*\*\* | < .00001 | \*\*\* |
|  | | | | | | | | | | | | | | | | | | | |
|  |  |  |  |  |  |  |  |  |  |  |  |  |  |  |  |  |  |  |  |
| --- | --- | --- | --- | --- | --- | --- | --- | --- | --- | --- | --- | --- | --- | --- | --- | --- | --- | --- | --- |
| \* p < .05, \*\* p < .01, \*\*\* p < .001 | | | | | | | | | | | | | | | | | | | |
| *Note.*  P-value and confidence intervals adjusted for comparing a family of 7 estimates (confidence intervals corrected using the tukey method). | | | | | | | | | | | | | | | | | | | |

| Letter-Based Grouping - Animal group | | | |
| --- | --- | --- | --- |
| Animal group | | Letter | |
| C |  | cd |  |
| OV |  | a |  |
| AL |  | ab |  |
| AH |  | cd |  |
| AL-X |  | bc |  |
| AH-X |  | a |  |
| X |  | d |  |
|  | | | |
|  |  |  |  |
| --- | --- | --- | --- |
| *Note.*  If two or more means share the same grouping symbol, then we cannot show them to be different, but we also did not show them to be the same. | | | |

### Marginal Means

| Marginal Means - Animal group | | | | | | | | | |
| --- | --- | --- | --- | --- | --- | --- | --- | --- | --- |
|  | | | | 95% CI for Mean Difference | | | |  | |
| Animal group | | Marginal Mean | | Lower | | Upper | | SE | |
| C |  | 97.47100 |  | 90.76309 |  | 104.17891 |  | 2.40818 |  |
| OV |  | 73.98310 |  | 66.91235 |  | 81.05385 |  | 2.53844 |  |
| AL |  | 83.99281 |  | 76.92206 |  | 91.06356 |  | 2.53844 |  |
| AH |  | 100.32952 |  | 93.62161 |  | 107.03743 |  | 2.40818 |  |
| AL-X |  | 94.61633 |  | 87.90842 |  | 101.32424 |  | 2.40818 |  |
| AH-X |  | 81.00531 |  | 74.29740 |  | 87.71322 |  | 2.40818 |  |
| X |  | 105.95830 |  | 98.88755 |  | 113.02905 |  | 2.53844 |  |
|  | | | | | | | | | |
|  |  |  |  |  |  |  |  |  |  |
| --- | --- | --- | --- | --- | --- | --- | --- | --- | --- |
| *Note.*  Bonferroni CI adjustment | | | | | | | | | |

## Bone volume fraction BV/TV

| ANOVA - Bone volume fraction (BV/TV) | | | | | | | | | | | | | | | | | | | | | | | | | |
| --- | --- | --- | --- | --- | --- | --- | --- | --- | --- | --- | --- | --- | --- | --- | --- | --- | --- | --- | --- | --- | --- | --- | --- | --- | --- |
|  | | | | | | | | | | | | | | | | 95% CI for η² | | | |  | | 95% CI for ω² | | | |
| Homogeneity Correction | | Cases | | Sum of Squares | | df | | Mean Square | | F | | p | | η² | | Lower | | Upper | | ω² | | Lower | | Upper | |
| None |  | Animal group |  | 0.20264 |  | 6.00000 |  | 0.03377 |  | 25.35686 |  | < .00001 |  | 0.71717 |  | 0.58034 |  | 0.79137 |  | 0.68565 |  | 0.53510 |  | 0.76718 |  |
|  |  | Residuals |  | 0.07992 |  | 60.00000 |  | 0.00133 |  |  |  |  |  |  |  |  |  |  |  |  |  |  |  |  |  |
| Welch |  | Animal group |  | 0.20264 |  | 6.00000 |  | 0.03377 |  | 24.67108 |  | < .00001 |  | 0.71717 |  | 0.58034 |  | 0.79137 |  | 0.68565 |  | 0.53510 |  | 0.76718 |  |
|  |  | Residuals |  | 0.07992 |  | 26.43182 |  | 0.00302 |  |  |  |  |  |  |  |  |  |  |  |  |  |  |  |  |  |
|  | | | | | | | | | | | | | | | | | | | | | | | | | |
|  |  |  |  |  |  |  |  |  |  |  |  |  |  |  |  |  |  |  |  |  |  |  |  |  |  |
| --- | --- | --- | --- | --- | --- | --- | --- | --- | --- | --- | --- | --- | --- | --- | --- | --- | --- | --- | --- | --- | --- | --- | --- | --- | --- |
| *Note.*  Type III Sum of Squares | | | | | | | | | | | | | | | | | | | | | | | | | |

### Descriptives

| Descriptives - Bone volume fraction (BV/TV) | | | | | | | | | | | |
| --- | --- | --- | --- | --- | --- | --- | --- | --- | --- | --- | --- |
| Animal group | | N | | Mean | | SD | | SE | | Coefficient of variation | |
| C |  | 10 |  | 0.58890 |  | 0.04132 |  | 0.01307 |  | 0.07016 |  |
| OV |  | 9 |  | 0.45761 |  | 0.04892 |  | 0.01631 |  | 0.10690 |  |
| AL |  | 9 |  | 0.51346 |  | 0.03559 |  | 0.01186 |  | 0.06932 |  |
| AH |  | 10 |  | 0.59810 |  | 0.03521 |  | 0.01114 |  | 0.05888 |  |
| AL-X |  | 10 |  | 0.57675 |  | 0.03586 |  | 0.01134 |  | 0.06218 |  |
| AH-X |  | 10 |  | 0.49262 |  | 0.02710 |  | 0.00857 |  | 0.05502 |  |
| X |  | 9 |  | 0.61278 |  | 0.02722 |  | 0.00907 |  | 0.04442 |  |
|  | | | | | | | | | | | |

#### Bar plots

#####

### Assumption Checks

| Test for Equality of Variances (Levene's) | | | | | | | |
| --- | --- | --- | --- | --- | --- | --- | --- |
| F | | df1 | | df2 | | p | |
| 0.56537 |  | 6.00000 |  | 60.00000 |  | .75616 |  |
|  | | | | | | | |

### Post Hoc Tests

#### Standard (HSD)

| Post Hoc Comparisons - Animal group | | | | | | | | | | | | | | | | | | | |
| --- | --- | --- | --- | --- | --- | --- | --- | --- | --- | --- | --- | --- | --- | --- | --- | --- | --- | --- | --- |
|  | | | | | | 95% CI for Mean Difference | | | |  | | | | | | | | | |
|  | |  | | Mean Difference | | Lower | | Upper | | SE | | df | | t | | ptukey | | pbonf | |
| C |  | OV |  | 0.13129 |  | 0.08014 |  | 0.18244 |  | 0.01677 |  | 60 |  | 7.82941 |  | < .00001 | \*\*\* | < .00001 | \*\*\* |
|  |  | AL |  | 0.07544 |  | 0.02429 |  | 0.12660 |  | 0.01677 |  | 60 |  | 4.49913 |  | .00061 | \*\*\* | .00067 | \*\*\* |
|  |  | AH |  | -0.00920 |  | -0.05899 |  | 0.04059 |  | 0.01632 |  | 60 |  | -0.56368 |  | .99758 |  | 1.00000 |  |
|  |  | (AL-X) |  | 0.01215 |  | -0.03764 |  | 0.06194 |  | 0.01632 |  | 60 |  | 0.74442 |  | .98907 |  | 1.00000 |  |
|  |  | (AH-X) |  | 0.09628 |  | 0.04649 |  | 0.14607 |  | 0.01632 |  | 60 |  | 5.89899 |  | < .00001 | \*\*\* | < .00001 | \*\*\* |
|  |  | X |  | -0.02388 |  | -0.07503 |  | 0.02728 |  | 0.01677 |  | 60 |  | -1.42395 |  | .78680 |  | 1.00000 |  |
| OV |  | AL |  | -0.05584 |  | -0.10833 |  | -0.00336 |  | 0.01720 |  | 60 |  | -3.24596 |  | .02987 | \* | .04025 | \* |
|  |  | AH |  | -0.14049 |  | -0.19164 |  | -0.08934 |  | 0.01677 |  | 60 |  | -8.37805 |  | < .00001 | \*\*\* | < .00001 | \*\*\* |
|  |  | (AL-X) |  | -0.11914 |  | -0.17029 |  | -0.06799 |  | 0.01677 |  | 60 |  | -7.10485 |  | < .00001 | \*\*\* | < .00001 | \*\*\* |
|  |  | (AH-X) |  | -0.03501 |  | -0.08616 |  | 0.01614 |  | 0.01677 |  | 60 |  | -2.08775 |  | .37307 |  | .86255 |  |
|  |  | X |  | -0.15517 |  | -0.20765 |  | -0.10268 |  | 0.01720 |  | 60 |  | -9.01906 |  | < .00001 | \*\*\* | < .00001 | \*\*\* |
| AL |  | AH |  | -0.08464 |  | -0.13580 |  | -0.03349 |  | 0.01677 |  | 60 |  | -5.04777 |  | .00009 | \*\*\* | .00009 | \*\*\* |
|  |  | (AL-X) |  | -0.06329 |  | -0.11445 |  | -0.01214 |  | 0.01677 |  | 60 |  | -3.77456 |  | .00645 | \*\* | .00776 | \*\* |
|  |  | (AH-X) |  | 0.02084 |  | -0.03032 |  | 0.07199 |  | 0.01677 |  | 60 |  | 1.24253 |  | .87446 |  | 1.00000 |  |
|  |  | X |  | -0.09932 |  | -0.15180 |  | -0.04684 |  | 0.01720 |  | 60 |  | -5.77310 |  | < .00001 | \*\*\* | < .00001 | \*\*\* |
| AH |  | (AL-X) |  | 0.02135 |  | -0.02844 |  | 0.07114 |  | 0.01632 |  | 60 |  | 1.30810 |  | .84552 |  | 1.00000 |  |
|  |  | (AH-X) |  | 0.10548 |  | 0.05569 |  | 0.15527 |  | 0.01632 |  | 60 |  | 6.46267 |  | < .00001 | \*\*\* | < .00001 | \*\*\* |
|  |  | X |  | -0.01468 |  | -0.06583 |  | 0.03648 |  | 0.01677 |  | 60 |  | -0.87531 |  | .97491 |  | 1.00000 |  |
| (AL-X) |  | (AH-X) |  | 0.08413 |  | 0.03434 |  | 0.13392 |  | 0.01632 |  | 60 |  | 5.15457 |  | .00006 | \*\*\* | .00006 | \*\*\* |
|  |  | X |  | -0.03603 |  | -0.08718 |  | 0.01513 |  | 0.01677 |  | 60 |  | -2.14852 |  | .33871 |  | .75009 |  |
| (AH-X) |  | X |  | -0.12016 |  | -0.17131 |  | -0.06900 |  | 0.01677 |  | 60 |  | -7.16561 |  | < .00001 | \*\*\* | < .00001 | \*\*\* |
|  | | | | | | | | | | | | | | | | | | | |
|  |  |  |  |  |  |  |  |  |  |  |  |  |  |  |  |  |  |  |  |
| --- | --- | --- | --- | --- | --- | --- | --- | --- | --- | --- | --- | --- | --- | --- | --- | --- | --- | --- | --- |
| \* p < .05, \*\* p < .01, \*\*\* p < .001 | | | | | | | | | | | | | | | | | | | |
| *Note.*  P-value and confidence intervals adjusted for comparing a family of 7 estimates (confidence intervals corrected using the tukey method). | | | | | | | | | | | | | | | | | | | |

| Letter-Based Grouping - Animal group | | | |
| --- | --- | --- | --- |
| Animal group | | Letter | |
| C |  | c |  |
| OV |  | a |  |
| AL |  | b |  |
| AH |  | c |  |
| AL-X |  | c |  |
| AH-X |  | ab |  |
| X |  | c |  |
|  | | | |
|  |  |  |  |
| --- | --- | --- | --- |
| *Note.*  If two or more means share the same grouping symbol, then we cannot show them to be different, but we also did not show them to be the same. | | | |

### Marginal Means

| Marginal Means - Animal group | | | | | | | | | |
| --- | --- | --- | --- | --- | --- | --- | --- | --- | --- |
|  | | | | 95% CI for Mean Difference | | | |  | |
| Animal group | | Marginal Mean | | Lower | | Upper | | SE | |
| C |  | 0.58890 |  | 0.55675 |  | 0.62105 |  | 0.01154 |  |
| OV |  | 0.45761 |  | 0.42373 |  | 0.49150 |  | 0.01217 |  |
| AL |  | 0.51346 |  | 0.47957 |  | 0.54734 |  | 0.01217 |  |
| AH |  | 0.59810 |  | 0.56595 |  | 0.63025 |  | 0.01154 |  |
| AL-X |  | 0.57675 |  | 0.54460 |  | 0.60890 |  | 0.01154 |  |
| AH-X |  | 0.49262 |  | 0.46047 |  | 0.52477 |  | 0.01154 |  |
| X |  | 0.61278 |  | 0.57889 |  | 0.64666 |  | 0.01217 |  |
|  | | | | | | | | | |
|  |  |  |  |  |  |  |  |  |  |
| --- | --- | --- | --- | --- | --- | --- | --- | --- | --- |
| *Note.*  Bonferroni CI adjustment | | | | | | | | | |

## Average Cortical area mm2

| ANOVA - Average cortical area (Ct.Ar) mm² | | | | | | | | | | | | | | | | | | | | | | | | | |
| --- | --- | --- | --- | --- | --- | --- | --- | --- | --- | --- | --- | --- | --- | --- | --- | --- | --- | --- | --- | --- | --- | --- | --- | --- | --- |
|  | | | | | | | | | | | | | | | | 95% CI for η² | | | |  | | 95% CI for ω² | | | |
| Homogeneity Correction | | Cases | | Sum of Squares | | df | | Mean Square | | F | | p | | η² | | Lower | | Upper | | ω² | | Lower | | Upper | |
| None |  | Animal group |  | 23.67149 |  | 6.00000 |  | 3.94525 |  | 28.46402 |  | < .00001 |  | 0.74002 |  | 0.61350 |  | 0.80875 |  | 0.71094 |  | 0.57135 |  | 0.78661 |  |
|  |  | Residuals |  | 8.31628 |  | 60.00000 |  | 0.13860 |  |  |  |  |  |  |  |  |  |  |  |  |  |  |  |  |  |
| Welch |  | Animal group |  | 23.67149 |  | 6.00000 |  | 3.94525 |  | 20.13942 |  | < .00001 |  | 0.74002 |  | 0.61350 |  | 0.80875 |  | 0.71094 |  | 0.57135 |  | 0.78661 |  |
|  |  | Residuals |  | 8.31628 |  | 26.23295 |  | 0.31702 |  |  |  |  |  |  |  |  |  |  |  |  |  |  |  |  |  |
|  | | | | | | | | | | | | | | | | | | | | | | | | | |
|  |  |  |  |  |  |  |  |  |  |  |  |  |  |  |  |  |  |  |  |  |  |  |  |  |  |
| --- | --- | --- | --- | --- | --- | --- | --- | --- | --- | --- | --- | --- | --- | --- | --- | --- | --- | --- | --- | --- | --- | --- | --- | --- | --- |
| *Note.*  Type III Sum of Squares | | | | | | | | | | | | | | | | | | | | | | | | | |

### Descriptives

| Descriptives - Average cortical area (Ct.Ar) mm² | | | | | | | | | | | |
| --- | --- | --- | --- | --- | --- | --- | --- | --- | --- | --- | --- |
| Animal group | | N | | Mean | | SD | | SE | | Coefficient of variation | |
| C |  | 10 |  | 5.41452 |  | 0.31010 |  | 0.09806 |  | 0.05727 |  |
| OV |  | 9 |  | 4.16287 |  | 0.46627 |  | 0.15542 |  | 0.11201 |  |
| AL |  | 9 |  | 4.68506 |  | 0.38682 |  | 0.12894 |  | 0.08257 |  |
| AH |  | 10 |  | 5.45905 |  | 0.31084 |  | 0.09830 |  | 0.05694 |  |
| AL-X |  | 10 |  | 5.24225 |  | 0.26644 |  | 0.08426 |  | 0.05083 |  |
| AH-X |  | 10 |  | 4.55897 |  | 0.31335 |  | 0.09909 |  | 0.06873 |  |
| X |  | 9 |  | 6.08961 |  | 0.51506 |  | 0.17169 |  | 0.08458 |  |
|  | | | | | | | | | | | |

#### Bar plots

#####

### Assumption Checks

| Test for Equality of Variances (Levene's) | | | | | | | |
| --- | --- | --- | --- | --- | --- | --- | --- |
| F | | df1 | | df2 | | p | |
| 1.12247 |  | 6.00000 |  | 60.00000 |  | .36028 |  |
|  | | | | | | | |

### Post Hoc Tests

#### Standard (HSD)

| Post Hoc Comparisons - Animal group | | | | | | | | | | | | | | | | | | | |
| --- | --- | --- | --- | --- | --- | --- | --- | --- | --- | --- | --- | --- | --- | --- | --- | --- | --- | --- | --- |
|  | | | | | | 95% CI for Mean Difference | | | |  | | | | | | | | | |
|  | |  | | Mean Difference | | Lower | | Upper | | SE | | df | | t | | ptukey | | pbonf | |
| C |  | OV |  | 1.25165 |  | 0.72983 |  | 1.77348 |  | 0.17106 |  | 60 |  | 7.31711 |  | < .00001 | \*\*\* | < .00001 | \*\*\* |
|  |  | AL |  | 0.72946 |  | 0.20764 |  | 1.25129 |  | 0.17106 |  | 60 |  | 4.26442 |  | .00134 | \*\* | .00151 | \*\* |
|  |  | AH |  | -0.04453 |  | -0.55244 |  | 0.46338 |  | 0.16650 |  | 60 |  | -0.26745 |  | .99997 |  | 1.00000 |  |
|  |  | (AL-X) |  | 0.17227 |  | -0.33564 |  | 0.68018 |  | 0.16650 |  | 60 |  | 1.03468 |  | .94378 |  | 1.00000 |  |
|  |  | (AH-X) |  | 0.85555 |  | 0.34764 |  | 1.36346 |  | 0.16650 |  | 60 |  | 5.13856 |  | .00006 | \*\*\* | .00007 | \*\*\* |
|  |  | X |  | -0.67509 |  | -1.19692 |  | -0.15327 |  | 0.17106 |  | 60 |  | -3.94655 |  | .00377 | \*\* | .00442 | \*\* |
| OV |  | AL |  | -0.52219 |  | -1.05757 |  | 0.01319 |  | 0.17550 |  | 60 |  | -2.97540 |  | .06045 |  | .08846 |  |
|  |  | AH |  | -1.29618 |  | -1.81801 |  | -0.77436 |  | 0.17106 |  | 60 |  | -7.57743 |  | < .00001 | \*\*\* | < .00001 | \*\*\* |
|  |  | (AL-X) |  | -1.07938 |  | -1.60121 |  | -0.55756 |  | 0.17106 |  | 60 |  | -6.31003 |  | < .00001 | \*\*\* | < .00001 | \*\*\* |
|  |  | (AH-X) |  | -0.39610 |  | -0.91793 |  | 0.12572 |  | 0.17106 |  | 60 |  | -2.31560 |  | .25380 |  | .50433 |  |
|  |  | X |  | -1.92674 |  | -2.46212 |  | -1.39136 |  | 0.17550 |  | 60 |  | -10.97846 |  | < .00001 | \*\*\* | < .00001 | \*\*\* |
| AL |  | AH |  | -0.77399 |  | -1.29582 |  | -0.25217 |  | 0.17106 |  | 60 |  | -4.52474 |  | .00056 | \*\*\* | .00061 | \*\*\* |
|  |  | (AL-X) |  | -0.55719 |  | -1.07902 |  | -0.03537 |  | 0.17106 |  | 60 |  | -3.25733 |  | .02896 | \* | .03891 | \* |
|  |  | (AH-X) |  | 0.12609 |  | -0.39574 |  | 0.64791 |  | 0.17106 |  | 60 |  | 0.73709 |  | .98963 |  | 1.00000 |  |
|  |  | X |  | -1.40456 |  | -1.93994 |  | -0.86918 |  | 0.17550 |  | 60 |  | -8.00306 |  | < .00001 | \*\*\* | < .00001 | \*\*\* |
| AH |  | (AL-X) |  | 0.21680 |  | -0.29111 |  | 0.72471 |  | 0.16650 |  | 60 |  | 1.30213 |  | .84829 |  | 1.00000 |  |
|  |  | (AH-X) |  | 0.90008 |  | 0.39217 |  | 1.40799 |  | 0.16650 |  | 60 |  | 5.40601 |  | .00002 | \*\*\* | .00002 | \*\*\* |
|  |  | X |  | -0.63056 |  | -1.15239 |  | -0.10874 |  | 0.17106 |  | 60 |  | -3.68623 |  | .00844 | \*\* | .01032 | \* |
| (AL-X) |  | (AH-X) |  | 0.68328 |  | 0.17537 |  | 1.19119 |  | 0.16650 |  | 60 |  | 4.10388 |  | .00227 | \*\* | .00261 | \*\* |
|  |  | X |  | -0.84736 |  | -1.36919 |  | -0.32554 |  | 0.17106 |  | 60 |  | -4.95363 |  | .00012 | \*\*\* | .00013 | \*\*\* |
| (AH-X) |  | X |  | -1.53064 |  | -2.05247 |  | -1.00882 |  | 0.17106 |  | 60 |  | -8.94806 |  | < .00001 | \*\*\* | < .00001 | \*\*\* |
|  | | | | | | | | | | | | | | | | | | | |
|  |  |  |  |  |  |  |  |  |  |  |  |  |  |  |  |  |  |  |  |
| --- | --- | --- | --- | --- | --- | --- | --- | --- | --- | --- | --- | --- | --- | --- | --- | --- | --- | --- | --- |
| \* p < .05, \*\* p < .01, \*\*\* p < .001 | | | | | | | | | | | | | | | | | | | |
| *Note.*  P-value and confidence intervals adjusted for comparing a family of 7 estimates (confidence intervals corrected using the tukey method). | | | | | | | | | | | | | | | | | | | |

| Letter-Based Grouping - Animal group | | | |
| --- | --- | --- | --- |
| Animal group | | Letter | |
| C |  | b |  |
| OV |  | a |  |
| AL |  | a |  |
| AH |  | b |  |
| AL-X |  | b |  |
| AH-X |  | a |  |
| X |  | c |  |
|  | | | |
|  |  |  |  |
| --- | --- | --- | --- |
| *Note.*  If two or more means share the same grouping symbol, then we cannot show them to be different, but we also did not show them to be the same. | | | |

### Marginal Means

| Marginal Means - Animal group | | | | | | | | | |
| --- | --- | --- | --- | --- | --- | --- | --- | --- | --- |
|  | | | | 95% CI for Mean Difference | | | |  | |
| Animal group | | Marginal Mean | | Lower | | Upper | | SE | |
| C |  | 5.41452 |  | 5.08659 |  | 5.74245 |  | 0.11773 |  |
| OV |  | 4.16287 |  | 3.81719 |  | 4.50854 |  | 0.12410 |  |
| AL |  | 4.68506 |  | 4.33938 |  | 5.03073 |  | 0.12410 |  |
| AH |  | 5.45905 |  | 5.13112 |  | 5.78698 |  | 0.11773 |  |
| AL-X |  | 5.24225 |  | 4.91432 |  | 5.57018 |  | 0.11773 |  |
| AH-X |  | 4.55897 |  | 4.23104 |  | 4.88690 |  | 0.11773 |  |
| X |  | 6.08961 |  | 5.74394 |  | 6.43528 |  | 0.12410 |  |
|  | | | | | | | | | |
|  |  |  |  |  |  |  |  |  |  |
| --- | --- | --- | --- | --- | --- | --- | --- | --- | --- |
| *Note.*  Bonferroni CI adjustment | | | | | | | | | |

## Average cortical area fraction

| ANOVA - Average cortical area fraction (Ct.Ar/Tt.Ar) | | | | | | | | | | | | | | | | | | | | | | | | | |
| --- | --- | --- | --- | --- | --- | --- | --- | --- | --- | --- | --- | --- | --- | --- | --- | --- | --- | --- | --- | --- | --- | --- | --- | --- | --- |
|  | | | | | | | | | | | | | | | | 95% CI for η² | | | |  | | 95% CI for ω² | | | |
| Homogeneity Correction | | Cases | | Sum of Squares | | df | | Mean Square | | F | | p | | η² | | Lower | | Upper | | ω² | | Lower | | Upper | |
| None |  | Animal group |  | 0.06171 |  | 6.00000 |  | 0.01029 |  | 25.76462 |  | < .00001 |  | 0.72039 |  | 0.58501 |  | 0.79383 |  | 0.68922 |  | 0.54019 |  | 0.76993 |  |
|  |  | Residuals |  | 0.02395 |  | 60.00000 |  | 0.00040 |  |  |  |  |  |  |  |  |  |  |  |  |  |  |  |  |  |
| Welch |  | Animal group |  | 0.06171 |  | 6.00000 |  | 0.01029 |  | 20.36143 |  | < .00001 |  | 0.72039 |  | 0.58501 |  | 0.79383 |  | 0.68922 |  | 0.54019 |  | 0.76993 |  |
|  |  | Residuals |  | 0.02395 |  | 26.35790 |  | 0.00091 |  |  |  |  |  |  |  |  |  |  |  |  |  |  |  |  |  |
|  | | | | | | | | | | | | | | | | | | | | | | | | | |
|  |  |  |  |  |  |  |  |  |  |  |  |  |  |  |  |  |  |  |  |  |  |  |  |  |  |
| --- | --- | --- | --- | --- | --- | --- | --- | --- | --- | --- | --- | --- | --- | --- | --- | --- | --- | --- | --- | --- | --- | --- | --- | --- | --- |
| *Note.*  Type III Sum of Squares | | | | | | | | | | | | | | | | | | | | | | | | | |

### Descriptives

| Descriptives - Average cortical area fraction (Ct.Ar/Tt.Ar) | | | | | | | | | | | |
| --- | --- | --- | --- | --- | --- | --- | --- | --- | --- | --- | --- |
| Animal group | | N | | Mean | | SD | | SE | | Coefficient of variation | |
| C |  | 10 |  | 0.32708 |  | 0.01476 |  | 0.00467 |  | 0.04512 |  |
| OV |  | 9 |  | 0.25782 |  | 0.02909 |  | 0.00970 |  | 0.11284 |  |
| AL |  | 9 |  | 0.28593 |  | 0.01670 |  | 0.00557 |  | 0.05840 |  |
| AH |  | 10 |  | 0.32401 |  | 0.01999 |  | 0.00632 |  | 0.06168 |  |
| AL-X |  | 10 |  | 0.31921 |  | 0.01767 |  | 0.00559 |  | 0.05535 |  |
| AH-X |  | 10 |  | 0.27700 |  | 0.01780 |  | 0.00563 |  | 0.06426 |  |
| X |  | 9 |  | 0.35240 |  | 0.02161 |  | 0.00720 |  | 0.06131 |  |
|  | | | | | | | | | | | |

#### Bar plots

#####

### Assumption Checks

| Test for Equality of Variances (Levene's) | | | | | | | |
| --- | --- | --- | --- | --- | --- | --- | --- |
| F | | df1 | | df2 | | p | |
| 0.74453 |  | 6.00000 |  | 60.00000 |  | .61603 |  |
|  | | | | | | | |

### Post Hoc Tests

#### Standard (HSD)

| Post Hoc Comparisons - Animal group | | | | | | | | | | | | | | | | | | | |
| --- | --- | --- | --- | --- | --- | --- | --- | --- | --- | --- | --- | --- | --- | --- | --- | --- | --- | --- | --- |
|  | | | | | | 95% CI for Mean Difference | | | |  | | | | | | | | | |
|  | |  | | Mean Difference | | Lower | | Upper | | SE | | df | | t | | ptukey | | pbonf | |
| C |  | OV |  | 0.06926 |  | 0.04125 |  | 0.09726 |  | 0.00918 |  | 60 |  | 7.54418 |  | < .00001 | \*\*\* | < .00001 | \*\*\* |
|  |  | AL |  | 0.04115 |  | 0.01314 |  | 0.06915 |  | 0.00918 |  | 60 |  | 4.48206 |  | .00064 | \*\*\* | .00071 | \*\*\* |
|  |  | AH |  | 0.00307 |  | -0.02419 |  | 0.03033 |  | 0.00894 |  | 60 |  | 0.34358 |  | .99986 |  | 1.00000 |  |
|  |  | (AL-X) |  | 0.00787 |  | -0.01939 |  | 0.03513 |  | 0.00894 |  | 60 |  | 0.88076 |  | .97412 |  | 1.00000 |  |
|  |  | (AH-X) |  | 0.05008 |  | 0.02282 |  | 0.07734 |  | 0.00894 |  | 60 |  | 5.60465 |  | .00001 | \*\*\* | .00001 | \*\*\* |
|  |  | X |  | -0.02532 |  | -0.05333 |  | 0.00269 |  | 0.00918 |  | 60 |  | -2.75808 |  | .10171 |  | .16159 |  |
| OV |  | AL |  | -0.02811 |  | -0.05684 |  | 0.00062 |  | 0.00942 |  | 60 |  | -2.98458 |  | .05908 |  | .08618 |  |
|  |  | AH |  | -0.06619 |  | -0.09419 |  | -0.03818 |  | 0.00918 |  | 60 |  | -7.20976 |  | < .00001 | \*\*\* | < .00001 | \*\*\* |
|  |  | (AL-X) |  | -0.06139 |  | -0.08939 |  | -0.03338 |  | 0.00918 |  | 60 |  | -6.68691 |  | < .00001 | \*\*\* | < .00001 | \*\*\* |
|  |  | (AH-X) |  | -0.01918 |  | -0.04718 |  | 0.00883 |  | 0.00918 |  | 60 |  | -2.08902 |  | .37234 |  | .86008 |  |
|  |  | X |  | -0.09458 |  | -0.12331 |  | -0.06585 |  | 0.00942 |  | 60 |  | -10.04140 |  | < .00001 | \*\*\* | < .00001 | \*\*\* |
| AL |  | AH |  | -0.03808 |  | -0.06608 |  | -0.01007 |  | 0.00918 |  | 60 |  | -4.14765 |  | .00197 | \*\* | .00225 | \*\* |
|  |  | (AL-X) |  | -0.03328 |  | -0.06128 |  | -0.00527 |  | 0.00918 |  | 60 |  | -3.62479 |  | .01014 | \* | .01254 | \* |
|  |  | (AH-X) |  | 0.00893 |  | -0.01907 |  | 0.03694 |  | 0.00918 |  | 60 |  | 0.97310 |  | .95788 |  | 1.00000 |  |
|  |  | X |  | -0.06647 |  | -0.09520 |  | -0.03773 |  | 0.00942 |  | 60 |  | -7.05682 |  | < .00001 | \*\*\* | < .00001 | \*\*\* |
| AH |  | (AL-X) |  | 0.00480 |  | -0.02246 |  | 0.03206 |  | 0.00894 |  | 60 |  | 0.53719 |  | .99815 |  | 1.00000 |  |
|  |  | (AH-X) |  | 0.04701 |  | 0.01975 |  | 0.07427 |  | 0.00894 |  | 60 |  | 5.26107 |  | .00004 | \*\*\* | .00004 | \*\*\* |
|  |  | X |  | -0.02839 |  | -0.05640 |  | -0.00038 |  | 0.00918 |  | 60 |  | -3.09249 |  | .04488 | \* | .06322 |  |
| (AL-X) |  | (AH-X) |  | 0.04221 |  | 0.01495 |  | 0.06947 |  | 0.00894 |  | 60 |  | 4.72388 |  | .00028 | \*\*\* | .00030 | \*\*\* |
|  |  | X |  | -0.03319 |  | -0.06120 |  | -0.00518 |  | 0.00918 |  | 60 |  | -3.61535 |  | .01043 | \* | .01293 | \* |
| (AH-X) |  | X |  | -0.07540 |  | -0.10341 |  | -0.04739 |  | 0.00918 |  | 60 |  | -8.21324 |  | < .00001 | \*\*\* | < .00001 | \*\*\* |
|  | | | | | | | | | | | | | | | | | | | |
|  |  |  |  |  |  |  |  |  |  |  |  |  |  |  |  |  |  |  |  |
| --- | --- | --- | --- | --- | --- | --- | --- | --- | --- | --- | --- | --- | --- | --- | --- | --- | --- | --- | --- |
| \* p < .05, \*\* p < .01, \*\*\* p < .001 | | | | | | | | | | | | | | | | | | | |
| *Note.*  P-value and confidence intervals adjusted for comparing a family of 7 estimates (confidence intervals corrected using the tukey method). | | | | | | | | | | | | | | | | | | | |

| Letter-Based Grouping - Animal group | | | |
| --- | --- | --- | --- |
| Animal group | | Letter | |
| C |  | bc |  |
| OV |  | a |  |
| AL |  | a |  |
| AH |  | b |  |
| AL-X |  | b |  |
| AH-X |  | a |  |
| X |  | c |  |
|  | | | |
|  |  |  |  |
| --- | --- | --- | --- |
| *Note.*  If two or more means share the same grouping symbol, then we cannot show them to be different, but we also did not show them to be the same. | | | |

### Marginal Means

| Marginal Means - Animal group | | | | | | | | | |
| --- | --- | --- | --- | --- | --- | --- | --- | --- | --- |
|  | | | | 95% CI for Mean Difference | | | |  | |
| Animal group | | Marginal Mean | | Lower | | Upper | | SE | |
| C |  | 0.32708 |  | 0.30948 |  | 0.34468 |  | 0.00632 |  |
| OV |  | 0.25782 |  | 0.23927 |  | 0.27637 |  | 0.00666 |  |
| AL |  | 0.28593 |  | 0.26738 |  | 0.30448 |  | 0.00666 |  |
| AH |  | 0.32401 |  | 0.30641 |  | 0.34161 |  | 0.00632 |  |
| AL-X |  | 0.31921 |  | 0.30161 |  | 0.33681 |  | 0.00632 |  |
| AH-X |  | 0.27700 |  | 0.25940 |  | 0.29460 |  | 0.00632 |  |
| X |  | 0.35240 |  | 0.33385 |  | 0.37095 |  | 0.00666 |  |
|  | | | | | | | | | |
|  |  |  |  |  |  |  |  |  |  |
| --- | --- | --- | --- | --- | --- | --- | --- | --- | --- |
| *Note.*  Bonferroni CI adjustment | | | | | | | | | |

## Average cortical thickness mm

| ANOVA - Average cortical thickness (Ct.Th) mm | | | | | | | | | | | | | | | | | | | | | | | | | |
| --- | --- | --- | --- | --- | --- | --- | --- | --- | --- | --- | --- | --- | --- | --- | --- | --- | --- | --- | --- | --- | --- | --- | --- | --- | --- |
|  | | | | | | | | | | | | | | | | 95% CI for η² | | | |  | | 95% CI for ω² | | | |
| Homogeneity Correction | | Cases | | Sum of Squares | | df | | Mean Square | | F | | p | | η² | | Lower | | Upper | | ω² | | Lower | | Upper | |
| None |  | Animal group |  | 0.01007 |  | 6.00000 |  | 0.00168 |  | 5.79587 |  | .00008 |  | 0.36692 |  | 0.13327 |  | 0.49999 |  | 0.30045 |  | 0.06954 |  | 0.43565 |  |
|  |  | Residuals |  | 0.01737 |  | 60.00000 |  | 0.00029 |  |  |  |  |  |  |  |  |  |  |  |  |  |  |  |  |  |
| Welch |  | Animal group |  | 0.01007 |  | 6.00000 |  | 0.00168 |  | 6.61544 |  | .00025 |  | 0.36692 |  | 0.13327 |  | 0.49999 |  | 0.30045 |  | 0.06954 |  | 0.43565 |  |
|  |  | Residuals |  | 0.01737 |  | 25.90250 |  | 0.00067 |  |  |  |  |  |  |  |  |  |  |  |  |  |  |  |  |  |
|  | | | | | | | | | | | | | | | | | | | | | | | | | |
|  |  |  |  |  |  |  |  |  |  |  |  |  |  |  |  |  |  |  |  |  |  |  |  |  |  |
| --- | --- | --- | --- | --- | --- | --- | --- | --- | --- | --- | --- | --- | --- | --- | --- | --- | --- | --- | --- | --- | --- | --- | --- | --- | --- |
| *Note.*  Type III Sum of Squares | | | | | | | | | | | | | | | | | | | | | | | | | |

### Descriptives

| Descriptives - Average cortical thickness (Ct.Th) mm | | | | | | | | | | | |
| --- | --- | --- | --- | --- | --- | --- | --- | --- | --- | --- | --- |
| Animal group | | N | | Mean | | SD | | SE | | Coefficient of variation | |
| C |  | 10 |  | 0.41464 |  | 0.01926 |  | 0.00609 |  | 0.04646 |  |
| OV |  | 9 |  | 0.40439 |  | 0.02043 |  | 0.00681 |  | 0.05052 |  |
| AL |  | 9 |  | 0.39359 |  | 0.01664 |  | 0.00555 |  | 0.04228 |  |
| AH |  | 10 |  | 0.40475 |  | 0.01748 |  | 0.00553 |  | 0.04318 |  |
| AL-X |  | 10 |  | 0.41594 |  | 0.01468 |  | 0.00464 |  | 0.03529 |  |
| AH-X |  | 10 |  | 0.39360 |  | 0.00854 |  | 0.00270 |  | 0.02169 |  |
| X |  | 9 |  | 0.43118 |  | 0.01979 |  | 0.00660 |  | 0.04590 |  |
|  | | | | | | | | | | | |

#### Bar plots

#####

### Assumption Checks

| Test for Equality of Variances (Levene's) | | | | | | | |
| --- | --- | --- | --- | --- | --- | --- | --- |
| F | | df1 | | df2 | | p | |
| 1.19266 |  | 6.00000 |  | 60.00000 |  | .32259 |  |
|  | | | | | | | |

### Post Hoc Tests

#### Standard (HSD)

| Post Hoc Comparisons - Animal group | | | | | | | | | | | | | | | | | | | |
| --- | --- | --- | --- | --- | --- | --- | --- | --- | --- | --- | --- | --- | --- | --- | --- | --- | --- | --- | --- |
|  | | | | | | 95% CI for Mean Difference | | | |  | | | | | | | | | |
|  | |  | | Mean Difference | | Lower | | Upper | | SE | | df | | t | | ptukey | | pbonf | |
| C |  | OV |  | 0.01025 |  | -0.01360 |  | 0.03410 |  | 0.00782 |  | 60 |  | 1.31121 |  | .84406 |  | 1.00000 |  |
|  |  | AL |  | 0.02105 |  | -0.00280 |  | 0.04490 |  | 0.00782 |  | 60 |  | 2.69263 |  | .11794 |  | .19269 |  |
|  |  | AH |  | 0.00989 |  | -0.01332 |  | 0.03310 |  | 0.00761 |  | 60 |  | 1.29969 |  | .84941 |  | 1.00000 |  |
|  |  | (AL-X) |  | -0.00130 |  | -0.02451 |  | 0.02191 |  | 0.00761 |  | 60 |  | -0.17084 |  | 1.00000 |  | 1.00000 |  |
|  |  | (AH-X) |  | 0.02104 |  | -0.00217 |  | 0.04425 |  | 0.00761 |  | 60 |  | 2.76496 |  | .10012 |  | .15860 |  |
|  |  | X |  | -0.01654 |  | -0.04039 |  | 0.00731 |  | 0.00782 |  | 60 |  | -2.11534 |  | .35726 |  | .80980 |  |
| OV |  | AL |  | 0.01080 |  | -0.01367 |  | 0.03527 |  | 0.00802 |  | 60 |  | 1.34644 |  | .82711 |  | 1.00000 |  |
|  |  | AH |  | -0.00036 |  | -0.02421 |  | 0.02349 |  | 0.00782 |  | 60 |  | -0.04619 |  | 1.00000 |  | 1.00000 |  |
|  |  | (AL-X) |  | -0.01155 |  | -0.03540 |  | 0.01230 |  | 0.00782 |  | 60 |  | -1.47750 |  | .75678 |  | 1.00000 |  |
|  |  | (AH-X) |  | 0.01079 |  | -0.01306 |  | 0.03464 |  | 0.00782 |  | 60 |  | 1.38000 |  | .81014 |  | 1.00000 |  |
|  |  | X |  | -0.02679 |  | -0.05126 |  | -0.00232 |  | 0.00802 |  | 60 |  | -3.33979 |  | .02308 | \* | .03036 | \* |
| AL |  | AH |  | -0.01116 |  | -0.03501 |  | 0.01269 |  | 0.00782 |  | 60 |  | -1.42761 |  | .78480 |  | 1.00000 |  |
|  |  | (AL-X) |  | -0.02235 |  | -0.04620 |  | 0.00150 |  | 0.00782 |  | 60 |  | -2.85892 |  | .08033 |  | .12259 |  |
|  |  | (AH-X) |  | -0.00001 |  | -0.02386 |  | 0.02384 |  | 0.00782 |  | 60 |  | -0.00142 |  | 1.00000 |  | 1.00000 |  |
|  |  | X |  | -0.03759 |  | -0.06206 |  | -0.01312 |  | 0.00802 |  | 60 |  | -4.68623 |  | .00032 | \*\*\* | .00035 | \*\*\* |
| AH |  | (AL-X) |  | -0.01119 |  | -0.03440 |  | 0.01202 |  | 0.00761 |  | 60 |  | -1.47053 |  | .76078 |  | 1.00000 |  |
|  |  | (AH-X) |  | 0.01115 |  | -0.01206 |  | 0.03436 |  | 0.00761 |  | 60 |  | 1.46527 |  | .76377 |  | 1.00000 |  |
|  |  | X |  | -0.02643 |  | -0.05028 |  | -0.00258 |  | 0.00782 |  | 60 |  | -3.38036 |  | .02060 | \* | .02684 | \* |
| (AL-X) |  | (AH-X) |  | 0.02234 |  | -0.00087 |  | 0.04555 |  | 0.00761 |  | 60 |  | 2.93580 |  | .06667 |  | .09892 |  |
|  |  | X |  | -0.01524 |  | -0.03909 |  | 0.00861 |  | 0.00782 |  | 60 |  | -1.94905 |  | .45715 |  | 1.00000 |  |
| (AH-X) |  | X |  | -0.03758 |  | -0.06143 |  | -0.01373 |  | 0.00782 |  | 60 |  | -4.80655 |  | .00021 | \*\*\* | .00022 | \*\*\* |
|  | | | | | | | | | | | | | | | | | | | |
|  |  |  |  |  |  |  |  |  |  |  |  |  |  |  |  |  |  |  |  |
| --- | --- | --- | --- | --- | --- | --- | --- | --- | --- | --- | --- | --- | --- | --- | --- | --- | --- | --- | --- |
| \* p < .05, \*\*\* p < .001 | | | | | | | | | | | | | | | | | | | |
| *Note.*  P-value and confidence intervals adjusted for comparing a family of 7 estimates (confidence intervals corrected using the tukey method). | | | | | | | | | | | | | | | | | | | |

| Letter-Based Grouping - Animal group | | | |
| --- | --- | --- | --- |
| Animal group | | Letter | |
| C |  | ab |  |
| OV |  | a |  |
| AL |  | a |  |
| AH |  | a |  |
| AL-X |  | ab |  |
| AH-X |  | a |  |
| X |  | b |  |
|  | | | |
|  |  |  |  |
| --- | --- | --- | --- |
| *Note.*  If two or more means share the same grouping symbol, then we cannot show them to be different, but we also did not show them to be the same. | | | |

### Marginal Means

| Marginal Means - Animal group | | | | | | | | | |
| --- | --- | --- | --- | --- | --- | --- | --- | --- | --- |
|  | | | | 95% CI for Mean Difference | | | |  | |
| Animal group | | Marginal Mean | | Lower | | Upper | | SE | |
| C |  | 0.41464 |  | 0.39965 |  | 0.42963 |  | 0.00538 |  |
| OV |  | 0.40439 |  | 0.38859 |  | 0.42019 |  | 0.00567 |  |
| AL |  | 0.39359 |  | 0.37779 |  | 0.40939 |  | 0.00567 |  |
| AH |  | 0.40475 |  | 0.38976 |  | 0.41974 |  | 0.00538 |  |
| AL-X |  | 0.41594 |  | 0.40095 |  | 0.43093 |  | 0.00538 |  |
| AH-X |  | 0.39360 |  | 0.37861 |  | 0.40859 |  | 0.00538 |  |
| X |  | 0.43118 |  | 0.41538 |  | 0.44698 |  | 0.00567 |  |
|  | | | | | | | | | |
|  |  |  |  |  |  |  |  |  |  |
| --- | --- | --- | --- | --- | --- | --- | --- | --- | --- |
| *Note.*  Bonferroni CI adjustment | | | | | | | | | |

## Endocortical perimeter mm

| ANOVA - Endocortical perimeter (Ec.Pm) mm | | | | | | | | | | | | | | | | | | | | | | | | | |
| --- | --- | --- | --- | --- | --- | --- | --- | --- | --- | --- | --- | --- | --- | --- | --- | --- | --- | --- | --- | --- | --- | --- | --- | --- | --- |
|  | | | | | | | | | | | | | | | | 95% CI for η² | | | |  | | 95% CI for ω² | | | |
| Homogeneity Correction | | Cases | | Sum of Squares | | df | | Mean Square | | F | | p | | η² | | Lower | | Upper | | ω² | | Lower | | Upper | |
| None |  | Animal group |  | 305.35613 |  | 6.00000 |  | 50.89269 |  | 32.34835 |  | < .00001 |  | 0.76386 |  | 0.64838 |  | 0.82676 |  | 0.73735 |  | 0.60961 |  | 0.80673 |  |
|  |  | Residuals |  | 94.39620 |  | 60.00000 |  | 1.57327 |  |  |  |  |  |  |  |  |  |  |  |  |  |  |  |  |  |
| Welch |  | Animal group |  | 305.35613 |  | 6.00000 |  | 50.89269 |  | 27.33963 |  | < .00001 |  | 0.76386 |  | 0.64838 |  | 0.82676 |  | 0.73735 |  | 0.60961 |  | 0.80673 |  |
|  |  | Residuals |  | 94.39620 |  | 26.33110 |  | 3.58497 |  |  |  |  |  |  |  |  |  |  |  |  |  |  |  |  |  |
|  | | | | | | | | | | | | | | | | | | | | | | | | | |
|  |  |  |  |  |  |  |  |  |  |  |  |  |  |  |  |  |  |  |  |  |  |  |  |  |  |
| --- | --- | --- | --- | --- | --- | --- | --- | --- | --- | --- | --- | --- | --- | --- | --- | --- | --- | --- | --- | --- | --- | --- | --- | --- | --- |
| *Note.*  Type III Sum of Squares | | | | | | | | | | | | | | | | | | | | | | | | | |

### Descriptives

| Descriptives - Endocortical perimeter (Ec.Pm) mm | | | | | | | | | | | |
| --- | --- | --- | --- | --- | --- | --- | --- | --- | --- | --- | --- |
| Animal group | | N | | Mean | | SD | | SE | | Coefficient of variation | |
| C |  | 10 |  | 23.13400 |  | 1.71776 |  | 0.54320 |  | 0.07425 |  |
| OV |  | 9 |  | 18.82752 |  | 1.47952 |  | 0.49317 |  | 0.07858 |  |
| AL |  | 9 |  | 20.74687 |  | 0.87527 |  | 0.29176 |  | 0.04219 |  |
| AH |  | 10 |  | 23.79100 |  | 0.88495 |  | 0.27985 |  | 0.03720 |  |
| AL-X |  | 10 |  | 22.32202 |  | 0.93182 |  | 0.29467 |  | 0.04174 |  |
| AH-X |  | 10 |  | 20.33415 |  | 1.10588 |  | 0.34971 |  | 0.05439 |  |
| X |  | 9 |  | 25.77717 |  | 1.51367 |  | 0.50456 |  | 0.05872 |  |
|  | | | | | | | | | | | |

#### Bar plots

#####

### Assumption Checks

| Test for Equality of Variances (Levene's) | | | | | | | |
| --- | --- | --- | --- | --- | --- | --- | --- |
| F | | df1 | | df2 | | p | |
| 1.48370 |  | 6.00000 |  | 60.00000 |  | .19917 |  |
|  | | | | | | | |

### Post Hoc Tests

#### Standard (HSD)

| Post Hoc Comparisons - Animal group | | | | | | | | | | | | | | | | | | | |
| --- | --- | --- | --- | --- | --- | --- | --- | --- | --- | --- | --- | --- | --- | --- | --- | --- | --- | --- | --- |
|  | | | | | | 95% CI for Mean Difference | | | |  | | | | | | | | | |
|  | |  | | Mean Difference | | Lower | | Upper | | SE | | df | | t | | ptukey | | pbonf | |
| C |  | OV |  | 4.30648 |  | 2.54841 |  | 6.06455 |  | 0.57631 |  | 60 |  | 7.47248 |  | < .00001 | \*\*\* | < .00001 | \*\*\* |
|  |  | AL |  | 2.38713 |  | 0.62906 |  | 4.14521 |  | 0.57631 |  | 60 |  | 4.14209 |  | .00201 | \*\* | .00230 | \*\* |
|  |  | AH |  | -0.65700 |  | -2.36818 |  | 1.05418 |  | 0.56094 |  | 60 |  | -1.17125 |  | .90211 |  | 1.00000 |  |
|  |  | (AL-X) |  | 0.81198 |  | -0.89920 |  | 2.52316 |  | 0.56094 |  | 60 |  | 1.44753 |  | .77378 |  | 1.00000 |  |
|  |  | (AH-X) |  | 2.79985 |  | 1.08867 |  | 4.51103 |  | 0.56094 |  | 60 |  | 4.99135 |  | .00011 | \*\*\* | .00011 | \*\*\* |
|  |  | X |  | -2.64317 |  | -4.40124 |  | -0.88509 |  | 0.57631 |  | 60 |  | -4.58635 |  | .00045 | \*\*\* | .00049 | \*\*\* |
| OV |  | AL |  | -1.91934 |  | -3.72309 |  | -0.11560 |  | 0.59128 |  | 60 |  | -3.24607 |  | .02986 | \* | .04024 | \* |
|  |  | AH |  | -4.96348 |  | -6.72155 |  | -3.20541 |  | 0.57631 |  | 60 |  | -8.61249 |  | < .00001 | \*\*\* | < .00001 | \*\*\* |
|  |  | (AL-X) |  | -3.49450 |  | -5.25257 |  | -1.73643 |  | 0.57631 |  | 60 |  | -6.06356 |  | < .00001 | \*\*\* | < .00001 | \*\*\* |
|  |  | (AH-X) |  | -1.50663 |  | -3.26470 |  | 0.25144 |  | 0.57631 |  | 60 |  | -2.61426 |  | .14003 |  | .23708 |  |
|  |  | X |  | -6.94964 |  | -8.75339 |  | -5.14590 |  | 0.59128 |  | 60 |  | -11.75350 |  | < .00001 | \*\*\* | < .00001 | \*\*\* |
| AL |  | AH |  | -3.04413 |  | -4.80221 |  | -1.28606 |  | 0.57631 |  | 60 |  | -5.28210 |  | .00004 | \*\*\* | .00004 | \*\*\* |
|  |  | (AL-X) |  | -1.57515 |  | -3.33323 |  | 0.18292 |  | 0.57631 |  | 60 |  | -2.73316 |  | .10766 |  | .17284 |  |
|  |  | (AH-X) |  | 0.41272 |  | -1.34536 |  | 2.17079 |  | 0.57631 |  | 60 |  | 0.71613 |  | .99109 |  | 1.00000 |  |
|  |  | X |  | -5.03030 |  | -6.83404 |  | -3.22656 |  | 0.59128 |  | 60 |  | -8.50743 |  | < .00001 | \*\*\* | < .00001 | \*\*\* |
| AH |  | (AL-X) |  | 1.46898 |  | -0.24220 |  | 3.18016 |  | 0.56094 |  | 60 |  | 2.61878 |  | .13867 |  | .23429 |  |
|  |  | (AH-X) |  | 3.45685 |  | 1.74567 |  | 5.16803 |  | 0.56094 |  | 60 |  | 6.16260 |  | < .00001 | \*\*\* | < .00001 | \*\*\* |
|  |  | X |  | -1.98617 |  | -3.74424 |  | -0.22809 |  | 0.57631 |  | 60 |  | -3.44634 |  | .01708 | \* | .02192 | \* |
| (AL-X) |  | (AH-X) |  | 1.98787 |  | 0.27669 |  | 3.69905 |  | 0.56094 |  | 60 |  | 3.54382 |  | .01288 | \* | .01619 | \* |
|  |  | X |  | -3.45515 |  | -5.21322 |  | -1.69707 |  | 0.57631 |  | 60 |  | -5.99528 |  | < .00001 | \*\*\* | < .00001 | \*\*\* |
| (AH-X) |  | X |  | -5.44302 |  | -7.20109 |  | -3.68494 |  | 0.57631 |  | 60 |  | -9.44458 |  | < .00001 | \*\*\* | < .00001 | \*\*\* |
|  | | | | | | | | | | | | | | | | | | | |
|  |  |  |  |  |  |  |  |  |  |  |  |  |  |  |  |  |  |  |  |
| --- | --- | --- | --- | --- | --- | --- | --- | --- | --- | --- | --- | --- | --- | --- | --- | --- | --- | --- | --- |
| \* p < .05, \*\* p < .01, \*\*\* p < .001 | | | | | | | | | | | | | | | | | | | |
| *Note.*  P-value and confidence intervals adjusted for comparing a family of 7 estimates (confidence intervals corrected using the tukey method). | | | | | | | | | | | | | | | | | | | |

| Letter-Based Grouping - Animal group | | | |
| --- | --- | --- | --- |
| Animal group | | Letter | |
| C |  | d |  |
| OV |  | a |  |
| AL |  | bc |  |
| AH |  | d |  |
| AL-X |  | cd |  |
| AH-X |  | ab |  |
| X |  | e |  |
|  | | | |
|  |  |  |  |
| --- | --- | --- | --- |
| *Note.*  If two or more means share the same grouping symbol, then we cannot show them to be different, but we also did not show them to be the same. | | | |

### Marginal Means

| Marginal Means - Animal group | | | | | | | | | |
| --- | --- | --- | --- | --- | --- | --- | --- | --- | --- |
|  | | | | 95% CI for Mean Difference | | | |  | |
| Animal group | | Marginal Mean | | Lower | | Upper | | SE | |
| C |  | 23.13400 |  | 22.02916 |  | 24.23884 |  | 0.39664 |  |
| OV |  | 18.82752 |  | 17.66292 |  | 19.99213 |  | 0.41810 |  |
| AL |  | 20.74687 |  | 19.58226 |  | 21.91147 |  | 0.41810 |  |
| AH |  | 23.79100 |  | 22.68616 |  | 24.89584 |  | 0.39664 |  |
| AL-X |  | 22.32202 |  | 21.21718 |  | 23.42686 |  | 0.39664 |  |
| AH-X |  | 20.33415 |  | 19.22931 |  | 21.43899 |  | 0.39664 |  |
| X |  | 25.77717 |  | 24.61256 |  | 26.94177 |  | 0.41810 |  |
|  | | | | | | | | | |
|  |  |  |  |  |  |  |  |  |  |
| --- | --- | --- | --- | --- | --- | --- | --- | --- | --- |
| *Note.*  Bonferroni CI adjustment | | | | | | | | | |

## Endocortical surface mm2

| ANOVA - Endocortical surface (3D) (Ec.S3D) mm² | | | | | | | | | | | | | | | | | | | | | | | | | |
| --- | --- | --- | --- | --- | --- | --- | --- | --- | --- | --- | --- | --- | --- | --- | --- | --- | --- | --- | --- | --- | --- | --- | --- | --- | --- |
|  | | | | | | | | | | | | | | | | 95% CI for η² | | | |  | | 95% CI for ω² | | | |
| Homogeneity Correction | | Cases | | Sum of Squares | | df | | Mean Square | | F | | p | | η² | | Lower | | Upper | | ω² | | Lower | | Upper | |
| None |  | Animal group |  | 70260.30029 |  | 6.00000 |  | 11710.05005 |  | 41.18311 |  | < .00001 |  | 0.80462 |  | 0.70853 |  | 0.85727 |  | 0.78254 |  | 0.67586 |  | 0.84078 |  |
|  |  | Residuals |  | 17060.46564 |  | 60.00000 |  | 284.34109 |  |  |  |  |  |  |  |  |  |  |  |  |  |  |  |  |  |
| Welch |  | Animal group |  | 70260.30029 |  | 6.00000 |  | 11710.05005 |  | 37.49222 |  | < .00001 |  | 0.80462 |  | 0.70853 |  | 0.85727 |  | 0.78254 |  | 0.67586 |  | 0.84078 |  |
|  |  | Residuals |  | 17060.46564 |  | 26.48174 |  | 644.23500 |  |  |  |  |  |  |  |  |  |  |  |  |  |  |  |  |  |
|  | | | | | | | | | | | | | | | | | | | | | | | | | |
|  |  |  |  |  |  |  |  |  |  |  |  |  |  |  |  |  |  |  |  |  |  |  |  |  |  |
| --- | --- | --- | --- | --- | --- | --- | --- | --- | --- | --- | --- | --- | --- | --- | --- | --- | --- | --- | --- | --- | --- | --- | --- | --- | --- |
| *Note.*  Type III Sum of Squares | | | | | | | | | | | | | | | | | | | | | | | | | |

### Descriptives

| Descriptives - Endocortical surface (3D) (Ec.S3D) mm² | | | | | | | | | | | |
| --- | --- | --- | --- | --- | --- | --- | --- | --- | --- | --- | --- |
| Animal group | | N | | Mean | | SD | | SE | | Coefficient of variation | |
| C |  | 10 |  | 295.46392 |  | 22.44363 |  | 7.09730 |  | 0.07596 |  |
| OV |  | 9 |  | 232.59904 |  | 19.02657 |  | 6.34219 |  | 0.08180 |  |
| AL |  | 9 |  | 259.47508 |  | 13.70932 |  | 4.56977 |  | 0.05283 |  |
| AH |  | 10 |  | 303.57891 |  | 16.34735 |  | 5.16949 |  | 0.05385 |  |
| AL-X |  | 10 |  | 284.39630 |  | 13.55117 |  | 4.28526 |  | 0.04765 |  |
| AH-X |  | 10 |  | 253.02799 |  | 14.75244 |  | 4.66513 |  | 0.05830 |  |
| X |  | 9 |  | 338.61907 |  | 16.24357 |  | 5.41452 |  | 0.04797 |  |
|  | | | | | | | | | | | |

#### Bar plots

#####

### Assumption Checks

| Test for Equality of Variances (Levene's) | | | | | | | |
| --- | --- | --- | --- | --- | --- | --- | --- |
| F | | df1 | | df2 | | p | |
| 0.78114 |  | 6.00000 |  | 60.00000 |  | .58801 |  |
|  | | | | | | | |

### Post Hoc Tests

#### Standard (HSD)

| Post Hoc Comparisons - Animal group | | | | | | | | | | | | | | | | | | | |
| --- | --- | --- | --- | --- | --- | --- | --- | --- | --- | --- | --- | --- | --- | --- | --- | --- | --- | --- | --- |
|  | | | | | | 95% CI for Mean Difference | | | |  | | | | | | | | | |
|  | |  | | Mean Difference | | Lower | | Upper | | SE | | df | | t | | ptukey | | pbonf | |
| C |  | OV |  | 62.86488 |  | 39.22992 |  | 86.49984 |  | 7.74775 |  | 60 |  | 8.11396 |  | < .00001 | \*\*\* | < .00001 | \*\*\* |
|  |  | AL |  | 35.98884 |  | 12.35388 |  | 59.62380 |  | 7.74775 |  | 60 |  | 4.64507 |  | .00037 | \*\*\* | .00040 | \*\*\* |
|  |  | AH |  | -8.11499 |  | -31.11957 |  | 14.88959 |  | 7.54110 |  | 60 |  | -1.07610 |  | .93269 |  | 1.00000 |  |
|  |  | (AL-X) |  | 11.06762 |  | -11.93696 |  | 34.07220 |  | 7.54110 |  | 60 |  | 1.46764 |  | .76242 |  | 1.00000 |  |
|  |  | (AH-X) |  | 42.43593 |  | 19.43135 |  | 65.44051 |  | 7.54110 |  | 60 |  | 5.62729 |  | .00001 | \*\*\* | .00001 | \*\*\* |
|  |  | X |  | -43.15515 |  | -66.79011 |  | -19.52019 |  | 7.74775 |  | 60 |  | -5.57003 |  | .00001 | \*\*\* | .00001 | \*\*\* |
| OV |  | AL |  | -26.87603 |  | -51.12499 |  | -2.62708 |  | 7.94902 |  | 60 |  | -3.38105 |  | .02056 | \* | .02678 | \* |
|  |  | AH |  | -70.97987 |  | -94.61483 |  | -47.34491 |  | 7.74775 |  | 60 |  | -9.16136 |  | < .00001 | \*\*\* | < .00001 | \*\*\* |
|  |  | (AL-X) |  | -51.79726 |  | -75.43222 |  | -28.16230 |  | 7.74775 |  | 60 |  | -6.68546 |  | < .00001 | \*\*\* | < .00001 | \*\*\* |
|  |  | (AH-X) |  | -20.42895 |  | -44.06391 |  | 3.20601 |  | 7.74775 |  | 60 |  | -2.63676 |  | .13338 |  | .22347 |  |
|  |  | X |  | -106.02002 |  | -130.26898 |  | -81.77106 |  | 7.94902 |  | 60 |  | -13.33750 |  | < .00001 | \*\*\* | < .00001 | \*\*\* |
| AL |  | AH |  | -44.10383 |  | -67.73879 |  | -20.46887 |  | 7.74775 |  | 60 |  | -5.69247 |  | < .00001 | \*\*\* | < .00001 | \*\*\* |
|  |  | (AL-X) |  | -24.92122 |  | -48.55618 |  | -1.28626 |  | 7.74775 |  | 60 |  | -3.21658 |  | .03234 | \* | .04393 | \* |
|  |  | (AH-X) |  | 6.44709 |  | -17.18787 |  | 30.08205 |  | 7.74775 |  | 60 |  | 0.83212 |  | .98055 |  | 1.00000 |  |
|  |  | X |  | -79.14399 |  | -103.39295 |  | -54.89503 |  | 7.94902 |  | 60 |  | -9.95645 |  | < .00001 | \*\*\* | < .00001 | \*\*\* |
| AH |  | (AL-X) |  | 19.18261 |  | -3.82197 |  | 42.18719 |  | 7.54110 |  | 60 |  | 2.54374 |  | .16254 |  | .28479 |  |
|  |  | (AH-X) |  | 50.55092 |  | 27.54634 |  | 73.55550 |  | 7.54110 |  | 60 |  | 6.70339 |  | < .00001 | \*\*\* | < .00001 | \*\*\* |
|  |  | X |  | -35.04016 |  | -58.67512 |  | -11.40520 |  | 7.74775 |  | 60 |  | -4.52263 |  | .00056 | \*\*\* | .00062 | \*\*\* |
| (AL-X) |  | (AH-X) |  | 31.36831 |  | 8.36373 |  | 54.37289 |  | 7.54110 |  | 60 |  | 4.15965 |  | .00190 | \*\* | .00216 | \*\* |
|  |  | X |  | -54.22277 |  | -77.85773 |  | -30.58781 |  | 7.74775 |  | 60 |  | -6.99852 |  | < .00001 | \*\*\* | < .00001 | \*\*\* |
| (AH-X) |  | X |  | -85.59108 |  | -109.22604 |  | -61.95612 |  | 7.74775 |  | 60 |  | -11.04722 |  | < .00001 | \*\*\* | < .00001 | \*\*\* |
|  | | | | | | | | | | | | | | | | | | | |
|  |  |  |  |  |  |  |  |  |  |  |  |  |  |  |  |  |  |  |  |
| --- | --- | --- | --- | --- | --- | --- | --- | --- | --- | --- | --- | --- | --- | --- | --- | --- | --- | --- | --- |
| \* p < .05, \*\* p < .01, \*\*\* p < .001 | | | | | | | | | | | | | | | | | | | |
| *Note.*  P-value and confidence intervals adjusted for comparing a family of 7 estimates (confidence intervals corrected using the tukey method). | | | | | | | | | | | | | | | | | | | |

| Letter-Based Grouping - Animal group | | | |
| --- | --- | --- | --- |
| Animal group | | Letter | |
| C |  | c |  |
| OV |  | a |  |
| AL |  | b |  |
| AH |  | c |  |
| AL-X |  | c |  |
| AH-X |  | ab |  |
| X |  | d |  |
|  | | | |
|  |  |  |  |
| --- | --- | --- | --- |
| *Note.*  If two or more means share the same grouping symbol, then we cannot show them to be different, but we also did not show them to be the same. | | | |

### Marginal Means

| Marginal Means - Animal group | | | | | | | | | |
| --- | --- | --- | --- | --- | --- | --- | --- | --- | --- |
|  | | | | 95% CI for Mean Difference | | | |  | |
| Animal group | | Marginal Mean | | Lower | | Upper | | SE | |
| C |  | 295.46392 |  | 280.61078 |  | 310.31706 |  | 5.33236 |  |
| OV |  | 232.59904 |  | 216.94246 |  | 248.25563 |  | 5.62081 |  |
| AL |  | 259.47508 |  | 243.81849 |  | 275.13166 |  | 5.62081 |  |
| AH |  | 303.57891 |  | 288.72577 |  | 318.43205 |  | 5.33236 |  |
| AL-X |  | 284.39630 |  | 269.54316 |  | 299.24944 |  | 5.33236 |  |
| AH-X |  | 253.02799 |  | 238.17485 |  | 267.88113 |  | 5.33236 |  |
| X |  | 338.61907 |  | 322.96248 |  | 354.27565 |  | 5.62081 |  |
|  | | | | | | | | | |
|  |  |  |  |  |  |  |  |  |  |
| --- | --- | --- | --- | --- | --- | --- | --- | --- | --- |
| *Note.*  Bonferroni CI adjustment | | | | | | | | | |

## Average marrow area mm2

| ANOVA - Average marrow area (Ma.Ar) mm² | | | | | | | | | | | | | | | | | | | | | | | | | |
| --- | --- | --- | --- | --- | --- | --- | --- | --- | --- | --- | --- | --- | --- | --- | --- | --- | --- | --- | --- | --- | --- | --- | --- | --- | --- |
|  | | | | | | | | | | | | | | | | 95% CI for η² | | | |  | | 95% CI for ω² | | | |
| Homogeneity Correction | | Cases | | Sum of Squares | | df | | Mean Square | | F | | p | | η² | | Lower | | Upper | | ω² | | Lower | | Upper | |
| None |  | Animal group |  | 7.78457 |  | 6.00000 |  | 1.29743 |  | 2.29539 |  | .04632 |  | 0.18669 |  | 0.00000 |  | 0.31134 |  | 0.10395 |  | 0.00000 |  | 0.19931 |  |
|  |  | Residuals |  | 33.91396 |  | 60.00000 |  | 0.56523 |  |  |  |  |  |  |  |  |  |  |  |  |  |  |  |  |  |
| Welch |  | Animal group |  | 7.78457 |  | 6.00000 |  | 1.29743 |  | 2.01699 |  | .09920 |  | 0.18669 |  | 0.00000 |  | 0.31134 |  | 0.10395 |  | 0.00000 |  | 0.19931 |  |
|  |  | Residuals |  | 33.91396 |  | 26.22606 |  | 1.29314 |  |  |  |  |  |  |  |  |  |  |  |  |  |  |  |  |  |
|  | | | | | | | | | | | | | | | | | | | | | | | | | |
|  |  |  |  |  |  |  |  |  |  |  |  |  |  |  |  |  |  |  |  |  |  |  |  |  |  |
| --- | --- | --- | --- | --- | --- | --- | --- | --- | --- | --- | --- | --- | --- | --- | --- | --- | --- | --- | --- | --- | --- | --- | --- | --- | --- |
| *Note.*  Type III Sum of Squares | | | | | | | | | | | | | | | | | | | | | | | | | |

### Descriptives

| Descriptives - Average marrow area (Ma.Ar) mm² | | | | | | | | | | | |
| --- | --- | --- | --- | --- | --- | --- | --- | --- | --- | --- | --- |
| Animal group | | N | | Mean | | SD | | SE | | Coefficient of variation | |
| C |  | 10 |  | 11.15302 |  | 0.76331 |  | 0.24138 |  | 0.06844 |  |
| OV |  | 9 |  | 12.03120 |  | 1.15723 |  | 0.38574 |  | 0.09619 |  |
| AL |  | 9 |  | 11.70239 |  | 0.77725 |  | 0.25908 |  | 0.06642 |  |
| AH |  | 10 |  | 11.40426 |  | 0.64736 |  | 0.20471 |  | 0.05677 |  |
| AL-X |  | 10 |  | 11.18702 |  | 0.45442 |  | 0.14370 |  | 0.04062 |  |
| AH-X |  | 10 |  | 11.91194 |  | 0.70876 |  | 0.22413 |  | 0.05950 |  |
| X |  | 9 |  | 11.18278 |  | 0.60955 |  | 0.20318 |  | 0.05451 |  |
|  | | | | | | | | | | | |

#### Bar plots

#####

### Assumption Checks

| Test for Equality of Variances (Levene's) | | | | | | | |
| --- | --- | --- | --- | --- | --- | --- | --- |
| F | | df1 | | df2 | | p | |
| 0.90822 |  | 6.00000 |  | 60.00000 |  | .49526 |  |
|  | | | | | | | |

### Post Hoc Tests

#### Standard (HSD)

| Post Hoc Comparisons - Animal group | | | | | | | | | | | | | | | | | | | |
| --- | --- | --- | --- | --- | --- | --- | --- | --- | --- | --- | --- | --- | --- | --- | --- | --- | --- | --- | --- |
|  | | | | | | 95% CI for Mean Difference | | | |  | | | | | | | | | |
|  | |  | | Mean Difference | | Lower | | Upper | | SE | | df | | t | | ptukey | | pbonf | |
| C |  | OV |  | -0.87818 |  | -1.93196 |  | 0.17560 |  | 0.34544 |  | 60 |  | -2.54223 |  | .16305 |  | .28590 |  |
|  |  | AL |  | -0.54937 |  | -1.60315 |  | 0.50441 |  | 0.34544 |  | 60 |  | -1.59036 |  | .68883 |  | 1.00000 |  |
|  |  | AH |  | -0.25124 |  | -1.27691 |  | 0.77443 |  | 0.33622 |  | 60 |  | -0.74724 |  | .98885 |  | 1.00000 |  |
|  |  | (AL-X) |  | -0.03400 |  | -1.05967 |  | 0.99167 |  | 0.33622 |  | 60 |  | -0.10112 |  | 1.00000 |  | 1.00000 |  |
|  |  | (AH-X) |  | -0.75892 |  | -1.78459 |  | 0.26675 |  | 0.33622 |  | 60 |  | -2.25719 |  | .28180 |  | .58064 |  |
|  |  | X |  | -0.02976 |  | -1.08353 |  | 1.02402 |  | 0.34544 |  | 60 |  | -0.08615 |  | 1.00000 |  | 1.00000 |  |
| OV |  | AL |  | 0.32881 |  | -0.75234 |  | 1.40996 |  | 0.35441 |  | 60 |  | 0.92777 |  | .96655 |  | 1.00000 |  |
|  |  | AH |  | 0.62694 |  | -0.42684 |  | 1.68072 |  | 0.34544 |  | 60 |  | 1.81492 |  | .54353 |  | 1.00000 |  |
|  |  | (AL-X) |  | 0.84418 |  | -0.20960 |  | 1.89796 |  | 0.34544 |  | 60 |  | 2.44380 |  | .19898 |  | .36731 |  |
|  |  | (AH-X) |  | 0.11926 |  | -0.93452 |  | 1.17304 |  | 0.34544 |  | 60 |  | 0.34524 |  | .99985 |  | 1.00000 |  |
|  |  | X |  | 0.84842 |  | -0.23273 |  | 1.92957 |  | 0.35441 |  | 60 |  | 2.39389 |  | .21923 |  | .41609 |  |
| AL |  | AH |  | 0.29813 |  | -0.75565 |  | 1.35191 |  | 0.34544 |  | 60 |  | 0.86305 |  | .97662 |  | 1.00000 |  |
|  |  | (AL-X) |  | 0.51537 |  | -0.53841 |  | 1.56915 |  | 0.34544 |  | 60 |  | 1.49193 |  | .74841 |  | 1.00000 |  |
|  |  | (AH-X) |  | -0.20955 |  | -1.26333 |  | 0.84423 |  | 0.34544 |  | 60 |  | -0.60663 |  | .99637 |  | 1.00000 |  |
|  |  | X |  | 0.51961 |  | -0.56154 |  | 1.60076 |  | 0.35441 |  | 60 |  | 1.46613 |  | .76329 |  | 1.00000 |  |
| AH |  | (AL-X) |  | 0.21724 |  | -0.80843 |  | 1.24291 |  | 0.33622 |  | 60 |  | 0.64612 |  | .99488 |  | 1.00000 |  |
|  |  | (AH-X) |  | -0.50768 |  | -1.53335 |  | 0.51799 |  | 0.33622 |  | 60 |  | -1.50995 |  | .73783 |  | 1.00000 |  |
|  |  | X |  | 0.22148 |  | -0.83229 |  | 1.27526 |  | 0.34544 |  | 60 |  | 0.64116 |  | .99509 |  | 1.00000 |  |
| (AL-X) |  | (AH-X) |  | -0.72492 |  | -1.75059 |  | 0.30075 |  | 0.33622 |  | 60 |  | -2.15606 |  | .33457 |  | .73706 |  |
|  |  | X |  | 0.00424 |  | -1.04953 |  | 1.05802 |  | 0.34544 |  | 60 |  | 0.01228 |  | 1.00000 |  | 1.00000 |  |
| (AH-X) |  | X |  | 0.72916 |  | -0.32461 |  | 1.78294 |  | 0.34544 |  | 60 |  | 2.11084 |  | .35982 |  | .81821 |  |
|  | | | | | | | | | | | | | | | | | | | |
|  |  |  |  |  |  |  |  |  |  |  |  |  |  |  |  |  |  |  |  |
| --- | --- | --- | --- | --- | --- | --- | --- | --- | --- | --- | --- | --- | --- | --- | --- | --- | --- | --- | --- |
| *Note.*  P-value and confidence intervals adjusted for comparing a family of 7 estimates (confidence intervals corrected using the tukey method). | | | | | | | | | | | | | | | | | | | |

| Letter-Based Grouping - Animal group | | | |
| --- | --- | --- | --- |
| Animal group | | Letter | |
| C |  | a |  |
| OV |  | a |  |
| AL |  | a |  |
| AH |  | a |  |
| AL-X |  | a |  |
| AH-X |  | a |  |
| X |  | a |  |
|  | | | |
|  |  |  |  |
| --- | --- | --- | --- |
| *Note.*  If two or more means share the same grouping symbol, then we cannot show them to be different, but we also did not show them to be the same. | | | |

### Marginal Means

| Marginal Means - Animal group | | | | | | | | | |
| --- | --- | --- | --- | --- | --- | --- | --- | --- | --- |
|  | | | | 95% CI for Mean Difference | | | |  | |
| Animal group | | Marginal Mean | | Lower | | Upper | | SE | |
| C |  | 11.15302 |  | 10.49079 |  | 11.81525 |  | 0.23775 |  |
| OV |  | 12.03120 |  | 11.33314 |  | 12.72926 |  | 0.25061 |  |
| AL |  | 11.70239 |  | 11.00433 |  | 12.40045 |  | 0.25061 |  |
| AH |  | 11.40426 |  | 10.74203 |  | 12.06649 |  | 0.23775 |  |
| AL-X |  | 11.18702 |  | 10.52479 |  | 11.84925 |  | 0.23775 |  |
| AH-X |  | 11.91194 |  | 11.24971 |  | 12.57417 |  | 0.23775 |  |
| X |  | 11.18278 |  | 10.48472 |  | 11.88083 |  | 0.25061 |  |
|  | | | | | | | | | |
|  |  |  |  |  |  |  |  |  |  |
| --- | --- | --- | --- | --- | --- | --- | --- | --- | --- |
| *Note.*  Bonferroni CI adjustment | | | | | | | | | |

## Periosteal perimeter mm

| ANOVA - Periosteal perimeter (Ps.Pm) mm | | | | | | | | | | | | | | | | | | | | | | | | | |
| --- | --- | --- | --- | --- | --- | --- | --- | --- | --- | --- | --- | --- | --- | --- | --- | --- | --- | --- | --- | --- | --- | --- | --- | --- | --- |
|  | | | | | | | | | | | | | | | | 95% CI for η² | | | |  | | 95% CI for ω² | | | |
| Homogeneity Correction | | Cases | | Sum of Squares | | df | | Mean Square | | F | | p | | η² | | Lower | | Upper | | ω² | | Lower | | Upper | |
| None |  | Animal group |  | 5.66098 |  | 6.00000 |  | 0.94350 |  | 3.14793 |  | .00947 |  | 0.23942 |  | 0.02069 |  | 0.37180 |  | 0.16132 |  | 0.00000 |  | 0.27982 |  |
|  |  | Residuals |  | 17.98321 |  | 60.00000 |  | 0.29972 |  |  |  |  |  |  |  |  |  |  |  |  |  |  |  |  |  |
| Welch |  | Animal group |  | 5.66098 |  | 6.00000 |  | 0.94350 |  | 2.59984 |  | .04119 |  | 0.23942 |  | 0.02069 |  | 0.37180 |  | 0.16132 |  | 0.00000 |  | 0.27982 |  |
|  |  | Residuals |  | 17.98321 |  | 26.26423 |  | 0.68470 |  |  |  |  |  |  |  |  |  |  |  |  |  |  |  |  |  |
|  | | | | | | | | | | | | | | | | | | | | | | | | | |
|  |  |  |  |  |  |  |  |  |  |  |  |  |  |  |  |  |  |  |  |  |  |  |  |  |  |
| --- | --- | --- | --- | --- | --- | --- | --- | --- | --- | --- | --- | --- | --- | --- | --- | --- | --- | --- | --- | --- | --- | --- | --- | --- | --- |
| *Note.*  Type III Sum of Squares | | | | | | | | | | | | | | | | | | | | | | | | | |

### Descriptives

| Descriptives - Periosteal perimeter (Ps.Pm) mm | | | | | | | | | | | |
| --- | --- | --- | --- | --- | --- | --- | --- | --- | --- | --- | --- |
| Animal group | | N | | Mean | | SD | | SE | | Coefficient of variation | |
| C |  | 10 |  | 17.10995 |  | 0.60087 |  | 0.19001 |  | 0.03512 |  |
| OV |  | 9 |  | 16.51848 |  | 0.81231 |  | 0.27077 |  | 0.04918 |  |
| AL |  | 9 |  | 16.96918 |  | 0.43804 |  | 0.14601 |  | 0.02581 |  |
| AH |  | 10 |  | 17.30260 |  | 0.45318 |  | 0.14331 |  | 0.02619 |  |
| AL-X |  | 10 |  | 16.85386 |  | 0.33990 |  | 0.10749 |  | 0.02017 |  |
| AH-X |  | 10 |  | 17.03073 |  | 0.54821 |  | 0.17336 |  | 0.03219 |  |
| X |  | 9 |  | 17.52122 |  | 0.53932 |  | 0.17977 |  | 0.03078 |  |
|  | | | | | | | | | | | |

#### Bar plots

#####

### Assumption Checks

| Test for Equality of Variances (Levene's) | | | | | | | |
| --- | --- | --- | --- | --- | --- | --- | --- |
| F | | df1 | | df2 | | p | |
| 0.96239 |  | 6.00000 |  | 60.00000 |  | .45839 |  |
|  | | | | | | | |

### Post Hoc Tests

#### Standard (HSD)

| Post Hoc Comparisons - Animal group | | | | | | | | | | | | | | | | | | | |
| --- | --- | --- | --- | --- | --- | --- | --- | --- | --- | --- | --- | --- | --- | --- | --- | --- | --- | --- | --- |
|  | | | | | | 95% CI for Mean Difference | | | |  | | | | | | | | | |
|  | |  | | Mean Difference | | Lower | | Upper | | SE | | df | | t | | ptukey | | pbonf | |
| C |  | OV |  | 0.59147 |  | -0.17588 |  | 1.35882 |  | 0.25154 |  | 60 |  | 2.35137 |  | .23758 |  | .46214 |  |
|  |  | AL |  | 0.14077 |  | -0.62658 |  | 0.90812 |  | 0.25154 |  | 60 |  | 0.55963 |  | .99768 |  | 1.00000 |  |
|  |  | AH |  | -0.19265 |  | -0.93953 |  | 0.55423 |  | 0.24483 |  | 60 |  | -0.78686 |  | .98540 |  | 1.00000 |  |
|  |  | (AL-X) |  | 0.25609 |  | -0.49079 |  | 1.00297 |  | 0.24483 |  | 60 |  | 1.04597 |  | .94088 |  | 1.00000 |  |
|  |  | (AH-X) |  | 0.07922 |  | -0.66766 |  | 0.82610 |  | 0.24483 |  | 60 |  | 0.32357 |  | .99990 |  | 1.00000 |  |
|  |  | X |  | -0.41127 |  | -1.17862 |  | 0.35608 |  | 0.25154 |  | 60 |  | -1.63499 |  | .66064 |  | 1.00000 |  |
| OV |  | AL |  | -0.45070 |  | -1.23798 |  | 0.33658 |  | 0.25808 |  | 60 |  | -1.74637 |  | .58843 |  | 1.00000 |  |
|  |  | AH |  | -0.78412 |  | -1.55147 |  | -0.01677 |  | 0.25154 |  | 60 |  | -3.11724 |  | .04208 | \* | .05883 |  |
|  |  | (AL-X) |  | -0.33538 |  | -1.10273 |  | 0.43197 |  | 0.25154 |  | 60 |  | -1.33330 |  | .83354 |  | 1.00000 |  |
|  |  | (AH-X) |  | -0.51225 |  | -1.27960 |  | 0.25510 |  | 0.25154 |  | 60 |  | -2.03643 |  | .40335 |  | .96868 |  |
|  |  | X |  | -1.00274 |  | -1.79003 |  | -0.21546 |  | 0.25808 |  | 60 |  | -3.88542 |  | .00457 | \*\* | .00541 | \*\* |
| AL |  | AH |  | -0.33342 |  | -1.10077 |  | 0.43393 |  | 0.25154 |  | 60 |  | -1.32550 |  | .83729 |  | 1.00000 |  |
|  |  | (AL-X) |  | 0.11532 |  | -0.65203 |  | 0.88267 |  | 0.25154 |  | 60 |  | 0.45844 |  | .99925 |  | 1.00000 |  |
|  |  | (AH-X) |  | -0.06155 |  | -0.82890 |  | 0.70580 |  | 0.25154 |  | 60 |  | -0.24470 |  | .99998 |  | 1.00000 |  |
|  |  | X |  | -0.55204 |  | -1.33933 |  | 0.23524 |  | 0.25808 |  | 60 |  | -2.13906 |  | .34395 |  | .76671 |  |
| AH |  | (AL-X) |  | 0.44874 |  | -0.29814 |  | 1.19562 |  | 0.24483 |  | 60 |  | 1.83283 |  | .53184 |  | 1.00000 |  |
|  |  | (AH-X) |  | 0.27187 |  | -0.47501 |  | 1.01875 |  | 0.24483 |  | 60 |  | 1.11042 |  | .92249 |  | 1.00000 |  |
|  |  | X |  | -0.21862 |  | -0.98597 |  | 0.54873 |  | 0.25154 |  | 60 |  | -0.86912 |  | .97578 |  | 1.00000 |  |
| (AL-X) |  | (AH-X) |  | -0.17687 |  | -0.92375 |  | 0.57001 |  | 0.24483 |  | 60 |  | -0.72241 |  | .99067 |  | 1.00000 |  |
|  |  | X |  | -0.66736 |  | -1.43471 |  | 0.09999 |  | 0.25154 |  | 60 |  | -2.65307 |  | .12872 |  | .21405 |  |
| (AH-X) |  | X |  | -0.49049 |  | -1.25784 |  | 0.27686 |  | 0.25154 |  | 60 |  | -1.94993 |  | .45660 |  | 1.00000 |  |
|  | | | | | | | | | | | | | | | | | | | |
|  |  |  |  |  |  |  |  |  |  |  |  |  |  |  |  |  |  |  |  |
| --- | --- | --- | --- | --- | --- | --- | --- | --- | --- | --- | --- | --- | --- | --- | --- | --- | --- | --- | --- |
| \* p < .05, \*\* p < .01 | | | | | | | | | | | | | | | | | | | |
| *Note.*  P-value and confidence intervals adjusted for comparing a family of 7 estimates (confidence intervals corrected using the tukey method). | | | | | | | | | | | | | | | | | | | |

| Letter-Based Grouping - Animal group | | | |
| --- | --- | --- | --- |
| Animal group | | Letter | |
| C |  | ab |  |
| OV |  | a |  |
| AL |  | ab |  |
| AH |  | b |  |
| AL-X |  | ab |  |
| AH-X |  | ab |  |
| X |  | b |  |
|  | | | |
|  |  |  |  |
| --- | --- | --- | --- |
| *Note.*  If two or more means share the same grouping symbol, then we cannot show them to be different, but we also did not show them to be the same. | | | |

### Marginal Means

| Marginal Means - Animal group | | | | | | | | | |
| --- | --- | --- | --- | --- | --- | --- | --- | --- | --- |
|  | | | | 95% CI for Mean Difference | | | |  | |
| Animal group | | Marginal Mean | | Lower | | Upper | | SE | |
| C |  | 17.10995 |  | 16.62772 |  | 17.59218 |  | 0.17312 |  |
| OV |  | 16.51848 |  | 16.01016 |  | 17.02680 |  | 0.18249 |  |
| AL |  | 16.96918 |  | 16.46086 |  | 17.47750 |  | 0.18249 |  |
| AH |  | 17.30260 |  | 16.82037 |  | 17.78483 |  | 0.17312 |  |
| AL-X |  | 16.85386 |  | 16.37163 |  | 17.33609 |  | 0.17312 |  |
| AH-X |  | 17.03073 |  | 16.54850 |  | 17.51296 |  | 0.17312 |  |
| X |  | 17.52122 |  | 17.01290 |  | 18.02954 |  | 0.18249 |  |
|  | | | | | | | | | |
|  |  |  |  |  |  |  |  |  |  |
| --- | --- | --- | --- | --- | --- | --- | --- | --- | --- |
| *Note.*  Bonferroni CI adjustment | | | | | | | | | |

## Periosteal surface 3D mm2

| ANOVA - Periosteal surface (3D) (Ps.S3D) mm² | | | | | | | | | | | | | | | | | | | | | | | | | |
| --- | --- | --- | --- | --- | --- | --- | --- | --- | --- | --- | --- | --- | --- | --- | --- | --- | --- | --- | --- | --- | --- | --- | --- | --- | --- |
|  | | | | | | | | | | | | | | | | 95% CI for η² | | | |  | | 95% CI for ω² | | | |
| Homogeneity Correction | | Cases | | Sum of Squares | | df | | Mean Square | | F | | p | | η² | | Lower | | Upper | | ω² | | Lower | | Upper | |
| None |  | Animal group |  | 1350.07597 |  | 6.00000 |  | 225.01266 |  | 4.56528 |  | .00071 |  | 0.31344 |  | 0.08121 |  | 0.44859 |  | 0.24201 |  | 0.02254 |  | 0.37462 |  |
|  |  | Residuals |  | 2957.26959 |  | 60.00000 |  | 49.28783 |  |  |  |  |  |  |  |  |  |  |  |  |  |  |  |  |  |
| Welch |  | Animal group |  | 1350.07597 |  | 6.00000 |  | 225.01266 |  | 3.59494 |  | .00987 |  | 0.31344 |  | 0.08121 |  | 0.44859 |  | 0.24201 |  | 0.02254 |  | 0.37462 |  |
|  |  | Residuals |  | 2957.26959 |  | 26.16576 |  | 113.02058 |  |  |  |  |  |  |  |  |  |  |  |  |  |  |  |  |  |
|  | | | | | | | | | | | | | | | | | | | | | | | | | |
|  |  |  |  |  |  |  |  |  |  |  |  |  |  |  |  |  |  |  |  |  |  |  |  |  |  |
| --- | --- | --- | --- | --- | --- | --- | --- | --- | --- | --- | --- | --- | --- | --- | --- | --- | --- | --- | --- | --- | --- | --- | --- | --- | --- |
| *Note.*  Type III Sum of Squares | | | | | | | | | | | | | | | | | | | | | | | | | |

### Descriptives

| Descriptives - Periosteal surface (3D) (Ps.S3D) mm² | | | | | | | | | | | |
| --- | --- | --- | --- | --- | --- | --- | --- | --- | --- | --- | --- |
| Animal group | | N | | Mean | | SD | | SE | | Coefficient of variation | |
| C |  | 10 |  | 199.12591 |  | 7.36420 |  | 2.32877 |  | 0.03698 |  |
| OV |  | 9 |  | 191.92126 |  | 10.36424 |  | 3.45475 |  | 0.05400 |  |
| AL |  | 9 |  | 197.03729 |  | 6.57648 |  | 2.19216 |  | 0.03338 |  |
| AH |  | 10 |  | 201.34941 |  | 5.32723 |  | 1.68462 |  | 0.02646 |  |
| AL-X |  | 10 |  | 196.25881 |  | 4.24276 |  | 1.34168 |  | 0.02162 |  |
| AH-X |  | 10 |  | 197.84167 |  | 6.68205 |  | 2.11305 |  | 0.03377 |  |
| X |  | 9 |  | 207.96802 |  | 7.45464 |  | 2.48488 |  | 0.03585 |  |
|  | | | | | | | | | | | |

#### Bar plots

#####

### Assumption Checks

| Test for Equality of Variances (Levene's) | | | | | | | |
| --- | --- | --- | --- | --- | --- | --- | --- |
| F | | df1 | | df2 | | p | |
| 1.01270 |  | 6.00000 |  | 60.00000 |  | .42575 |  |
|  | | | | | | | |

### Post Hoc Tests

#### Standard (HSD)

| Post Hoc Comparisons - Animal group | | | | | | | | | | | | | | | | | | | |
| --- | --- | --- | --- | --- | --- | --- | --- | --- | --- | --- | --- | --- | --- | --- | --- | --- | --- | --- | --- |
|  | | | | | | 95% CI for Mean Difference | | | |  | | | | | | | | | |
|  | |  | | Mean Difference | | Lower | | Upper | | SE | | df | | t | | ptukey | | pbonf | |
| C |  | OV |  | 7.20465 |  | -2.63557 |  | 17.04488 |  | 3.22571 |  | 60 |  | 2.23351 |  | .29368 |  | .61437 |  |
|  |  | AL |  | 2.08862 |  | -7.75160 |  | 11.92884 |  | 3.22571 |  | 60 |  | 0.64749 |  | .99482 |  | 1.00000 |  |
|  |  | AH |  | -2.22350 |  | -11.80127 |  | 7.35427 |  | 3.13968 |  | 60 |  | -0.70819 |  | .99161 |  | 1.00000 |  |
|  |  | (AL-X) |  | 2.86710 |  | -6.71067 |  | 12.44487 |  | 3.13968 |  | 60 |  | 0.91318 |  | .96905 |  | 1.00000 |  |
|  |  | (AH-X) |  | 1.28424 |  | -8.29353 |  | 10.86201 |  | 3.13968 |  | 60 |  | 0.40904 |  | .99961 |  | 1.00000 |  |
|  |  | X |  | -8.84211 |  | -18.68233 |  | 0.99811 |  | 3.22571 |  | 60 |  | -2.74114 |  | .10573 |  | .16916 |  |
| OV |  | AL |  | -5.11603 |  | -15.21189 |  | 4.97982 |  | 3.30951 |  | 60 |  | -1.54586 |  | .71627 |  | 1.00000 |  |
|  |  | AH |  | -9.42815 |  | -19.26838 |  | 0.41207 |  | 3.22571 |  | 60 |  | -2.92281 |  | .06883 |  | .10260 |  |
|  |  | (AL-X) |  | -4.33755 |  | -14.17778 |  | 5.50267 |  | 3.22571 |  | 60 |  | -1.34468 |  | .82798 |  | 1.00000 |  |
|  |  | (AH-X) |  | -5.92041 |  | -15.76064 |  | 3.91981 |  | 3.22571 |  | 60 |  | -1.83538 |  | .53017 |  | 1.00000 |  |
|  |  | X |  | -16.04677 |  | -26.14262 |  | -5.95091 |  | 3.30951 |  | 60 |  | -4.84868 |  | .00018 | \*\*\* | .00019 | \*\*\* |
| AL |  | AH |  | -4.31212 |  | -14.15234 |  | 5.52810 |  | 3.22571 |  | 60 |  | -1.33680 |  | .83184 |  | 1.00000 |  |
|  |  | (AL-X) |  | 0.77848 |  | -9.06174 |  | 10.61870 |  | 3.22571 |  | 60 |  | 0.24134 |  | .99998 |  | 1.00000 |  |
|  |  | (AH-X) |  | -0.80438 |  | -10.64460 |  | 9.03584 |  | 3.22571 |  | 60 |  | -0.24937 |  | .99998 |  | 1.00000 |  |
|  |  | X |  | -10.93073 |  | -21.02659 |  | -0.83488 |  | 3.30951 |  | 60 |  | -3.30283 |  | .02557 | \* | .03395 | \* |
| AH |  | (AL-X) |  | 5.09060 |  | -4.48717 |  | 14.66837 |  | 3.13968 |  | 60 |  | 1.62138 |  | .66930 |  | 1.00000 |  |
|  |  | (AH-X) |  | 3.50774 |  | -6.07003 |  | 13.08551 |  | 3.13968 |  | 60 |  | 1.11723 |  | .92035 |  | 1.00000 |  |
|  |  | X |  | -6.61861 |  | -16.45883 |  | 3.22161 |  | 3.22571 |  | 60 |  | -2.05183 |  | .39415 |  | .93571 |  |
| (AL-X) |  | (AH-X) |  | -1.58286 |  | -11.16063 |  | 7.99491 |  | 3.13968 |  | 60 |  | -0.50415 |  | .99871 |  | 1.00000 |  |
|  |  | X |  | -11.70921 |  | -21.54943 |  | -1.86899 |  | 3.22571 |  | 60 |  | -3.62996 |  | .00999 | \*\* | .01234 | \* |
| (AH-X) |  | X |  | -10.12635 |  | -19.96657 |  | -0.28613 |  | 3.22571 |  | 60 |  | -3.13926 |  | .03972 | \* | .05517 |  |
|  | | | | | | | | | | | | | | | | | | | |
|  |  |  |  |  |  |  |  |  |  |  |  |  |  |  |  |  |  |  |  |
| --- | --- | --- | --- | --- | --- | --- | --- | --- | --- | --- | --- | --- | --- | --- | --- | --- | --- | --- | --- |
| \* p < .05, \*\* p < .01, \*\*\* p < .001 | | | | | | | | | | | | | | | | | | | |
| *Note.*  P-value and confidence intervals adjusted for comparing a family of 7 estimates (confidence intervals corrected using the tukey method). | | | | | | | | | | | | | | | | | | | |

| Letter-Based Grouping - Animal group | | | |
| --- | --- | --- | --- |
| Animal group | | Letter | |
| C |  | ab |  |
| OV |  | a |  |
| AL |  | a |  |
| AH |  | ab |  |
| AL-X |  | a |  |
| AH-X |  | a |  |
| X |  | b |  |
|  | | | |
|  |  |  |  |
| --- | --- | --- | --- |
| *Note.*  If two or more means share the same grouping symbol, then we cannot show them to be different, but we also did not show them to be the same. | | | |

### Marginal Means

| Marginal Means - Animal group | | | | | | | | | |
| --- | --- | --- | --- | --- | --- | --- | --- | --- | --- |
|  | | | | 95% CI for Mean Difference | | | |  | |
| Animal group | | Marginal Mean | | Lower | | Upper | | SE | |
| C |  | 199.12591 |  | 192.94193 |  | 205.30989 |  | 2.22009 |  |
| OV |  | 191.92126 |  | 185.40277 |  | 198.43975 |  | 2.34018 |  |
| AL |  | 197.03729 |  | 190.51880 |  | 203.55578 |  | 2.34018 |  |
| AH |  | 201.34941 |  | 195.16543 |  | 207.53339 |  | 2.22009 |  |
| AL-X |  | 196.25881 |  | 190.07483 |  | 202.44279 |  | 2.22009 |  |
| AH-X |  | 197.84167 |  | 191.65769 |  | 204.02565 |  | 2.22009 |  |
| X |  | 207.96802 |  | 201.44953 |  | 214.48651 |  | 2.34018 |  |
|  | | | | | | | | | |
|  |  |  |  |  |  |  |  |  |  |
| --- | --- | --- | --- | --- | --- | --- | --- | --- | --- |
| *Note.*  Bonferroni CI adjustment | | | | | | | | | |

## Total volume mm3

| ANOVA - Total volume (TV) mm³ | | | | | | | | | | | | | | | | | | | | | | | | | |
| --- | --- | --- | --- | --- | --- | --- | --- | --- | --- | --- | --- | --- | --- | --- | --- | --- | --- | --- | --- | --- | --- | --- | --- | --- | --- |
|  | | | | | | | | | | | | | | | | 95% CI for η² | | | |  | | 95% CI for ω² | | | |
| Homogeneity Correction | | Cases | | Sum of Squares | | df | | Mean Square | | F | | p | | η² | | Lower | | Upper | | ω² | | Lower | | Upper | |
| None |  | Animal group |  | 738.40675 |  | 6.00000 |  | 123.06779 |  | 1.79596 |  | .11519 |  | 0.15225 |  | 0.00000 |  | 0.26806 |  | 0.06654 |  | 0.00000 |  | 0.13439 |  |
|  |  | Residuals |  | 4111.47856 |  | 60.00000 |  | 68.52464 |  |  |  |  |  |  |  |  |  |  |  |  |  |  |  |  |  |
| Welch |  | Animal group |  | 738.40675 |  | 6.00000 |  | 123.06779 |  | 2.56958 |  | .04350 |  | 0.15225 |  | 0.00000 |  | 0.26806 |  | 0.06654 |  | 0.00000 |  | 0.13439 |  |
|  |  | Residuals |  | 4111.47856 |  | 25.88221 |  | 158.85345 |  |  |  |  |  |  |  |  |  |  |  |  |  |  |  |  |  |
|  | | | | | | | | | | | | | | | | | | | | | | | | | |
|  |  |  |  |  |  |  |  |  |  |  |  |  |  |  |  |  |  |  |  |  |  |  |  |  |  |
| --- | --- | --- | --- | --- | --- | --- | --- | --- | --- | --- | --- | --- | --- | --- | --- | --- | --- | --- | --- | --- | --- | --- | --- | --- | --- |
| *Note.*  Type III Sum of Squares | | | | | | | | | | | | | | | | | | | | | | | | | |

### Descriptives

| Descriptives - Total volume (TV) mm³ | | | | | | | | | | | |
| --- | --- | --- | --- | --- | --- | --- | --- | --- | --- | --- | --- |
| Animal group | | N | | Mean | | SD | | SE | | Coefficient of variation | |
| C |  | 10 |  | 165.35914 |  | 9.40053 |  | 2.97271 |  | 0.05685 |  |
| OV |  | 9 |  | 161.65399 |  | 12.26152 |  | 4.08717 |  | 0.07585 |  |
| AL |  | 9 |  | 163.54687 |  | 10.06296 |  | 3.35432 |  | 0.06153 |  |
| AH |  | 10 |  | 167.84323 |  | 6.06519 |  | 1.91798 |  | 0.03614 |  |
| AL-X |  | 10 |  | 164.09678 |  | 3.81820 |  | 1.20742 |  | 0.02327 |  |
| AH-X |  | 10 |  | 164.39746 |  | 7.86562 |  | 2.48733 |  | 0.04785 |  |
| X |  | 9 |  | 172.84671 |  | 5.96014 |  | 1.98671 |  | 0.03448 |  |
|  | | | | | | | | | | | |

#### Bar plots

#####

### Assumption Checks

| Test for Equality of Variances (Levene's) | | | | | | | |
| --- | --- | --- | --- | --- | --- | --- | --- |
| F | | df1 | | df2 | | p | |
| 2.49064 |  | 6.00000 |  | 60.00000 |  | .03225 |  |
|  | | | | | | | |

### Post Hoc Tests

#### Standard (HSD)

| Post Hoc Comparisons - Animal group | | | | | | | | | | | | | | | | | | | |
| --- | --- | --- | --- | --- | --- | --- | --- | --- | --- | --- | --- | --- | --- | --- | --- | --- | --- | --- | --- |
|  | | | | | | 95% CI for Mean Difference | | | |  | | | | | | | | | |
|  | |  | | Mean Difference | | Lower | | Upper | | SE | | df | | t | | ptukey | | pbonf | |
| C |  | OV |  | 3.70515 |  | -7.89753 |  | 15.30783 |  | 3.80346 |  | 60 |  | 0.97415 |  | .95766 |  | 1.00000 |  |
|  |  | AL |  | 1.81227 |  | -9.79041 |  | 13.41496 |  | 3.80346 |  | 60 |  | 0.47648 |  | .99906 |  | 1.00000 |  |
|  |  | AH |  | -2.48409 |  | -13.77731 |  | 8.80913 |  | 3.70202 |  | 60 |  | -0.67101 |  | .99371 |  | 1.00000 |  |
|  |  | (AL-X) |  | 1.26236 |  | -10.03086 |  | 12.55558 |  | 3.70202 |  | 60 |  | 0.34099 |  | .99986 |  | 1.00000 |  |
|  |  | (AH-X) |  | 0.96168 |  | -10.33154 |  | 12.25490 |  | 3.70202 |  | 60 |  | 0.25977 |  | .99997 |  | 1.00000 |  |
|  |  | X |  | -7.48757 |  | -19.09025 |  | 4.11511 |  | 3.80346 |  | 60 |  | -1.96862 |  | .44488 |  | 1.00000 |  |
| OV |  | AL |  | -1.89288 |  | -13.79698 |  | 10.01122 |  | 3.90227 |  | 60 |  | -0.48507 |  | .99896 |  | 1.00000 |  |
|  |  | AH |  | -6.18924 |  | -17.79192 |  | 5.41344 |  | 3.80346 |  | 60 |  | -1.62727 |  | .66556 |  | 1.00000 |  |
|  |  | (AL-X) |  | -2.44279 |  | -14.04547 |  | 9.15989 |  | 3.80346 |  | 60 |  | -0.64225 |  | .99504 |  | 1.00000 |  |
|  |  | (AH-X) |  | -2.74347 |  | -14.34615 |  | 8.85921 |  | 3.80346 |  | 60 |  | -0.72131 |  | .99075 |  | 1.00000 |  |
|  |  | X |  | -11.19272 |  | -23.09682 |  | 0.71138 |  | 3.90227 |  | 60 |  | -2.86826 |  | .07855 |  | .11946 |  |
| AL |  | AH |  | -4.29636 |  | -15.89905 |  | 7.30632 |  | 3.80346 |  | 60 |  | -1.12959 |  | .91638 |  | 1.00000 |  |
|  |  | (AL-X) |  | -0.54991 |  | -12.15260 |  | 11.05277 |  | 3.80346 |  | 60 |  | -0.14458 |  | 1.00000 |  | 1.00000 |  |
|  |  | (AH-X) |  | -0.85059 |  | -12.45328 |  | 10.75209 |  | 3.80346 |  | 60 |  | -0.22364 |  | .99999 |  | 1.00000 |  |
|  |  | X |  | -9.29984 |  | -21.20395 |  | 2.60426 |  | 3.90227 |  | 60 |  | -2.38319 |  | .22376 |  | .42727 |  |
| AH |  | (AL-X) |  | 3.74645 |  | -7.54677 |  | 15.03967 |  | 3.70202 |  | 60 |  | 1.01200 |  | .94930 |  | 1.00000 |  |
|  |  | (AH-X) |  | 3.44577 |  | -7.84745 |  | 14.73899 |  | 3.70202 |  | 60 |  | 0.93078 |  | .96602 |  | 1.00000 |  |
|  |  | X |  | -5.00348 |  | -16.60616 |  | 6.59920 |  | 3.80346 |  | 60 |  | -1.31551 |  | .84205 |  | 1.00000 |  |
| (AL-X) |  | (AH-X) |  | -0.30068 |  | -11.59390 |  | 10.99254 |  | 3.70202 |  | 60 |  | -0.08122 |  | 1.00000 |  | 1.00000 |  |
|  |  | X |  | -8.74993 |  | -20.35261 |  | 2.85275 |  | 3.80346 |  | 60 |  | -2.30052 |  | .26085 |  | .52313 |  |
| (AH-X) |  | X |  | -8.44925 |  | -20.05193 |  | 3.15343 |  | 3.80346 |  | 60 |  | -2.22146 |  | .29983 |  | .63218 |  |
|  | | | | | | | | | | | | | | | | | | | |
|  |  |  |  |  |  |  |  |  |  |  |  |  |  |  |  |  |  |  |  |
| --- | --- | --- | --- | --- | --- | --- | --- | --- | --- | --- | --- | --- | --- | --- | --- | --- | --- | --- | --- |
| *Note.*  P-value and confidence intervals adjusted for comparing a family of 7 estimates (confidence intervals corrected using the tukey method). | | | | | | | | | | | | | | | | | | | |

| Letter-Based Grouping - Animal group | | | |
| --- | --- | --- | --- |
| Animal group | | Letter | |
| C |  | a |  |
| OV |  | a |  |
| AL |  | a |  |
| AH |  | a |  |
| AL-X |  | a |  |
| AH-X |  | a |  |
| X |  | a |  |
|  | | | |
|  |  |  |  |
| --- | --- | --- | --- |
| *Note.*  If two or more means share the same grouping symbol, then we cannot show them to be different, but we also did not show them to be the same. | | | |

### Marginal Means

| Marginal Means - Animal group | | | | | | | | | |
| --- | --- | --- | --- | --- | --- | --- | --- | --- | --- |
|  | | | | 95% CI for Mean Difference | | | |  | |
| Animal group | | Marginal Mean | | Lower | | Upper | | SE | |
| C |  | 165.35914 |  | 158.06756 |  | 172.65072 |  | 2.61772 |  |
| OV |  | 161.65399 |  | 153.96799 |  | 169.33999 |  | 2.75932 |  |
| AL |  | 163.54687 |  | 155.86086 |  | 171.23287 |  | 2.75932 |  |
| AH |  | 167.84323 |  | 160.55165 |  | 175.13481 |  | 2.61772 |  |
| AL-X |  | 164.09678 |  | 156.80520 |  | 171.38836 |  | 2.61772 |  |
| AH-X |  | 164.39746 |  | 157.10588 |  | 171.68904 |  | 2.61772 |  |
| X |  | 172.84671 |  | 165.16071 |  | 180.53271 |  | 2.75932 |  |
|  | | | | | | | | | |
|  |  |  |  |  |  |  |  |  |  |
| --- | --- | --- | --- | --- | --- | --- | --- | --- | --- |
| *Note.*  Bonferroni CI adjustment | | | | | | | | | |

## Average trabecular separation mm

| ANOVA - Average trabecular separation (Tb.Sp) mm | | | | | | | | | | | | | | | | | | | | | | | | | |
| --- | --- | --- | --- | --- | --- | --- | --- | --- | --- | --- | --- | --- | --- | --- | --- | --- | --- | --- | --- | --- | --- | --- | --- | --- | --- |
|  | | | | | | | | | | | | | | | | 95% CI for η² | | | |  | | 95% CI for ω² | | | |
| Homogeneity Correction | | Cases | | Sum of Squares | | df | | Mean Square | | F | | p | | η² | | Lower | | Upper | | ω² | | Lower | | Upper | |
| None |  | Animal group |  | 0.02960 |  | 6.00000 |  | 0.00493 |  | 0.87673 |  | .51748 |  | 0.08061 |  | 0.00000 |  | 0.16062 |  | 0.00000 |  | 0.00000 |  | 0.00000 |  |
|  |  | Residuals |  | 0.33760 |  | 60.00000 |  | 0.00563 |  |  |  |  |  |  |  |  |  |  |  |  |  |  |  |  |  |
| Welch |  | Animal group |  | 0.02960 |  | 6.00000 |  | 0.00493 |  | 1.40102 |  | .25170 |  | 0.08061 |  | 0.00000 |  | 0.16062 |  | 0.00000 |  | 0.00000 |  | 0.00000 |  |
|  |  | Residuals |  | 0.33760 |  | 26.06164 |  | 0.01295 |  |  |  |  |  |  |  |  |  |  |  |  |  |  |  |  |  |
|  | | | | | | | | | | | | | | | | | | | | | | | | | |
|  |  |  |  |  |  |  |  |  |  |  |  |  |  |  |  |  |  |  |  |  |  |  |  |  |  |
| --- | --- | --- | --- | --- | --- | --- | --- | --- | --- | --- | --- | --- | --- | --- | --- | --- | --- | --- | --- | --- | --- | --- | --- | --- | --- |
| *Note.*  Type III Sum of Squares | | | | | | | | | | | | | | | | | | | | | | | | | |

### Descriptives

| Descriptives - Average trabecular separation (Tb.Sp) mm | | | | | | | | | | | |
| --- | --- | --- | --- | --- | --- | --- | --- | --- | --- | --- | --- |
| Animal group | | N | | Mean | | SD | | SE | | Coefficient of variation | |
| C |  | 10 |  | 0.34453 |  | 0.09204 |  | 0.02911 |  | 0.26715 |  |
| OV |  | 9 |  | 0.33862 |  | 0.08913 |  | 0.02971 |  | 0.26323 |  |
| AL |  | 9 |  | 0.33093 |  | 0.10188 |  | 0.03396 |  | 0.30787 |  |
| AH |  | 10 |  | 0.28302 |  | 0.04035 |  | 0.01276 |  | 0.14258 |  |
| AL-X |  | 10 |  | 0.32710 |  | 0.07081 |  | 0.02239 |  | 0.21649 |  |
| AH-X |  | 10 |  | 0.32978 |  | 0.05403 |  | 0.01709 |  | 0.16385 |  |
| X |  | 9 |  | 0.29781 |  | 0.05988 |  | 0.01996 |  | 0.20108 |  |
|  | | | | | | | | | | | |

#### Bar plots

#####

### Assumption Checks

| Test for Equality of Variances (Levene's) | | | | | | | |
| --- | --- | --- | --- | --- | --- | --- | --- |
| F | | df1 | | df2 | | p | |
| 0.72246 |  | 6.00000 |  | 60.00000 |  | .63312 |  |
|  | | | | | | | |

### Marginal Means

| Marginal Means - Animal group | | | | | | | | | |
| --- | --- | --- | --- | --- | --- | --- | --- | --- | --- |
|  | | | | 95% CI for Mean Difference | | | |  | |
| Animal group | | Marginal Mean | | Lower | | Upper | | SE | |
| C |  | 0.34453 |  | 0.27846 |  | 0.41060 |  | 0.02372 |  |
| OV |  | 0.33862 |  | 0.26898 |  | 0.40827 |  | 0.02500 |  |
| AL |  | 0.33093 |  | 0.26129 |  | 0.40058 |  | 0.02500 |  |
| AH |  | 0.28302 |  | 0.21695 |  | 0.34909 |  | 0.02372 |  |
| AL-X |  | 0.32710 |  | 0.26103 |  | 0.39317 |  | 0.02372 |  |
| AH-X |  | 0.32978 |  | 0.26371 |  | 0.39585 |  | 0.02372 |  |
| X |  | 0.29781 |  | 0.22816 |  | 0.36746 |  | 0.02500 |  |
|  | | | | | | | | | |
|  |  |  |  |  |  |  |  |  |  |
| --- | --- | --- | --- | --- | --- | --- | --- | --- | --- |
| *Note.*  Bonferroni CI adjustment | | | | | | | | | |

## Average trabecular thickness mm

| ANOVA - Average trabecular thickness (Tb.Th) mm | | | | | | | | | | | | | | | | | | | | | | | | | |
| --- | --- | --- | --- | --- | --- | --- | --- | --- | --- | --- | --- | --- | --- | --- | --- | --- | --- | --- | --- | --- | --- | --- | --- | --- | --- |
|  | | | | | | | | | | | | | | | | 95% CI for η² | | | |  | | 95% CI for ω² | | | |
| Homogeneity Correction | | Cases | | Sum of Squares | | df | | Mean Square | | F | | p | | η² | | Lower | | Upper | | ω² | | Lower | | Upper | |
| None |  | Animal group |  | 0.00439 |  | 6.00000 |  | 0.00073 |  | 52.49388 |  | < .00001 |  | 0.83998 |  | 0.76111 |  | 0.88349 |  | 0.82179 |  | 0.73402 |  | 0.87002 |  |
|  |  | Residuals |  | 0.00084 |  | 60.00000 |  | 0.00001 |  |  |  |  |  |  |  |  |  |  |  |  |  |  |  |  |  |
| Welch |  | Animal group |  | 0.00439 |  | 6.00000 |  | 0.00073 |  | 51.20497 |  | < .00001 |  | 0.83998 |  | 0.76111 |  | 0.88349 |  | 0.82179 |  | 0.73402 |  | 0.87002 |  |
|  |  | Residuals |  | 0.00084 |  | 26.49865 |  | 0.00003 |  |  |  |  |  |  |  |  |  |  |  |  |  |  |  |  |  |
|  | | | | | | | | | | | | | | | | | | | | | | | | | |
|  |  |  |  |  |  |  |  |  |  |  |  |  |  |  |  |  |  |  |  |  |  |  |  |  |  |
| --- | --- | --- | --- | --- | --- | --- | --- | --- | --- | --- | --- | --- | --- | --- | --- | --- | --- | --- | --- | --- | --- | --- | --- | --- | --- |
| *Note.*  Type III Sum of Squares | | | | | | | | | | | | | | | | | | | | | | | | | |

### Descriptives

| Descriptives - Average trabecular thickness (Tb.Th) mm | | | | | | | | | | | |
| --- | --- | --- | --- | --- | --- | --- | --- | --- | --- | --- | --- |
| Animal group | | N | | Mean | | SD | | SE | | Coefficient of variation | |
| C |  | 10 |  | 0.12827 |  | 0.00438 |  | 0.00138 |  | 0.03413 |  |
| OV |  | 9 |  | 0.11014 |  | 0.00398 |  | 0.00133 |  | 0.03617 |  |
| AL |  | 9 |  | 0.11890 |  | 0.00408 |  | 0.00136 |  | 0.03434 |  |
| AH |  | 10 |  | 0.13184 |  | 0.00415 |  | 0.00131 |  | 0.03146 |  |
| AL-X |  | 10 |  | 0.12856 |  | 0.00319 |  | 0.00101 |  | 0.02481 |  |
| AH-X |  | 10 |  | 0.11519 |  | 0.00328 |  | 0.00104 |  | 0.02851 |  |
| X |  | 9 |  | 0.13251 |  | 0.00274 |  | 0.00091 |  | 0.02070 |  |
|  | | | | | | | | | | | |

#### Bar plots

#####

### Assumption Checks

| Test for Equality of Variances (Levene's) | | | | | | | |
| --- | --- | --- | --- | --- | --- | --- | --- |
| F | | df1 | | df2 | | p | |
| 0.74016 |  | 6.00000 |  | 60.00000 |  | .61940 |  |
|  | | | | | | | |

### Post Hoc Tests

#### Standard (HSD)

| Post Hoc Comparisons - Animal group | | | | | | | | | | | | | | | | | | | |
| --- | --- | --- | --- | --- | --- | --- | --- | --- | --- | --- | --- | --- | --- | --- | --- | --- | --- | --- | --- |
|  | | | | | | 95% CI for Mean Difference | | | |  | | | | | | | | | |
|  | |  | | Mean Difference | | Lower | | Upper | | SE | | df | | t | | ptukey | | pbonf | |
| C |  | OV |  | 0.01813 |  | 0.01289 |  | 0.02336 |  | 0.00172 |  | 60 |  | 10.56491 |  | < .00001 | \*\*\* | < .00001 | \*\*\* |
|  |  | AL |  | 0.00937 |  | 0.00414 |  | 0.01460 |  | 0.00172 |  | 60 |  | 5.46153 |  | .00002 | \*\*\* | .00002 | \*\*\* |
|  |  | AH |  | -0.00357 |  | -0.00866 |  | 0.00152 |  | 0.00167 |  | 60 |  | -2.13788 |  | .34460 |  | .76880 |  |
|  |  | (AL-X) |  | -0.00029 |  | -0.00538 |  | 0.00480 |  | 0.00167 |  | 60 |  | -0.17367 |  | 1.00000 |  | 1.00000 |  |
|  |  | (AH-X) |  | 0.01308 |  | 0.00799 |  | 0.01817 |  | 0.00167 |  | 60 |  | 7.83290 |  | < .00001 | \*\*\* | < .00001 | \*\*\* |
|  |  | X |  | -0.00424 |  | -0.00947 |  | 0.00099 |  | 0.00172 |  | 60 |  | -2.47203 |  | .18813 |  | .34205 |  |
| OV |  | AL |  | -0.00876 |  | -0.01413 |  | -0.00339 |  | 0.00176 |  | 60 |  | -4.97416 |  | .00011 | \*\*\* | .00012 | \*\*\* |
|  |  | AH |  | -0.02170 |  | -0.02693 |  | -0.01646 |  | 0.00172 |  | 60 |  | -12.64577 |  | < .00001 | \*\*\* | < .00001 | \*\*\* |
|  |  | (AL-X) |  | -0.01842 |  | -0.02365 |  | -0.01318 |  | 0.00172 |  | 60 |  | -10.73394 |  | < .00001 | \*\*\* | < .00001 | \*\*\* |
|  |  | (AH-X) |  | -0.00505 |  | -0.01028 |  | 0.00019 |  | 0.00172 |  | 60 |  | -2.94092 |  | .06584 |  | .09751 |  |
|  |  | X |  | -0.02237 |  | -0.02774 |  | -0.01700 |  | 0.00176 |  | 60 |  | -12.70684 |  | < .00001 | \*\*\* | < .00001 | \*\*\* |
| AL |  | AH |  | -0.01294 |  | -0.01817 |  | -0.00771 |  | 0.00172 |  | 60 |  | -7.54238 |  | < .00001 | \*\*\* | < .00001 | \*\*\* |
|  |  | (AL-X) |  | -0.00966 |  | -0.01489 |  | -0.00443 |  | 0.00172 |  | 60 |  | -5.63056 |  | .00001 | \*\*\* | .00001 | \*\*\* |
|  |  | (AH-X) |  | 0.00371 |  | -0.00152 |  | 0.00894 |  | 0.00172 |  | 60 |  | 2.16246 |  | .33107 |  | .72617 |  |
|  |  | X |  | -0.01361 |  | -0.01898 |  | -0.00824 |  | 0.00176 |  | 60 |  | -7.73267 |  | < .00001 | \*\*\* | < .00001 | \*\*\* |
| AH |  | (AL-X) |  | 0.00328 |  | -0.00181 |  | 0.00837 |  | 0.00167 |  | 60 |  | 1.96421 |  | .44764 |  | 1.00000 |  |
|  |  | (AH-X) |  | 0.01665 |  | 0.01156 |  | 0.02174 |  | 0.00167 |  | 60 |  | 9.97078 |  | < .00001 | \*\*\* | < .00001 | \*\*\* |
|  |  | X |  | -0.00067 |  | -0.00590 |  | 0.00456 |  | 0.00172 |  | 60 |  | -0.39117 |  | .99970 |  | 1.00000 |  |
| (AL-X) |  | (AH-X) |  | 0.01337 |  | 0.00828 |  | 0.01846 |  | 0.00167 |  | 60 |  | 8.00657 |  | < .00001 | \*\*\* | < .00001 | \*\*\* |
|  |  | X |  | -0.00395 |  | -0.00918 |  | 0.00128 |  | 0.00172 |  | 60 |  | -2.30300 |  | .25968 |  | .52000 |  |
| (AH-X) |  | X |  | -0.01732 |  | -0.02255 |  | -0.01209 |  | 0.00172 |  | 60 |  | -10.09602 |  | < .00001 | \*\*\* | < .00001 | \*\*\* |
|  | | | | | | | | | | | | | | | | | | | |
|  |  |  |  |  |  |  |  |  |  |  |  |  |  |  |  |  |  |  |  |
| --- | --- | --- | --- | --- | --- | --- | --- | --- | --- | --- | --- | --- | --- | --- | --- | --- | --- | --- | --- |
| \*\*\* p < .001 | | | | | | | | | | | | | | | | | | | |
| *Note.*  P-value and confidence intervals adjusted for comparing a family of 7 estimates (confidence intervals corrected using the tukey method). | | | | | | | | | | | | | | | | | | | |

| Letter-Based Grouping - Animal group | | | |
| --- | --- | --- | --- |
| Animal group | | Letter | |
| C |  | c |  |
| OV |  | a |  |
| AL |  | b |  |
| AH |  | c |  |
| AL-X |  | c |  |
| AH-X |  | ab |  |
| X |  | c |  |
|  | | | |
|  |  |  |  |
| --- | --- | --- | --- |
| *Note.*  If two or more means share the same grouping symbol, then we cannot show them to be different, but we also did not show them to be the same. | | | |

### Marginal Means

| Marginal Means - Animal group | | | | | | | | | |
| --- | --- | --- | --- | --- | --- | --- | --- | --- | --- |
|  | | | | 95% CI for Mean Difference | | | |  | |
| Animal group | | Marginal Mean | | Lower | | Upper | | SE | |
| C |  | 0.12827 |  | 0.12498 |  | 0.13156 |  | 0.00118 |  |
| OV |  | 0.11014 |  | 0.10668 |  | 0.11361 |  | 0.00124 |  |
| AL |  | 0.11890 |  | 0.11543 |  | 0.12237 |  | 0.00124 |  |
| AH |  | 0.13184 |  | 0.12855 |  | 0.13513 |  | 0.00118 |  |
| AL-X |  | 0.12856 |  | 0.12527 |  | 0.13185 |  | 0.00118 |  |
| AH-X |  | 0.11519 |  | 0.11190 |  | 0.11848 |  | 0.00118 |  |
| X |  | 0.13251 |  | 0.12904 |  | 0.13598 |  | 0.00124 |  |
|  | | | | | | | | | |
|  |  |  |  |  |  |  |  |  |  |
| --- | --- | --- | --- | --- | --- | --- | --- | --- | --- |
| *Note.*  Bonferroni CI adjustment | | | | | | | | | |

## Average total marrow area

| ANOVA - Average total (cortical + marrow) area (Tt.Ar) mm² | | | | | | | | | | | | | | | | | | | | | | | | | |
| --- | --- | --- | --- | --- | --- | --- | --- | --- | --- | --- | --- | --- | --- | --- | --- | --- | --- | --- | --- | --- | --- | --- | --- | --- | --- |
|  | | | | | | | | | | | | | | | | 95% CI for η² | | | |  | | 95% CI for ω² | | | |
| Homogeneity Correction | | Cases | | Sum of Squares | | df | | Mean Square | | F | | p | | η² | | Lower | | Upper | | ω² | | Lower | | Upper | |
| None |  | Animal group |  | 7.11968 |  | 6.00000 |  | 1.18661 |  | 1.60939 |  | .16025 |  | 0.13863 |  | 0.00000 |  | 0.24980 |  | 0.05175 |  | 0.00000 |  | 0.10303 |  |
|  |  | Residuals |  | 44.23848 |  | 60.00000 |  | 0.73731 |  |  |  |  |  |  |  |  |  |  |  |  |  |  |  |  |  |
| Welch |  | Animal group |  | 7.11968 |  | 6.00000 |  | 1.18661 |  | 1.65335 |  | .17314 |  | 0.13863 |  | 0.00000 |  | 0.24980 |  | 0.05175 |  | 0.00000 |  | 0.10303 |  |
|  |  | Residuals |  | 44.23848 |  | 25.68166 |  | 1.72257 |  |  |  |  |  |  |  |  |  |  |  |  |  |  |  |  |  |
|  | | | | | | | | | | | | | | | | | | | | | | | | | |
|  |  |  |  |  |  |  |  |  |  |  |  |  |  |  |  |  |  |  |  |  |  |  |  |  |  |
| --- | --- | --- | --- | --- | --- | --- | --- | --- | --- | --- | --- | --- | --- | --- | --- | --- | --- | --- | --- | --- | --- | --- | --- | --- | --- |
| *Note.*  Type III Sum of Squares | | | | | | | | | | | | | | | | | | | | | | | | | |

### Descriptives

| Descriptives - Average total (cortical + marrow) area (Tt.Ar) mm² | | | | | | | | | | | |
| --- | --- | --- | --- | --- | --- | --- | --- | --- | --- | --- | --- |
| Animal group | | N | | Mean | | SD | | SE | | Coefficient of variation | |
| C |  | 10 |  | 16.56753 |  | 0.94427 |  | 0.29861 |  | 0.05700 |  |
| OV |  | 9 |  | 16.19408 |  | 1.22639 |  | 0.40880 |  | 0.07573 |  |
| AL |  | 9 |  | 16.38747 |  | 1.00775 |  | 0.33592 |  | 0.06150 |  |
| AH |  | 10 |  | 16.86334 |  | 0.64229 |  | 0.20311 |  | 0.03809 |  |
| AL-X |  | 10 |  | 16.42928 |  | 0.37637 |  | 0.11902 |  | 0.02291 |  |
| AH-X |  | 10 |  | 16.47091 |  | 0.78482 |  | 0.24818 |  | 0.04765 |  |
| X |  | 9 |  | 17.27237 |  | 0.83109 |  | 0.27703 |  | 0.04812 |  |
|  | | | | | | | | | | | |

#### Bar plots

#####

### Assumption Checks

| Test for Equality of Variances (Levene's) | | | | | | | |
| --- | --- | --- | --- | --- | --- | --- | --- |
| F | | df1 | | df2 | | p | |
| 2.03323 |  | 6.00000 |  | 60.00000 |  | .07501 |  |
|  | | | | | | | |

### Marginal Means

| Marginal Means - Animal group | | | | | | | | | |
| --- | --- | --- | --- | --- | --- | --- | --- | --- | --- |
|  | | | | 95% CI for Mean Difference | | | |  | |
| Animal group | | Marginal Mean | | Lower | | Upper | | SE | |
| C |  | 16.56753 |  | 15.81118 |  | 17.32388 |  | 0.27153 |  |
| OV |  | 16.19408 |  | 15.39681 |  | 16.99134 |  | 0.28622 |  |
| AL |  | 16.38747 |  | 15.59020 |  | 17.18473 |  | 0.28622 |  |
| AH |  | 16.86334 |  | 16.10699 |  | 17.61969 |  | 0.27153 |  |
| AL-X |  | 16.42928 |  | 15.67293 |  | 17.18563 |  | 0.27153 |  |
| AH-X |  | 16.47091 |  | 15.71456 |  | 17.22726 |  | 0.27153 |  |
| X |  | 17.27237 |  | 16.47510 |  | 18.06963 |  | 0.28622 |  |
|  | | | | | | | | | |
|  |  |  |  |  |  |  |  |  |  |
| --- | --- | --- | --- | --- | --- | --- | --- | --- | --- |
| *Note.*  Bonferroni CI adjustment | | | | | | | | | |

## Support Vector Machine Regression

| Model Summary: Support Vector Machine Regression | | | | | | | | | | | | | |
| --- | --- | --- | --- | --- | --- | --- | --- | --- | --- | --- | --- | --- | --- |
| Violation cost | | Support Vectors | | n(Train) | | n(Validation) | | n(Test) | | Validation MSE | | Test MSE | |
| 0.80000 |  | 25 |  | 25 |  | 18 |  | 10 |  | 136.42457 |  | 98.23251 |  |
|  | | | | | | | | | | | | | |

### Data Split

| Model Performance Metrics | | | |
| --- | --- | --- | --- |
|  | | Value | |
| MSE |  | 98.233 |  |
| MSE(scaled) |  | 0.806 |  |
| RMSE |  | 9.911 |  |
| MAE / MAD |  | 8.727 |  |
| MAPE |  | 9.46% |  |
| R² |  | 0.305 |  |
|  | | | |

| Feature Importance Metrics | | | |
| --- | --- | --- | --- |
|  | | Mean dropout loss | |
| Cre umol/L |  | 9.49379 |  |
| Animal group |  | 9.46491 |  |
| TP g/L |  | 9.38205 |  |
| P mmol/L |  | 9.09580 |  |
| MCV fl |  | 9.01873 |  |
| ALP U/L |  | 8.98731 |  |
| TBIL umol/L |  | 8.86921 |  |
| ALB g/l |  | 8.51466 |  |
| AMY U/L |  | 8.47297 |  |
| ALT U/L |  | 8.45255 |  |
| K mmol/L |  | 8.38414 |  |
| Na mmol/L |  | 8.35314 |  |
| RBC 10^12/l |  | 8.34358 |  |
| HGB g/l |  | 8.32740 |  |
| Ca mmol/L |  | 8.30589 |  |
|  | | | |
|  |  |  |  |
| --- | --- | --- | --- |
| *Note.*  Mean dropout loss (defined as root mean squared error (RMSE)) is based on 50 permutations. | | | |

### Predictive Performance Plot

### Mean Squared Error Plot
