# Supplementary material for: The Early Effect of Alendronate, Hop Extract and Their Combination on Bone Structural Properties in a Rat Model of Osteoporosis
Source: Med Sci (Basel). 2026 May 5;14(2):239. doi: 10.3390/medsci14020239 (PMC13214732; doi:10.3390/medsci14020239)
Supplement: Supplementary file 1 [file medsci-14-00239-s001.zip › Table S2.html]

JASP 


# Results

## Descriptive Statistics

| Descriptive Statistics | | | | | | | | | | | | | | | | | | | | | | | | | |
| --- | --- | --- | --- | --- | --- | --- | --- | --- | --- | --- | --- | --- | --- | --- | --- | --- | --- | --- | --- | --- | --- | --- | --- | --- | --- |
|  | |  | | Valid | | Missing | | Median | | Mean | | Std. Deviation | | Shapiro-Wilk | | P-value of Shapiro-Wilk | | Minimum | | Maximum | | 25th percentile | | 75th percentile | |
| ALB g/l |  | C |  | 10 |  | 0 |  | 53.000 |  | 52.600 |  | 3.950 |  | 0.959 |  | .772 |  | 45.00 |  | 58.00 |  | 51.25 |  | 54.75 |  |
| ALB g/l |  | OV |  | 7 |  | 1 |  | 46.000 |  | 46.286 |  | 4.499 |  | 0.896 |  | .305 |  | 40.00 |  | 51.00 |  | 43.00 |  | 50.50 |  |
| ALB g/l |  | AL |  | 8 |  | 0 |  | 48.000 |  | 46.750 |  | 4.496 |  | 0.829 |  | .058 |  | 37.00 |  | 51.00 |  | 46.25 |  | 49.25 |  |
| ALB g/l |  | AH |  | 9 |  | 0 |  | 49.000 |  | 50.111 |  | 5.061 |  | 0.838 |  | .055 |  | 44.00 |  | 62.00 |  | 47.00 |  | 51.00 |  |
| ALB g/l |  | AL-X |  | 10 |  | 0 |  | 48.500 |  | 49.400 |  | 5.168 |  | 0.857 |  | .071 |  | 43.00 |  | 62.00 |  | 46.25 |  | 50.75 |  |
| ALB g/l |  | AH-X |  | 10 |  | 0 |  | 47.000 |  | 47.100 |  | 2.514 |  | 0.952 |  | .692 |  | 42.00 |  | 51.00 |  | 46.25 |  | 48.00 |  |
| ALB g/l |  | X |  | 6 |  | 2 |  | 47.500 |  | 48.000 |  | 2.366 |  | 0.947 |  | .719 |  | 45.00 |  | 52.00 |  | 47.00 |  | 48.75 |  |
| ALP U/L |  | C |  | 10 |  | 0 |  | 58.000 |  | 66.100 |  | 29.339 |  | 0.853 |  | .064 |  | 38.00 |  | 132.0 |  | 48.50 |  | 71.25 |  |
| ALP U/L |  | OV |  | 7 |  | 1 |  | 87.000 |  | 97.571 |  | 40.476 |  | 0.910 |  | .399 |  | 54.00 |  | 160.0 |  | 67.00 |  | 124.0 |  |
| ALP U/L |  | AL |  | 8 |  | 0 |  | 65.500 |  | 68.125 |  | 19.423 |  | 0.737 |  | .006 |  | 53.00 |  | 113.0 |  | 54.75 |  | 68.75 |  |
| ALP U/L |  | AH |  | 5 |  | 4 |  | 89.000 |  | 92.000 |  | 33.985 |  | 0.868 |  | .257 |  | 63.00 |  | 147.0 |  | 65.00 |  | 96.00 |  |
| ALP U/L |  | AL-X |  | 10 |  | 0 |  | 73.500 |  | 90.900 |  | 43.457 |  | 0.822 |  | .027 |  | 47.00 |  | 188.0 |  | 65.50 |  | 95.75 |  |
| ALP U/L |  | AH-X |  | 10 |  | 0 |  | 85.000 |  | 102.200 |  | 49.175 |  | 0.735 |  | .002 |  | 66.00 |  | 227.0 |  | 71.25 |  | 105.5 |  |
| ALP U/L |  | X |  | 6 |  | 2 |  | 93.500 |  | 112.500 |  | 36.828 |  | 0.780 |  | .039 |  | 85.00 |  | 174.0 |  | 88.50 |  | 130.0 |  |
| ALT U/L |  | C |  | 10 |  | 0 |  | 21.000 |  | 24.100 |  | 10.723 |  | 0.870 |  | .100 |  | 12.00 |  | 49.00 |  | 19.00 |  | 29.25 |  |
| ALT U/L |  | OV |  | 7 |  | 1 |  | 20.000 |  | 21.857 |  | 7.335 |  | 0.977 |  | .946 |  | 11.00 |  | 34.00 |  | 18.50 |  | 25.50 |  |
| ALT U/L |  | AL |  | 8 |  | 0 |  | 22.000 |  | 28.250 |  | 19.248 |  | 0.751 |  | .008 |  | 14.00 |  | 72.00 |  | 16.50 |  | 29.50 |  |
| ALT U/L |  | AH |  | 9 |  | 0 |  | 21.000 |  | 21.333 |  | 9.682 |  | 0.967 |  | .871 |  | 5.000 |  | 35.00 |  | 16.00 |  | 27.00 |  |
| ALT U/L |  | AL-X |  | 10 |  | 0 |  | 30.000 |  | 32.700 |  | 18.300 |  | 0.838 |  | .042 |  | 15.00 |  | 77.00 |  | 19.75 |  | 39.00 |  |
| ALT U/L |  | AH-X |  | 10 |  | 0 |  | 40.000 |  | 44.400 |  | 33.244 |  | 0.783 |  | .009 |  | 15.00 |  | 128.0 |  | 21.00 |  | 50.25 |  |
| ALT U/L |  | X |  | 7 |  | 1 |  | 26.000 |  | 24.429 |  | 7.934 |  | 0.982 |  | .968 |  | 12.00 |  | 35.00 |  | 19.50 |  | 29.50 |  |
| AMY U/L |  | C |  | 10 |  | 0 |  | 486.500 |  | 508.200 |  | 100.112 |  | 0.912 |  | .296 |  | 376.0 |  | 730.0 |  | 445.8 |  | 544.0 |  |
| AMY U/L |  | OV |  | 7 |  | 1 |  | 450.000 |  | 454.143 |  | 59.675 |  | 0.931 |  | .561 |  | 375.0 |  | 529.0 |  | 414.5 |  | 498.0 |  |
| AMY U/L |  | AL |  | 8 |  | 0 |  | 464.000 |  | 477.625 |  | 63.877 |  | 0.896 |  | .265 |  | 397.0 |  | 579.0 |  | 437.0 |  | 500.8 |  |
| AMY U/L |  | AH |  | 9 |  | 0 |  | 496.000 |  | 512.333 |  | 53.842 |  | 0.852 |  | .079 |  | 463.0 |  | 625.0 |  | 474.0 |  | 549.0 |  |
| AMY U/L |  | AL-X |  | 10 |  | 0 |  | 453.500 |  | 468.500 |  | 71.525 |  | 0.940 |  | .553 |  | 356.0 |  | 569.0 |  | 418.3 |  | 531.8 |  |
| AMY U/L |  | AH-X |  | 10 |  | 0 |  | 498.000 |  | 492.300 |  | 60.408 |  | 0.948 |  | .641 |  | 370.0 |  | 575.0 |  | 464.8 |  | 536.3 |  |
| AMY U/L |  | X |  | 7 |  | 1 |  | 469.000 |  | 466.429 |  | 58.688 |  | 0.963 |  | .844 |  | 366.0 |  | 547.0 |  | 443.0 |  | 498.5 |  |
| TBIL umol/L |  | C |  | 10 |  | 0 |  | 4.500 |  | 4.500 |  | 0.527 |  | 0.655 |  | < .001 |  | 4.000 |  | 5.000 |  | 4.000 |  | 5.000 |  |
| TBIL umol/L |  | OV |  | 7 |  | 1 |  | 5.000 |  | 4.714 |  | 1.496 |  | 0.851 |  | .126 |  | 2.000 |  | 6.000 |  | 4.000 |  | 6.000 |  |
| TBIL umol/L |  | AL |  | 8 |  | 0 |  | 5.000 |  | 5.000 |  | 0.926 |  | 0.757 |  | .010 |  | 4.000 |  | 7.000 |  | 4.750 |  | 5.000 |  |
| TBIL umol/L |  | AH |  | 9 |  | 0 |  | 5.000 |  | 4.778 |  | 0.441 |  | 0.536 |  | < .001 |  | 4.000 |  | 5.000 |  | 5.000 |  | 5.000 |  |
| TBIL umol/L |  | AL-X |  | 10 |  | 0 |  | 5.000 |  | 4.800 |  | 0.789 |  | 0.820 |  | .025 |  | 4.000 |  | 6.000 |  | 4.000 |  | 5.000 |  |
| TBIL umol/L |  | AH-X |  | 10 |  | 0 |  | 5.000 |  | 4.800 |  | 0.632 |  | 0.794 |  | .012 |  | 4.000 |  | 6.000 |  | 4.250 |  | 5.000 |  |
| TBIL umol/L |  | X |  | 6 |  | 2 |  | 5.000 |  | 5.000 |  | 0.894 |  | 0.853 |  | .167 |  | 4.000 |  | 6.000 |  | 4.250 |  | 5.750 |  |
| BUN mmol/L |  | C |  | 10 |  | 0 |  | 5.750 |  | 5.660 |  | 0.908 |  | 0.970 |  | .891 |  | 4.300 |  | 7.100 |  | 4.975 |  | 6.325 |  |
| BUN mmol/L |  | OV |  | 7 |  | 1 |  | 5.900 |  | 5.786 |  | 1.051 |  | 0.945 |  | .682 |  | 4.400 |  | 7.700 |  | 5.150 |  | 6.100 |  |
| BUN mmol/L |  | AL |  | 8 |  | 0 |  | 5.850 |  | 5.950 |  | 0.641 |  | 0.951 |  | .718 |  | 4.800 |  | 6.800 |  | 5.675 |  | 6.450 |  |
| BUN mmol/L |  | AH |  | 5 |  | 4 |  | 6.200 |  | 6.000 |  | 0.927 |  | 0.892 |  | .368 |  | 4.600 |  | 6.800 |  | 5.600 |  | 6.800 |  |
| BUN mmol/L |  | AL-X |  | 10 |  | 0 |  | 5.650 |  | 5.640 |  | 0.467 |  | 0.961 |  | .792 |  | 4.900 |  | 6.500 |  | 5.500 |  | 5.875 |  |
| BUN mmol/L |  | AH-X |  | 10 |  | 0 |  | 6.450 |  | 6.470 |  | 0.572 |  | 0.936 |  | .506 |  | 5.400 |  | 7.200 |  | 6.100 |  | 6.975 |  |
| BUN mmol/L |  | X |  | 7 |  | 1 |  | 7.000 |  | 6.486 |  | 0.997 |  | 0.904 |  | .354 |  | 5.200 |  | 7.900 |  | 5.650 |  | 7.000 |  |
| Ca mmol/L |  | C |  | 10 |  | 0 |  | 2.725 |  | 2.728 |  | 0.093 |  | 0.977 |  | .946 |  | 2.570 |  | 2.900 |  | 2.693 |  | 2.758 |  |
| Ca mmol/L |  | OV |  | 7 |  | 1 |  | 2.630 |  | 2.623 |  | 0.051 |  | 0.917 |  | .447 |  | 2.540 |  | 2.680 |  | 2.595 |  | 2.660 |  |
| Ca mmol/L |  | AL |  | 8 |  | 0 |  | 2.675 |  | 2.659 |  | 0.136 |  | 0.726 |  | .004 |  | 2.340 |  | 2.780 |  | 2.660 |  | 2.735 |  |
| Ca mmol/L |  | AH |  | 9 |  | 0 |  | 2.530 |  | 2.576 |  | 0.097 |  | 0.833 |  | .048 |  | 2.490 |  | 2.780 |  | 2.510 |  | 2.590 |  |
| Ca mmol/L |  | AL-X |  | 10 |  | 0 |  | 2.685 |  | 2.671 |  | 0.092 |  | 0.906 |  | .252 |  | 2.490 |  | 2.840 |  | 2.657 |  | 2.698 |  |
| Ca mmol/L |  | AH-X |  | 10 |  | 0 |  | 2.685 |  | 2.659 |  | 0.053 |  | 0.869 |  | .098 |  | 2.560 |  | 2.720 |  | 2.635 |  | 2.690 |  |
| Ca mmol/L |  | X |  | 7 |  | 1 |  | 2.610 |  | 2.519 |  | 0.311 |  | 0.667 |  | .002 |  | 1.830 |  | 2.750 |  | 2.565 |  | 2.655 |  |
| P mmol/L |  | C |  | 10 |  | 0 |  | 2.110 |  | 2.127 |  | 0.268 |  | 0.919 |  | .346 |  | 1.730 |  | 2.450 |  | 1.920 |  | 2.385 |  |
| P mmol/L |  | OV |  | 7 |  | 1 |  | 2.130 |  | 2.109 |  | 0.244 |  | 0.920 |  | .471 |  | 1.730 |  | 2.380 |  | 1.980 |  | 2.280 |  |
| P mmol/L |  | AL |  | 8 |  | 0 |  | 1.950 |  | 1.980 |  | 0.304 |  | 0.851 |  | .097 |  | 1.660 |  | 2.640 |  | 1.808 |  | 2.043 |  |
| P mmol/L |  | AH |  | 9 |  | 0 |  | 1.930 |  | 1.969 |  | 0.501 |  | 0.872 |  | .130 |  | 1.360 |  | 3.110 |  | 1.640 |  | 2.050 |  |
| P mmol/L |  | AL-X |  | 10 |  | 0 |  | 2.005 |  | 2.055 |  | 0.199 |  | 0.923 |  | .380 |  | 1.800 |  | 2.450 |  | 1.925 |  | 2.148 |  |
| P mmol/L |  | AH-X |  | 10 |  | 0 |  | 2.030 |  | 2.075 |  | 0.213 |  | 0.891 |  | .173 |  | 1.810 |  | 2.390 |  | 1.905 |  | 2.275 |  |
| P mmol/L |  | X |  | 7 |  | 1 |  | 2.050 |  | 2.100 |  | 0.235 |  | 0.851 |  | .125 |  | 1.850 |  | 2.580 |  | 1.970 |  | 2.140 |  |
| Cre umol/L |  | C |  | 10 |  | 0 |  | 39.000 |  | 39.700 |  | 12.401 |  | 0.945 |  | .613 |  | 20.00 |  | 59.00 |  | 35.00 |  | 44.75 |  |
| Cre umol/L |  | OV |  | 7 |  | 1 |  | 34.000 |  | 36.143 |  | 8.153 |  | 0.923 |  | .490 |  | 26.00 |  | 47.00 |  | 30.00 |  | 43.00 |  |
| Cre umol/L |  | AL |  | 8 |  | 0 |  | 36.000 |  | 38.125 |  | 6.999 |  | 0.944 |  | .649 |  | 27.00 |  | 50.00 |  | 35.00 |  | 42.50 |  |
| Cre umol/L |  | AH |  | 5 |  | 4 |  | 19.000 |  | 27.000 |  | 14.595 |  | 0.734 |  | .021 |  | 18.00 |  | 52.00 |  | 18.00 |  | 28.00 |  |
| Cre umol/L |  | AL-X |  | 10 |  | 0 |  | 48.500 |  | 45.700 |  | 16.607 |  | 0.912 |  | .292 |  | 18.00 |  | 66.00 |  | 37.50 |  | 58.50 |  |
| Cre umol/L |  | AH-X |  | 10 |  | 0 |  | 38.500 |  | 42.200 |  | 19.054 |  | 0.882 |  | .137 |  | 20.00 |  | 87.00 |  | 31.25 |  | 48.75 |  |
| Cre umol/L |  | X |  | 6 |  | 2 |  | 51.000 |  | 56.667 |  | 14.542 |  | 0.886 |  | .296 |  | 40.00 |  | 77.00 |  | 49.00 |  | 67.25 |  |
| Glu mmol/L |  | C |  | 10 |  | 0 |  | 13.550 |  | 13.700 |  | 2.486 |  | 0.914 |  | .313 |  | 9.800 |  | 18.90 |  | 13.13 |  | 13.98 |  |
| Glu mmol/L |  | OV |  | 7 |  | 1 |  | 15.500 |  | 15.400 |  | 2.946 |  | 0.931 |  | .557 |  | 10.30 |  | 18.60 |  | 14.00 |  | 17.70 |  |
| Glu mmol/L |  | AL |  | 8 |  | 0 |  | 16.000 |  | 15.350 |  | 2.305 |  | 0.897 |  | .269 |  | 10.70 |  | 17.70 |  | 14.40 |  | 16.70 |  |
| Glu mmol/L |  | AH |  | 8 |  | 1 |  | 15.850 |  | 15.863 |  | 1.939 |  | 0.965 |  | .858 |  | 13.30 |  | 19.10 |  | 14.25 |  | 17.15 |  |
| Glu mmol/L |  | AL-X |  | 10 |  | 0 |  | 16.350 |  | 16.250 |  | 1.590 |  | 0.971 |  | .902 |  | 13.90 |  | 19.20 |  | 15.03 |  | 16.88 |  |
| Glu mmol/L |  | AH-X |  | 10 |  | 0 |  | 15.950 |  | 15.680 |  | 1.874 |  | 0.955 |  | .723 |  | 12.40 |  | 18.60 |  | 14.30 |  | 16.65 |  |
| Glu mmol/L |  | X |  | 7 |  | 1 |  | 15.200 |  | 15.186 |  | 1.345 |  | 0.986 |  | .983 |  | 13.20 |  | 17.10 |  | 14.40 |  | 16.00 |  |
| Na mmol/L |  | C |  | 10 |  | 0 |  | 134.000 |  | 131.300 |  | 6.019 |  | 0.819 |  | .025 |  | 123.0 |  | 138.0 |  | 125.0 |  | 136.0 |  |
| Na mmol/L |  | OV |  | 7 |  | 1 |  | 132.000 |  | 131.571 |  | 4.429 |  | 0.711 |  | .005 |  | 122.0 |  | 135.0 |  | 132.0 |  | 134.0 |  |
| Na mmol/L |  | AL |  | 8 |  | 0 |  | 133.000 |  | 131.500 |  | 4.899 |  | 0.836 |  | .068 |  | 121.0 |  | 136.0 |  | 129.8 |  | 135.0 |  |
| Na mmol/L |  | AH |  | 9 |  | 0 |  | 124.000 |  | 127.667 |  | 7.314 |  | 0.785 |  | .014 |  | 120.0 |  | 136.0 |  | 122.0 |  | 135.0 |  |
| Na mmol/L |  | AL-X |  | 10 |  | 0 |  | 133.500 |  | 132.300 |  | 3.860 |  | 0.860 |  | .077 |  | 125.0 |  | 136.0 |  | 131.0 |  | 135.0 |  |
| Na mmol/L |  | AH-X |  | 10 |  | 0 |  | 133.000 |  | 132.500 |  | 4.743 |  | 0.922 |  | .376 |  | 123.0 |  | 139.0 |  | 130.0 |  | 136.0 |  |
| Na mmol/L |  | X |  | 6 |  | 2 |  | 133.000 |  | 132.000 |  | 4.858 |  | 0.934 |  | .614 |  | 126.0 |  | 139.0 |  | 128.3 |  | 134.0 |  |
| K mmol/L |  | C |  | 10 |  | 0 |  | 4.550 |  | 4.520 |  | 0.391 |  | 0.927 |  | .416 |  | 4.000 |  | 5.100 |  | 4.225 |  | 4.800 |  |
| K mmol/L |  | OV |  | 7 |  | 1 |  | 4.600 |  | 4.500 |  | 0.447 |  | 0.909 |  | .390 |  | 4.000 |  | 5.200 |  | 4.100 |  | 4.750 |  |
| K mmol/L |  | AL |  | 8 |  | 0 |  | 4.650 |  | 4.588 |  | 0.314 |  | 0.912 |  | .368 |  | 4.000 |  | 4.900 |  | 4.450 |  | 4.825 |  |
| K mmol/L |  | AH |  | 9 |  | 0 |  | 4.500 |  | 4.700 |  | 0.642 |  | 0.953 |  | .723 |  | 3.800 |  | 5.800 |  | 4.300 |  | 5.200 |  |
| K mmol/L |  | AL-X |  | 10 |  | 0 |  | 4.600 |  | 4.670 |  | 0.574 |  | 0.969 |  | .883 |  | 3.800 |  | 5.600 |  | 4.325 |  | 5.150 |  |
| K mmol/L |  | AH-X |  | 10 |  | 0 |  | 4.900 |  | 4.810 |  | 0.495 |  | 0.945 |  | .605 |  | 4.000 |  | 5.500 |  | 4.600 |  | 5.075 |  |
| K mmol/L |  | X |  | 6 |  | 2 |  | 4.500 |  | 4.700 |  | 0.885 |  | 0.899 |  | .371 |  | 3.800 |  | 6.300 |  | 4.175 |  | 4.900 |  |
| TP g/L |  | C |  | 10 |  | 0 |  | 65.500 |  | 64.600 |  | 2.633 |  | 0.872 |  | .106 |  | 59.00 |  | 67.00 |  | 63.25 |  | 66.75 |  |
| TP g/L |  | OV |  | 7 |  | 1 |  | 58.000 |  | 58.571 |  | 3.505 |  | 0.978 |  | .948 |  | 53.00 |  | 64.00 |  | 57.00 |  | 60.50 |  |
| TP g/L |  | AL |  | 8 |  | 0 |  | 61.000 |  | 60.750 |  | 3.732 |  | 0.956 |  | .775 |  | 54.00 |  | 67.00 |  | 59.00 |  | 62.25 |  |
| TP g/L |  | AH |  | 9 |  | 0 |  | 60.000 |  | 60.667 |  | 4.444 |  | 0.827 |  | .042 |  | 56.00 |  | 71.00 |  | 58.00 |  | 62.00 |  |
| TP g/L |  | AL-X |  | 10 |  | 0 |  | 61.000 |  | 61.600 |  | 4.477 |  | 0.880 |  | .130 |  | 54.00 |  | 71.00 |  | 61.00 |  | 61.75 |  |
| TP g/L |  | AH-X |  | 10 |  | 0 |  | 58.500 |  | 58.600 |  | 2.011 |  | 0.925 |  | .397 |  | 55.00 |  | 61.00 |  | 58.00 |  | 60.00 |  |
| TP g/L |  | X |  | 7 |  | 1 |  | 61.000 |  | 61.143 |  | 3.532 |  | 0.895 |  | .304 |  | 57.00 |  | 68.00 |  | 59.00 |  | 62.00 |  |
| Glob g/L |  | C |  | 10 |  | 0 |  | 11.000 |  | 12.000 |  | 3.266 |  | 0.902 |  | .229 |  | 8.000 |  | 19.00 |  | 10.00 |  | 14.00 |  |
| Glob g/L |  | OV |  | 7 |  | 1 |  | 13.000 |  | 12.286 |  | 4.716 |  | 0.915 |  | .434 |  | 7.000 |  | 19.00 |  | 8.000 |  | 15.50 |  |
| Glob g/L |  | AL |  | 8 |  | 0 |  | 14.000 |  | 14.000 |  | 2.268 |  | 0.914 |  | .385 |  | 11.00 |  | 17.00 |  | 12.00 |  | 15.50 |  |
| Glob g/L |  | AH |  | 9 |  | 0 |  | 10.000 |  | 10.444 |  | 1.424 |  | 0.899 |  | .246 |  | 9.000 |  | 13.00 |  | 9.000 |  | 11.00 |  |
| Glob g/L |  | AL-X |  | 10 |  | 0 |  | 12.000 |  | 12.300 |  | 2.312 |  | 0.882 |  | .139 |  | 8.000 |  | 15.00 |  | 11.00 |  | 14.50 |  |
| Glob g/L |  | AH-X |  | 10 |  | 0 |  | 11.500 |  | 11.400 |  | 2.503 |  | 0.965 |  | .842 |  | 7.000 |  | 15.00 |  | 10.00 |  | 13.50 |  |
| Glob g/L |  | X |  | 6 |  | 2 |  | 11.000 |  | 11.667 |  | 1.862 |  | 0.788 |  | .045 |  | 10.00 |  | 14.00 |  | 10.25 |  | 13.25 |  |
| WBC 10^9/l |  | C |  | 10 |  | 0 |  | 3.400 |  | 3.520 |  | 0.899 |  | 0.958 |  | .759 |  | 2.100 |  | 4.900 |  | 2.850 |  | 4.250 |  |
| WBC 10^9/l |  | OV |  | 7 |  | 1 |  | 4.200 |  | 5.300 |  | 2.792 |  | 0.625 |  | < .001 |  | 3.500 |  | 11.50 |  | 4.100 |  | 4.850 |  |
| WBC 10^9/l |  | AL |  | 8 |  | 0 |  | 4.150 |  | 4.313 |  | 1.137 |  | 0.943 |  | .639 |  | 2.600 |  | 6.100 |  | 3.850 |  | 4.725 |  |
| WBC 10^9/l |  | AH |  | 9 |  | 0 |  | 5.300 |  | 5.222 |  | 1.213 |  | 0.933 |  | .514 |  | 3.500 |  | 7.000 |  | 4.700 |  | 5.700 |  |
| WBC 10^9/l |  | AL-X |  | 10 |  | 0 |  | 3.700 |  | 3.920 |  | 1.166 |  | 0.965 |  | .846 |  | 2.100 |  | 6.100 |  | 3.500 |  | 4.350 |  |
| WBC 10^9/l |  | AH-X |  | 10 |  | 0 |  | 5.500 |  | 5.220 |  | 1.311 |  | 0.897 |  | .205 |  | 3.400 |  | 6.900 |  | 3.900 |  | 6.350 |  |
| WBC 10^9/l |  | X |  | 6 |  | 2 |  | 5.150 |  | 5.567 |  | 0.896 |  | 0.815 |  | .079 |  | 4.700 |  | 6.800 |  | 5.025 |  | 6.250 |  |
| RBC 10^12/l |  | C |  | 10 |  | 0 |  | 6.995 |  | 6.956 |  | 0.258 |  | 0.968 |  | .872 |  | 6.490 |  | 7.340 |  | 6.793 |  | 7.073 |  |
| RBC 10^12/l |  | OV |  | 7 |  | 1 |  | 7.750 |  | 7.819 |  | 0.515 |  | 0.929 |  | .544 |  | 7.280 |  | 8.730 |  | 7.420 |  | 8.065 |  |
| RBC 10^12/l |  | AL |  | 8 |  | 0 |  | 7.820 |  | 7.791 |  | 0.813 |  | 0.858 |  | .114 |  | 6.800 |  | 8.720 |  | 7.073 |  | 8.555 |  |
| RBC 10^12/l |  | AH |  | 9 |  | 0 |  | 7.480 |  | 7.268 |  | 0.631 |  | 0.910 |  | .316 |  | 6.090 |  | 7.950 |  | 6.830 |  | 7.720 |  |
| RBC 10^12/l |  | AL-X |  | 9 |  | 1 |  | 7.950 |  | 7.986 |  | 0.321 |  | 0.927 |  | .457 |  | 7.490 |  | 8.660 |  | 7.860 |  | 8.130 |  |
| RBC 10^12/l |  | AH-X |  | 10 |  | 0 |  | 7.235 |  | 7.322 |  | 0.812 |  | 0.964 |  | .825 |  | 5.910 |  | 8.880 |  | 7.073 |  | 7.783 |  |
| RBC 10^12/l |  | X |  | 6 |  | 2 |  | 7.130 |  | 7.020 |  | 0.488 |  | 0.779 |  | .038 |  | 6.070 |  | 7.430 |  | 7.047 |  | 7.288 |  |
| HGB g/l |  | C |  | 10 |  | 0 |  | 153.000 |  | 152.200 |  | 5.203 |  | 0.951 |  | .677 |  | 143.0 |  | 159.0 |  | 148.0 |  | 155.8 |  |
| HGB g/l |  | OV |  | 7 |  | 1 |  | 170.000 |  | 170.143 |  | 11.335 |  | 0.879 |  | .222 |  | 156.0 |  | 183.0 |  | 160.5 |  | 180.5 |  |
| HGB g/l |  | AL |  | 8 |  | 0 |  | 176.000 |  | 175.875 |  | 17.868 |  | 0.932 |  | .530 |  | 150.0 |  | 200.0 |  | 163.0 |  | 190.3 |  |
| HGB g/l |  | AH |  | 9 |  | 0 |  | 171.000 |  | 167.889 |  | 10.349 |  | 0.879 |  | .152 |  | 154.0 |  | 180.0 |  | 158.0 |  | 177.0 |  |
| HGB g/l |  | AL-X |  | 10 |  | 0 |  | 172.500 |  | 176.700 |  | 17.733 |  | 0.841 |  | .045 |  | 155.0 |  | 220.0 |  | 168.5 |  | 182.3 |  |
| HGB g/l |  | AH-X |  | 10 |  | 0 |  | 171.500 |  | 166.800 |  | 11.612 |  | 0.869 |  | .097 |  | 140.0 |  | 180.0 |  | 162.3 |  | 174.3 |  |
| HGB g/l |  | X |  | 6 |  | 2 |  | 162.500 |  | 161.500 |  | 9.203 |  | 0.807 |  | .068 |  | 144.0 |  | 170.0 |  | 162.0 |  | 166.8 |  |
| HCT l/l |  | C |  | 10 |  | 0 |  | 0.404 |  | 0.406 |  | 0.012 |  | 0.920 |  | .356 |  | 0.391 |  | 0.428 |  | 0.399 |  | 0.413 |  |
| HCT l/l |  | OV |  | 7 |  | 1 |  | 0.453 |  | 0.456 |  | 0.030 |  | 0.930 |  | .555 |  | 0.419 |  | 0.496 |  | 0.433 |  | 0.479 |  |
| HCT l/l |  | AL |  | 8 |  | 0 |  | 0.459 |  | 0.458 |  | 0.051 |  | 0.838 |  | .073 |  | 0.397 |  | 0.519 |  | 0.410 |  | 0.503 |  |
| HCT l/l |  | AH |  | 9 |  | 0 |  | 0.424 |  | 0.427 |  | 0.033 |  | 0.945 |  | .635 |  | 0.366 |  | 0.465 |  | 0.409 |  | 0.453 |  |
| HCT l/l |  | AL-X |  | 9 |  | 1 |  | 0.461 |  | 0.476 |  | 0.050 |  | 0.783 |  | .013 |  | 0.435 |  | 0.594 |  | 0.447 |  | 0.489 |  |
| HCT l/l |  | AH-X |  | 10 |  | 0 |  | 0.436 |  | 0.434 |  | 0.036 |  | 0.991 |  | .998 |  | 0.367 |  | 0.496 |  | 0.419 |  | 0.455 |  |
| HCT l/l |  | X |  | 6 |  | 2 |  | 0.421 |  | 0.414 |  | 0.025 |  | 0.826 |  | .100 |  | 0.368 |  | 0.442 |  | 0.415 |  | 0.422 |  |
| MCV fl |  | C |  | 10 |  | 0 |  | 58.500 |  | 58.400 |  | 1.955 |  | 0.940 |  | .555 |  | 55.00 |  | 61.00 |  | 57.25 |  | 60.00 |  |
| MCV fl |  | OV |  | 7 |  | 1 |  | 57.000 |  | 58.286 |  | 2.628 |  | 0.784 |  | .028 |  | 56.00 |  | 62.00 |  | 56.50 |  | 60.00 |  |
| MCV fl |  | AL |  | 8 |  | 0 |  | 59.000 |  | 58.750 |  | 1.035 |  | 0.917 |  | .408 |  | 57.00 |  | 60.00 |  | 58.00 |  | 59.25 |  |
| MCV fl |  | AH |  | 9 |  | 0 |  | 59.000 |  | 58.889 |  | 1.364 |  | 0.931 |  | .494 |  | 57.00 |  | 61.00 |  | 58.00 |  | 60.00 |  |
| MCV fl |  | AL-X |  | 10 |  | 0 |  | 58.000 |  | 59.400 |  | 3.950 |  | 0.789 |  | .011 |  | 56.00 |  | 69.00 |  | 57.25 |  | 59.75 |  |
| MCV fl |  | AH-X |  | 10 |  | 0 |  | 59.500 |  | 59.500 |  | 1.900 |  | 0.953 |  | .699 |  | 56.00 |  | 62.00 |  | 58.25 |  | 60.75 |  |
| MCV fl |  | X |  | 6 |  | 2 |  | 59.000 |  | 59.167 |  | 1.169 |  | 0.908 |  | .421 |  | 58.00 |  | 61.00 |  | 58.25 |  | 59.75 |  |
| MCH pg |  | C |  | 10 |  | 0 |  | 22.000 |  | 21.890 |  | 0.679 |  | 0.865 |  | .087 |  | 20.80 |  | 22.60 |  | 21.33 |  | 22.50 |  |
| MCH pg |  | OV |  | 7 |  | 1 |  | 21.500 |  | 21.800 |  | 1.066 |  | 0.924 |  | .500 |  | 20.60 |  | 23.40 |  | 21.05 |  | 22.50 |  |
| MCH pg |  | AL |  | 8 |  | 0 |  | 22.450 |  | 22.563 |  | 0.735 |  | 0.984 |  | .980 |  | 21.40 |  | 23.80 |  | 22.17 |  | 22.97 |  |
| MCH pg |  | AH |  | 9 |  | 0 |  | 22.600 |  | 23.211 |  | 1.947 |  | 0.647 |  | < .001 |  | 21.50 |  | 28.20 |  | 22.50 |  | 23.10 |  |
| MCH pg |  | AL-X |  | 9 |  | 1 |  | 22.100 |  | 22.367 |  | 1.339 |  | 0.831 |  | .046 |  | 21.20 |  | 25.40 |  | 21.40 |  | 22.80 |  |
| MCH pg |  | AH-X |  | 10 |  | 0 |  | 23.200 |  | 22.880 |  | 1.223 |  | 0.915 |  | .318 |  | 20.30 |  | 24.30 |  | 22.23 |  | 23.58 |  |
| MCH pg |  | X |  | 6 |  | 2 |  | 23.000 |  | 23.017 |  | 0.567 |  | 0.961 |  | .824 |  | 22.10 |  | 23.70 |  | 22.83 |  | 23.40 |  |
| MCHC g/l |  | C |  | 10 |  | 0 |  | 376.500 |  | 375.100 |  | 8.034 |  | 0.959 |  | .777 |  | 364.0 |  | 389.0 |  | 369.0 |  | 378.5 |  |
| MCHC g/l |  | OV |  | 7 |  | 1 |  | 372.000 |  | 373.286 |  | 4.821 |  | 0.818 |  | .062 |  | 369.0 |  | 383.0 |  | 370.5 |  | 374.0 |  |
| MCHC g/l |  | AL |  | 8 |  | 0 |  | 387.000 |  | 384.625 |  | 10.676 |  | 0.958 |  | .789 |  | 365.0 |  | 399.0 |  | 378.0 |  | 390.8 |  |
| MCHC g/l |  | AH |  | 9 |  | 0 |  | 385.000 |  | 394.889 |  | 29.165 |  | 0.654 |  | < .001 |  | 375.0 |  | 469.0 |  | 379.0 |  | 392.0 |  |
| MCHC g/l |  | AL-X |  | 9 |  | 1 |  | 378.000 |  | 376.222 |  | 6.300 |  | 0.947 |  | .659 |  | 366.0 |  | 385.0 |  | 370.0 |  | 380.0 |  |
| MCHC g/l |  | AH-X |  | 10 |  | 0 |  | 386.500 |  | 384.600 |  | 10.741 |  | 0.949 |  | .662 |  | 364.0 |  | 398.0 |  | 377.0 |  | 391.3 |  |
| MCHC g/l |  | X |  | 6 |  | 2 |  | 386.500 |  | 390.000 |  | 8.832 |  | 0.752 |  | .021 |  | 384.0 |  | 407.0 |  | 384.5 |  | 390.8 |  |
| PLT 10^9/l |  | C |  | 10 |  | 0 |  | 722.500 |  | 716.500 |  | 37.728 |  | 0.956 |  | .741 |  | 662.0 |  | 783.0 |  | 692.0 |  | 739.3 |  |
| PLT 10^9/l |  | OV |  | 7 |  | 1 |  | 653.000 |  | 623.000 |  | 64.200 |  | 0.843 |  | .106 |  | 524.0 |  | 684.0 |  | 582.0 |  | 668.0 |  |
| PLT 10^9/l |  | AL |  | 8 |  | 0 |  | 569.000 |  | 594.375 |  | 104.033 |  | 0.937 |  | .584 |  | 436.0 |  | 729.0 |  | 542.5 |  | 680.5 |  |
| PLT 10^9/l |  | AH |  | 9 |  | 0 |  | 549.000 |  | 543.444 |  | 94.714 |  | 0.913 |  | .337 |  | 382.0 |  | 649.0 |  | 515.0 |  | 615.0 |  |
| PLT 10^9/l |  | AL-X |  | 10 |  | 0 |  | 534.500 |  | 556.200 |  | 98.295 |  | 0.967 |  | .865 |  | 413.0 |  | 754.0 |  | 503.3 |  | 611.0 |  |
| PLT 10^9/l |  | AH-X |  | 10 |  | 0 |  | 541.000 |  | 507.200 |  | 90.081 |  | 0.851 |  | .060 |  | 379.0 |  | 602.0 |  | 420.8 |  | 579.5 |  |
| PLT 10^9/l |  | X |  | 6 |  | 2 |  | 628.500 |  | 620.833 |  | 29.715 |  | 0.955 |  | .781 |  | 574.0 |  | 657.0 |  | 605.5 |  | 636.5 |  |
|  | | | | | | | | | | | | | | | | | | | | | | | | | |

### Boxplots

#### ALB g/l

#### ALP U/L

#### ALT U/L

#### AMY U/L

#### TBIL umol/L

#### BUN mmol/L

#### Ca mmol/L

#### P mmol/L

#### Cre umol/L

#### Glu mmol/L

#### Na mmol/L

#### K mmol/L

#### TP g/L

#### Glob g/L

#### WBC 10^9/l

#### RBC 10^12/l

#### HGB g/l

#### HCT l/l

#### MCV fl

#### MCH pg

#### MCHC g/l

#### PLT 10^9/l

## Albumin

| ANOVA - ALB g/l | | | | | | | | | | | | | | | | | | | | | | | | | |
| --- | --- | --- | --- | --- | --- | --- | --- | --- | --- | --- | --- | --- | --- | --- | --- | --- | --- | --- | --- | --- | --- | --- | --- | --- | --- |
|  | | | | | | | | | | | | | | | | 95% CI for η² | | | |  | | 95% CI for ω² | | | |
| Homogeneity Correction | | Cases | | Sum of Squares | | df | | Mean Square | | F | | p | | η² | | Lower | | Upper | | ω² | | Lower | | Upper | |
| None |  | Animal group |  | 274.1 |  | 6.000 |  | 45.68 |  | 2.593 |  | .028 |  | 0.227 |  | 0.000 |  | 0.364 |  | 0.137 |  | 0.000 |  | 0.252 |  |
|  |  | Residuals |  | 933.5 |  | 53.000 |  | 17.61 |  |  |  |  |  |  |  |  |  |  |  |  |  |  |  |  |  |
| Welch |  | Animal group |  | 274.1 |  | 6.000 |  | 45.68 |  | 2.634 |  | .044 |  | 0.227 |  | 0.000 |  | 0.364 |  | 0.137 |  | 0.000 |  | 0.252 |  |
|  |  | Residuals |  | 933.5 |  | 22.288 |  | 41.88 |  |  |  |  |  |  |  |  |  |  |  |  |  |  |  |  |  |
|  | | | | | | | | | | | | | | | | | | | | | | | | | |
|  |  |  |  |  |  |  |  |  |  |  |  |  |  |  |  |  |  |  |  |  |  |  |  |  |  |
| --- | --- | --- | --- | --- | --- | --- | --- | --- | --- | --- | --- | --- | --- | --- | --- | --- | --- | --- | --- | --- | --- | --- | --- | --- | --- |
| *Note.*  Type III Sum of Squares | | | | | | | | | | | | | | | | | | | | | | | | | |

### Descriptives

| Descriptives - ALB g/l | | | | | | | | | | | |
| --- | --- | --- | --- | --- | --- | --- | --- | --- | --- | --- | --- |
| Animal group | | N | | Mean | | SD | | SE | | Coefficient of variation | |
| C |  | 10 |  | 52.60 |  | 3.950 |  | 1.249 |  | 0.075 |  |
| OV |  | 7 |  | 46.29 |  | 4.499 |  | 1.700 |  | 0.097 |  |
| AL |  | 8 |  | 46.75 |  | 4.496 |  | 1.590 |  | 0.096 |  |
| AH |  | 9 |  | 50.11 |  | 5.061 |  | 1.687 |  | 0.101 |  |
| AL-X |  | 10 |  | 49.40 |  | 5.168 |  | 1.634 |  | 0.105 |  |
| AH-X |  | 10 |  | 47.10 |  | 2.514 |  | 0.795 |  | 0.053 |  |
| X |  | 6 |  | 48.00 |  | 2.366 |  | 0.966 |  | 0.049 |  |
|  | | | | | | | | | | | |

#### Bar plots

#####

### Assumption Checks

| Test for Equality of Variances (Levene's) | | | | | | | |
| --- | --- | --- | --- | --- | --- | --- | --- |
| F | | df1 | | df2 | | p | |
| 0.726 |  | 6.000 |  | 53.00 |  | .631 |  |
|  | | | | | | | |

### Post Hoc Tests

#### Standard (HSD)

| Post Hoc Comparisons - Animal group | | | | | | | | | | | | | | | | | | | |
| --- | --- | --- | --- | --- | --- | --- | --- | --- | --- | --- | --- | --- | --- | --- | --- | --- | --- | --- | --- |
|  | | | | | | 95% CI for Mean Difference | | | |  | | | | | | | | | |
|  | |  | | Mean Difference | | Lower | | Upper | | SE | | df | | t | | ptukey | | pbonf | |
| C |  | OV |  | 6.314 |  | -0.023 |  | 12.652 |  | 2.068 |  | 53 |  | 3.053 |  | .051 |  | .074 |  |
|  |  | AL |  | 5.850 |  | -0.250 |  | 11.950 |  | 1.991 |  | 53 |  | 2.939 |  | .068 |  | .102 |  |
|  |  | AH |  | 2.489 |  | -3.420 |  | 8.398 |  | 1.928 |  | 53 |  | 1.291 |  | .853 |  | 1.000 |  |
|  |  | (AL-X) |  | 3.200 |  | -2.551 |  | 8.951 |  | 1.877 |  | 53 |  | 1.705 |  | .616 |  | 1.000 |  |
|  |  | (AH-X) |  | 5.500 |  | -0.251 |  | 11.251 |  | 1.877 |  | 53 |  | 2.930 |  | .070 |  | .105 |  |
|  |  | X |  | 4.600 |  | -2.041 |  | 11.241 |  | 2.167 |  | 53 |  | 2.123 |  | .355 |  | .808 |  |
| OV |  | AL |  | -0.464 |  | -7.120 |  | 6.192 |  | 2.172 |  | 53 |  | -0.214 |  | 1.000 |  | 1.000 |  |
|  |  | AH |  | -3.825 |  | -10.307 |  | 2.656 |  | 2.115 |  | 53 |  | -1.809 |  | .548 |  | 1.000 |  |
|  |  | (AL-X) |  | -3.114 |  | -9.452 |  | 3.223 |  | 2.068 |  | 53 |  | -1.506 |  | .740 |  | 1.000 |  |
|  |  | (AH-X) |  | -0.814 |  | -7.152 |  | 5.523 |  | 2.068 |  | 53 |  | -0.394 |  | 1.000 |  | 1.000 |  |
|  |  | X |  | -1.714 |  | -8.869 |  | 5.441 |  | 2.335 |  | 53 |  | -0.734 |  | .990 |  | 1.000 |  |
| AL |  | AH |  | -3.361 |  | -9.610 |  | 2.888 |  | 2.039 |  | 53 |  | -1.648 |  | .652 |  | 1.000 |  |
|  |  | (AL-X) |  | -2.650 |  | -8.750 |  | 3.450 |  | 1.991 |  | 53 |  | -1.331 |  | .834 |  | 1.000 |  |
|  |  | (AH-X) |  | -0.350 |  | -6.450 |  | 5.750 |  | 1.991 |  | 53 |  | -0.176 |  | 1.000 |  | 1.000 |  |
|  |  | X |  | -1.250 |  | -8.196 |  | 5.696 |  | 2.267 |  | 53 |  | -0.551 |  | .998 |  | 1.000 |  |
| AH |  | (AL-X) |  | 0.711 |  | -5.198 |  | 6.620 |  | 1.928 |  | 53 |  | 0.369 |  | 1.000 |  | 1.000 |  |
|  |  | (AH-X) |  | 3.011 |  | -2.898 |  | 8.920 |  | 1.928 |  | 53 |  | 1.562 |  | .707 |  | 1.000 |  |
|  |  | X |  | 2.111 |  | -4.667 |  | 8.889 |  | 2.212 |  | 53 |  | 0.954 |  | .961 |  | 1.000 |  |
| (AL-X) |  | (AH-X) |  | 2.300 |  | -3.451 |  | 8.051 |  | 1.877 |  | 53 |  | 1.225 |  | .881 |  | 1.000 |  |
|  |  | X |  | 1.400 |  | -5.241 |  | 8.041 |  | 2.167 |  | 53 |  | 0.646 |  | .995 |  | 1.000 |  |
| (AH-X) |  | X |  | -0.900 |  | -7.541 |  | 5.741 |  | 2.167 |  | 53 |  | -0.415 |  | 1.000 |  | 1.000 |  |
|  | | | | | | | | | | | | | | | | | | | |
|  |  |  |  |  |  |  |  |  |  |  |  |  |  |  |  |  |  |  |  |
| --- | --- | --- | --- | --- | --- | --- | --- | --- | --- | --- | --- | --- | --- | --- | --- | --- | --- | --- | --- |
| *Note.*  P-value and confidence intervals adjusted for comparing a family of 7 estimates (confidence intervals corrected using the tukey method). | | | | | | | | | | | | | | | | | | | |

| Letter-Based Grouping - Animal group | | | |
| --- | --- | --- | --- |
| Animal group | | Letter | |
| C |  | a |  |
| OV |  | a |  |
| AL |  | a |  |
| AH |  | a |  |
| AL-X |  | a |  |
| AH-X |  | a |  |
| X |  | a |  |
|  | | | |
|  |  |  |  |
| --- | --- | --- | --- |
| *Note.*  If two or more means share the same grouping symbol, then we cannot show them to be different, but we also did not show them to be the same. | | | |

## ALP

| ANOVA - ALP U/L | | | | | | | | | | | | | | | | | | | | | | | | | |
| --- | --- | --- | --- | --- | --- | --- | --- | --- | --- | --- | --- | --- | --- | --- | --- | --- | --- | --- | --- | --- | --- | --- | --- | --- | --- |
|  | | | | | | | | | | | | | | | | 95% CI for η² | | | |  | | 95% CI for ω² | | | |
| Homogeneity Correction | | Cases | | Sum of Squares | | df | | Mean Square | | F | | p | | η² | | Lower | | Upper | | ω² | | Lower | | Upper | |
| None |  | Animal group |  | 14366 |  | 6.000 |  | 2394 |  | 1.667 |  | .149 |  | 0.170 |  | 0.000 |  | 0.298 |  | 0.067 |  | 0.000 |  | 0.133 |  |
|  |  | Residuals |  | 70379 |  | 49.000 |  | 1436 |  |  |  |  |  |  |  |  |  |  |  |  |  |  |  |  |  |
| Welch |  | Animal group |  | 14366 |  | 6.000 |  | 2394 |  | 2.062 |  | .106 |  | 0.170 |  | 0.000 |  | 0.298 |  | 0.067 |  | 0.000 |  | 0.133 |  |
|  |  | Residuals |  | 70379 |  | 19.206 |  | 3665 |  |  |  |  |  |  |  |  |  |  |  |  |  |  |  |  |  |
|  | | | | | | | | | | | | | | | | | | | | | | | | | |
|  |  |  |  |  |  |  |  |  |  |  |  |  |  |  |  |  |  |  |  |  |  |  |  |  |  |
| --- | --- | --- | --- | --- | --- | --- | --- | --- | --- | --- | --- | --- | --- | --- | --- | --- | --- | --- | --- | --- | --- | --- | --- | --- | --- |
| *Note.*  Type III Sum of Squares | | | | | | | | | | | | | | | | | | | | | | | | | |

### Descriptives

| Descriptives - ALP U/L | | | | | | | | | | | |
| --- | --- | --- | --- | --- | --- | --- | --- | --- | --- | --- | --- |
| Animal group | | N | | Mean | | SD | | SE | | Coefficient of variation | |
| C |  | 10 |  | 66.10 |  | 29.34 |  | 9.278 |  | 0.444 |  |
| OV |  | 7 |  | 97.57 |  | 40.48 |  | 15.298 |  | 0.415 |  |
| AL |  | 8 |  | 68.13 |  | 19.42 |  | 6.867 |  | 0.285 |  |
| AH |  | 5 |  | 92.00 |  | 33.99 |  | 15.199 |  | 0.369 |  |
| AL-X |  | 10 |  | 90.90 |  | 43.46 |  | 13.742 |  | 0.478 |  |
| AH-X |  | 10 |  | 102.20 |  | 49.17 |  | 15.550 |  | 0.481 |  |
| X |  | 6 |  | 112.50 |  | 36.83 |  | 15.035 |  | 0.327 |  |
|  | | | | | | | | | | | |

#### Bar plots

#####

### Assumption Checks

| Test for Equality of Variances (Levene's) | | | | | | | |
| --- | --- | --- | --- | --- | --- | --- | --- |
| F | | df1 | | df2 | | p | |
| 0.933 |  | 6.000 |  | 49.00 |  | .480 |  |
|  | | | | | | | |

## ALT

| ANOVA - ALT U/L | | | | | | | | | | | | | | | | | | | | | | | | | |
| --- | --- | --- | --- | --- | --- | --- | --- | --- | --- | --- | --- | --- | --- | --- | --- | --- | --- | --- | --- | --- | --- | --- | --- | --- | --- |
|  | | | | | | | | | | | | | | | | 95% CI for η² | | | |  | | 95% CI for ω² | | | |
| Homogeneity Correction | | Cases | | Sum of Squares | | df | | Mean Square | | F | | p | | η² | | Lower | | Upper | | ω² | | Lower | | Upper | |
| None |  | Animal group |  | 3782 |  | 6.000 |  | 630.3 |  | 1.887 |  | .100 |  | 0.173 |  | 0.000 |  | 0.299 |  | 0.080 |  | 0.000 |  | 0.161 |  |
|  |  | Residuals |  | 18039 |  | 54.000 |  | 334.1 |  |  |  |  |  |  |  |  |  |  |  |  |  |  |  |  |  |
| Welch |  | Animal group |  | 3782 |  | 6.000 |  | 630.3 |  | 1.126 |  | .378 |  | 0.173 |  | 0.000 |  | 0.299 |  | 0.080 |  | 0.000 |  | 0.161 |  |
|  |  | Residuals |  | 18039 |  | 23.576 |  | 765.2 |  |  |  |  |  |  |  |  |  |  |  |  |  |  |  |  |  |
|  | | | | | | | | | | | | | | | | | | | | | | | | | |
|  |  |  |  |  |  |  |  |  |  |  |  |  |  |  |  |  |  |  |  |  |  |  |  |  |  |
| --- | --- | --- | --- | --- | --- | --- | --- | --- | --- | --- | --- | --- | --- | --- | --- | --- | --- | --- | --- | --- | --- | --- | --- | --- | --- |
| *Note.*  Type III Sum of Squares | | | | | | | | | | | | | | | | | | | | | | | | | |

### Descriptives

| Descriptives - ALT U/L | | | | | | | | | | | |
| --- | --- | --- | --- | --- | --- | --- | --- | --- | --- | --- | --- |
| Animal group | | N | | Mean | | SD | | SE | | Coefficient of variation | |
| C |  | 10 |  | 24.10 |  | 10.723 |  | 3.391 |  | 0.445 |  |
| OV |  | 7 |  | 21.86 |  | 7.335 |  | 2.773 |  | 0.336 |  |
| AL |  | 8 |  | 28.25 |  | 19.248 |  | 6.805 |  | 0.681 |  |
| AH |  | 9 |  | 21.33 |  | 9.682 |  | 3.227 |  | 0.454 |  |
| AL-X |  | 10 |  | 32.70 |  | 18.300 |  | 5.787 |  | 0.560 |  |
| AH-X |  | 10 |  | 44.40 |  | 33.244 |  | 10.513 |  | 0.749 |  |
| X |  | 7 |  | 24.43 |  | 7.934 |  | 2.999 |  | 0.325 |  |
|  | | | | | | | | | | | |

#### Bar plots

#####

### Assumption Checks

| Test for Equality of Variances (Levene's) | | | | | | | |
| --- | --- | --- | --- | --- | --- | --- | --- |
| F | | df1 | | df2 | | p | |
| 2.072 |  | 6.000 |  | 54.00 |  | .072 |  |
|  | | | | | | | |

## AMY

| ANOVA - AMY U/L | | | | | | | | | | | | | | | | | | | | | | | | | |
| --- | --- | --- | --- | --- | --- | --- | --- | --- | --- | --- | --- | --- | --- | --- | --- | --- | --- | --- | --- | --- | --- | --- | --- | --- | --- |
|  | | | | | | | | | | | | | | | | 95% CI for η² | | | |  | | 95% CI for ω² | | | |
| Homogeneity Correction | | Cases | | Sum of Squares | | df | | Mean Square | | F | | p | | η² | | Lower | | Upper | | ω² | | Lower | | Upper | |
| None |  | Animal group |  | 24870 |  | 6.000 |  | 4145 |  | 0.851 |  | .536 |  | 0.086 |  | 0.000 |  | 0.172 |  | 0.000 |  | 0.000 |  | 0.000 |  |
|  |  | Residuals |  | 262873 |  | 54.000 |  | 4868 |  |  |  |  |  |  |  |  |  |  |  |  |  |  |  |  |  |
| Welch |  | Animal group |  | 24870 |  | 6.000 |  | 4145 |  | 0.903 |  | .510 |  | 0.086 |  | 0.000 |  | 0.172 |  | 0.000 |  | 0.000 |  | 0.000 |  |
|  |  | Residuals |  | 262873 |  | 23.243 |  | 11310 |  |  |  |  |  |  |  |  |  |  |  |  |  |  |  |  |  |
|  | | | | | | | | | | | | | | | | | | | | | | | | | |
|  |  |  |  |  |  |  |  |  |  |  |  |  |  |  |  |  |  |  |  |  |  |  |  |  |  |
| --- | --- | --- | --- | --- | --- | --- | --- | --- | --- | --- | --- | --- | --- | --- | --- | --- | --- | --- | --- | --- | --- | --- | --- | --- | --- |
| *Note.*  Type III Sum of Squares | | | | | | | | | | | | | | | | | | | | | | | | | |

### Descriptives

| Descriptives - AMY U/L | | | | | | | | | | | |
| --- | --- | --- | --- | --- | --- | --- | --- | --- | --- | --- | --- |
| Animal group | | N | | Mean | | SD | | SE | | Coefficient of variation | |
| C |  | 10 |  | 508.2 |  | 100.11 |  | 31.66 |  | 0.197 |  |
| OV |  | 7 |  | 454.1 |  | 59.68 |  | 22.56 |  | 0.131 |  |
| AL |  | 8 |  | 477.6 |  | 63.88 |  | 22.58 |  | 0.134 |  |
| AH |  | 9 |  | 512.3 |  | 53.84 |  | 17.95 |  | 0.105 |  |
| AL-X |  | 10 |  | 468.5 |  | 71.53 |  | 22.62 |  | 0.153 |  |
| AH-X |  | 10 |  | 492.3 |  | 60.41 |  | 19.10 |  | 0.123 |  |
| X |  | 7 |  | 466.4 |  | 58.69 |  | 22.18 |  | 0.126 |  |
|  | | | | | | | | | | | |

#### Bar plots

#####

### Assumption Checks

| Test for Equality of Variances (Levene's) | | | | | | | |
| --- | --- | --- | --- | --- | --- | --- | --- |
| F | | df1 | | df2 | | p | |
| 0.610 |  | 6.000 |  | 54.00 |  | .721 |  |
|  | | | | | | | |

## TBIL

| ANOVA - TBIL umol/L | | | | | | | | | | | | | | | | | | | | | | | | | |
| --- | --- | --- | --- | --- | --- | --- | --- | --- | --- | --- | --- | --- | --- | --- | --- | --- | --- | --- | --- | --- | --- | --- | --- | --- | --- |
|  | | | | | | | | | | | | | | | | 95% CI for η² | | | |  | | 95% CI for ω² | | | |
| Homogeneity Correction | | Cases | | Sum of Squares | | df | | Mean Square | | F | | p | | η² | | Lower | | Upper | | ω² | | Lower | | Upper | |
| None |  | Animal group |  | 1.499 |  | 6.000 |  | 0.250 |  | 0.361 |  | .900 |  | 0.039 |  | 0.000 |  | 0.066 |  | 0.000 |  | 0.000 |  | 0.000 |  |
|  |  | Residuals |  | 36.684 |  | 53.000 |  | 0.692 |  |  |  |  |  |  |  |  |  |  |  |  |  |  |  |  |  |
| Welch |  | Animal group |  | 1.499 |  | 6.000 |  | 0.250 |  | 0.494 |  | .805 |  | 0.039 |  | 0.000 |  | 0.066 |  | 0.000 |  | 0.000 |  | 0.000 |  |
|  |  | Residuals |  | 36.684 |  | 21.423 |  | 1.712 |  |  |  |  |  |  |  |  |  |  |  |  |  |  |  |  |  |
|  | | | | | | | | | | | | | | | | | | | | | | | | | |
|  |  |  |  |  |  |  |  |  |  |  |  |  |  |  |  |  |  |  |  |  |  |  |  |  |  |
| --- | --- | --- | --- | --- | --- | --- | --- | --- | --- | --- | --- | --- | --- | --- | --- | --- | --- | --- | --- | --- | --- | --- | --- | --- | --- |
| *Note.*  Type III Sum of Squares | | | | | | | | | | | | | | | | | | | | | | | | | |

### Descriptives

| Descriptives - TBIL umol/L | | | | | | | | | | | |
| --- | --- | --- | --- | --- | --- | --- | --- | --- | --- | --- | --- |
| Animal group | | N | | Mean | | SD | | SE | | Coefficient of variation | |
| C |  | 10 |  | 4.500 |  | 0.527 |  | 0.167 |  | 0.117 |  |
| OV |  | 7 |  | 4.714 |  | 1.496 |  | 0.565 |  | 0.317 |  |
| AL |  | 8 |  | 5.000 |  | 0.926 |  | 0.327 |  | 0.185 |  |
| AH |  | 9 |  | 4.778 |  | 0.441 |  | 0.147 |  | 0.092 |  |
| AL-X |  | 10 |  | 4.800 |  | 0.789 |  | 0.249 |  | 0.164 |  |
| AH-X |  | 10 |  | 4.800 |  | 0.632 |  | 0.200 |  | 0.132 |  |
| X |  | 6 |  | 5.000 |  | 0.894 |  | 0.365 |  | 0.179 |  |
|  | | | | | | | | | | | |

#### Bar plots

#####

### Assumption Checks

| Test for Equality of Variances (Levene's) | | | | | | | |
| --- | --- | --- | --- | --- | --- | --- | --- |
| F | | df1 | | df2 | | p | |
| 2.405 |  | 6.000 |  | 53.00 |  | .040 |  |
|  | | | | | | | |

## BUN

| ANOVA - BUN mmol/L | | | | | | | | | | | | | | | | | | | | | | | | | |
| --- | --- | --- | --- | --- | --- | --- | --- | --- | --- | --- | --- | --- | --- | --- | --- | --- | --- | --- | --- | --- | --- | --- | --- | --- | --- |
|  | | | | | | | | | | | | | | | | 95% CI for η² | | | |  | | 95% CI for ω² | | | |
| Homogeneity Correction | | Cases | | Sum of Squares | | df | | Mean Square | | F | | p | | η² | | Lower | | Upper | | ω² | | Lower | | Upper | |
| None |  | Animal group |  | 6.643 |  | 6.000 |  | 1.107 |  | 1.772 |  | .124 |  | 0.175 |  | 0.000 |  | 0.304 |  | 0.075 |  | 0.000 |  | 0.151 |  |
|  |  | Residuals |  | 31.246 |  | 50.000 |  | 0.625 |  |  |  |  |  |  |  |  |  |  |  |  |  |  |  |  |  |
| Welch |  | Animal group |  | 6.643 |  | 6.000 |  | 1.107 |  | 2.258 |  | .081 |  | 0.175 |  | 0.000 |  | 0.304 |  | 0.075 |  | 0.000 |  | 0.151 |  |
|  |  | Residuals |  | 31.246 |  | 19.280 |  | 1.621 |  |  |  |  |  |  |  |  |  |  |  |  |  |  |  |  |  |
|  | | | | | | | | | | | | | | | | | | | | | | | | | |
|  |  |  |  |  |  |  |  |  |  |  |  |  |  |  |  |  |  |  |  |  |  |  |  |  |  |
| --- | --- | --- | --- | --- | --- | --- | --- | --- | --- | --- | --- | --- | --- | --- | --- | --- | --- | --- | --- | --- | --- | --- | --- | --- | --- |
| *Note.*  Type III Sum of Squares | | | | | | | | | | | | | | | | | | | | | | | | | |

### Descriptives

| Descriptives - BUN mmol/L | | | | | | | | | | | |
| --- | --- | --- | --- | --- | --- | --- | --- | --- | --- | --- | --- |
| Animal group | | N | | Mean | | SD | | SE | | Coefficient of variation | |
| C |  | 10 |  | 5.660 |  | 0.908 |  | 0.287 |  | 0.160 |  |
| OV |  | 7 |  | 5.786 |  | 1.051 |  | 0.397 |  | 0.182 |  |
| AL |  | 8 |  | 5.950 |  | 0.641 |  | 0.227 |  | 0.108 |  |
| AH |  | 5 |  | 6.000 |  | 0.927 |  | 0.415 |  | 0.155 |  |
| AL-X |  | 10 |  | 5.640 |  | 0.467 |  | 0.148 |  | 0.083 |  |
| AH-X |  | 10 |  | 6.470 |  | 0.572 |  | 0.181 |  | 0.088 |  |
| X |  | 7 |  | 6.486 |  | 0.997 |  | 0.377 |  | 0.154 |  |
|  | | | | | | | | | | | |

#### Bar plots

#####

### Assumption Checks

| Test for Equality of Variances (Levene's) | | | | | | | |
| --- | --- | --- | --- | --- | --- | --- | --- |
| F | | df1 | | df2 | | p | |
| 1.856 |  | 6.000 |  | 50.00 |  | .107 |  |
|  | | | | | | | |

## Ca

| ANOVA - Ca mmol/L | | | | | | | | | | | | | | | | | | | | | | | | | |
| --- | --- | --- | --- | --- | --- | --- | --- | --- | --- | --- | --- | --- | --- | --- | --- | --- | --- | --- | --- | --- | --- | --- | --- | --- | --- |
|  | | | | | | | | | | | | | | | | 95% CI for η² | | | |  | | 95% CI for ω² | | | |
| Homogeneity Correction | | Cases | | Sum of Squares | | df | | Mean Square | | F | | p | | η² | | Lower | | Upper | | ω² | | Lower | | Upper | |
| None |  | Animal group |  | 0.236 |  | 6.000 |  | 0.039 |  | 2.160 |  | .061 |  | 0.194 |  | 0.000 |  | 0.324 |  | 0.102 |  | 0.000 |  | 0.199 |  |
|  |  | Residuals |  | 0.984 |  | 54.000 |  | 0.018 |  |  |  |  |  |  |  |  |  |  |  |  |  |  |  |  |  |
| Welch |  | Animal group |  | 0.236 |  | 6.000 |  | 0.039 |  | 2.372 |  | .063 |  | 0.194 |  | 0.000 |  | 0.324 |  | 0.102 |  | 0.000 |  | 0.199 |  |
|  |  | Residuals |  | 0.984 |  | 22.647 |  | 0.043 |  |  |  |  |  |  |  |  |  |  |  |  |  |  |  |  |  |
|  | | | | | | | | | | | | | | | | | | | | | | | | | |
|  |  |  |  |  |  |  |  |  |  |  |  |  |  |  |  |  |  |  |  |  |  |  |  |  |  |
| --- | --- | --- | --- | --- | --- | --- | --- | --- | --- | --- | --- | --- | --- | --- | --- | --- | --- | --- | --- | --- | --- | --- | --- | --- | --- |
| *Note.*  Type III Sum of Squares | | | | | | | | | | | | | | | | | | | | | | | | | |

### Descriptives

| Descriptives - Ca mmol/L | | | | | | | | | | | |
| --- | --- | --- | --- | --- | --- | --- | --- | --- | --- | --- | --- |
| Animal group | | N | | Mean | | SD | | SE | | Coefficient of variation | |
| C |  | 10 |  | 2.728 |  | 0.093 |  | 0.030 |  | 0.034 |  |
| OV |  | 7 |  | 2.623 |  | 0.051 |  | 0.019 |  | 0.020 |  |
| AL |  | 8 |  | 2.659 |  | 0.136 |  | 0.048 |  | 0.051 |  |
| AH |  | 9 |  | 2.576 |  | 0.097 |  | 0.032 |  | 0.038 |  |
| AL-X |  | 10 |  | 2.671 |  | 0.092 |  | 0.029 |  | 0.034 |  |
| AH-X |  | 10 |  | 2.659 |  | 0.053 |  | 0.017 |  | 0.020 |  |
| X |  | 7 |  | 2.519 |  | 0.311 |  | 0.118 |  | 0.124 |  |
|  | | | | | | | | | | | |

#### Bar plots

#####

### Assumption Checks

| Test for Equality of Variances (Levene's) | | | | | | | |
| --- | --- | --- | --- | --- | --- | --- | --- |
| F | | df1 | | df2 | | p | |
| 2.242 |  | 6.000 |  | 54.00 |  | .053 |  |
|  | | | | | | | |

## Phosphate

| ANOVA - P mmol/L | | | | | | | | | | | | | | | | | | | | | | | | | |
| --- | --- | --- | --- | --- | --- | --- | --- | --- | --- | --- | --- | --- | --- | --- | --- | --- | --- | --- | --- | --- | --- | --- | --- | --- | --- |
|  | | | | | | | | | | | | | | | | 95% CI for η² | | | |  | | 95% CI for ω² | | | |
| Homogeneity Correction | | Cases | | Sum of Squares | | df | | Mean Square | | F | | p | | η² | | Lower | | Upper | | ω² | | Lower | | Upper | |
| None |  | Animal group |  | 0.201 |  | 6.000 |  | 0.033 |  | 0.381 |  | .888 |  | 0.041 |  | 0.000 |  | 0.071 |  | 0.000 |  | 0.000 |  | 0.000 |  |
|  |  | Residuals |  | 4.750 |  | 54.000 |  | 0.088 |  |  |  |  |  |  |  |  |  |  |  |  |  |  |  |  |  |
| Welch |  | Animal group |  | 0.201 |  | 6.000 |  | 0.033 |  | 0.272 |  | .944 |  | 0.041 |  | 0.000 |  | 0.071 |  | 0.000 |  | 0.000 |  | 0.000 |  |
|  |  | Residuals |  | 4.750 |  | 22.868 |  | 0.208 |  |  |  |  |  |  |  |  |  |  |  |  |  |  |  |  |  |
|  | | | | | | | | | | | | | | | | | | | | | | | | | |
|  |  |  |  |  |  |  |  |  |  |  |  |  |  |  |  |  |  |  |  |  |  |  |  |  |  |
| --- | --- | --- | --- | --- | --- | --- | --- | --- | --- | --- | --- | --- | --- | --- | --- | --- | --- | --- | --- | --- | --- | --- | --- | --- | --- |
| *Note.*  Type III Sum of Squares | | | | | | | | | | | | | | | | | | | | | | | | | |

### Descriptives

| Descriptives - P mmol/L | | | | | | | | | | | |
| --- | --- | --- | --- | --- | --- | --- | --- | --- | --- | --- | --- |
| Animal group | | N | | Mean | | SD | | SE | | Coefficient of variation | |
| C |  | 10 |  | 2.127 |  | 0.268 |  | 0.085 |  | 0.126 |  |
| OV |  | 7 |  | 2.109 |  | 0.244 |  | 0.092 |  | 0.116 |  |
| AL |  | 8 |  | 1.980 |  | 0.304 |  | 0.107 |  | 0.153 |  |
| AH |  | 9 |  | 1.969 |  | 0.501 |  | 0.167 |  | 0.254 |  |
| AL-X |  | 10 |  | 2.055 |  | 0.199 |  | 0.063 |  | 0.097 |  |
| AH-X |  | 10 |  | 2.075 |  | 0.213 |  | 0.067 |  | 0.103 |  |
| X |  | 7 |  | 2.100 |  | 0.235 |  | 0.089 |  | 0.112 |  |
|  | | | | | | | | | | | |

#### Bar plots

#####

### Assumption Checks

| Test for Equality of Variances (Levene's) | | | | | | | |
| --- | --- | --- | --- | --- | --- | --- | --- |
| F | | df1 | | df2 | | p | |
| 0.898 |  | 6.000 |  | 54.00 |  | .503 |  |
|  | | | | | | | |

## CRE

| ANOVA - Cre umol/L | | | | | | | | | | | | | | | | | | | | | | | | | |
| --- | --- | --- | --- | --- | --- | --- | --- | --- | --- | --- | --- | --- | --- | --- | --- | --- | --- | --- | --- | --- | --- | --- | --- | --- | --- |
|  | | | | | | | | | | | | | | | | 95% CI for η² | | | |  | | 95% CI for ω² | | | |
| Homogeneity Correction | | Cases | | Sum of Squares | | df | | Mean Square | | F | | p | | η² | | Lower | | Upper | | ω² | | Lower | | Upper | |
| None |  | Animal group |  | 2933 |  | 6.000 |  | 488.9 |  | 2.448 |  | .038 |  | 0.231 |  | 0.000 |  | 0.371 |  | 0.134 |  | 0.000 |  | 0.250 |  |
|  |  | Residuals |  | 9785 |  | 49.000 |  | 199.7 |  |  |  |  |  |  |  |  |  |  |  |  |  |  |  |  |  |
| Welch |  | Animal group |  | 2933 |  | 6.000 |  | 488.9 |  | 2.148 |  | .094 |  | 0.231 |  | 0.000 |  | 0.371 |  | 0.134 |  | 0.000 |  | 0.250 |  |
|  |  | Residuals |  | 9785 |  | 19.306 |  | 506.8 |  |  |  |  |  |  |  |  |  |  |  |  |  |  |  |  |  |
|  | | | | | | | | | | | | | | | | | | | | | | | | | |
|  |  |  |  |  |  |  |  |  |  |  |  |  |  |  |  |  |  |  |  |  |  |  |  |  |  |
| --- | --- | --- | --- | --- | --- | --- | --- | --- | --- | --- | --- | --- | --- | --- | --- | --- | --- | --- | --- | --- | --- | --- | --- | --- | --- |
| *Note.*  Type III Sum of Squares | | | | | | | | | | | | | | | | | | | | | | | | | |

### Descriptives

| Descriptives - Cre umol/L | | | | | | | | | | | |
| --- | --- | --- | --- | --- | --- | --- | --- | --- | --- | --- | --- |
| Animal group | | N | | Mean | | SD | | SE | | Coefficient of variation | |
| C |  | 10 |  | 39.70 |  | 12.401 |  | 3.922 |  | 0.312 |  |
| OV |  | 7 |  | 36.14 |  | 8.153 |  | 3.082 |  | 0.226 |  |
| AL |  | 8 |  | 38.13 |  | 6.999 |  | 2.474 |  | 0.184 |  |
| AH |  | 5 |  | 27.00 |  | 14.595 |  | 6.527 |  | 0.541 |  |
| AL-X |  | 10 |  | 45.70 |  | 16.607 |  | 5.252 |  | 0.363 |  |
| AH-X |  | 10 |  | 42.20 |  | 19.054 |  | 6.026 |  | 0.452 |  |
| X |  | 6 |  | 56.67 |  | 14.542 |  | 5.937 |  | 0.257 |  |
|  | | | | | | | | | | | |

#### Bar plots

#####

### Assumption Checks

| Test for Equality of Variances (Levene's) | | | | | | | |
| --- | --- | --- | --- | --- | --- | --- | --- |
| F | | df1 | | df2 | | p | |
| 1.421 |  | 6.000 |  | 49.00 |  | .226 |  |
|  | | | | | | | |

### Post Hoc Tests

#### Standard (HSD)

| Post Hoc Comparisons - Animal group | | | | | | | | | | | | | | | | | | | |
| --- | --- | --- | --- | --- | --- | --- | --- | --- | --- | --- | --- | --- | --- | --- | --- | --- | --- | --- | --- |
|  | | | | | | 95% CI for Mean Difference | | | |  | | | | | | | | | |
|  | |  | | Mean Difference | | Lower | | Upper | | SE | | df | | t | | ptukey | | pbonf | |
| C |  | OV |  | 3.557 |  | -17.85 |  | 24.965 |  | 6.964 |  | 49 |  | 0.511 |  | .999 |  | 1.000 |  |
|  |  | AL |  | 1.575 |  | -19.03 |  | 22.180 |  | 6.703 |  | 49 |  | 0.235 |  | 1.000 |  | 1.000 |  |
|  |  | AH |  | 12.700 |  | -11.09 |  | 36.493 |  | 7.740 |  | 49 |  | 1.641 |  | .657 |  | 1.000 |  |
|  |  | (AL-X) |  | -6.000 |  | -25.43 |  | 13.427 |  | 6.320 |  | 49 |  | -0.949 |  | .962 |  | 1.000 |  |
|  |  | (AH-X) |  | -2.500 |  | -21.93 |  | 16.927 |  | 6.320 |  | 49 |  | -0.396 |  | 1.000 |  | 1.000 |  |
|  |  | X |  | -16.967 |  | -39.40 |  | 5.466 |  | 7.297 |  | 49 |  | -2.325 |  | .253 |  | .509 |  |
| OV |  | AL |  | -1.982 |  | -24.46 |  | 20.500 |  | 7.314 |  | 49 |  | -0.271 |  | 1.000 |  | 1.000 |  |
|  |  | AH |  | 9.143 |  | -16.29 |  | 34.579 |  | 8.274 |  | 49 |  | 1.105 |  | .924 |  | 1.000 |  |
|  |  | (AL-X) |  | -9.557 |  | -30.96 |  | 11.850 |  | 6.964 |  | 49 |  | -1.372 |  | .814 |  | 1.000 |  |
|  |  | (AH-X) |  | -6.057 |  | -27.46 |  | 15.350 |  | 6.964 |  | 49 |  | -0.870 |  | .975 |  | 1.000 |  |
|  |  | X |  | -20.524 |  | -44.69 |  | 3.644 |  | 7.862 |  | 49 |  | -2.611 |  | .145 |  | .251 |  |
| AL |  | AH |  | 11.125 |  | -13.64 |  | 35.890 |  | 8.056 |  | 49 |  | 1.381 |  | .809 |  | 1.000 |  |
|  |  | (AL-X) |  | -7.575 |  | -28.18 |  | 13.030 |  | 6.703 |  | 49 |  | -1.130 |  | .916 |  | 1.000 |  |
|  |  | (AH-X) |  | -4.075 |  | -24.68 |  | 16.530 |  | 6.703 |  | 49 |  | -0.608 |  | .996 |  | 1.000 |  |
|  |  | X |  | -18.542 |  | -42.00 |  | 4.919 |  | 7.632 |  | 49 |  | -2.430 |  | .209 |  | .395 |  |
| AH |  | (AL-X) |  | -18.700 |  | -42.49 |  | 5.093 |  | 7.740 |  | 49 |  | -2.416 |  | .214 |  | .409 |  |
|  |  | (AH-X) |  | -15.200 |  | -38.99 |  | 8.593 |  | 7.740 |  | 49 |  | -1.964 |  | .450 |  | 1.000 |  |
|  |  | X |  | -29.667 |  | -55.97 |  | -3.363 |  | 8.557 |  | 49 |  | -3.467 |  | .018 | \* | .023 | \* |
| (AL-X) |  | (AH-X) |  | 3.500 |  | -15.93 |  | 22.927 |  | 6.320 |  | 49 |  | 0.554 |  | .998 |  | 1.000 |  |
|  |  | X |  | -10.967 |  | -33.40 |  | 11.466 |  | 7.297 |  | 49 |  | -1.503 |  | .742 |  | 1.000 |  |
| (AH-X) |  | X |  | -14.467 |  | -36.90 |  | 7.966 |  | 7.297 |  | 49 |  | -1.982 |  | .438 |  | 1.000 |  |
|  | | | | | | | | | | | | | | | | | | | |
|  |  |  |  |  |  |  |  |  |  |  |  |  |  |  |  |  |  |  |  |
| --- | --- | --- | --- | --- | --- | --- | --- | --- | --- | --- | --- | --- | --- | --- | --- | --- | --- | --- | --- |
| \* p < .05 | | | | | | | | | | | | | | | | | | | |
| *Note.*  P-value and confidence intervals adjusted for comparing a family of 7 estimates (confidence intervals corrected using the tukey method). | | | | | | | | | | | | | | | | | | | |

| Letter-Based Grouping - Animal group | | | |
| --- | --- | --- | --- |
| Animal group | | Letter | |
| C |  | ab |  |
| OV |  | ab |  |
| AL |  | ab |  |
| AH |  | a |  |
| AL-X |  | ab |  |
| AH-X |  | ab |  |
| X |  | b |  |
|  | | | |
|  |  |  |  |
| --- | --- | --- | --- |
| *Note.*  If two or more means share the same grouping symbol, then we cannot show them to be different, but we also did not show them to be the same. | | | |

## Glucose

| ANOVA - Glu mmol/L | | | | | | | | | | | | | | | | | | | | | | | | | |
| --- | --- | --- | --- | --- | --- | --- | --- | --- | --- | --- | --- | --- | --- | --- | --- | --- | --- | --- | --- | --- | --- | --- | --- | --- | --- |
|  | | | | | | | | | | | | | | | | 95% CI for η² | | | |  | | 95% CI for ω² | | | |
| Homogeneity Correction | | Cases | | Sum of Squares | | df | | Mean Square | | F | | p | | η² | | Lower | | Upper | | ω² | | Lower | | Upper | |
| None |  | Animal group |  | 38.71 |  | 6.000 |  | 6.451 |  | 1.446 |  | .215 |  | 0.141 |  | 0.000 |  | 0.256 |  | 0.043 |  | 0.000 |  | 0.076 |  |
|  |  | Residuals |  | 236.43 |  | 53.000 |  | 4.461 |  |  |  |  |  |  |  |  |  |  |  |  |  |  |  |  |  |
| Welch |  | Animal group |  | 38.71 |  | 6.000 |  | 6.451 |  | 1.200 |  | .342 |  | 0.141 |  | 0.000 |  | 0.256 |  | 0.043 |  | 0.000 |  | 0.076 |  |
|  |  | Residuals |  | 236.43 |  | 22.581 |  | 10.470 |  |  |  |  |  |  |  |  |  |  |  |  |  |  |  |  |  |
|  | | | | | | | | | | | | | | | | | | | | | | | | | |
|  |  |  |  |  |  |  |  |  |  |  |  |  |  |  |  |  |  |  |  |  |  |  |  |  |  |
| --- | --- | --- | --- | --- | --- | --- | --- | --- | --- | --- | --- | --- | --- | --- | --- | --- | --- | --- | --- | --- | --- | --- | --- | --- | --- |
| *Note.*  Type III Sum of Squares | | | | | | | | | | | | | | | | | | | | | | | | | |

### Descriptives

| Descriptives - Glu mmol/L | | | | | | | | | | | |
| --- | --- | --- | --- | --- | --- | --- | --- | --- | --- | --- | --- |
| Animal group | | N | | Mean | | SD | | SE | | Coefficient of variation | |
| C |  | 10 |  | 13.70 |  | 2.486 |  | 0.786 |  | 0.181 |  |
| OV |  | 7 |  | 15.40 |  | 2.946 |  | 1.113 |  | 0.191 |  |
| AL |  | 8 |  | 15.35 |  | 2.305 |  | 0.815 |  | 0.150 |  |
| AH |  | 8 |  | 15.86 |  | 1.939 |  | 0.686 |  | 0.122 |  |
| AL-X |  | 10 |  | 16.25 |  | 1.590 |  | 0.503 |  | 0.098 |  |
| AH-X |  | 10 |  | 15.68 |  | 1.874 |  | 0.593 |  | 0.120 |  |
| X |  | 7 |  | 15.19 |  | 1.345 |  | 0.508 |  | 0.089 |  |
|  | | | | | | | | | | | |

#### Bar plots

#####

### Assumption Checks

| Test for Equality of Variances (Levene's) | | | | | | | |
| --- | --- | --- | --- | --- | --- | --- | --- |
| F | | df1 | | df2 | | p | |
| 0.701 |  | 6.000 |  | 53.00 |  | .650 |  |
|  | | | | | | | |

## Sodium

| ANOVA - Na mmol/L | | | | | | | | | | | | | | | | | | | | | | | | | |
| --- | --- | --- | --- | --- | --- | --- | --- | --- | --- | --- | --- | --- | --- | --- | --- | --- | --- | --- | --- | --- | --- | --- | --- | --- | --- |
|  | | | | | | | | | | | | | | | | 95% CI for η² | | | |  | | 95% CI for ω² | | | |
| Homogeneity Correction | | Cases | | Sum of Squares | | df | | Mean Square | | F | | p | | η² | | Lower | | Upper | | ω² | | Lower | | Upper | |
| None |  | Animal group |  | 146.8 |  | 6.000 |  | 24.47 |  | 0.868 |  | .525 |  | 0.089 |  | 0.000 |  | 0.177 |  | 0.000 |  | 0.000 |  | 0.000 |  |
|  |  | Residuals |  | 1494.4 |  | 53.000 |  | 28.20 |  |  |  |  |  |  |  |  |  |  |  |  |  |  |  |  |  |
| Welch |  | Animal group |  | 146.8 |  | 6.000 |  | 24.47 |  | 0.485 |  | .813 |  | 0.089 |  | 0.000 |  | 0.177 |  | 0.000 |  | 0.000 |  | 0.000 |  |
|  |  | Residuals |  | 1494.4 |  | 22.136 |  | 67.51 |  |  |  |  |  |  |  |  |  |  |  |  |  |  |  |  |  |
|  | | | | | | | | | | | | | | | | | | | | | | | | | |
|  |  |  |  |  |  |  |  |  |  |  |  |  |  |  |  |  |  |  |  |  |  |  |  |  |  |
| --- | --- | --- | --- | --- | --- | --- | --- | --- | --- | --- | --- | --- | --- | --- | --- | --- | --- | --- | --- | --- | --- | --- | --- | --- | --- |
| *Note.*  Type III Sum of Squares | | | | | | | | | | | | | | | | | | | | | | | | | |

### Descriptives

| Descriptives - Na mmol/L | | | | | | | | | | | |
| --- | --- | --- | --- | --- | --- | --- | --- | --- | --- | --- | --- |
| Animal group | | N | | Mean | | SD | | SE | | Coefficient of variation | |
| C |  | 10 |  | 131.3 |  | 6.019 |  | 1.904 |  | 0.046 |  |
| OV |  | 7 |  | 131.6 |  | 4.429 |  | 1.674 |  | 0.034 |  |
| AL |  | 8 |  | 131.5 |  | 4.899 |  | 1.732 |  | 0.037 |  |
| AH |  | 9 |  | 127.7 |  | 7.314 |  | 2.438 |  | 0.057 |  |
| AL-X |  | 10 |  | 132.3 |  | 3.860 |  | 1.221 |  | 0.029 |  |
| AH-X |  | 10 |  | 132.5 |  | 4.743 |  | 1.500 |  | 0.036 |  |
| X |  | 6 |  | 132.0 |  | 4.858 |  | 1.983 |  | 0.037 |  |
|  | | | | | | | | | | | |

#### Bar plots

#####

### Assumption Checks

| Test for Equality of Variances (Levene's) | | | | | | | |
| --- | --- | --- | --- | --- | --- | --- | --- |
| F | | df1 | | df2 | | p | |
| 3.079 |  | 6.000 |  | 53.00 |  | .012 |  |
|  | | | | | | | |

## Potassium

| ANOVA - K mmol/L | | | | | | | | | | | | | | | | | | | | | | | | | |
| --- | --- | --- | --- | --- | --- | --- | --- | --- | --- | --- | --- | --- | --- | --- | --- | --- | --- | --- | --- | --- | --- | --- | --- | --- | --- |
|  | | | | | | | | | | | | | | | | 95% CI for η² | | | |  | | 95% CI for ω² | | | |
| Homogeneity Correction | | Cases | | Sum of Squares | | df | | Mean Square | | F | | p | | η² | | Lower | | Upper | | ω² | | Lower | | Upper | |
| None |  | Animal group |  | 0.654 |  | 6.000 |  | 0.109 |  | 0.369 |  | .895 |  | 0.040 |  | 0.000 |  | 0.069 |  | 0.000 |  | 0.000 |  | 0.000 |  |
|  |  | Residuals |  | 15.655 |  | 53.000 |  | 0.295 |  |  |  |  |  |  |  |  |  |  |  |  |  |  |  |  |  |
| Welch |  | Animal group |  | 0.654 |  | 6.000 |  | 0.109 |  | 0.429 |  | .851 |  | 0.040 |  | 0.000 |  | 0.069 |  | 0.000 |  | 0.000 |  | 0.000 |  |
|  |  | Residuals |  | 15.655 |  | 21.874 |  | 0.716 |  |  |  |  |  |  |  |  |  |  |  |  |  |  |  |  |  |
|  | | | | | | | | | | | | | | | | | | | | | | | | | |
|  |  |  |  |  |  |  |  |  |  |  |  |  |  |  |  |  |  |  |  |  |  |  |  |  |  |
| --- | --- | --- | --- | --- | --- | --- | --- | --- | --- | --- | --- | --- | --- | --- | --- | --- | --- | --- | --- | --- | --- | --- | --- | --- | --- |
| *Note.*  Type III Sum of Squares | | | | | | | | | | | | | | | | | | | | | | | | | |

### Descriptives

| Descriptives - K mmol/L | | | | | | | | | | | |
| --- | --- | --- | --- | --- | --- | --- | --- | --- | --- | --- | --- |
| Animal group | | N | | Mean | | SD | | SE | | Coefficient of variation | |
| C |  | 10 |  | 4.520 |  | 0.391 |  | 0.124 |  | 0.087 |  |
| OV |  | 7 |  | 4.500 |  | 0.447 |  | 0.169 |  | 0.099 |  |
| AL |  | 8 |  | 4.588 |  | 0.314 |  | 0.111 |  | 0.068 |  |
| AH |  | 9 |  | 4.700 |  | 0.642 |  | 0.214 |  | 0.137 |  |
| AL-X |  | 10 |  | 4.670 |  | 0.574 |  | 0.181 |  | 0.123 |  |
| AH-X |  | 10 |  | 4.810 |  | 0.495 |  | 0.157 |  | 0.103 |  |
| X |  | 6 |  | 4.700 |  | 0.885 |  | 0.361 |  | 0.188 |  |
|  | | | | | | | | | | | |

#### Bar plots

#####

### Assumption Checks

| Test for Equality of Variances (Levene's) | | | | | | | |
| --- | --- | --- | --- | --- | --- | --- | --- |
| F | | df1 | | df2 | | p | |
| 1.354 |  | 6.000 |  | 53.00 |  | .250 |  |
|  | | | | | | | |

## Total proteins

| ANOVA - TP g/L | | | | | | | | | | | | | | | | | | | | | | | | | |
| --- | --- | --- | --- | --- | --- | --- | --- | --- | --- | --- | --- | --- | --- | --- | --- | --- | --- | --- | --- | --- | --- | --- | --- | --- | --- |
|  | | | | | | | | | | | | | | | | 95% CI for η² | | | |  | | 95% CI for ω² | | | |
| Homogeneity Correction | | Cases | | Sum of Squares | | df | | Mean Square | | F | | p | | η² | | Lower | | Upper | | ω² | | Lower | | Upper | |
| None |  | Animal group |  | 233.6 |  | 6.000 |  | 38.93 |  | 3.077 |  | .012 |  | 0.255 |  | 0.018 |  | 0.393 |  | 0.170 |  | 0.000 |  | 0.294 |  |
|  |  | Residuals |  | 683.3 |  | 54.000 |  | 12.65 |  |  |  |  |  |  |  |  |  |  |  |  |  |  |  |  |  |
| Welch |  | Animal group |  | 233.6 |  | 6.000 |  | 38.93 |  | 5.216 |  | .002 |  | 0.255 |  | 0.018 |  | 0.393 |  | 0.170 |  | 0.000 |  | 0.294 |  |
|  |  | Residuals |  | 683.3 |  | 22.485 |  | 30.39 |  |  |  |  |  |  |  |  |  |  |  |  |  |  |  |  |  |
|  | | | | | | | | | | | | | | | | | | | | | | | | | |
|  |  |  |  |  |  |  |  |  |  |  |  |  |  |  |  |  |  |  |  |  |  |  |  |  |  |
| --- | --- | --- | --- | --- | --- | --- | --- | --- | --- | --- | --- | --- | --- | --- | --- | --- | --- | --- | --- | --- | --- | --- | --- | --- | --- |
| *Note.*  Type III Sum of Squares | | | | | | | | | | | | | | | | | | | | | | | | | |

### Descriptives

| Descriptives - TP g/L | | | | | | | | | | | |
| --- | --- | --- | --- | --- | --- | --- | --- | --- | --- | --- | --- |
| Animal group | | N | | Mean | | SD | | SE | | Coefficient of variation | |
| C |  | 10 |  | 64.60 |  | 2.633 |  | 0.833 |  | 0.041 |  |
| OV |  | 7 |  | 58.57 |  | 3.505 |  | 1.325 |  | 0.060 |  |
| AL |  | 8 |  | 60.75 |  | 3.732 |  | 1.319 |  | 0.061 |  |
| AH |  | 9 |  | 60.67 |  | 4.444 |  | 1.481 |  | 0.073 |  |
| AL-X |  | 10 |  | 61.60 |  | 4.477 |  | 1.416 |  | 0.073 |  |
| AH-X |  | 10 |  | 58.60 |  | 2.011 |  | 0.636 |  | 0.034 |  |
| X |  | 7 |  | 61.14 |  | 3.532 |  | 1.335 |  | 0.058 |  |
|  | | | | | | | | | | | |

#### Bar plots

#####

### Assumption Checks

| Test for Equality of Variances (Levene's) | | | | | | | |
| --- | --- | --- | --- | --- | --- | --- | --- |
| F | | df1 | | df2 | | p | |
| 0.429 |  | 6.000 |  | 54.00 |  | .856 |  |
|  | | | | | | | |

### Post Hoc Tests

#### Standard (HSD)

| Post Hoc Comparisons - Animal group | | | | | | | | | | | | | | | | | | | |
| --- | --- | --- | --- | --- | --- | --- | --- | --- | --- | --- | --- | --- | --- | --- | --- | --- | --- | --- | --- |
|  | | | | | | 95% CI for Mean Difference | | | |  | | | | | | | | | |
|  | |  | | Mean Difference | | Lower | | Upper | | SE | | df | | t | | ptukey | | pbonf | |
| C |  | OV |  | 6.029 |  | 0.661 |  | 11.396 |  | 1.753 |  | 54 |  | 3.439 |  | .018 | \* | .024 | \* |
|  |  | AL |  | 3.850 |  | -1.317 |  | 9.017 |  | 1.687 |  | 54 |  | 2.282 |  | .272 |  | .556 |  |
|  |  | AH |  | 3.933 |  | -1.071 |  | 8.938 |  | 1.634 |  | 54 |  | 2.407 |  | .216 |  | .411 |  |
|  |  | (AL-X) |  | 3.000 |  | -1.871 |  | 7.871 |  | 1.591 |  | 54 |  | 1.886 |  | .498 |  | 1.000 |  |
|  |  | (AH-X) |  | 6.000 |  | 1.129 |  | 10.871 |  | 1.591 |  | 54 |  | 3.772 |  | .007 | \*\* | .008 | \*\* |
|  |  | X |  | 3.457 |  | -1.911 |  | 8.825 |  | 1.753 |  | 54 |  | 1.972 |  | .444 |  | 1.000 |  |
| OV |  | AL |  | -2.179 |  | -7.816 |  | 3.459 |  | 1.841 |  | 54 |  | -1.183 |  | .897 |  | 1.000 |  |
|  |  | AH |  | -2.095 |  | -7.585 |  | 3.394 |  | 1.793 |  | 54 |  | -1.169 |  | .903 |  | 1.000 |  |
|  |  | (AL-X) |  | -3.029 |  | -8.396 |  | 2.339 |  | 1.753 |  | 54 |  | -1.728 |  | .601 |  | 1.000 |  |
|  |  | (AH-X) |  | -0.029 |  | -5.396 |  | 5.339 |  | 1.753 |  | 54 |  | -0.016 |  | 1.000 |  | 1.000 |  |
|  |  | X |  | -2.571 |  | -8.394 |  | 3.251 |  | 1.901 |  | 54 |  | -1.352 |  | .824 |  | 1.000 |  |
| AL |  | AH |  | 0.083 |  | -5.209 |  | 5.376 |  | 1.728 |  | 54 |  | 0.048 |  | 1.000 |  | 1.000 |  |
|  |  | (AL-X) |  | -0.850 |  | -6.017 |  | 4.317 |  | 1.687 |  | 54 |  | -0.504 |  | .999 |  | 1.000 |  |
|  |  | (AH-X) |  | 2.150 |  | -3.017 |  | 7.317 |  | 1.687 |  | 54 |  | 1.274 |  | .861 |  | 1.000 |  |
|  |  | X |  | -0.393 |  | -6.030 |  | 5.245 |  | 1.841 |  | 54 |  | -0.213 |  | 1.000 |  | 1.000 |  |
| AH |  | (AL-X) |  | -0.933 |  | -5.938 |  | 4.071 |  | 1.634 |  | 54 |  | -0.571 |  | .997 |  | 1.000 |  |
|  |  | (AH-X) |  | 2.067 |  | -2.938 |  | 7.071 |  | 1.634 |  | 54 |  | 1.264 |  | .865 |  | 1.000 |  |
|  |  | X |  | -0.476 |  | -5.965 |  | 5.013 |  | 1.793 |  | 54 |  | -0.266 |  | 1.000 |  | 1.000 |  |
| (AL-X) |  | (AH-X) |  | 3.000 |  | -1.871 |  | 7.871 |  | 1.591 |  | 54 |  | 1.886 |  | .498 |  | 1.000 |  |
|  |  | X |  | 0.457 |  | -4.911 |  | 5.825 |  | 1.753 |  | 54 |  | 0.261 |  | 1.000 |  | 1.000 |  |
| (AH-X) |  | X |  | -2.543 |  | -7.911 |  | 2.825 |  | 1.753 |  | 54 |  | -1.451 |  | .772 |  | 1.000 |  |
|  | | | | | | | | | | | | | | | | | | | |
|  |  |  |  |  |  |  |  |  |  |  |  |  |  |  |  |  |  |  |  |
| --- | --- | --- | --- | --- | --- | --- | --- | --- | --- | --- | --- | --- | --- | --- | --- | --- | --- | --- | --- |
| \* p < .05, \*\* p < .01 | | | | | | | | | | | | | | | | | | | |
| *Note.*  P-value and confidence intervals adjusted for comparing a family of 7 estimates (confidence intervals corrected using the tukey method). | | | | | | | | | | | | | | | | | | | |

| Letter-Based Grouping - Animal group | | | |
| --- | --- | --- | --- |
| Animal group | | Letter | |
| C |  | b |  |
| OV |  | a |  |
| AL |  | ab |  |
| AH |  | ab |  |
| AL-X |  | ab |  |
| AH-X |  | a |  |
| X |  | ab |  |
|  | | | |
|  |  |  |  |
| --- | --- | --- | --- |
| *Note.*  If two or more means share the same grouping symbol, then we cannot show them to be different, but we also did not show them to be the same. | | | |

## Globulins

| ANOVA - Glob g/L | | | | | | | | | | | | | | | | | | | | | | | | | |
| --- | --- | --- | --- | --- | --- | --- | --- | --- | --- | --- | --- | --- | --- | --- | --- | --- | --- | --- | --- | --- | --- | --- | --- | --- | --- |
|  | | | | | | | | | | | | | | | | 95% CI for η² | | | |  | | 95% CI for ω² | | | |
| Homogeneity Correction | | Cases | | Sum of Squares | | df | | Mean Square | | F | | p | | η² | | Lower | | Upper | | ω² | | Lower | | Upper | |
| None |  | Animal group |  | 59.50 |  | 6.000 |  | 9.917 |  | 1.303 |  | .272 |  | 0.129 |  | 0.000 |  | 0.239 |  | 0.029 |  | 0.000 |  | 0.032 |  |
|  |  | Residuals |  | 403.48 |  | 53.000 |  | 7.613 |  |  |  |  |  |  |  |  |  |  |  |  |  |  |  |  |  |
| Welch |  | Animal group |  | 59.50 |  | 6.000 |  | 9.917 |  | 2.340 |  | .067 |  | 0.129 |  | 0.000 |  | 0.239 |  | 0.029 |  | 0.000 |  | 0.032 |  |
|  |  | Residuals |  | 403.48 |  | 21.998 |  | 18.342 |  |  |  |  |  |  |  |  |  |  |  |  |  |  |  |  |  |
|  | | | | | | | | | | | | | | | | | | | | | | | | | |
|  |  |  |  |  |  |  |  |  |  |  |  |  |  |  |  |  |  |  |  |  |  |  |  |  |  |
| --- | --- | --- | --- | --- | --- | --- | --- | --- | --- | --- | --- | --- | --- | --- | --- | --- | --- | --- | --- | --- | --- | --- | --- | --- | --- |
| *Note.*  Type III Sum of Squares | | | | | | | | | | | | | | | | | | | | | | | | | |

### Descriptives

| Descriptives - Glob g/L | | | | | | | | | | | |
| --- | --- | --- | --- | --- | --- | --- | --- | --- | --- | --- | --- |
| Animal group | | N | | Mean | | SD | | SE | | Coefficient of variation | |
| C |  | 10 |  | 12.00 |  | 3.266 |  | 1.033 |  | 0.272 |  |
| OV |  | 7 |  | 12.29 |  | 4.716 |  | 1.782 |  | 0.384 |  |
| AL |  | 8 |  | 14.00 |  | 2.268 |  | 0.802 |  | 0.162 |  |
| AH |  | 9 |  | 10.44 |  | 1.424 |  | 0.475 |  | 0.136 |  |
| AL-X |  | 10 |  | 12.30 |  | 2.312 |  | 0.731 |  | 0.188 |  |
| AH-X |  | 10 |  | 11.40 |  | 2.503 |  | 0.792 |  | 0.220 |  |
| X |  | 6 |  | 11.67 |  | 1.862 |  | 0.760 |  | 0.160 |  |
|  | | | | | | | | | | | |

#### Bar plots

#####

### Assumption Checks

| Test for Equality of Variances (Levene's) | | | | | | | |
| --- | --- | --- | --- | --- | --- | --- | --- |
| F | | df1 | | df2 | | p | |
| 3.384 |  | 6.000 |  | 53.00 |  | .007 |  |
|  | | | | | | | |

## WBC

| ANOVA - WBC 10^9/l | | | | | | | | | | | | | | | | | | | | | | | | | |
| --- | --- | --- | --- | --- | --- | --- | --- | --- | --- | --- | --- | --- | --- | --- | --- | --- | --- | --- | --- | --- | --- | --- | --- | --- | --- |
|  | | | | | | | | | | | | | | | | 95% CI for η² | | | |  | | 95% CI for ω² | | | |
| Homogeneity Correction | | Cases | | Sum of Squares | | df | | Mean Square | | F | | p | | η² | | Lower | | Upper | | ω² | | Lower | | Upper | |
| None |  | Animal group |  | 33.20 |  | 6.000 |  | 5.534 |  | 2.751 |  | .021 |  | 0.237 |  | 0.004 |  | 0.375 |  | 0.149 |  | 0.000 |  | 0.268 |  |
|  |  | Residuals |  | 106.61 |  | 53.000 |  | 2.011 |  |  |  |  |  |  |  |  |  |  |  |  |  |  |  |  |  |
| Welch |  | Animal group |  | 33.20 |  | 6.000 |  | 5.534 |  | 4.323 |  | .005 |  | 0.237 |  | 0.004 |  | 0.375 |  | 0.149 |  | 0.000 |  | 0.268 |  |
|  |  | Residuals |  | 106.61 |  | 22.065 |  | 4.831 |  |  |  |  |  |  |  |  |  |  |  |  |  |  |  |  |  |
|  | | | | | | | | | | | | | | | | | | | | | | | | | |
|  |  |  |  |  |  |  |  |  |  |  |  |  |  |  |  |  |  |  |  |  |  |  |  |  |  |
| --- | --- | --- | --- | --- | --- | --- | --- | --- | --- | --- | --- | --- | --- | --- | --- | --- | --- | --- | --- | --- | --- | --- | --- | --- | --- |
| *Note.*  Type III Sum of Squares | | | | | | | | | | | | | | | | | | | | | | | | | |

### Descriptives

| Descriptives - WBC 10^9/l | | | | | | | | | | | |
| --- | --- | --- | --- | --- | --- | --- | --- | --- | --- | --- | --- |
| Animal group | | N | | Mean | | SD | | SE | | Coefficient of variation | |
| C |  | 10 |  | 3.520 |  | 0.899 |  | 0.284 |  | 0.255 |  |
| OV |  | 7 |  | 5.300 |  | 2.792 |  | 1.055 |  | 0.527 |  |
| AL |  | 8 |  | 4.313 |  | 1.137 |  | 0.402 |  | 0.264 |  |
| AH |  | 9 |  | 5.222 |  | 1.213 |  | 0.404 |  | 0.232 |  |
| AL-X |  | 10 |  | 3.920 |  | 1.166 |  | 0.369 |  | 0.297 |  |
| AH-X |  | 10 |  | 5.220 |  | 1.311 |  | 0.415 |  | 0.251 |  |
| X |  | 6 |  | 5.567 |  | 0.896 |  | 0.366 |  | 0.161 |  |
|  | | | | | | | | | | | |

#### Bar plots

#####

### Assumption Checks

| Test for Equality of Variances (Levene's) | | | | | | | |
| --- | --- | --- | --- | --- | --- | --- | --- |
| F | | df1 | | df2 | | p | |
| 1.268 |  | 6.000 |  | 53.00 |  | .288 |  |
|  | | | | | | | |

### Post Hoc Tests

#### Standard (HSD)

| Post Hoc Comparisons - Animal group | | | | | | | | | | | | | | | | | | | |
| --- | --- | --- | --- | --- | --- | --- | --- | --- | --- | --- | --- | --- | --- | --- | --- | --- | --- | --- | --- |
|  | | | | | | 95% CI for Mean Difference | | | |  | | | | | | | | | |
|  | |  | | Mean Difference | | Lower | | Upper | | SE | | df | | t | | ptukey | | pbonf | |
| C |  | OV |  | -1.780 |  | -3.922 |  | 0.362 |  | 0.699 |  | 53 |  | -2.547 |  | .164 |  | .290 |  |
|  |  | AL |  | -0.793 |  | -2.854 |  | 1.269 |  | 0.673 |  | 53 |  | -1.178 |  | .899 |  | 1.000 |  |
|  |  | AH |  | -1.702 |  | -3.699 |  | 0.295 |  | 0.652 |  | 53 |  | -2.612 |  | .143 |  | .245 |  |
|  |  | (AL-X) |  | -0.400 |  | -2.344 |  | 1.544 |  | 0.634 |  | 53 |  | -0.631 |  | .995 |  | 1.000 |  |
|  |  | (AH-X) |  | -1.700 |  | -3.644 |  | 0.244 |  | 0.634 |  | 53 |  | -2.680 |  | .124 |  | .205 |  |
|  |  | X |  | -2.047 |  | -4.291 |  | 0.198 |  | 0.732 |  | 53 |  | -2.795 |  | .096 |  | .152 |  |
| OV |  | AL |  | 0.988 |  | -1.262 |  | 3.237 |  | 0.734 |  | 53 |  | 1.345 |  | .827 |  | 1.000 |  |
|  |  | AH |  | 0.078 |  | -2.112 |  | 2.268 |  | 0.715 |  | 53 |  | 0.109 |  | 1.000 |  | 1.000 |  |
|  |  | (AL-X) |  | 1.380 |  | -0.762 |  | 3.522 |  | 0.699 |  | 53 |  | 1.974 |  | .443 |  | 1.000 |  |
|  |  | (AH-X) |  | 0.080 |  | -2.062 |  | 2.222 |  | 0.699 |  | 53 |  | 0.114 |  | 1.000 |  | 1.000 |  |
|  |  | X |  | -0.267 |  | -2.685 |  | 2.151 |  | 0.789 |  | 53 |  | -0.338 |  | 1.000 |  | 1.000 |  |
| AL |  | AH |  | -0.910 |  | -3.022 |  | 1.202 |  | 0.689 |  | 53 |  | -1.320 |  | .840 |  | 1.000 |  |
|  |  | (AL-X) |  | 0.393 |  | -1.669 |  | 2.454 |  | 0.673 |  | 53 |  | 0.583 |  | .997 |  | 1.000 |  |
|  |  | (AH-X) |  | -0.908 |  | -2.969 |  | 1.154 |  | 0.673 |  | 53 |  | -1.349 |  | .826 |  | 1.000 |  |
|  |  | X |  | -1.254 |  | -3.601 |  | 1.093 |  | 0.766 |  | 53 |  | -1.637 |  | .659 |  | 1.000 |  |
| AH |  | (AL-X) |  | 1.302 |  | -0.695 |  | 3.299 |  | 0.652 |  | 53 |  | 1.998 |  | .428 |  | 1.000 |  |
|  |  | (AH-X) |  | 0.002 |  | -1.995 |  | 1.999 |  | 0.652 |  | 53 |  | 0.003 |  | 1.000 |  | 1.000 |  |
|  |  | X |  | -0.344 |  | -2.635 |  | 1.946 |  | 0.747 |  | 53 |  | -0.461 |  | .999 |  | 1.000 |  |
| (AL-X) |  | (AH-X) |  | -1.300 |  | -3.244 |  | 0.644 |  | 0.634 |  | 53 |  | -2.050 |  | .397 |  | .953 |  |
|  |  | X |  | -1.647 |  | -3.891 |  | 0.598 |  | 0.732 |  | 53 |  | -2.248 |  | .288 |  | .603 |  |
| (AH-X) |  | X |  | -0.347 |  | -2.591 |  | 1.898 |  | 0.732 |  | 53 |  | -0.473 |  | .999 |  | 1.000 |  |
|  | | | | | | | | | | | | | | | | | | | |
|  |  |  |  |  |  |  |  |  |  |  |  |  |  |  |  |  |  |  |  |
| --- | --- | --- | --- | --- | --- | --- | --- | --- | --- | --- | --- | --- | --- | --- | --- | --- | --- | --- | --- |
| *Note.*  P-value and confidence intervals adjusted for comparing a family of 7 estimates (confidence intervals corrected using the tukey method). | | | | | | | | | | | | | | | | | | | |

| Letter-Based Grouping - Animal group | | | |
| --- | --- | --- | --- |
| Animal group | | Letter | |
| C |  | a |  |
| OV |  | a |  |
| AL |  | a |  |
| AH |  | a |  |
| AL-X |  | a |  |
| AH-X |  | a |  |
| X |  | a |  |
|  | | | |
|  |  |  |  |
| --- | --- | --- | --- |
| *Note.*  If two or more means share the same grouping symbol, then we cannot show them to be different, but we also did not show them to be the same. | | | |

## RBC

| ANOVA - RBC 10^12/l | | | | | | | | | | | | | | | | | | | | | | | | | |
| --- | --- | --- | --- | --- | --- | --- | --- | --- | --- | --- | --- | --- | --- | --- | --- | --- | --- | --- | --- | --- | --- | --- | --- | --- | --- |
|  | | | | | | | | | | | | | | | | 95% CI for η² | | | |  | | 95% CI for ω² | | | |
| Homogeneity Correction | | Cases | | Sum of Squares | | df | | Mean Square | | F | | p | | η² | | Lower | | Upper | | ω² | | Lower | | Upper | |
| None |  | Animal group |  | 8.475 |  | 6.000 |  | 1.412 |  | 4.092 |  | .002 |  | 0.321 |  | 0.067 |  | 0.463 |  | 0.239 |  | 0.003 |  | 0.378 |  |
|  |  | Residuals |  | 17.951 |  | 52.000 |  | 0.345 |  |  |  |  |  |  |  |  |  |  |  |  |  |  |  |  |  |
| Welch |  | Animal group |  | 8.475 |  | 6.000 |  | 1.412 |  | 10.027 |  | < .001 |  | 0.321 |  | 0.067 |  | 0.463 |  | 0.239 |  | 0.003 |  | 0.378 |  |
|  |  | Residuals |  | 17.951 |  | 21.065 |  | 0.852 |  |  |  |  |  |  |  |  |  |  |  |  |  |  |  |  |  |
|  | | | | | | | | | | | | | | | | | | | | | | | | | |
|  |  |  |  |  |  |  |  |  |  |  |  |  |  |  |  |  |  |  |  |  |  |  |  |  |  |
| --- | --- | --- | --- | --- | --- | --- | --- | --- | --- | --- | --- | --- | --- | --- | --- | --- | --- | --- | --- | --- | --- | --- | --- | --- | --- |
| *Note.*  Type III Sum of Squares | | | | | | | | | | | | | | | | | | | | | | | | | |

### Descriptives

| Descriptives - RBC 10^12/l | | | | | | | | | | | |
| --- | --- | --- | --- | --- | --- | --- | --- | --- | --- | --- | --- |
| Animal group | | N | | Mean | | SD | | SE | | Coefficient of variation | |
| C |  | 10 |  | 6.956 |  | 0.258 |  | 0.082 |  | 0.037 |  |
| OV |  | 7 |  | 7.819 |  | 0.515 |  | 0.195 |  | 0.066 |  |
| AL |  | 8 |  | 7.791 |  | 0.813 |  | 0.288 |  | 0.104 |  |
| AH |  | 9 |  | 7.268 |  | 0.631 |  | 0.210 |  | 0.087 |  |
| AL-X |  | 9 |  | 7.986 |  | 0.321 |  | 0.107 |  | 0.040 |  |
| AH-X |  | 10 |  | 7.322 |  | 0.812 |  | 0.257 |  | 0.111 |  |
| X |  | 6 |  | 7.020 |  | 0.488 |  | 0.199 |  | 0.070 |  |
|  | | | | | | | | | | | |

#### Bar plots

#####

### Assumption Checks

| Test for Equality of Variances (Levene's) | | | | | | | |
| --- | --- | --- | --- | --- | --- | --- | --- |
| F | | df1 | | df2 | | p | |
| 3.290 |  | 6.000 |  | 52.00 |  | .008 |  |
|  | | | | | | | |

### Post Hoc Tests

#### Standard (HSD)

| Post Hoc Comparisons - Animal group | | | | | | | | | | | | | | | | | | | |
| --- | --- | --- | --- | --- | --- | --- | --- | --- | --- | --- | --- | --- | --- | --- | --- | --- | --- | --- | --- |
|  | | | | | | 95% CI for Mean Difference | | | |  | | | | | | | | | |
|  | |  | | Mean Difference | | Lower | | Upper | | SE | | df | | t | | ptukey | | pbonf | |
| C |  | OV |  | -0.863 |  | -1.750 |  | 0.025 |  | 0.290 |  | 52 |  | -2.979 |  | .062 |  | .092 |  |
|  |  | AL |  | -0.835 |  | -1.690 |  | 0.019 |  | 0.279 |  | 52 |  | -2.997 |  | .059 |  | .088 |  |
|  |  | AH |  | -0.312 |  | -1.140 |  | 0.516 |  | 0.270 |  | 52 |  | -1.155 |  | .907 |  | 1.000 |  |
|  |  | (AL-X) |  | -1.030 |  | -1.857 |  | -0.202 |  | 0.270 |  | 52 |  | -3.814 |  | .006 | \*\* | .008 | \*\* |
|  |  | (AH-X) |  | -0.366 |  | -1.172 |  | 0.440 |  | 0.263 |  | 52 |  | -1.393 |  | .803 |  | 1.000 |  |
|  |  | X |  | -0.064 |  | -0.994 |  | 0.866 |  | 0.303 |  | 52 |  | -0.211 |  | 1.000 |  | 1.000 |  |
| OV |  | AL |  | 0.027 |  | -0.905 |  | 0.960 |  | 0.304 |  | 52 |  | 0.090 |  | 1.000 |  | 1.000 |  |
|  |  | AH |  | 0.551 |  | -0.357 |  | 1.459 |  | 0.296 |  | 52 |  | 1.860 |  | .515 |  | 1.000 |  |
|  |  | (AL-X) |  | -0.167 |  | -1.075 |  | 0.741 |  | 0.296 |  | 52 |  | -0.564 |  | .998 |  | 1.000 |  |
|  |  | (AH-X) |  | 0.497 |  | -0.391 |  | 1.384 |  | 0.290 |  | 52 |  | 1.715 |  | .609 |  | 1.000 |  |
|  |  | X |  | 0.799 |  | -0.204 |  | 1.801 |  | 0.327 |  | 52 |  | 2.443 |  | .202 |  | .378 |  |
| AL |  | AH |  | 0.523 |  | -0.352 |  | 1.399 |  | 0.285 |  | 52 |  | 1.834 |  | .532 |  | 1.000 |  |
|  |  | (AL-X) |  | -0.194 |  | -1.070 |  | 0.681 |  | 0.285 |  | 52 |  | -0.681 |  | .993 |  | 1.000 |  |
|  |  | (AH-X) |  | 0.469 |  | -0.385 |  | 1.324 |  | 0.279 |  | 52 |  | 1.684 |  | .630 |  | 1.000 |  |
|  |  | X |  | 0.771 |  | -0.202 |  | 1.744 |  | 0.317 |  | 52 |  | 2.431 |  | .207 |  | .390 |  |
| AH |  | (AL-X) |  | -0.718 |  | -1.567 |  | 0.132 |  | 0.277 |  | 52 |  | -2.592 |  | .150 |  | .260 |  |
|  |  | (AH-X) |  | -0.054 |  | -0.882 |  | 0.774 |  | 0.270 |  | 52 |  | -0.201 |  | 1.000 |  | 1.000 |  |
|  |  | X |  | 0.248 |  | -0.702 |  | 1.197 |  | 0.310 |  | 52 |  | 0.800 |  | .984 |  | 1.000 |  |
| (AL-X) |  | (AH-X) |  | 0.664 |  | -0.164 |  | 1.491 |  | 0.270 |  | 52 |  | 2.458 |  | .196 |  | .364 |  |
|  |  | X |  | 0.966 |  | 0.016 |  | 1.915 |  | 0.310 |  | 52 |  | 3.118 |  | .044 | \* | .062 |  |
| (AH-X) |  | X |  | 0.302 |  | -0.628 |  | 1.232 |  | 0.303 |  | 52 |  | 0.995 |  | .953 |  | 1.000 |  |
|  | | | | | | | | | | | | | | | | | | | |
|  |  |  |  |  |  |  |  |  |  |  |  |  |  |  |  |  |  |  |  |
| --- | --- | --- | --- | --- | --- | --- | --- | --- | --- | --- | --- | --- | --- | --- | --- | --- | --- | --- | --- |
| \* p < .05, \*\* p < .01 | | | | | | | | | | | | | | | | | | | |
| *Note.*  P-value and confidence intervals adjusted for comparing a family of 7 estimates (confidence intervals corrected using the tukey method). | | | | | | | | | | | | | | | | | | | |

| Letter-Based Grouping - Animal group | | | |
| --- | --- | --- | --- |
| Animal group | | Letter | |
| C |  | a |  |
| OV |  | ab |  |
| AL |  | ab |  |
| AH |  | ab |  |
| AL-X |  | b |  |
| AH-X |  | ab |  |
| X |  | a |  |
|  | | | |
|  |  |  |  |
| --- | --- | --- | --- |
| *Note.*  If two or more means share the same grouping symbol, then we cannot show them to be different, but we also did not show them to be the same. | | | |

## HGB

| ANOVA - HGB g/l | | | | | | | | | | | | | | | | | | | | | | | | | |
| --- | --- | --- | --- | --- | --- | --- | --- | --- | --- | --- | --- | --- | --- | --- | --- | --- | --- | --- | --- | --- | --- | --- | --- | --- | --- |
|  | | | | | | | | | | | | | | | | 95% CI for η² | | | |  | | 95% CI for ω² | | | |
| Homogeneity Correction | | Cases | | Sum of Squares | | df | | Mean Square | | F | | p | | η² | | Lower | | Upper | | ω² | | Lower | | Upper | |
| None |  | Animal group |  | 4016 |  | 6.000 |  | 669.3 |  | 4.138 |  | .002 |  | 0.319 |  | 0.068 |  | 0.460 |  | 0.239 |  | 0.005 |  | 0.377 |  |
|  |  | Residuals |  | 8573 |  | 53.000 |  | 161.8 |  |  |  |  |  |  |  |  |  |  |  |  |  |  |  |  |  |
| Welch |  | Animal group |  | 4016 |  | 6.000 |  | 669.3 |  | 7.504 |  | < .001 |  | 0.319 |  | 0.068 |  | 0.460 |  | 0.239 |  | 0.005 |  | 0.377 |  |
|  |  | Residuals |  | 8573 |  | 21.325 |  | 402.0 |  |  |  |  |  |  |  |  |  |  |  |  |  |  |  |  |  |
|  | | | | | | | | | | | | | | | | | | | | | | | | | |
|  |  |  |  |  |  |  |  |  |  |  |  |  |  |  |  |  |  |  |  |  |  |  |  |  |  |
| --- | --- | --- | --- | --- | --- | --- | --- | --- | --- | --- | --- | --- | --- | --- | --- | --- | --- | --- | --- | --- | --- | --- | --- | --- | --- |
| *Note.*  Type III Sum of Squares | | | | | | | | | | | | | | | | | | | | | | | | | |

### Descriptives

| Descriptives - HGB g/l | | | | | | | | | | | |
| --- | --- | --- | --- | --- | --- | --- | --- | --- | --- | --- | --- |
| Animal group | | N | | Mean | | SD | | SE | | Coefficient of variation | |
| C |  | 10 |  | 152.2 |  | 5.203 |  | 1.645 |  | 0.034 |  |
| OV |  | 7 |  | 170.1 |  | 11.335 |  | 4.284 |  | 0.067 |  |
| AL |  | 8 |  | 175.9 |  | 17.868 |  | 6.317 |  | 0.102 |  |
| AH |  | 9 |  | 167.9 |  | 10.349 |  | 3.450 |  | 0.062 |  |
| AL-X |  | 10 |  | 176.7 |  | 17.733 |  | 5.608 |  | 0.100 |  |
| AH-X |  | 10 |  | 166.8 |  | 11.612 |  | 3.672 |  | 0.070 |  |
| X |  | 6 |  | 161.5 |  | 9.203 |  | 3.757 |  | 0.057 |  |
|  | | | | | | | | | | | |

#### Bar plots

#####

### Assumption Checks

| Test for Equality of Variances (Levene's) | | | | | | | |
| --- | --- | --- | --- | --- | --- | --- | --- |
| F | | df1 | | df2 | | p | |
| 2.553 |  | 6.000 |  | 53.00 |  | .030 |  |
|  | | | | | | | |

### Post Hoc Tests

#### Standard (HSD)

| Post Hoc Comparisons - Animal group | | | | | | | | | | | | | | | | | | | |
| --- | --- | --- | --- | --- | --- | --- | --- | --- | --- | --- | --- | --- | --- | --- | --- | --- | --- | --- | --- |
|  | | | | | | 95% CI for Mean Difference | | | |  | | | | | | | | | |
|  | |  | | Mean Difference | | Lower | | Upper | | SE | | df | | t | | ptukey | | pbonf | |
| C |  | OV |  | -17.943 |  | -37.150 |  | 1.264 |  | 6.268 |  | 53 |  | -2.863 |  | .082 |  | .126 |  |
|  |  | AL |  | -23.675 |  | -42.162 |  | -5.188 |  | 6.033 |  | 53 |  | -3.924 |  | .004 | \*\* | .005 | \*\* |
|  |  | AH |  | -15.689 |  | -33.596 |  | 2.219 |  | 5.844 |  | 53 |  | -2.685 |  | .122 |  | .203 |  |
|  |  | (AL-X) |  | -24.500 |  | -41.930 |  | -7.070 |  | 5.688 |  | 53 |  | -4.307 |  | .001 | \*\* | .002 | \*\* |
|  |  | (AH-X) |  | -14.600 |  | -32.030 |  | 2.830 |  | 5.688 |  | 53 |  | -2.567 |  | .157 |  | .276 |  |
|  |  | X |  | -9.300 |  | -29.426 |  | 10.826 |  | 6.568 |  | 53 |  | -1.416 |  | .791 |  | 1.000 |  |
| OV |  | AL |  | -5.732 |  | -25.903 |  | 14.439 |  | 6.582 |  | 53 |  | -0.871 |  | .975 |  | 1.000 |  |
|  |  | AH |  | 2.254 |  | -17.387 |  | 21.895 |  | 6.410 |  | 53 |  | 0.352 |  | 1.000 |  | 1.000 |  |
|  |  | (AL-X) |  | -6.557 |  | -25.764 |  | 12.650 |  | 6.268 |  | 53 |  | -1.046 |  | .941 |  | 1.000 |  |
|  |  | (AH-X) |  | 3.343 |  | -15.864 |  | 22.550 |  | 6.268 |  | 53 |  | 0.533 |  | .998 |  | 1.000 |  |
|  |  | X |  | 8.643 |  | -13.040 |  | 30.326 |  | 7.076 |  | 53 |  | 1.221 |  | .883 |  | 1.000 |  |
| AL |  | AH |  | 7.986 |  | -10.952 |  | 26.924 |  | 6.180 |  | 53 |  | 1.292 |  | .852 |  | 1.000 |  |
|  |  | (AL-X) |  | -0.825 |  | -19.312 |  | 17.662 |  | 6.033 |  | 53 |  | -0.137 |  | 1.000 |  | 1.000 |  |
|  |  | (AH-X) |  | 9.075 |  | -9.412 |  | 27.562 |  | 6.033 |  | 53 |  | 1.504 |  | .741 |  | 1.000 |  |
|  |  | X |  | 14.375 |  | -6.673 |  | 35.423 |  | 6.869 |  | 53 |  | 2.093 |  | .372 |  | .865 |  |
| AH |  | (AL-X) |  | -8.811 |  | -26.719 |  | 9.096 |  | 5.844 |  | 53 |  | -1.508 |  | .739 |  | 1.000 |  |
|  |  | (AH-X) |  | 1.089 |  | -16.819 |  | 18.996 |  | 5.844 |  | 53 |  | 0.186 |  | 1.000 |  | 1.000 |  |
|  |  | X |  | 6.389 |  | -14.152 |  | 26.930 |  | 6.703 |  | 53 |  | 0.953 |  | .962 |  | 1.000 |  |
| (AL-X) |  | (AH-X) |  | 9.900 |  | -7.530 |  | 27.330 |  | 5.688 |  | 53 |  | 1.741 |  | .593 |  | 1.000 |  |
|  |  | X |  | 15.200 |  | -4.926 |  | 35.326 |  | 6.568 |  | 53 |  | 2.314 |  | .257 |  | .516 |  |
| (AH-X) |  | X |  | 5.300 |  | -14.826 |  | 25.426 |  | 6.568 |  | 53 |  | 0.807 |  | .983 |  | 1.000 |  |
|  | | | | | | | | | | | | | | | | | | | |
|  |  |  |  |  |  |  |  |  |  |  |  |  |  |  |  |  |  |  |  |
| --- | --- | --- | --- | --- | --- | --- | --- | --- | --- | --- | --- | --- | --- | --- | --- | --- | --- | --- | --- |
| \*\* p < .01 | | | | | | | | | | | | | | | | | | | |
| *Note.*  P-value and confidence intervals adjusted for comparing a family of 7 estimates (confidence intervals corrected using the tukey method). | | | | | | | | | | | | | | | | | | | |

| Letter-Based Grouping - Animal group | | | |
| --- | --- | --- | --- |
| Animal group | | Letter | |
| C |  | a |  |
| OV |  | ab |  |
| AL |  | b |  |
| AH |  | ab |  |
| AL-X |  | b |  |
| AH-X |  | ab |  |
| X |  | ab |  |
|  | | | |
|  |  |  |  |
| --- | --- | --- | --- |
| *Note.*  If two or more means share the same grouping symbol, then we cannot show them to be different, but we also did not show them to be the same. | | | |

## HTC

| ANOVA - HCT l/l | | | | | | | | | | | | | | | | | | | | | | | | | |
| --- | --- | --- | --- | --- | --- | --- | --- | --- | --- | --- | --- | --- | --- | --- | --- | --- | --- | --- | --- | --- | --- | --- | --- | --- | --- |
|  | | | | | | | | | | | | | | | | 95% CI for η² | | | |  | | 95% CI for ω² | | | |
| Homogeneity Correction | | Cases | | Sum of Squares | | df | | Mean Square | | F | | p | | η² | | Lower | | Upper | | ω² | | Lower | | Upper | |
| None |  | Animal group |  | 0.033 |  | 6.000 |  | 0.006 |  | 4.173 |  | .002 |  | 0.325 |  | 0.071 |  | 0.467 |  | 0.244 |  | 0.006 |  | 0.383 |  |
|  |  | Residuals |  | 0.069 |  | 52.000 |  | 0.001 |  |  |  |  |  |  |  |  |  |  |  |  |  |  |  |  |  |
| Welch |  | Animal group |  | 0.033 |  | 6.000 |  | 0.006 |  | 5.809 |  | .001 |  | 0.325 |  | 0.071 |  | 0.467 |  | 0.244 |  | 0.006 |  | 0.383 |  |
|  |  | Residuals |  | 0.069 |  | 20.576 |  | 0.003 |  |  |  |  |  |  |  |  |  |  |  |  |  |  |  |  |  |
|  | | | | | | | | | | | | | | | | | | | | | | | | | |
|  |  |  |  |  |  |  |  |  |  |  |  |  |  |  |  |  |  |  |  |  |  |  |  |  |  |
| --- | --- | --- | --- | --- | --- | --- | --- | --- | --- | --- | --- | --- | --- | --- | --- | --- | --- | --- | --- | --- | --- | --- | --- | --- | --- |
| *Note.*  Type III Sum of Squares | | | | | | | | | | | | | | | | | | | | | | | | | |

### Descriptives

| Descriptives - HCT l/l | | | | | | | | | | | |
| --- | --- | --- | --- | --- | --- | --- | --- | --- | --- | --- | --- |
| Animal group | | N | | Mean | | SD | | SE | | Coefficient of variation | |
| C |  | 10 |  | 0.406 |  | 0.012 |  | 0.004 |  | 0.030 |  |
| OV |  | 7 |  | 0.456 |  | 0.030 |  | 0.011 |  | 0.065 |  |
| AL |  | 8 |  | 0.458 |  | 0.051 |  | 0.018 |  | 0.112 |  |
| AH |  | 9 |  | 0.427 |  | 0.033 |  | 0.011 |  | 0.077 |  |
| AL-X |  | 9 |  | 0.476 |  | 0.050 |  | 0.017 |  | 0.105 |  |
| AH-X |  | 10 |  | 0.434 |  | 0.036 |  | 0.011 |  | 0.084 |  |
| X |  | 6 |  | 0.414 |  | 0.025 |  | 0.010 |  | 0.060 |  |
|  | | | | | | | | | | | |

#### Bar plots

#####

### Assumption Checks

| Test for Equality of Variances (Levene's) | | | | | | | |
| --- | --- | --- | --- | --- | --- | --- | --- |
| F | | df1 | | df2 | | p | |
| 3.464 |  | 6.000 |  | 52.00 |  | .006 |  |
|  | | | | | | | |

### Post Hoc Tests

#### Standard (HSD)

| Post Hoc Comparisons - Animal group | | | | | | | | | | | | | | | | | | | |
| --- | --- | --- | --- | --- | --- | --- | --- | --- | --- | --- | --- | --- | --- | --- | --- | --- | --- | --- | --- |
|  | | | | | | 95% CI for Mean Difference | | | |  | | | | | | | | | |
|  | |  | | Mean Difference | | Lower | | Upper | | SE | | df | | t | | ptukey | | pbonf | |
| C |  | OV |  | -0.050 |  | -0.105 |  | 0.005 |  | 0.018 |  | 52 |  | -2.790 |  | .097 |  | .154 |  |
|  |  | AL |  | -0.051 |  | -0.104 |  | 0.001 |  | 0.017 |  | 52 |  | -2.985 |  | .061 |  | .090 |  |
|  |  | AH |  | -0.021 |  | -0.072 |  | 0.031 |  | 0.017 |  | 52 |  | -1.237 |  | .876 |  | 1.000 |  |
|  |  | (AL-X) |  | -0.070 |  | -0.121 |  | -0.019 |  | 0.017 |  | 52 |  | -4.183 |  | .002 | \*\* | .002 | \*\* |
|  |  | (AH-X) |  | -0.028 |  | -0.078 |  | 0.022 |  | 0.016 |  | 52 |  | -1.740 |  | .593 |  | 1.000 |  |
|  |  | X |  | -0.008 |  | -0.066 |  | 0.049 |  | 0.019 |  | 52 |  | -0.444 |  | .999 |  | 1.000 |  |
| OV |  | AL |  | -0.002 |  | -0.059 |  | 0.056 |  | 0.019 |  | 52 |  | -0.080 |  | 1.000 |  | 1.000 |  |
|  |  | AH |  | 0.029 |  | -0.027 |  | 0.086 |  | 0.018 |  | 52 |  | 1.601 |  | .683 |  | 1.000 |  |
|  |  | (AL-X) |  | -0.020 |  | -0.076 |  | 0.036 |  | 0.018 |  | 52 |  | -1.085 |  | .930 |  | 1.000 |  |
|  |  | (AH-X) |  | 0.022 |  | -0.033 |  | 0.077 |  | 0.018 |  | 52 |  | 1.211 |  | .887 |  | 1.000 |  |
|  |  | X |  | 0.042 |  | -0.020 |  | 0.104 |  | 0.020 |  | 52 |  | 2.059 |  | .392 |  | .934 |  |
| AL |  | AH |  | 0.031 |  | -0.023 |  | 0.085 |  | 0.018 |  | 52 |  | 1.745 |  | .590 |  | 1.000 |  |
|  |  | (AL-X) |  | -0.018 |  | -0.073 |  | 0.036 |  | 0.018 |  | 52 |  | -1.041 |  | .942 |  | 1.000 |  |
|  |  | (AH-X) |  | 0.023 |  | -0.030 |  | 0.076 |  | 0.017 |  | 52 |  | 1.345 |  | .827 |  | 1.000 |  |
|  |  | X |  | 0.043 |  | -0.017 |  | 0.103 |  | 0.020 |  | 52 |  | 2.198 |  | .314 |  | .681 |  |
| AH |  | (AL-X) |  | -0.049 |  | -0.102 |  | 0.003 |  | 0.017 |  | 52 |  | -2.871 |  | .080 |  | .124 |  |
|  |  | (AH-X) |  | -0.008 |  | -0.059 |  | 0.044 |  | 0.017 |  | 52 |  | -0.457 |  | .999 |  | 1.000 |  |
|  |  | X |  | 0.012 |  | -0.046 |  | 0.071 |  | 0.019 |  | 52 |  | 0.643 |  | .995 |  | 1.000 |  |
| (AL-X) |  | (AH-X) |  | 0.042 |  | -0.010 |  | 0.093 |  | 0.017 |  | 52 |  | 2.489 |  | .185 |  | .337 |  |
|  |  | X |  | 0.062 |  | 0.003 |  | 0.120 |  | 0.019 |  | 52 |  | 3.211 |  | .034 | \* | .048 | \* |
| (AH-X) |  | X |  | 0.020 |  | -0.038 |  | 0.078 |  | 0.019 |  | 52 |  | 1.063 |  | .936 |  | 1.000 |  |
|  | | | | | | | | | | | | | | | | | | | |
|  |  |  |  |  |  |  |  |  |  |  |  |  |  |  |  |  |  |  |  |
| --- | --- | --- | --- | --- | --- | --- | --- | --- | --- | --- | --- | --- | --- | --- | --- | --- | --- | --- | --- |
| \* p < .05, \*\* p < .01 | | | | | | | | | | | | | | | | | | | |
| *Note.*  P-value and confidence intervals adjusted for comparing a family of 7 estimates (confidence intervals corrected using the tukey method). | | | | | | | | | | | | | | | | | | | |

| Letter-Based Grouping - Animal group | | | |
| --- | --- | --- | --- |
| Animal group | | Letter | |
| C |  | a |  |
| OV |  | ab |  |
| AL |  | ab |  |
| AH |  | ab |  |
| AL-X |  | b |  |
| AH-X |  | ab |  |
| X |  | a |  |
|  | | | |
|  |  |  |  |
| --- | --- | --- | --- |
| *Note.*  If two or more means share the same grouping symbol, then we cannot show them to be different, but we also did not show them to be the same. | | | |

## MCV

| ANOVA - MCV fl | | | | | | | | | | | | | | | | | | | | | | | | | |
| --- | --- | --- | --- | --- | --- | --- | --- | --- | --- | --- | --- | --- | --- | --- | --- | --- | --- | --- | --- | --- | --- | --- | --- | --- | --- |
|  | | | | | | | | | | | | | | | | 95% CI for η² | | | |  | | 95% CI for ω² | | | |
| Homogeneity Correction | | Cases | | Sum of Squares | | df | | Mean Square | | F | | p | | η² | | Lower | | Upper | | ω² | | Lower | | Upper | |
| None |  | Animal group |  | 11.78 |  | 6.000 |  | 1.964 |  | 0.374 |  | .892 |  | 0.041 |  | 0.000 |  | 0.070 |  | 0.000 |  | 0.000 |  | 0.000 |  |
|  |  | Residuals |  | 277.95 |  | 53.000 |  | 5.244 |  |  |  |  |  |  |  |  |  |  |  |  |  |  |  |  |  |
| Welch |  | Animal group |  | 11.78 |  | 6.000 |  | 1.964 |  | 0.388 |  | .879 |  | 0.041 |  | 0.000 |  | 0.070 |  | 0.000 |  | 0.000 |  | 0.000 |  |
|  |  | Residuals |  | 277.95 |  | 22.479 |  | 12.365 |  |  |  |  |  |  |  |  |  |  |  |  |  |  |  |  |  |
|  | | | | | | | | | | | | | | | | | | | | | | | | | |
|  |  |  |  |  |  |  |  |  |  |  |  |  |  |  |  |  |  |  |  |  |  |  |  |  |  |
| --- | --- | --- | --- | --- | --- | --- | --- | --- | --- | --- | --- | --- | --- | --- | --- | --- | --- | --- | --- | --- | --- | --- | --- | --- | --- |
| *Note.*  Type III Sum of Squares | | | | | | | | | | | | | | | | | | | | | | | | | |

### Descriptives

| Descriptives - MCV fl | | | | | | | | | | | |
| --- | --- | --- | --- | --- | --- | --- | --- | --- | --- | --- | --- |
| Animal group | | N | | Mean | | SD | | SE | | Coefficient of variation | |
| C |  | 10 |  | 58.40 |  | 1.955 |  | 0.618 |  | 0.033 |  |
| OV |  | 7 |  | 58.29 |  | 2.628 |  | 0.993 |  | 0.045 |  |
| AL |  | 8 |  | 58.75 |  | 1.035 |  | 0.366 |  | 0.018 |  |
| AH |  | 9 |  | 58.89 |  | 1.364 |  | 0.455 |  | 0.023 |  |
| AL-X |  | 10 |  | 59.40 |  | 3.950 |  | 1.249 |  | 0.066 |  |
| AH-X |  | 10 |  | 59.50 |  | 1.900 |  | 0.601 |  | 0.032 |  |
| X |  | 6 |  | 59.17 |  | 1.169 |  | 0.477 |  | 0.020 |  |
|  | | | | | | | | | | | |

#### Bar plots

#####

### Assumption Checks

| Test for Equality of Variances (Levene's) | | | | | | | |
| --- | --- | --- | --- | --- | --- | --- | --- |
| F | | df1 | | df2 | | p | |
| 2.266 |  | 6.000 |  | 53.00 |  | .051 |  |
|  | | | | | | | |

## MCH

| ANOVA - MCH pg | | | | | | | | | | | | | | | | | | | | | | | | | |
| --- | --- | --- | --- | --- | --- | --- | --- | --- | --- | --- | --- | --- | --- | --- | --- | --- | --- | --- | --- | --- | --- | --- | --- | --- | --- |
|  | | | | | | | | | | | | | | | | 95% CI for η² | | | |  | | 95% CI for ω² | | | |
| Homogeneity Correction | | Cases | | Sum of Squares | | df | | Mean Square | | F | | p | | η² | | Lower | | Upper | | ω² | | Lower | | Upper | |
| None |  | Animal group |  | 14.90 |  | 6.000 |  | 2.483 |  | 1.733 |  | .132 |  | 0.167 |  | 0.000 |  | 0.292 |  | 0.069 |  | 0.000 |  | 0.139 |  |
|  |  | Residuals |  | 74.48 |  | 52.000 |  | 1.432 |  |  |  |  |  |  |  |  |  |  |  |  |  |  |  |  |  |
| Welch |  | Animal group |  | 14.90 |  | 6.000 |  | 2.483 |  | 2.641 |  | .044 |  | 0.167 |  | 0.000 |  | 0.292 |  | 0.069 |  | 0.000 |  | 0.139 |  |
|  |  | Residuals |  | 74.48 |  | 22.195 |  | 3.356 |  |  |  |  |  |  |  |  |  |  |  |  |  |  |  |  |  |
|  | | | | | | | | | | | | | | | | | | | | | | | | | |
|  |  |  |  |  |  |  |  |  |  |  |  |  |  |  |  |  |  |  |  |  |  |  |  |  |  |
| --- | --- | --- | --- | --- | --- | --- | --- | --- | --- | --- | --- | --- | --- | --- | --- | --- | --- | --- | --- | --- | --- | --- | --- | --- | --- |
| *Note.*  Type III Sum of Squares | | | | | | | | | | | | | | | | | | | | | | | | | |

### Descriptives

| Descriptives - MCH pg | | | | | | | | | | | |
| --- | --- | --- | --- | --- | --- | --- | --- | --- | --- | --- | --- |
| Animal group | | N | | Mean | | SD | | SE | | Coefficient of variation | |
| C |  | 10 |  | 21.89 |  | 0.679 |  | 0.215 |  | 0.031 |  |
| OV |  | 7 |  | 21.80 |  | 1.066 |  | 0.403 |  | 0.049 |  |
| AL |  | 8 |  | 22.56 |  | 0.735 |  | 0.260 |  | 0.033 |  |
| AH |  | 9 |  | 23.21 |  | 1.947 |  | 0.649 |  | 0.084 |  |
| AL-X |  | 9 |  | 22.37 |  | 1.339 |  | 0.446 |  | 0.060 |  |
| AH-X |  | 10 |  | 22.88 |  | 1.223 |  | 0.387 |  | 0.053 |  |
| X |  | 6 |  | 23.02 |  | 0.567 |  | 0.232 |  | 0.025 |  |
|  | | | | | | | | | | | |

#### Bar plots

#####

### Assumption Checks

| Test for Equality of Variances (Levene's) | | | | | | | |
| --- | --- | --- | --- | --- | --- | --- | --- |
| F | | df1 | | df2 | | p | |
| 0.915 |  | 6.000 |  | 52.00 |  | .492 |  |
|  | | | | | | | |

### Post Hoc Tests

#### Standard (HSD)

| Post Hoc Comparisons - Animal group | | | | | | | | | | | | | | | | | | | |
| --- | --- | --- | --- | --- | --- | --- | --- | --- | --- | --- | --- | --- | --- | --- | --- | --- | --- | --- | --- |
|  | | | | | | 95% CI for Mean Difference | | | |  | | | | | | | | | |
|  | |  | | Mean Difference | | Lower | | Upper | | SE | | df | | t | | ptukey | | pbonf | |
| C |  | OV |  | 0.090 |  | -1.719 |  | 1.899 |  | 0.590 |  | 52 |  | 0.153 |  | 1.000 |  | 1.000 |  |
|  |  | AL |  | -0.672 |  | -2.413 |  | 1.068 |  | 0.568 |  | 52 |  | -1.185 |  | .897 |  | 1.000 |  |
|  |  | AH |  | -1.321 |  | -3.007 |  | 0.365 |  | 0.550 |  | 52 |  | -2.402 |  | .218 |  | .418 |  |
|  |  | (AL-X) |  | -0.477 |  | -2.163 |  | 1.210 |  | 0.550 |  | 52 |  | -0.867 |  | .976 |  | 1.000 |  |
|  |  | (AH-X) |  | -0.990 |  | -2.631 |  | 0.651 |  | 0.535 |  | 52 |  | -1.850 |  | .522 |  | 1.000 |  |
|  |  | X |  | -1.127 |  | -3.022 |  | 0.769 |  | 0.618 |  | 52 |  | -1.823 |  | .539 |  | 1.000 |  |
| OV |  | AL |  | -0.762 |  | -2.662 |  | 1.137 |  | 0.619 |  | 52 |  | -1.231 |  | .879 |  | 1.000 |  |
|  |  | AH |  | -1.411 |  | -3.261 |  | 0.438 |  | 0.603 |  | 52 |  | -2.340 |  | .245 |  | .487 |  |
|  |  | (AL-X) |  | -0.567 |  | -2.416 |  | 1.283 |  | 0.603 |  | 52 |  | -0.940 |  | .964 |  | 1.000 |  |
|  |  | (AH-X) |  | -1.080 |  | -2.889 |  | 0.729 |  | 0.590 |  | 52 |  | -1.831 |  | .534 |  | 1.000 |  |
|  |  | X |  | -1.217 |  | -3.259 |  | 0.825 |  | 0.666 |  | 52 |  | -1.827 |  | .536 |  | 1.000 |  |
| AL |  | AH |  | -0.649 |  | -2.432 |  | 1.135 |  | 0.582 |  | 52 |  | -1.115 |  | .921 |  | 1.000 |  |
|  |  | (AL-X) |  | 0.196 |  | -1.588 |  | 1.979 |  | 0.582 |  | 52 |  | 0.337 |  | 1.000 |  | 1.000 |  |
|  |  | (AH-X) |  | -0.318 |  | -2.058 |  | 1.423 |  | 0.568 |  | 52 |  | -0.559 |  | .998 |  | 1.000 |  |
|  |  | X |  | -0.454 |  | -2.436 |  | 1.528 |  | 0.646 |  | 52 |  | -0.703 |  | .992 |  | 1.000 |  |
| AH |  | (AL-X) |  | 0.844 |  | -0.886 |  | 2.575 |  | 0.564 |  | 52 |  | 1.497 |  | .745 |  | 1.000 |  |
|  |  | (AH-X) |  | 0.331 |  | -1.355 |  | 2.017 |  | 0.550 |  | 52 |  | 0.602 |  | .996 |  | 1.000 |  |
|  |  | X |  | 0.194 |  | -1.740 |  | 2.129 |  | 0.631 |  | 52 |  | 0.308 |  | 1.000 |  | 1.000 |  |
| (AL-X) |  | (AH-X) |  | -0.513 |  | -2.200 |  | 1.173 |  | 0.550 |  | 52 |  | -0.934 |  | .965 |  | 1.000 |  |
|  |  | X |  | -0.650 |  | -2.584 |  | 1.284 |  | 0.631 |  | 52 |  | -1.030 |  | .944 |  | 1.000 |  |
| (AH-X) |  | X |  | -0.137 |  | -2.032 |  | 1.759 |  | 0.618 |  | 52 |  | -0.221 |  | 1.000 |  | 1.000 |  |
|  | | | | | | | | | | | | | | | | | | | |
|  |  |  |  |  |  |  |  |  |  |  |  |  |  |  |  |  |  |  |  |
| --- | --- | --- | --- | --- | --- | --- | --- | --- | --- | --- | --- | --- | --- | --- | --- | --- | --- | --- | --- |
| *Note.*  P-value and confidence intervals adjusted for comparing a family of 7 estimates (confidence intervals corrected using the tukey method). | | | | | | | | | | | | | | | | | | | |

| Letter-Based Grouping - Animal group | | | |
| --- | --- | --- | --- |
| Animal group | | Letter | |
| C |  | a |  |
| OV |  | a |  |
| AL |  | a |  |
| AH |  | a |  |
| AL-X |  | a |  |
| AH-X |  | a |  |
| X |  | a |  |
|  | | | |
|  |  |  |  |
| --- | --- | --- | --- |
| *Note.*  If two or more means share the same grouping symbol, then we cannot show them to be different, but we also did not show them to be the same. | | | |

## MCHC

| ANOVA - MCHC g/l | | | | | | | | | | | | | | | | | | | | | | | | | |
| --- | --- | --- | --- | --- | --- | --- | --- | --- | --- | --- | --- | --- | --- | --- | --- | --- | --- | --- | --- | --- | --- | --- | --- | --- | --- |
|  | | | | | | | | | | | | | | | | 95% CI for η² | | | |  | | 95% CI for ω² | | | |
| Homogeneity Correction | | Cases | | Sum of Squares | | df | | Mean Square | | F | | p | | η² | | Lower | | Upper | | ω² | | Lower | | Upper | |
| None |  | Animal group |  | 3296 |  | 6.000 |  | 549.3 |  | 2.837 |  | .018 |  | 0.247 |  | 0.008 |  | 0.386 |  | 0.157 |  | 0.000 |  | 0.280 |  |
|  |  | Residuals |  | 10069 |  | 52.000 |  | 193.6 |  |  |  |  |  |  |  |  |  |  |  |  |  |  |  |  |  |
| Welch |  | Animal group |  | 3296 |  | 6.000 |  | 549.3 |  | 4.290 |  | .005 |  | 0.247 |  | 0.008 |  | 0.386 |  | 0.157 |  | 0.000 |  | 0.280 |  |
|  |  | Residuals |  | 10069 |  | 21.975 |  | 458.2 |  |  |  |  |  |  |  |  |  |  |  |  |  |  |  |  |  |
|  | | | | | | | | | | | | | | | | | | | | | | | | | |
|  |  |  |  |  |  |  |  |  |  |  |  |  |  |  |  |  |  |  |  |  |  |  |  |  |  |
| --- | --- | --- | --- | --- | --- | --- | --- | --- | --- | --- | --- | --- | --- | --- | --- | --- | --- | --- | --- | --- | --- | --- | --- | --- | --- |
| *Note.*  Type III Sum of Squares | | | | | | | | | | | | | | | | | | | | | | | | | |

### Descriptives

| Descriptives - MCHC g/l | | | | | | | | | | | |
| --- | --- | --- | --- | --- | --- | --- | --- | --- | --- | --- | --- |
| Animal group | | N | | Mean | | SD | | SE | | Coefficient of variation | |
| C |  | 10 |  | 375.1 |  | 8.034 |  | 2.541 |  | 0.021 |  |
| OV |  | 7 |  | 373.3 |  | 4.821 |  | 1.822 |  | 0.013 |  |
| AL |  | 8 |  | 384.6 |  | 10.676 |  | 3.775 |  | 0.028 |  |
| AH |  | 9 |  | 394.9 |  | 29.165 |  | 9.722 |  | 0.074 |  |
| AL-X |  | 9 |  | 376.2 |  | 6.300 |  | 2.100 |  | 0.017 |  |
| AH-X |  | 10 |  | 384.6 |  | 10.741 |  | 3.397 |  | 0.028 |  |
| X |  | 6 |  | 390.0 |  | 8.832 |  | 3.606 |  | 0.023 |  |
|  | | | | | | | | | | | |

#### Bar plots

#####

### Assumption Checks

| Test for Equality of Variances (Levene's) | | | | | | | |
| --- | --- | --- | --- | --- | --- | --- | --- |
| F | | df1 | | df2 | | p | |
| 2.381 |  | 6.000 |  | 52.00 |  | .042 |  |
|  | | | | | | | |

### Post Hoc Tests

#### Standard (HSD)

| Post Hoc Comparisons - Animal group | | | | | | | | | | | | | | | | | | | |
| --- | --- | --- | --- | --- | --- | --- | --- | --- | --- | --- | --- | --- | --- | --- | --- | --- | --- | --- | --- |
|  | | | | | | 95% CI for Mean Difference | | | |  | | | | | | | | | |
|  | |  | | Mean Difference | | Lower | | Upper | | SE | | df | | t | | ptukey | | pbonf | |
| C |  | OV |  | 1.814 |  | -19.215 |  | 22.844 |  | 6.858 |  | 52 |  | 0.265 |  | 1.000 |  | 1.000 |  |
|  |  | AL |  | -9.525 |  | -29.767 |  | 10.717 |  | 6.601 |  | 52 |  | -1.443 |  | .776 |  | 1.000 |  |
|  |  | AH |  | -19.789 |  | -39.396 |  | -0.182 |  | 6.394 |  | 52 |  | -3.095 |  | .047 | \* | .066 |  |
|  |  | (AL-X) |  | -1.122 |  | -20.729 |  | 18.485 |  | 6.394 |  | 52 |  | -0.176 |  | 1.000 |  | 1.000 |  |
|  |  | (AH-X) |  | -9.500 |  | -28.584 |  | 9.584 |  | 6.223 |  | 52 |  | -1.527 |  | .728 |  | 1.000 |  |
|  |  | X |  | -14.900 |  | -36.936 |  | 7.136 |  | 7.186 |  | 52 |  | -2.074 |  | .383 |  | .905 |  |
| OV |  | AL |  | -11.339 |  | -33.425 |  | 10.746 |  | 7.202 |  | 52 |  | -1.574 |  | .699 |  | 1.000 |  |
|  |  | AH |  | -21.603 |  | -43.108 |  | -0.098 |  | 7.013 |  | 52 |  | -3.081 |  | .048 | \* | .069 |  |
|  |  | (AL-X) |  | -2.937 |  | -24.442 |  | 18.569 |  | 7.013 |  | 52 |  | -0.419 |  | 1.000 |  | 1.000 |  |
|  |  | (AH-X) |  | -11.314 |  | -32.344 |  | 9.715 |  | 6.858 |  | 52 |  | -1.650 |  | .651 |  | 1.000 |  |
|  |  | X |  | -16.714 |  | -40.455 |  | 7.027 |  | 7.742 |  | 52 |  | -2.159 |  | .335 |  | .745 |  |
| AL |  | AH |  | -10.264 |  | -30.999 |  | 10.471 |  | 6.762 |  | 52 |  | -1.518 |  | .733 |  | 1.000 |  |
|  |  | (AL-X) |  | 8.403 |  | -12.333 |  | 29.138 |  | 6.762 |  | 52 |  | 1.243 |  | .874 |  | 1.000 |  |
|  |  | (AH-X) |  | 0.025 |  | -20.217 |  | 20.267 |  | 6.601 |  | 52 |  | 0.004 |  | 1.000 |  | 1.000 |  |
|  |  | X |  | -5.375 |  | -28.421 |  | 17.671 |  | 7.515 |  | 52 |  | -0.715 |  | .991 |  | 1.000 |  |
| AH |  | (AL-X) |  | 18.667 |  | -1.450 |  | 38.783 |  | 6.560 |  | 52 |  | 2.846 |  | .085 |  | .133 |  |
|  |  | (AH-X) |  | 10.289 |  | -9.318 |  | 29.896 |  | 6.394 |  | 52 |  | 1.609 |  | .677 |  | 1.000 |  |
|  |  | X |  | 4.889 |  | -17.602 |  | 27.380 |  | 7.334 |  | 52 |  | 0.667 |  | .994 |  | 1.000 |  |
| (AL-X) |  | (AH-X) |  | -8.378 |  | -27.985 |  | 11.229 |  | 6.394 |  | 52 |  | -1.310 |  | .844 |  | 1.000 |  |
|  |  | X |  | -13.778 |  | -36.268 |  | 8.713 |  | 7.334 |  | 52 |  | -1.879 |  | .503 |  | 1.000 |  |
| (AH-X) |  | X |  | -5.400 |  | -27.436 |  | 16.636 |  | 7.186 |  | 52 |  | -0.751 |  | .988 |  | 1.000 |  |
|  | | | | | | | | | | | | | | | | | | | |
|  |  |  |  |  |  |  |  |  |  |  |  |  |  |  |  |  |  |  |  |
| --- | --- | --- | --- | --- | --- | --- | --- | --- | --- | --- | --- | --- | --- | --- | --- | --- | --- | --- | --- |
| \* p < .05 | | | | | | | | | | | | | | | | | | | |
| *Note.*  P-value and confidence intervals adjusted for comparing a family of 7 estimates (confidence intervals corrected using the tukey method). | | | | | | | | | | | | | | | | | | | |

| Letter-Based Grouping - Animal group | | | |
| --- | --- | --- | --- |
| Animal group | | Letter | |
| C |  | a |  |
| OV |  | a |  |
| AL |  | ab |  |
| AH |  | b |  |
| AL-X |  | ab |  |
| AH-X |  | ab |  |
| X |  | ab |  |
|  | | | |
|  |  |  |  |
| --- | --- | --- | --- |
| *Note.*  If two or more means share the same grouping symbol, then we cannot show them to be different, but we also did not show them to be the same. | | | |

## PLT

| ANOVA - PLT 10^9/l | | | | | | | | | | | | | | | | | | | | | | | | | |
| --- | --- | --- | --- | --- | --- | --- | --- | --- | --- | --- | --- | --- | --- | --- | --- | --- | --- | --- | --- | --- | --- | --- | --- | --- | --- |
|  | | | | | | | | | | | | | | | | 95% CI for η² | | | |  | | 95% CI for ω² | | | |
| Homogeneity Correction | | Cases | | Sum of Squares | | df | | Mean Square | | F | | p | | η² | | Lower | | Upper | | ω² | | Lower | | Upper | |
| None |  | Animal group |  | 272706 |  | 6.000 |  | 45451 |  | 6.893 |  | < .001 |  | 0.438 |  | 0.192 |  | 0.571 |  | 0.371 |  | 0.118 |  | 0.510 |  |
|  |  | Residuals |  | 349471 |  | 53.000 |  | 6594 |  |  |  |  |  |  |  |  |  |  |  |  |  |  |  |  |  |
| Welch |  | Animal group |  | 272706 |  | 6.000 |  | 45451 |  | 12.192 |  | < .001 |  | 0.438 |  | 0.192 |  | 0.571 |  | 0.371 |  | 0.118 |  | 0.510 |  |
|  |  | Residuals |  | 349471 |  | 22.490 |  | 15539 |  |  |  |  |  |  |  |  |  |  |  |  |  |  |  |  |  |
|  | | | | | | | | | | | | | | | | | | | | | | | | | |
|  |  |  |  |  |  |  |  |  |  |  |  |  |  |  |  |  |  |  |  |  |  |  |  |  |  |
| --- | --- | --- | --- | --- | --- | --- | --- | --- | --- | --- | --- | --- | --- | --- | --- | --- | --- | --- | --- | --- | --- | --- | --- | --- | --- |
| *Note.*  Type III Sum of Squares | | | | | | | | | | | | | | | | | | | | | | | | | |

### Descriptives

| Descriptives - PLT 10^9/l | | | | | | | | | | | |
| --- | --- | --- | --- | --- | --- | --- | --- | --- | --- | --- | --- |
| Animal group | | N | | Mean | | SD | | SE | | Coefficient of variation | |
| C |  | 10 |  | 716.5 |  | 37.73 |  | 11.93 |  | 0.053 |  |
| OV |  | 7 |  | 623.0 |  | 64.20 |  | 24.27 |  | 0.103 |  |
| AL |  | 8 |  | 594.4 |  | 104.03 |  | 36.78 |  | 0.175 |  |
| AH |  | 9 |  | 543.4 |  | 94.71 |  | 31.57 |  | 0.174 |  |
| AL-X |  | 10 |  | 556.2 |  | 98.30 |  | 31.08 |  | 0.177 |  |
| AH-X |  | 10 |  | 507.2 |  | 90.08 |  | 28.49 |  | 0.178 |  |
| X |  | 6 |  | 620.8 |  | 29.71 |  | 12.13 |  | 0.048 |  |
|  | | | | | | | | | | | |

#### Bar plots

#####

### Assumption Checks

| Test for Equality of Variances (Levene's) | | | | | | | |
| --- | --- | --- | --- | --- | --- | --- | --- |
| F | | df1 | | df2 | | p | |
| 2.774 |  | 6.000 |  | 53.00 |  | .020 |  |
|  | | | | | | | |

### Post Hoc Tests

#### Standard (HSD)

| Post Hoc Comparisons - Animal group | | | | | | | | | | | | | | | | | | | |
| --- | --- | --- | --- | --- | --- | --- | --- | --- | --- | --- | --- | --- | --- | --- | --- | --- | --- | --- | --- |
|  | | | | | | 95% CI for Mean Difference | | | |  | | | | | | | | | |
|  | |  | | Mean Difference | | Lower | | Upper | | SE | | df | | t | | ptukey | | pbonf | |
| C |  | OV |  | 93.500 |  | -29.126 |  | 216.13 |  | 40.02 |  | 53 |  | 2.337 |  | .246 |  | .489 |  |
|  |  | AL |  | 122.125 |  | 4.094 |  | 240.16 |  | 38.52 |  | 53 |  | 3.171 |  | .038 | \* | .053 |  |
|  |  | AH |  | 173.056 |  | 58.725 |  | 287.39 |  | 37.31 |  | 53 |  | 4.638 |  | < .001 | \*\*\* | < .001 | \*\*\* |
|  |  | (AL-X) |  | 160.300 |  | 49.019 |  | 271.58 |  | 36.31 |  | 53 |  | 4.414 |  | < .001 | \*\*\* | .001 | \*\* |
|  |  | (AH-X) |  | 209.300 |  | 98.019 |  | 320.58 |  | 36.31 |  | 53 |  | 5.764 |  | < .001 | \*\*\* | < .001 | \*\*\* |
|  |  | X |  | 95.667 |  | -32.829 |  | 224.16 |  | 41.93 |  | 53 |  | 2.281 |  | .272 |  | .558 |  |
| OV |  | AL |  | 28.625 |  | -100.158 |  | 157.41 |  | 42.03 |  | 53 |  | 0.681 |  | .993 |  | 1.000 |  |
|  |  | AH |  | 79.556 |  | -45.844 |  | 204.95 |  | 40.92 |  | 53 |  | 1.944 |  | .461 |  | 1.000 |  |
|  |  | (AL-X) |  | 66.800 |  | -55.826 |  | 189.43 |  | 40.02 |  | 53 |  | 1.669 |  | .639 |  | 1.000 |  |
|  |  | (AH-X) |  | 115.800 |  | -6.826 |  | 238.43 |  | 40.02 |  | 53 |  | 2.894 |  | .076 |  | .116 |  |
|  |  | X |  | 2.167 |  | -136.270 |  | 140.60 |  | 45.18 |  | 53 |  | 0.048 |  | 1.000 |  | 1.000 |  |
| AL |  | AH |  | 50.931 |  | -69.980 |  | 171.84 |  | 39.46 |  | 53 |  | 1.291 |  | .853 |  | 1.000 |  |
|  |  | (AL-X) |  | 38.175 |  | -79.856 |  | 156.21 |  | 38.52 |  | 53 |  | 0.991 |  | .954 |  | 1.000 |  |
|  |  | (AH-X) |  | 87.175 |  | -30.856 |  | 205.21 |  | 38.52 |  | 53 |  | 2.263 |  | .281 |  | .583 |  |
|  |  | X |  | -26.458 |  | -160.843 |  | 107.93 |  | 43.85 |  | 53 |  | -0.603 |  | .996 |  | 1.000 |  |
| AH |  | (AL-X) |  | -12.756 |  | -127.086 |  | 101.57 |  | 37.31 |  | 53 |  | -0.342 |  | 1.000 |  | 1.000 |  |
|  |  | (AH-X) |  | 36.244 |  | -78.086 |  | 150.57 |  | 37.31 |  | 53 |  | 0.971 |  | .958 |  | 1.000 |  |
|  |  | X |  | -77.389 |  | -208.535 |  | 53.76 |  | 42.80 |  | 53 |  | -1.808 |  | .549 |  | 1.000 |  |
| (AL-X) |  | (AH-X) |  | 49.000 |  | -62.281 |  | 160.28 |  | 36.31 |  | 53 |  | 1.349 |  | .825 |  | 1.000 |  |
|  |  | X |  | -64.633 |  | -193.129 |  | 63.86 |  | 41.93 |  | 53 |  | -1.541 |  | .719 |  | 1.000 |  |
| (AH-X) |  | X |  | -113.633 |  | -242.129 |  | 14.86 |  | 41.93 |  | 53 |  | -2.710 |  | .116 |  | .190 |  |
|  | | | | | | | | | | | | | | | | | | | |
|  |  |  |  |  |  |  |  |  |  |  |  |  |  |  |  |  |  |  |  |
| --- | --- | --- | --- | --- | --- | --- | --- | --- | --- | --- | --- | --- | --- | --- | --- | --- | --- | --- | --- |
| \* p < .05, \*\* p < .01, \*\*\* p < .001 | | | | | | | | | | | | | | | | | | | |
| *Note.*  P-value and confidence intervals adjusted for comparing a family of 7 estimates (confidence intervals corrected using the tukey method). | | | | | | | | | | | | | | | | | | | |

| Letter-Based Grouping - Animal group | | | |
| --- | --- | --- | --- |
| Animal group | | Letter | |
| C |  | b |  |
| OV |  | ab |  |
| AL |  | a |  |
| AH |  | a |  |
| AL-X |  | a |  |
| AH-X |  | a |  |
| X |  | ab |  |
|  | | | |
|  |  |  |  |
| --- | --- | --- | --- |
| *Note.*  If two or more means share the same grouping symbol, then we cannot show them to be different, but we also did not show them to be the same. | | | |
