# Supplementary material for: The Early Effect of Alendronate, Hop Extract and Their Combination on Bone Structural Properties in a Rat Model of Osteoporosis
Source: Med Sci (Basel). 2026 May 5;14(2):239. doi: 10.3390/medsci14020239 (PMC13214732; doi:10.3390/medsci14020239)
Supplement: Supplementary file 1 [file medsci-14-00239-s001.zip › Table S3.html]

JASP 


# Results

## Descriptive Statistics

| Descriptive Statistics | | | | | | | | | | | | | | | | | | | | | | | | | |
| --- | --- | --- | --- | --- | --- | --- | --- | --- | --- | --- | --- | --- | --- | --- | --- | --- | --- | --- | --- | --- | --- | --- | --- | --- | --- |
|  | |  | | Valid | | Missing | | Median | | Mean | | Std. Deviation | | Shapiro-Wilk | | P-value of Shapiro-Wilk | | Minimum | | Maximum | | 25th percentile | | 75th percentile | |
| CTX1 |  | C |  | 10 |  | 0 |  | 1.000 |  | 1.000 |  | 0.139 |  | 0.952 |  | .692 |  | 0.729 |  | 1.271 |  | 0.950 |  | 1.050 |  |
| CTX1 |  | OV |  | 10 |  | 0 |  | 0.972 |  | 0.908 |  | 0.210 |  | 0.937 |  | .520 |  | 0.513 |  | 1.193 |  | 0.755 |  | 1.035 |  |
| CTX1 |  | AL |  | 10 |  | 0 |  | 0.944 |  | 0.977 |  | 0.167 |  | 0.931 |  | .458 |  | 0.743 |  | 1.213 |  | 0.838 |  | 1.118 |  |
| CTX1 |  | AH |  | 9 |  | 1 |  | 0.940 |  | 1.067 |  | 0.402 |  | 0.918 |  | .377 |  | 0.397 |  | 1.558 |  | 0.816 |  | 1.476 |  |
| CTX1 |  | AL-X |  | 10 |  | 0 |  | 1.114 |  | 1.084 |  | 0.244 |  | 0.899 |  | .213 |  | 0.735 |  | 1.368 |  | 0.864 |  | 1.286 |  |
| CTX1 |  | AH-X |  | 10 |  | 0 |  | 0.979 |  | 0.912 |  | 0.206 |  | 0.906 |  | .257 |  | 0.459 |  | 1.176 |  | 0.811 |  | 0.990 |  |
| CTX1 |  | X |  | 10 |  | 0 |  | 1.239 |  | 1.197 |  | 0.249 |  | 0.922 |  | .377 |  | 0.902 |  | 1.680 |  | 0.956 |  | 1.317 |  |
| P1NP |  | C |  | 10 |  | 0 |  | 1.000 |  | 1.000 |  | 0.084 |  | 0.985 |  | .985 |  | 0.850 |  | 1.150 |  | 0.960 |  | 1.040 |  |
| P1NP |  | OV |  | 9 |  | 1 |  | 0.954 |  | 0.980 |  | 0.069 |  | 0.920 |  | .391 |  | 0.904 |  | 1.114 |  | 0.929 |  | 1.030 |  |
| P1NP |  | AL |  | 10 |  | 0 |  | 0.956 |  | 0.962 |  | 0.142 |  | 0.943 |  | .586 |  | 0.741 |  | 1.272 |  | 0.888 |  | 1.008 |  |
| P1NP |  | AH |  | 10 |  | 0 |  | 1.233 |  | 1.321 |  | 0.324 |  | 0.883 |  | .140 |  | 0.957 |  | 1.839 |  | 1.071 |  | 1.590 |  |
| P1NP |  | AL-X |  | 10 |  | 0 |  | 0.995 |  | 1.033 |  | 0.139 |  | 0.929 |  | .440 |  | 0.873 |  | 1.274 |  | 0.931 |  | 1.135 |  |
| P1NP |  | AH-X |  | 10 |  | 0 |  | 1.209 |  | 1.192 |  | 0.162 |  | 0.943 |  | .586 |  | 0.962 |  | 1.435 |  | 1.057 |  | 1.294 |  |
| P1NP |  | X |  | 10 |  | 0 |  | 1.057 |  | 1.078 |  | 0.204 |  | 0.846 |  | .052 |  | 0.868 |  | 1.450 |  | 0.933 |  | 1.096 |  |
|  | | | | | | | | | | | | | | | | | | | | | | | | | |

### Boxplots

#### CTX1

#### P1NP

## CTX1

| ANOVA - CTX1 | | | | | | | | | | | | | | | | | | | |
| --- | --- | --- | --- | --- | --- | --- | --- | --- | --- | --- | --- | --- | --- | --- | --- | --- | --- | --- | --- |
| Homogeneity Correction | | Cases | | Sum of Squares | | df | | Mean Square | | F | | p | | VS-MPR\* | | η² | | ω² | |
| None |  | Animal group |  | 0.638 |  | 6.000 |  | 0.106 |  | 1.834 |  | .107 |  | 1.538 |  | 0.151 |  | 0.068 |  |
|  |  | Residuals |  | 3.595 |  | 62.000 |  | 0.058 |  |  |  |  |  |  |  |  |  |  |  |
| Welch |  | Animal group |  | 0.638 |  | 6.000 |  | 0.106 |  | 1.720 |  | .154 |  | 1.275 |  | 0.151 |  | 0.068 |  |
|  |  | Residuals |  | 3.595 |  | 27.142 |  | 0.132 |  |  |  |  |  |  |  |  |  |  |  |
|  | | | | | | | | | | | | | | | | | | | |
|  |  |  |  |  |  |  |  |  |  |  |  |  |  |  |  |  |  |  |  |
| --- | --- | --- | --- | --- | --- | --- | --- | --- | --- | --- | --- | --- | --- | --- | --- | --- | --- | --- | --- |
| *Note.*  Type III Sum of Squares | | | | | | | | | | | | | | | | | | | |
| \* Vovk-Sellke Maximum *p* -Ratio: Based on the *p* -value, the maximum possible odds in favor of H₁ over H₀ equals 1/(-e *p* log(*p* )) for *p* ≤ .37 (Sellke, Bayarri, & Berger, 2001). | | | | | | | | | | | | | | | | | | | |

### Descriptives

| Descriptives - CTX1 | | | | | | | | | | | |
| --- | --- | --- | --- | --- | --- | --- | --- | --- | --- | --- | --- |
| Animal group | | N | | Mean | | SD | | SE | | Coefficient of variation | |
| C |  | 10 |  | 1.000 |  | 0.139 |  | 0.044 |  | 0.139 |  |
| OV |  | 10 |  | 0.908 |  | 0.210 |  | 0.066 |  | 0.231 |  |
| AL |  | 10 |  | 0.977 |  | 0.167 |  | 0.053 |  | 0.171 |  |
| AH |  | 9 |  | 1.067 |  | 0.402 |  | 0.134 |  | 0.377 |  |
| AL-X |  | 10 |  | 1.084 |  | 0.244 |  | 0.077 |  | 0.226 |  |
| AH-X |  | 10 |  | 0.912 |  | 0.206 |  | 0.065 |  | 0.226 |  |
| X |  | 10 |  | 1.197 |  | 0.249 |  | 0.079 |  | 0.208 |  |
|  | | | | | | | | | | | |

### Assumption Checks

| Test for Equality of Variances (Levene's) | | | | | | | | | |
| --- | --- | --- | --- | --- | --- | --- | --- | --- | --- |
| F | | df1 | | df2 | | p | | VS-MPR\* | |
| 3.657 |  | 6.000 |  | 62.00 |  | .004 |  | 18.25 |  |
|  | | | | | | | | | |
|  |  |  |  |  |  |  |  |  |  |
| --- | --- | --- | --- | --- | --- | --- | --- | --- | --- |
| \* Vovk-Sellke Maximum *p* -Ratio: Based on the *p* -value, the maximum possible odds in favor of H₁ over H₀ equals 1/(-e *p* log(*p* )) for *p* ≤ .37 (Sellke, Bayarri, & Berger, 2001). | | | | | | | | | |

## P1NP

| ANOVA - P1NP | | | | | | | | | | | | | | | | | | | |
| --- | --- | --- | --- | --- | --- | --- | --- | --- | --- | --- | --- | --- | --- | --- | --- | --- | --- | --- | --- |
| Homogeneity Correction | | Cases | | Sum of Squares | | df | | Mean Square | | F | | p | | VS-MPR\* | | η² | | ω² | |
| None |  | Animal group |  | 1.023 |  | 6.000 |  | 0.171 |  | 5.264 |  | < .001 |  | 221.33 |  | 0.338 |  | 0.271 |  |
|  |  | Residuals |  | 2.009 |  | 62.000 |  | 0.032 |  |  |  |  |  |  |  |  |  |  |  |
| Welch |  | Animal group |  | 1.023 |  | 6.000 |  | 0.171 |  | 3.872 |  | .006 |  | 11.38 |  | 0.338 |  | 0.271 |  |
|  |  | Residuals |  | 2.009 |  | 27.187 |  | 0.074 |  |  |  |  |  |  |  |  |  |  |  |
|  | | | | | | | | | | | | | | | | | | | |
|  |  |  |  |  |  |  |  |  |  |  |  |  |  |  |  |  |  |  |  |
| --- | --- | --- | --- | --- | --- | --- | --- | --- | --- | --- | --- | --- | --- | --- | --- | --- | --- | --- | --- |
| *Note.*  Type III Sum of Squares | | | | | | | | | | | | | | | | | | | |
| \* Vovk-Sellke Maximum *p* -Ratio: Based on the *p* -value, the maximum possible odds in favor of H₁ over H₀ equals 1/(-e *p* log(*p* )) for *p* ≤ .37 (Sellke, Bayarri, & Berger, 2001). | | | | | | | | | | | | | | | | | | | |

### Descriptives

| Descriptives - P1NP | | | | | | | | | | | |
| --- | --- | --- | --- | --- | --- | --- | --- | --- | --- | --- | --- |
| Animal group | | N | | Mean | | SD | | SE | | Coefficient of variation | |
| C |  | 10 |  | 1.000 |  | 0.084 |  | 0.027 |  | 0.084 |  |
| OV |  | 9 |  | 0.980 |  | 0.069 |  | 0.023 |  | 0.071 |  |
| AL |  | 10 |  | 0.962 |  | 0.142 |  | 0.045 |  | 0.148 |  |
| AH |  | 10 |  | 1.321 |  | 0.324 |  | 0.102 |  | 0.245 |  |
| AL-X |  | 10 |  | 1.033 |  | 0.139 |  | 0.044 |  | 0.134 |  |
| AH-X |  | 10 |  | 1.192 |  | 0.162 |  | 0.051 |  | 0.135 |  |
| X |  | 10 |  | 1.078 |  | 0.204 |  | 0.064 |  | 0.189 |  |
|  | | | | | | | | | | | |

### Assumption Checks

| Test for Equality of Variances (Levene's) | | | | | | | | | |
| --- | --- | --- | --- | --- | --- | --- | --- | --- | --- |
| F | | df1 | | df2 | | p | | VS-MPR\* | |
| 4.483 |  | 6.000 |  | 62.00 |  | < .001 |  | 65.55 |  |
|  | | | | | | | | | |
|  |  |  |  |  |  |  |  |  |  |
| --- | --- | --- | --- | --- | --- | --- | --- | --- | --- |
| \* Vovk-Sellke Maximum *p* -Ratio: Based on the *p* -value, the maximum possible odds in favor of H₁ over H₀ equals 1/(-e *p* log(*p* )) for *p* ≤ .37 (Sellke, Bayarri, & Berger, 2001). | | | | | | | | | |

### Post Hoc Tests

#### Standard (HSD)

| Post Hoc Comparisons - Animal group | | | | | | | | | | | | | | | | | | | |
| --- | --- | --- | --- | --- | --- | --- | --- | --- | --- | --- | --- | --- | --- | --- | --- | --- | --- | --- | --- |
|  | | | | | | 95% CI for Mean Difference | | | |  | | | | | | | | | |
|  | |  | | Mean Difference | | Lower | | Upper | | SE | | df | | t | | ptukey | | pbonf | |
| C |  | OV |  | 0.020 |  | -0.232 |  | 0.272 |  | 0.083 |  | 62 |  | 0.239 |  | 1.000 |  | 1.000 |  |
|  |  | AL |  | 0.038 |  | -0.207 |  | 0.284 |  | 0.080 |  | 62 |  | 0.476 |  | .999 |  | 1.000 |  |
|  |  | AH |  | -0.321 |  | -0.566 |  | -0.076 |  | 0.080 |  | 62 |  | -3.990 |  | .003 | \*\* | .004 | \*\* |
|  |  | (AL-X) |  | -0.033 |  | -0.278 |  | 0.213 |  | 0.080 |  | 62 |  | -0.406 |  | 1.000 |  | 1.000 |  |
|  |  | (AH-X) |  | -0.192 |  | -0.437 |  | 0.053 |  | 0.080 |  | 62 |  | -2.388 |  | .221 |  | .420 |  |
|  |  | X |  | -0.078 |  | -0.323 |  | 0.168 |  | 0.080 |  | 62 |  | -0.964 |  | .960 |  | 1.000 |  |
| OV |  | AL |  | 0.019 |  | -0.233 |  | 0.271 |  | 0.083 |  | 62 |  | 0.224 |  | 1.000 |  | 1.000 |  |
|  |  | AH |  | -0.341 |  | -0.593 |  | -0.089 |  | 0.083 |  | 62 |  | -4.123 |  | .002 | \*\* | .002 | \*\* |
|  |  | (AL-X) |  | -0.052 |  | -0.304 |  | 0.200 |  | 0.083 |  | 62 |  | -0.635 |  | .995 |  | 1.000 |  |
|  |  | (AH-X) |  | -0.212 |  | -0.464 |  | 0.040 |  | 0.083 |  | 62 |  | -2.563 |  | .155 |  | .269 |  |
|  |  | X |  | -0.097 |  | -0.349 |  | 0.155 |  | 0.083 |  | 62 |  | -1.177 |  | .900 |  | 1.000 |  |
| AL |  | AH |  | -0.360 |  | -0.605 |  | -0.114 |  | 0.080 |  | 62 |  | -4.466 |  | < .001 | \*\*\* | < .001 | \*\*\* |
|  |  | (AL-X) |  | -0.071 |  | -0.316 |  | 0.174 |  | 0.080 |  | 62 |  | -0.882 |  | .974 |  | 1.000 |  |
|  |  | (AH-X) |  | -0.231 |  | -0.476 |  | 0.015 |  | 0.080 |  | 62 |  | -2.864 |  | .079 |  | .120 |  |
|  |  | X |  | -0.116 |  | -0.361 |  | 0.129 |  | 0.080 |  | 62 |  | -1.440 |  | .778 |  | 1.000 |  |
| AH |  | (AL-X) |  | 0.289 |  | 0.043 |  | 0.534 |  | 0.080 |  | 62 |  | 3.584 |  | .011 | \* | .014 | \* |
|  |  | (AH-X) |  | 0.129 |  | -0.116 |  | 0.374 |  | 0.080 |  | 62 |  | 1.603 |  | .681 |  | 1.000 |  |
|  |  | X |  | 0.244 |  | -0.002 |  | 0.489 |  | 0.080 |  | 62 |  | 3.026 |  | .053 |  | .076 |  |
| (AL-X) |  | (AH-X) |  | -0.160 |  | -0.405 |  | 0.086 |  | 0.080 |  | 62 |  | -1.981 |  | .437 |  | 1.000 |  |
|  |  | X |  | -0.045 |  | -0.290 |  | 0.200 |  | 0.080 |  | 62 |  | -0.558 |  | .998 |  | 1.000 |  |
| (AH-X) |  | X |  | 0.115 |  | -0.131 |  | 0.360 |  | 0.080 |  | 62 |  | 1.424 |  | .787 |  | 1.000 |  |
|  | | | | | | | | | | | | | | | | | | | |
|  |  |  |  |  |  |  |  |  |  |  |  |  |  |  |  |  |  |  |  |
| --- | --- | --- | --- | --- | --- | --- | --- | --- | --- | --- | --- | --- | --- | --- | --- | --- | --- | --- | --- |
| \* p < .05, \*\* p < .01, \*\*\* p < .001 | | | | | | | | | | | | | | | | | | | |
| *Note.*  P-value and confidence intervals adjusted for comparing a family of 7 estimates (confidence intervals corrected using the tukey method). | | | | | | | | | | | | | | | | | | | |

| Letter-Based Grouping - Animal group | | | |
| --- | --- | --- | --- |
| Animal group | | Letter | |
| C |  | a |  |
| OV |  | a |  |
| AL |  | a |  |
| AH |  | b |  |
| AL-X |  | a |  |
| AH-X |  | ab |  |
| X |  | ab |  |
|  | | | |
|  |  |  |  |
| --- | --- | --- | --- |
| *Note.*  If two or more means share the same grouping symbol, then we cannot show them to be different, but we also did not show them to be the same. | | | |
